# Supplementary material for: Synthesis and Identification of New N,N-Disubstituted Thiourea, and Thiazolidinone Scaffolds Based on Quinolone Moiety as Urease Inhibitor
Source: Molecules. 2022 Oct 21;27(20):7126. doi: 10.3390/molecules27207126 (PMC9608620; doi:10.3390/molecules27207126)
Supplement: Supplementary file 1 [file molecules-27-07126-s001.zip › molecules-1942839-supplementary.pdf]

Supplementary Material, part 1

Y.A.M.M. Elshaier, A.A. Aly,* M. Abd El-Aziz, H.M. Fathy, A.B. Brown, S. Bräse, and M. Ramadan

"Synthesis and identification of new *N*,*N*-disubstituted thiourea and thiazolidinone scaffolds based on quinolone moiety as urease inhibitors"

Contents

Compound **5g** **S1-S19**

Compound **7b** **S20-S39**

Compound **7a** **S40-S57**

Compound **7c** **S58-S75**

Compound **7d** **S76-S92**

Compound **7e** **S93-S116**

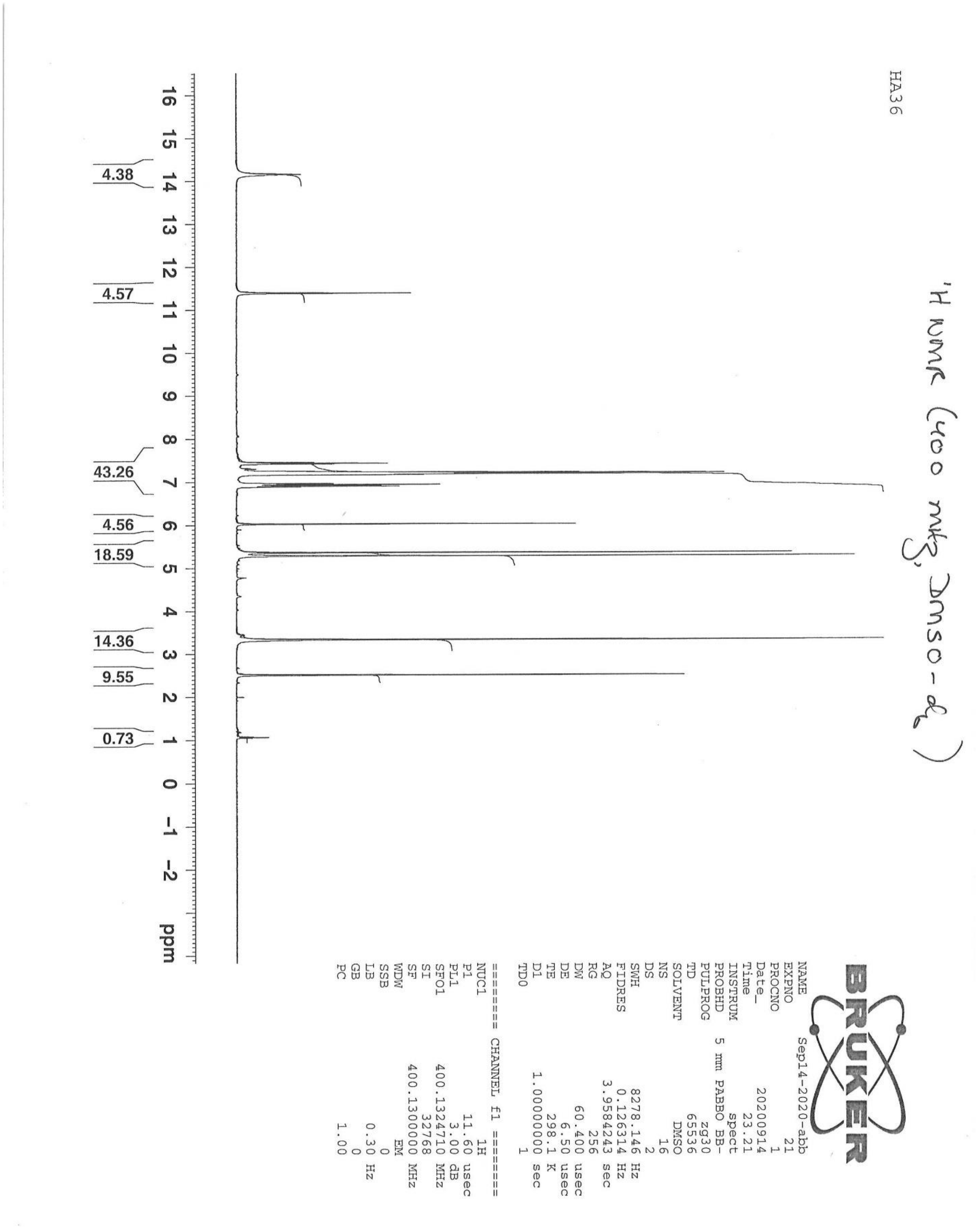

Figure S1. ^{1}H NMR spectrum of compound **5g** (400 MHz, DMSO-d_6).

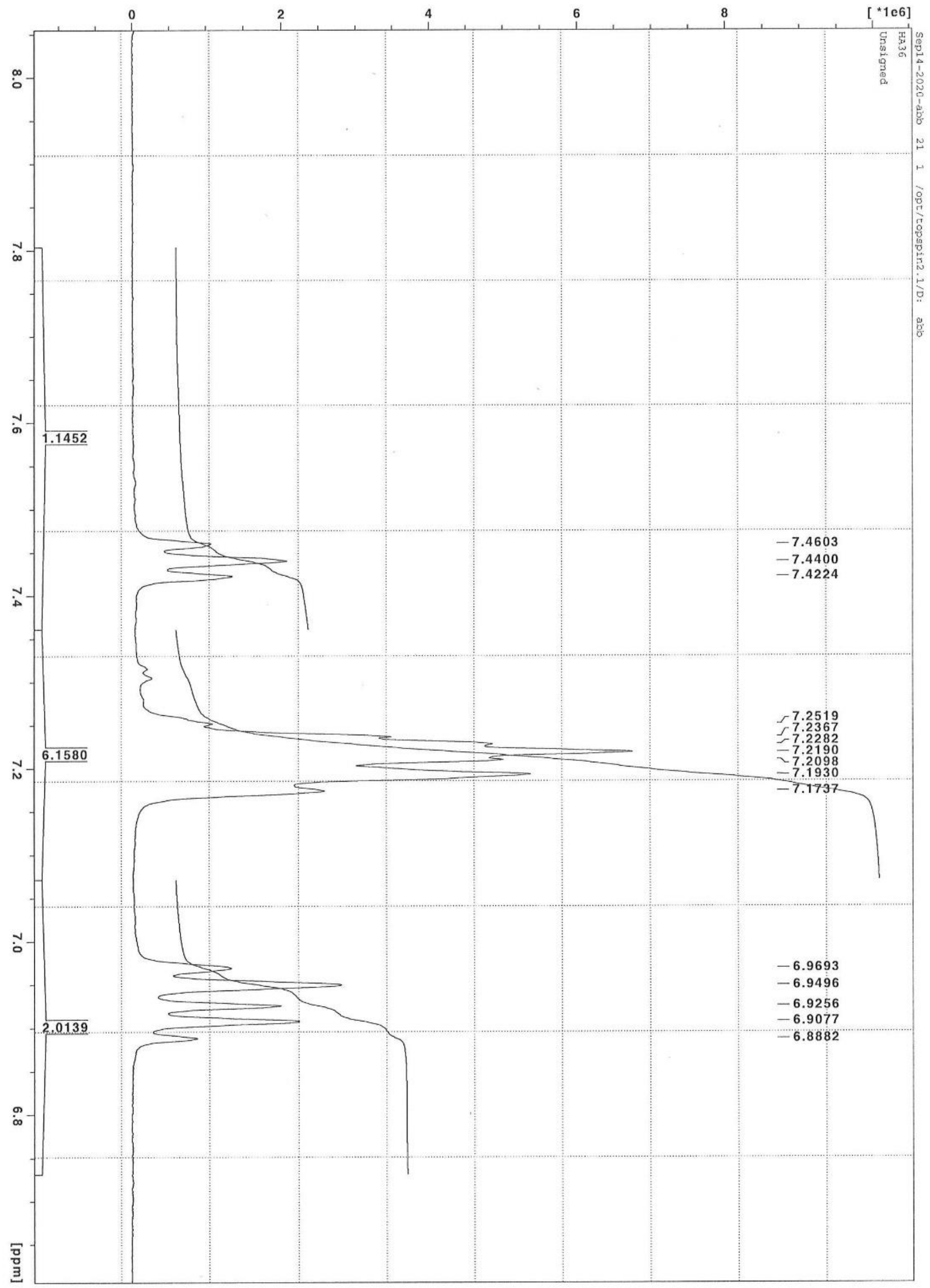

Figure S2. Detail of ^{1}H NMR spectrum of compound **5g.**

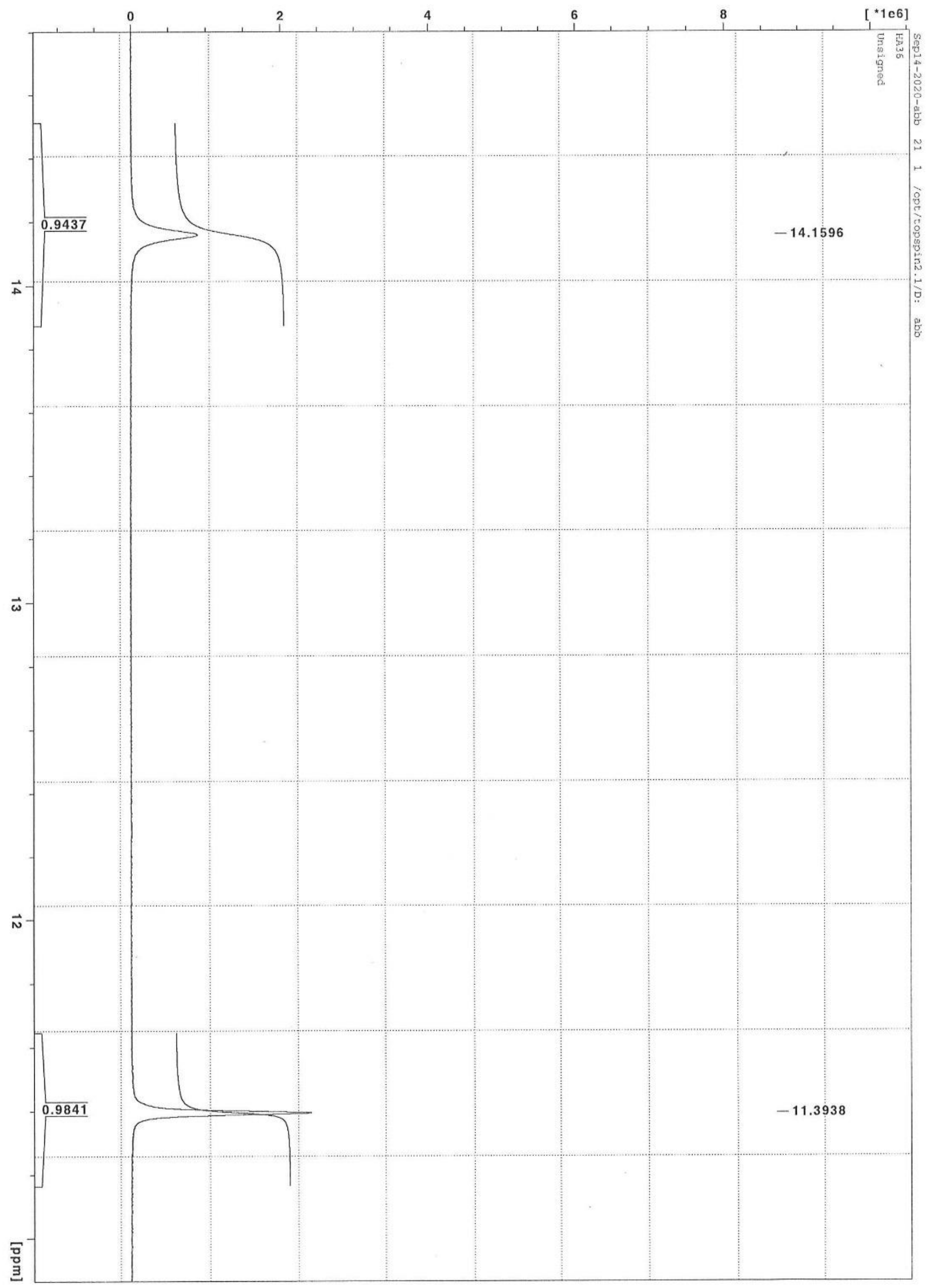

Figure S3. Detail of ^{1}H NMR spectrum of compound **5g**.

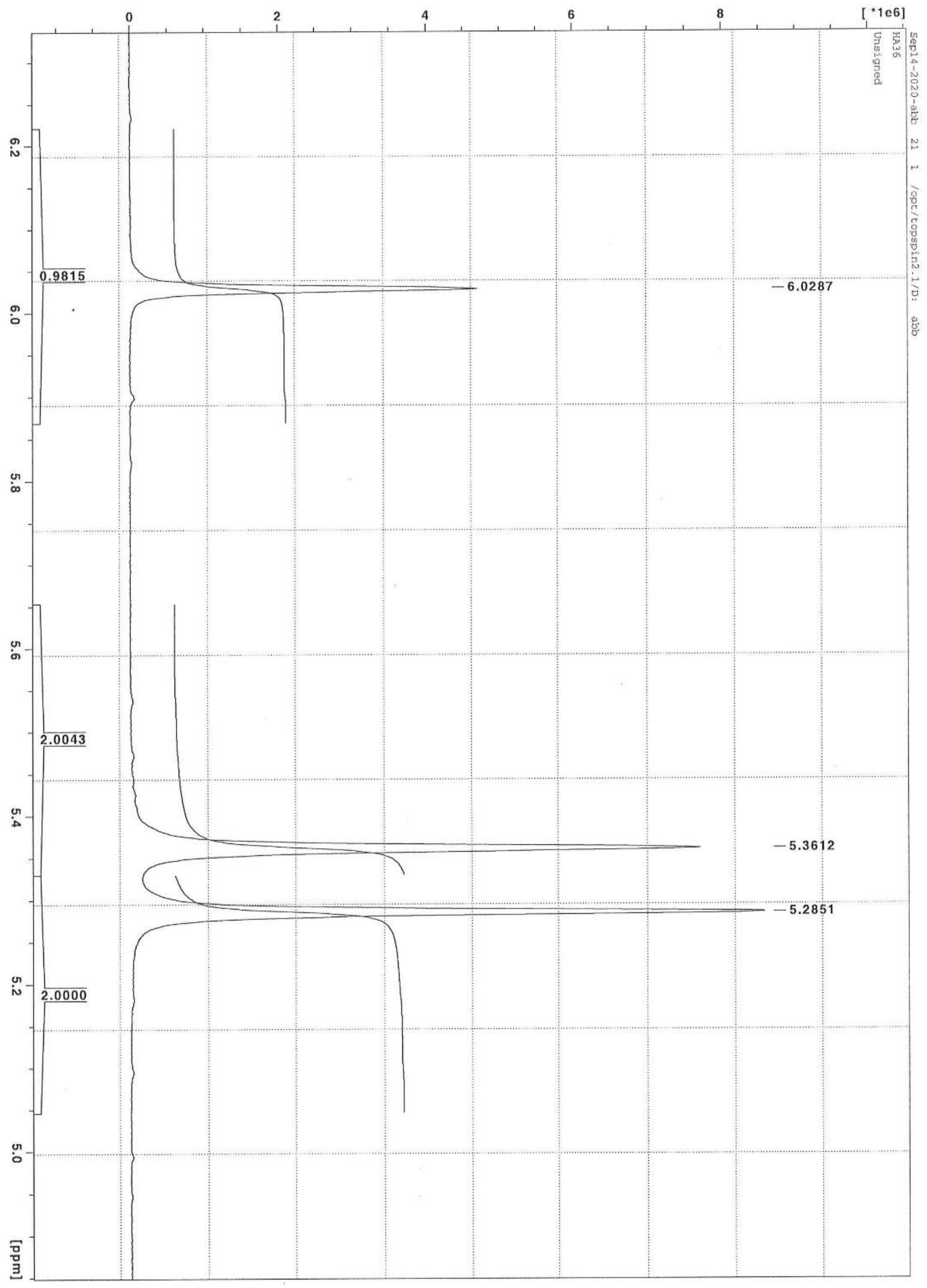

Figure S4. Detail of ^{1}H spectrum of compound **5g.**

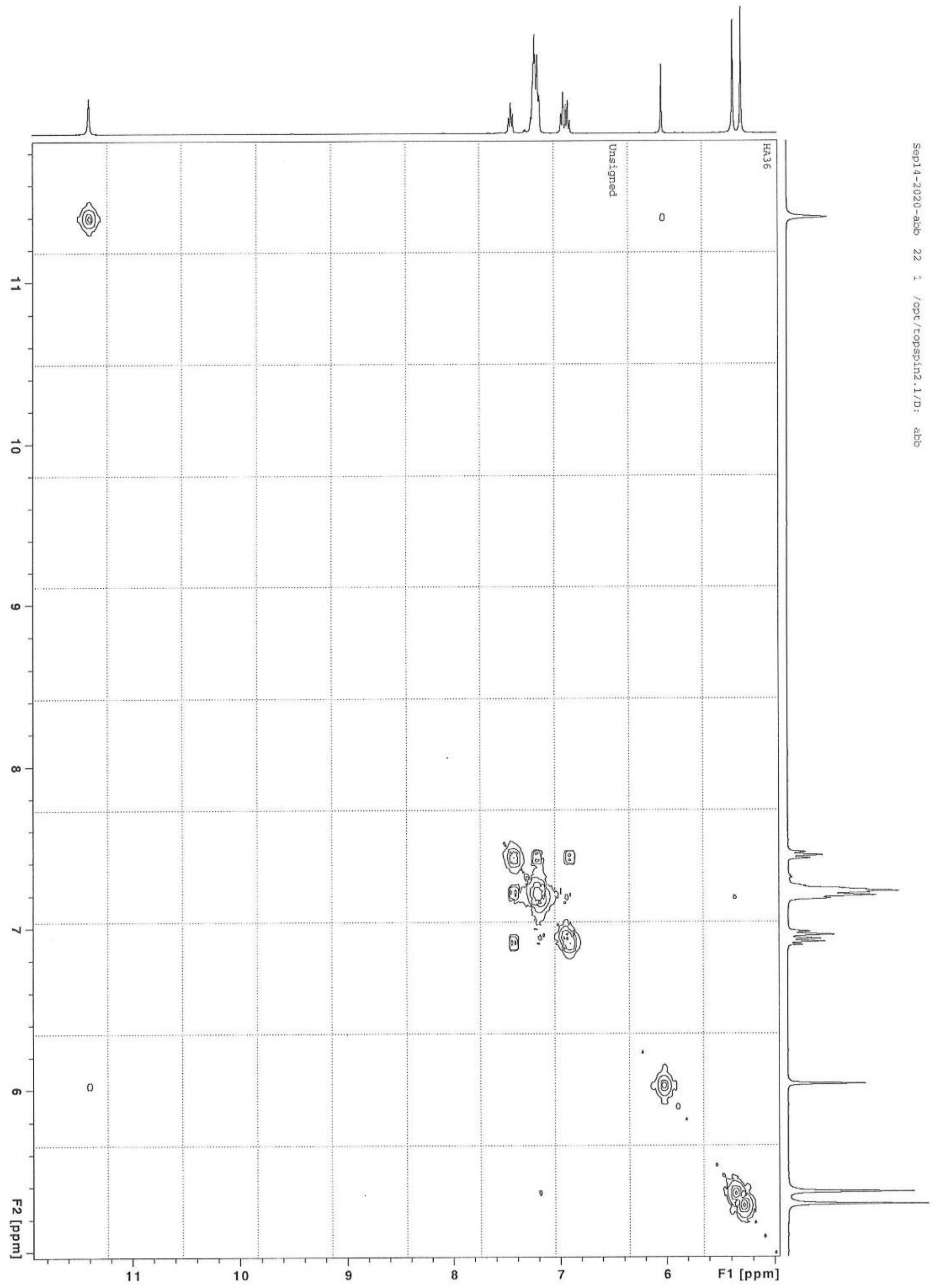

Figure S5. ^{1}H-^{1}H COSY spectrum of compound **5g.**

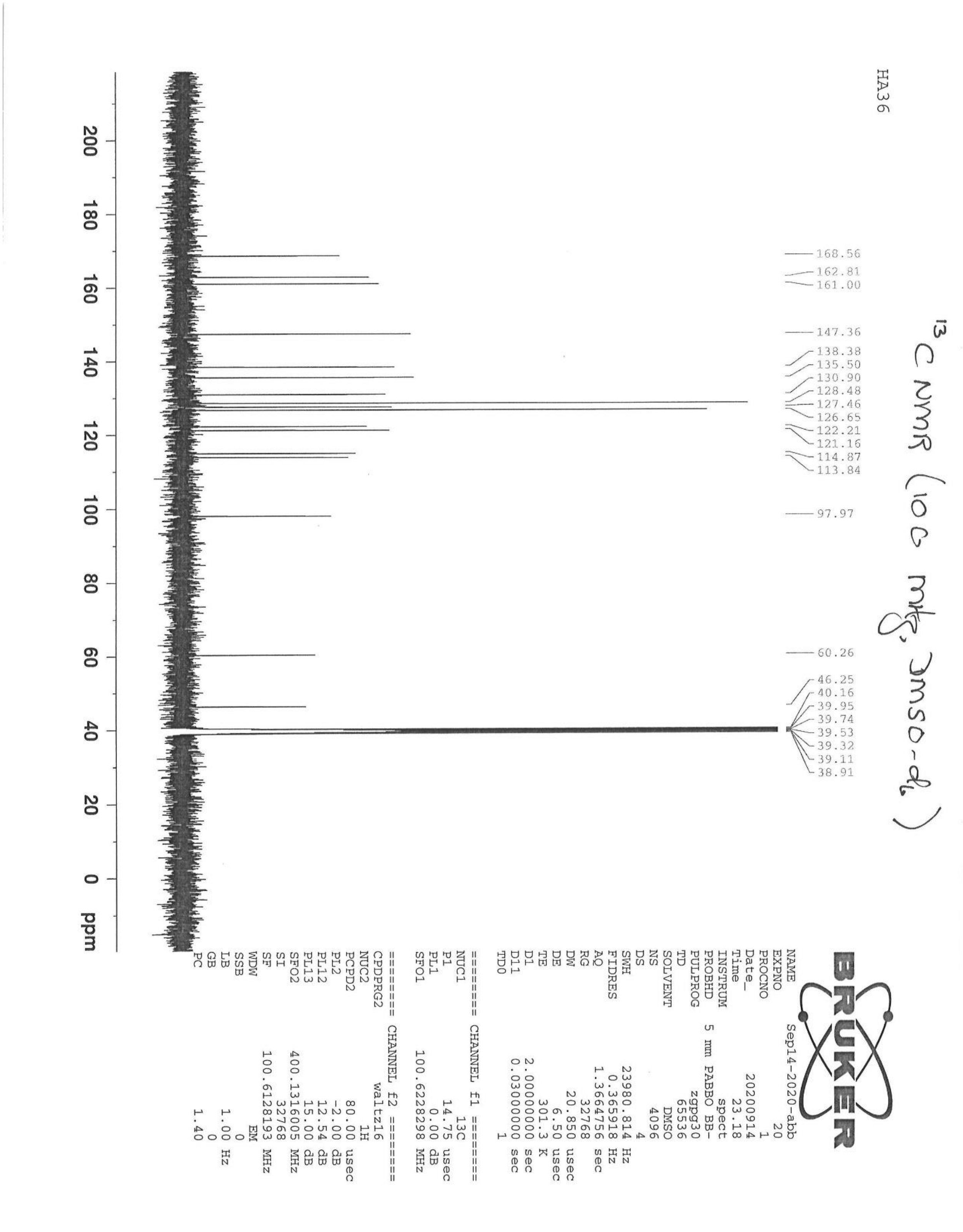

Figure S6. ^{13}C NMR spectrum of compound **5g** (100 MHz, DMSO-d_6)**.**

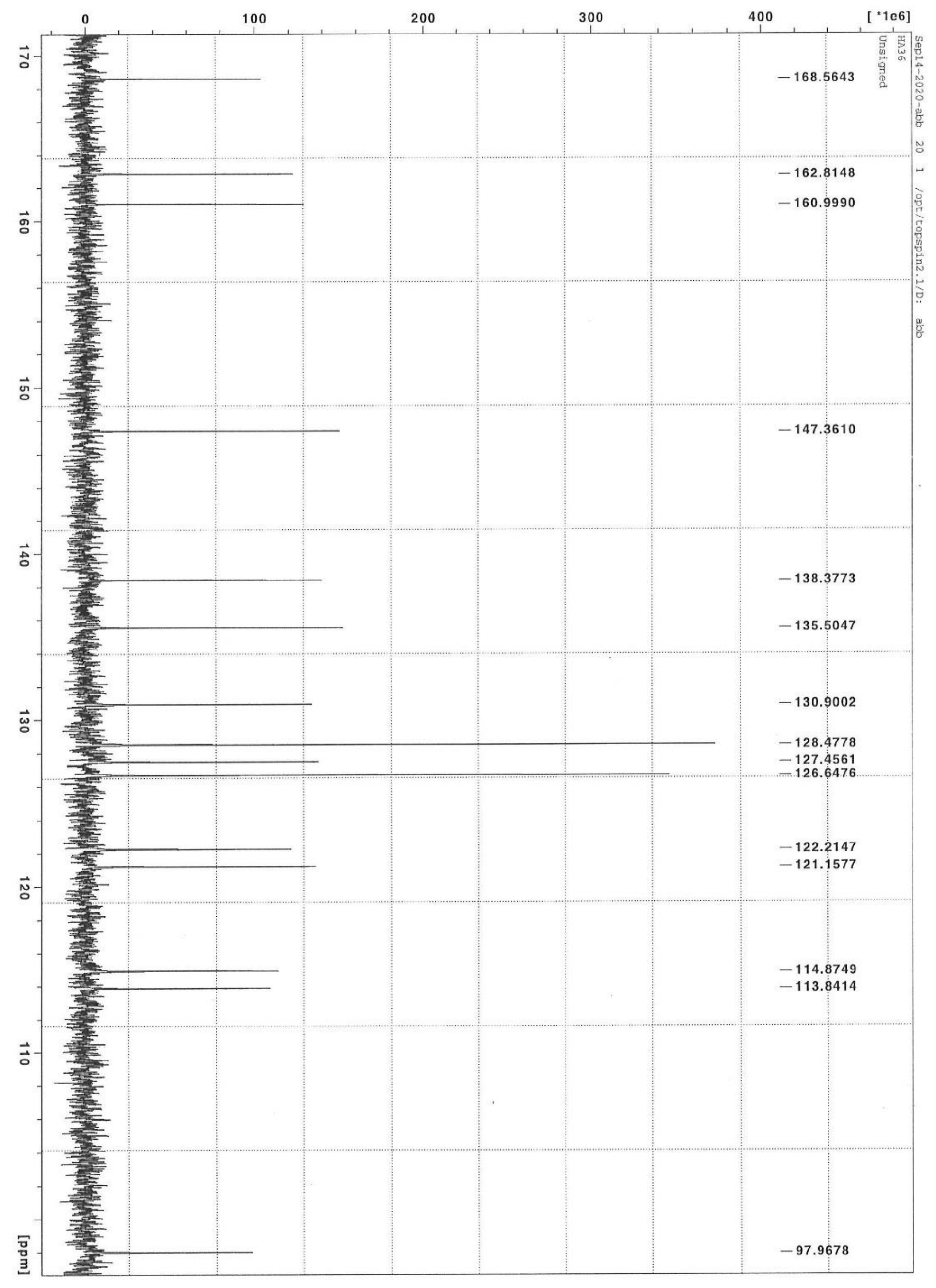

Figure S7. Detail of ^{13}C NMR spectrum of compound **5g.**

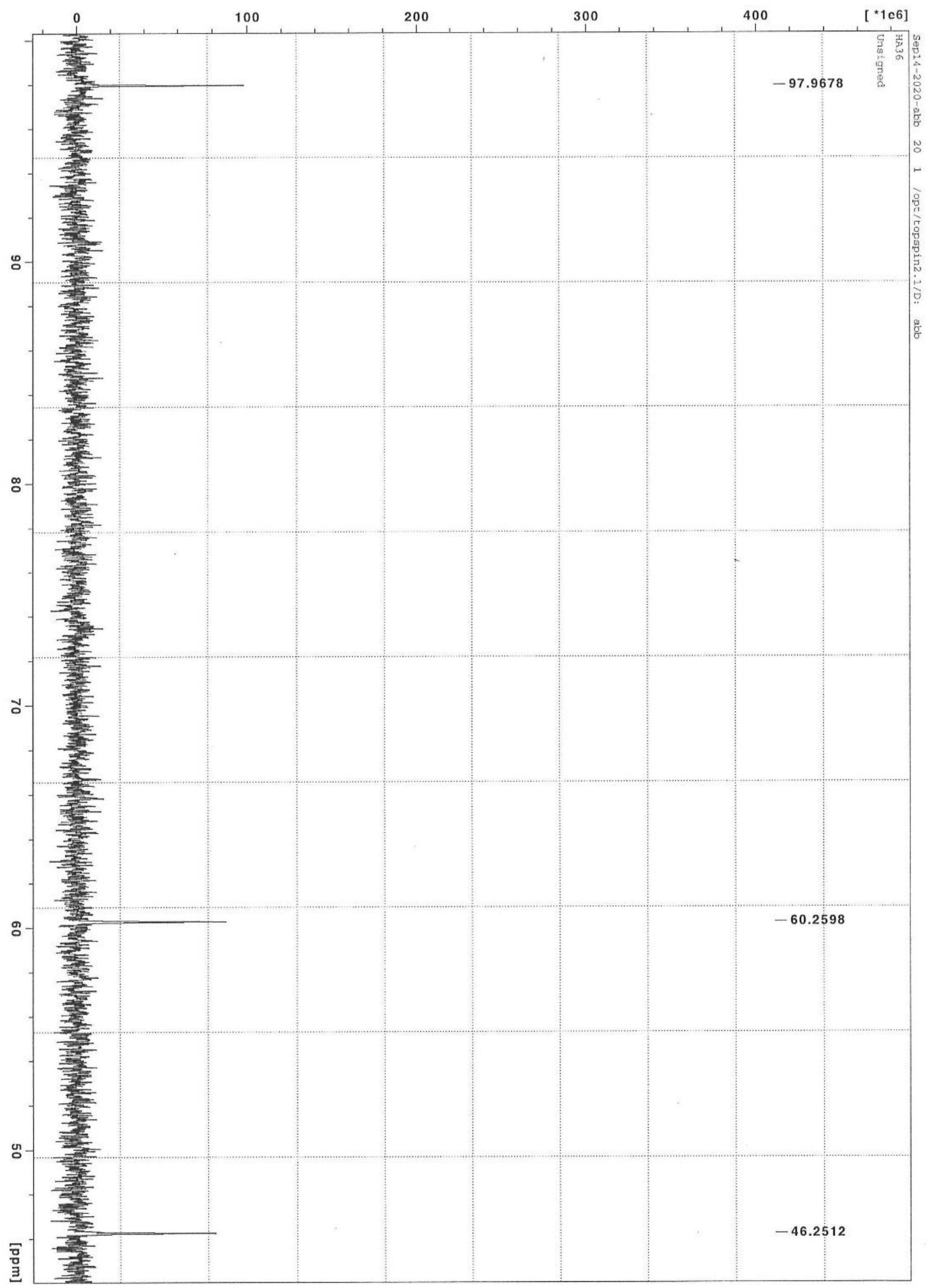

Figure S8. Detail of ^{13}C NMR spectrum of compound **5g.**.

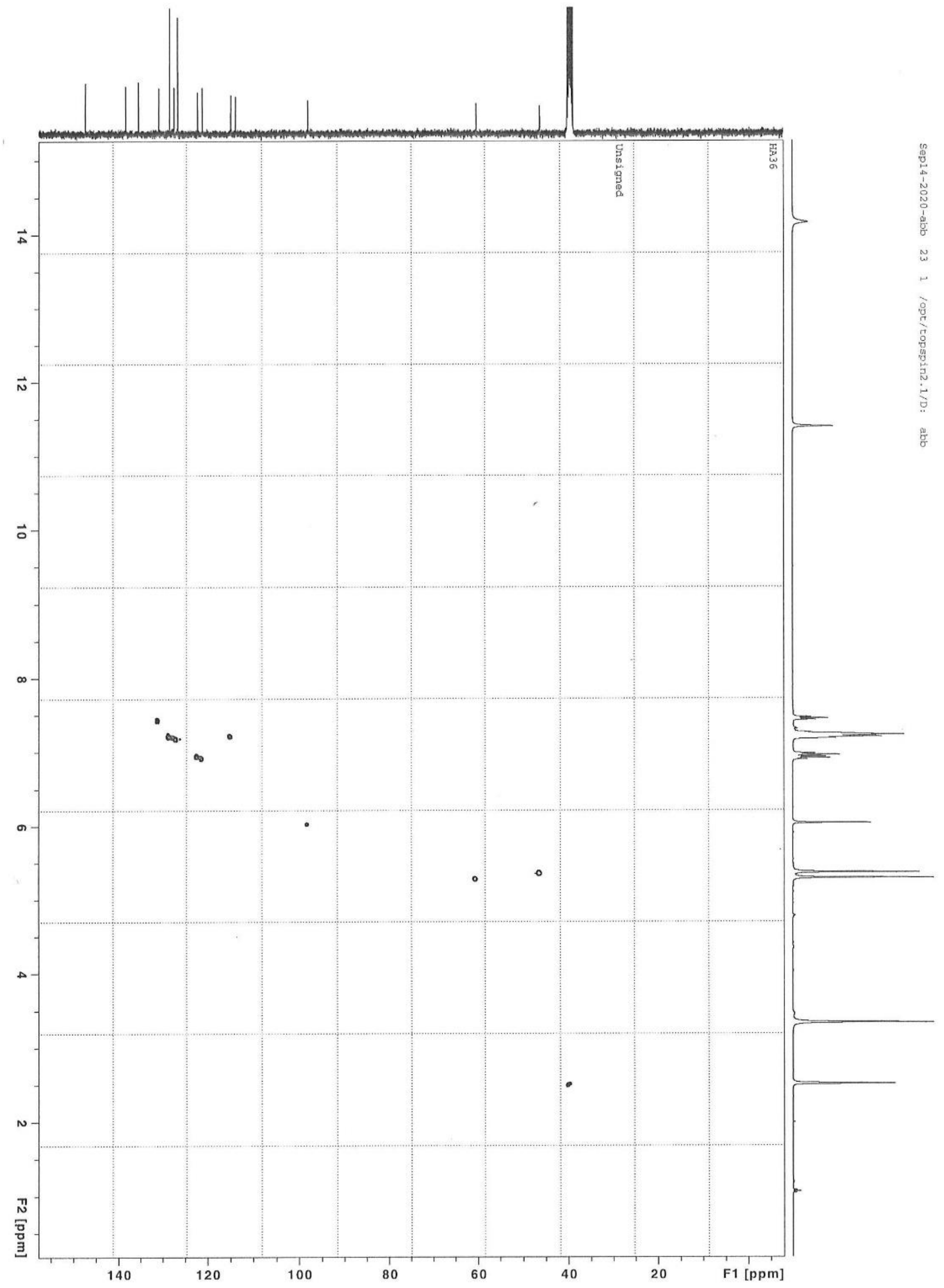

Figure S9. ^{1}H-^{13}C HSQC spectrum of compound **7a.**

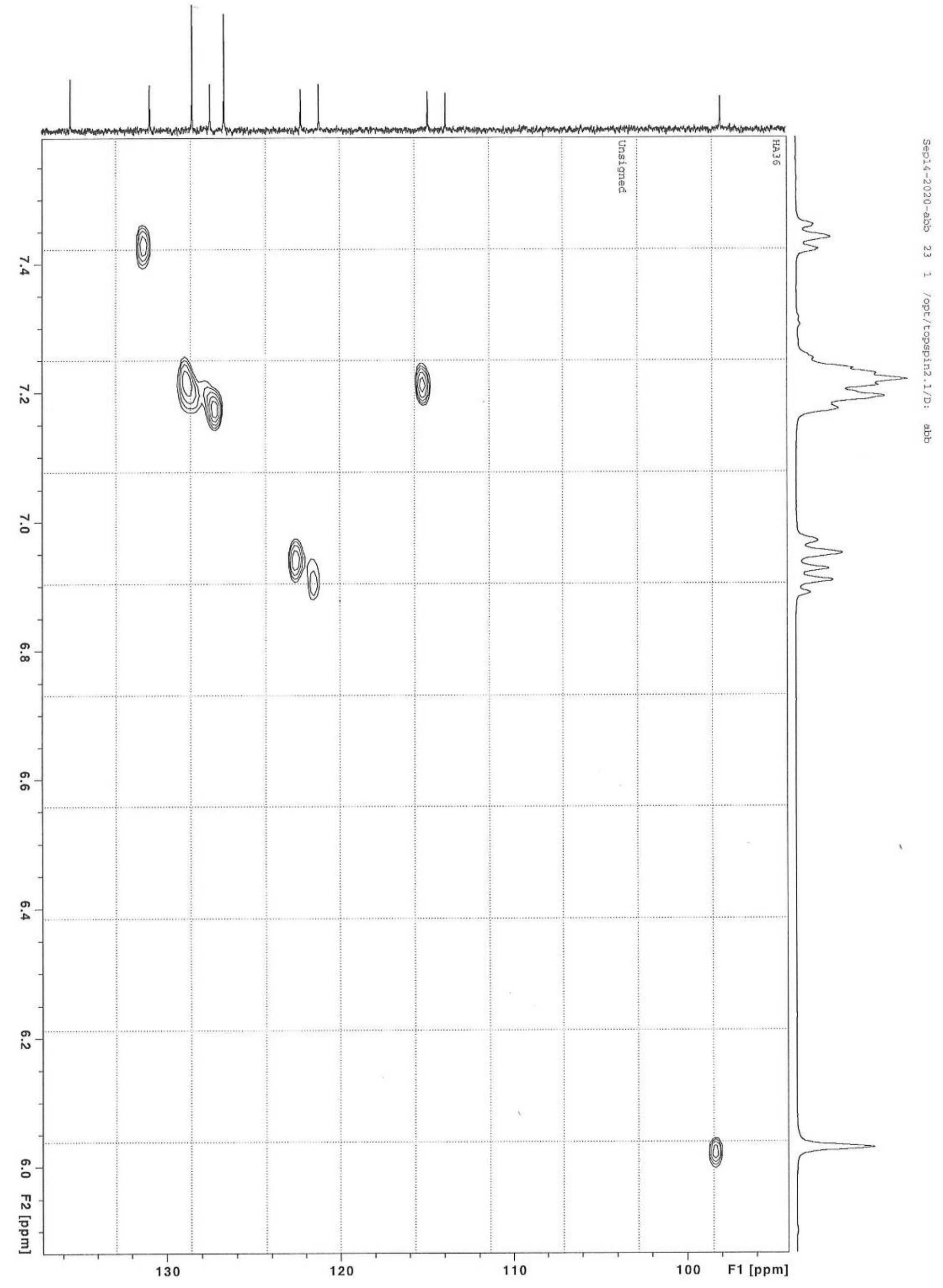

Figure S10. Detail of ^{1}H-^{13}C HSQC spectrum of compound **5g**.

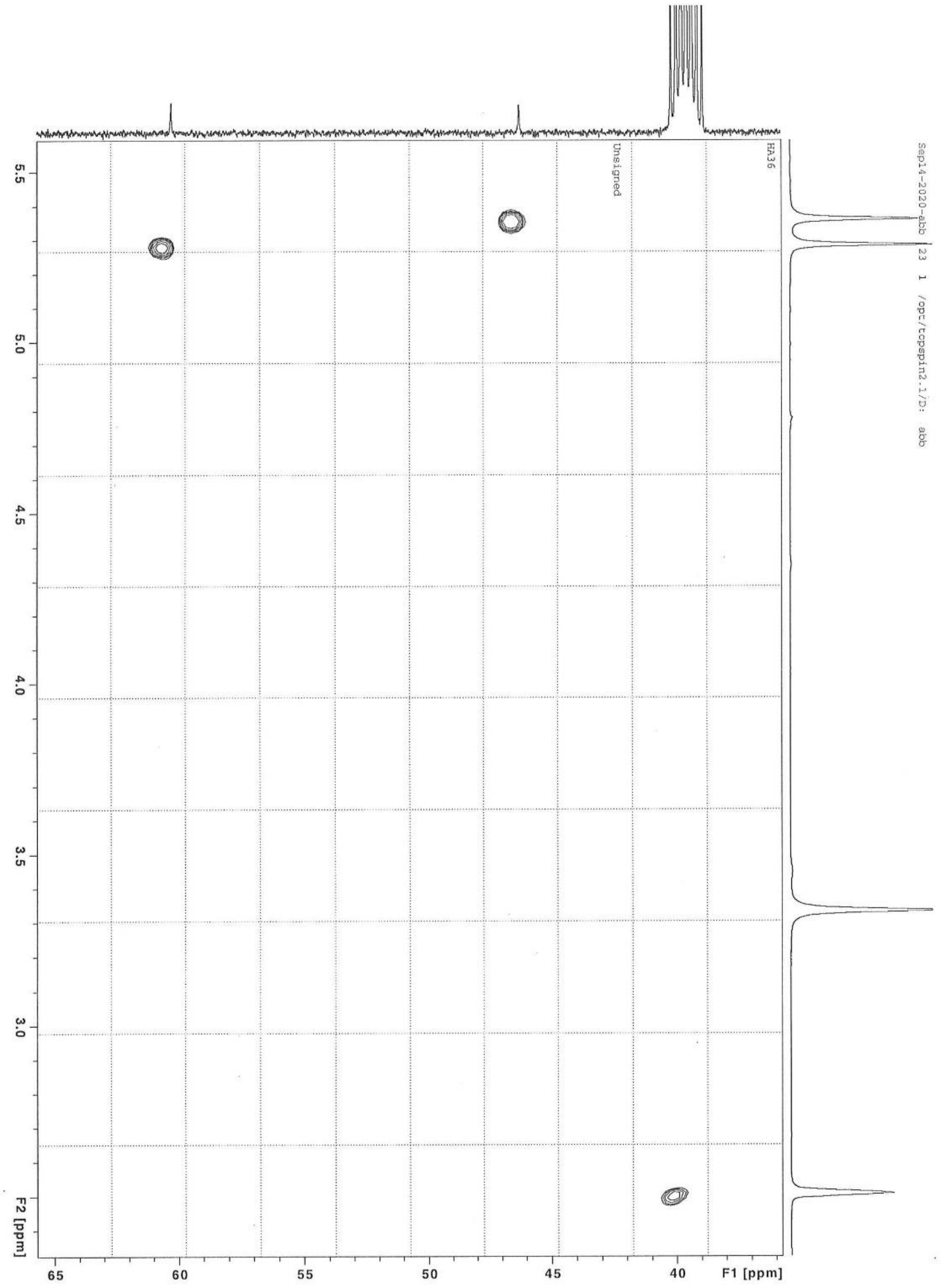

Figure S11. Detail of ^{1}H-^{13}C HSQC spectrum of compound **5g.**

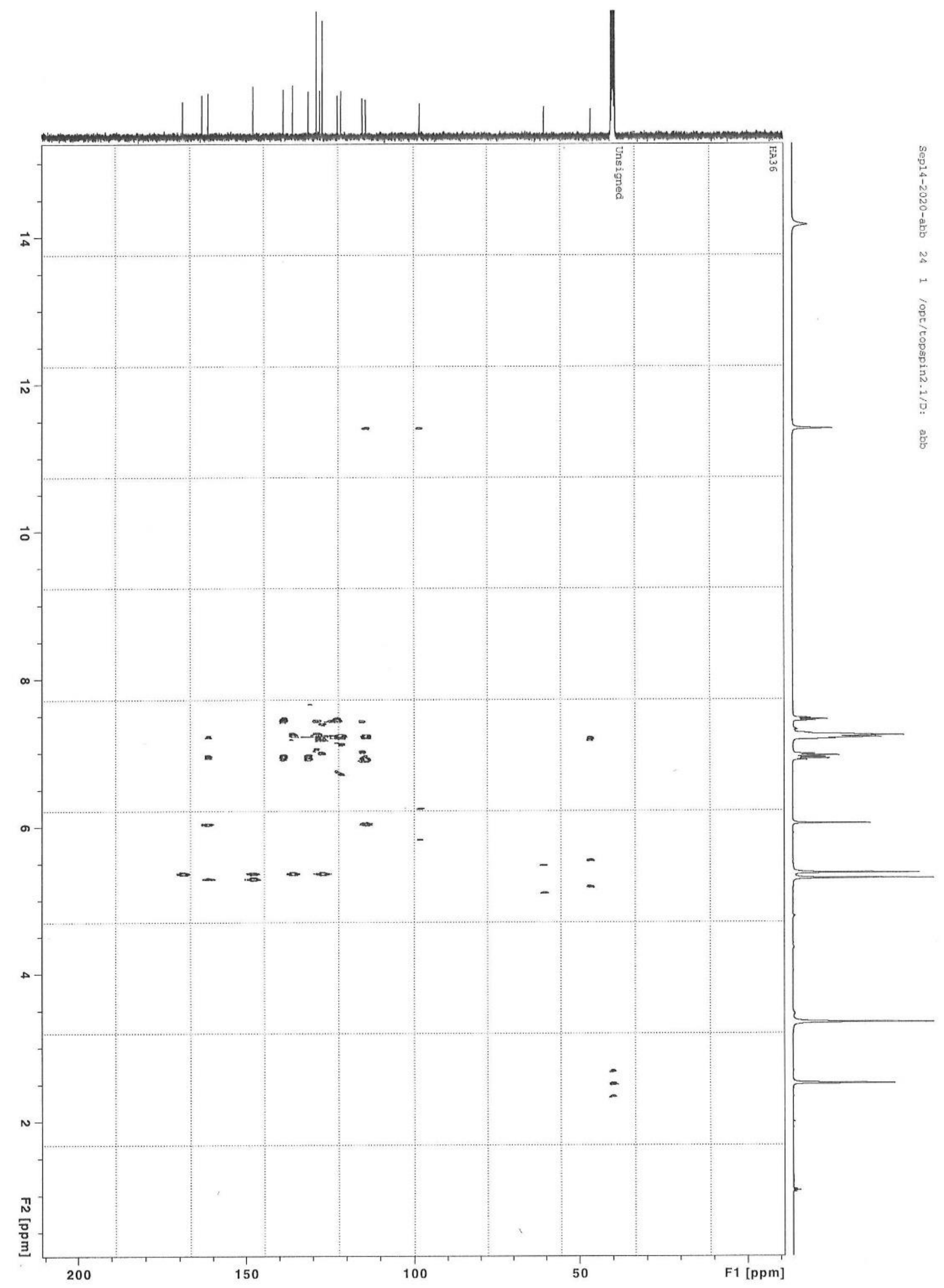

Figure S12. ^{1}H-^{13}C HMBC spectrum of compound **5g.**

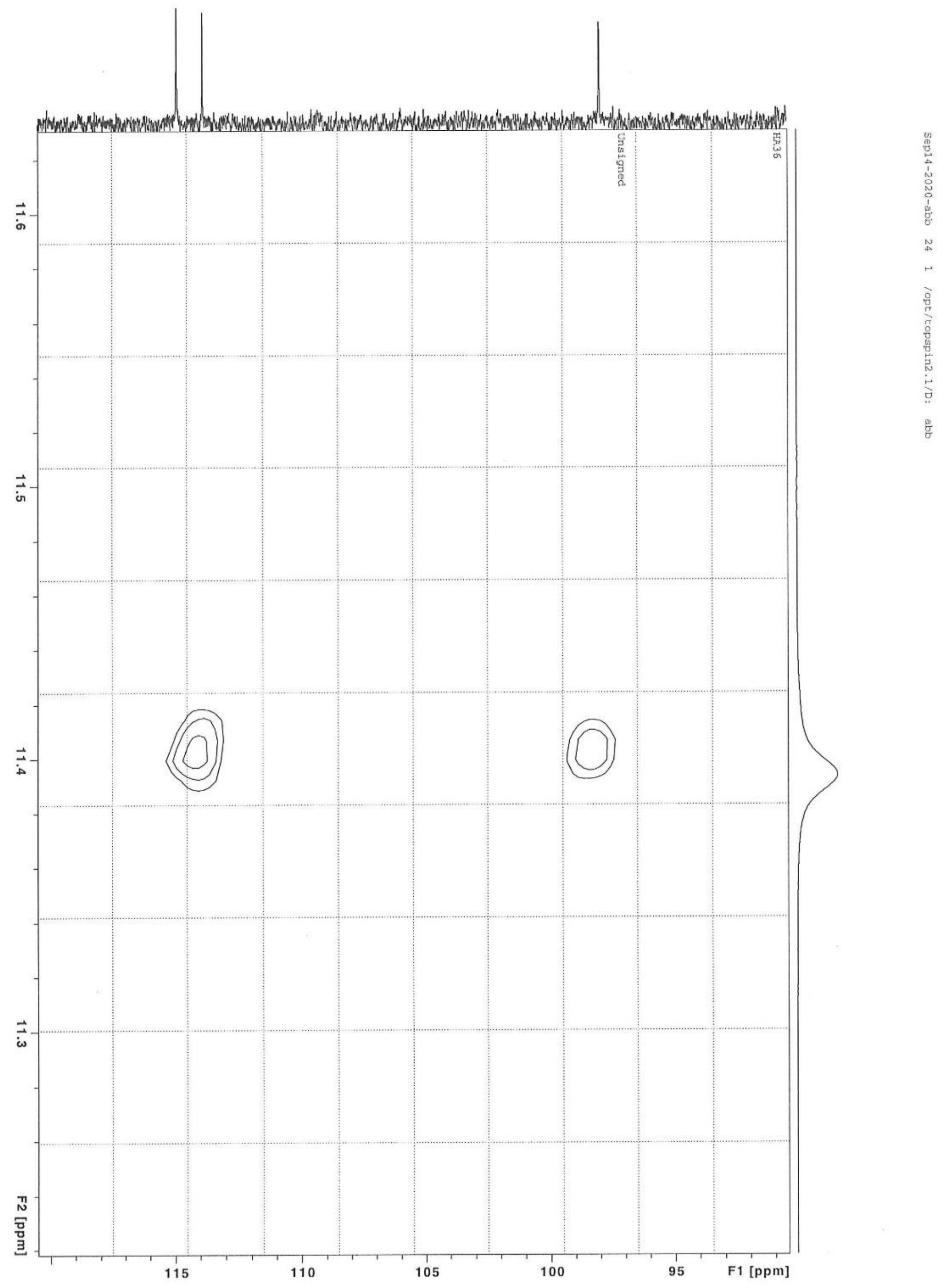

Figure S13. Detail of ^{1}H-^{13}C HMBC spectrum of compound **5g,**

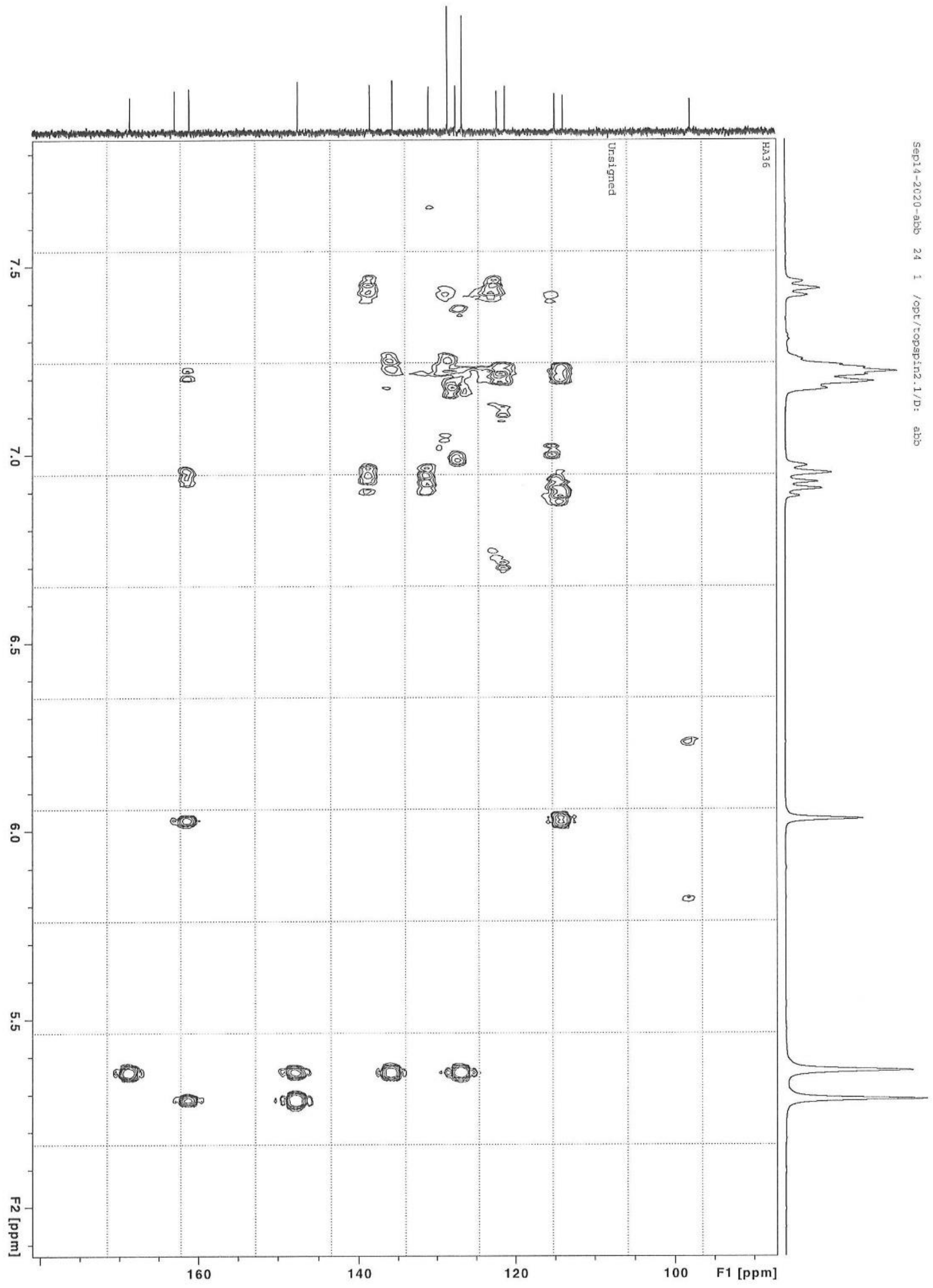

Figure S14. Detail of ^{1}H-^{13}C HMBC spectrum of compound **5g.**

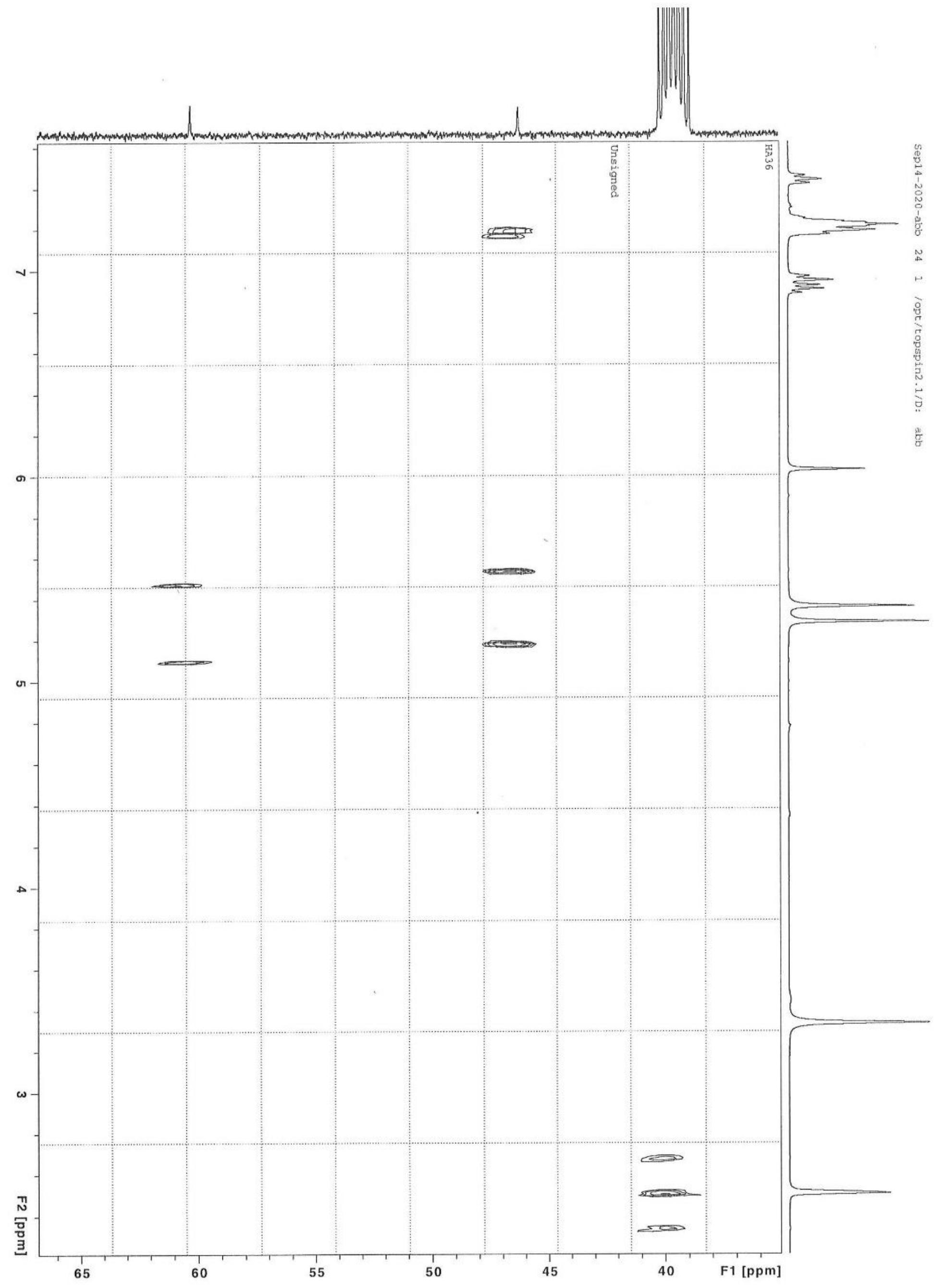

Figure S15. Detail of ^{1}H-^{13}C HMBC spectrum of compound **5g**.

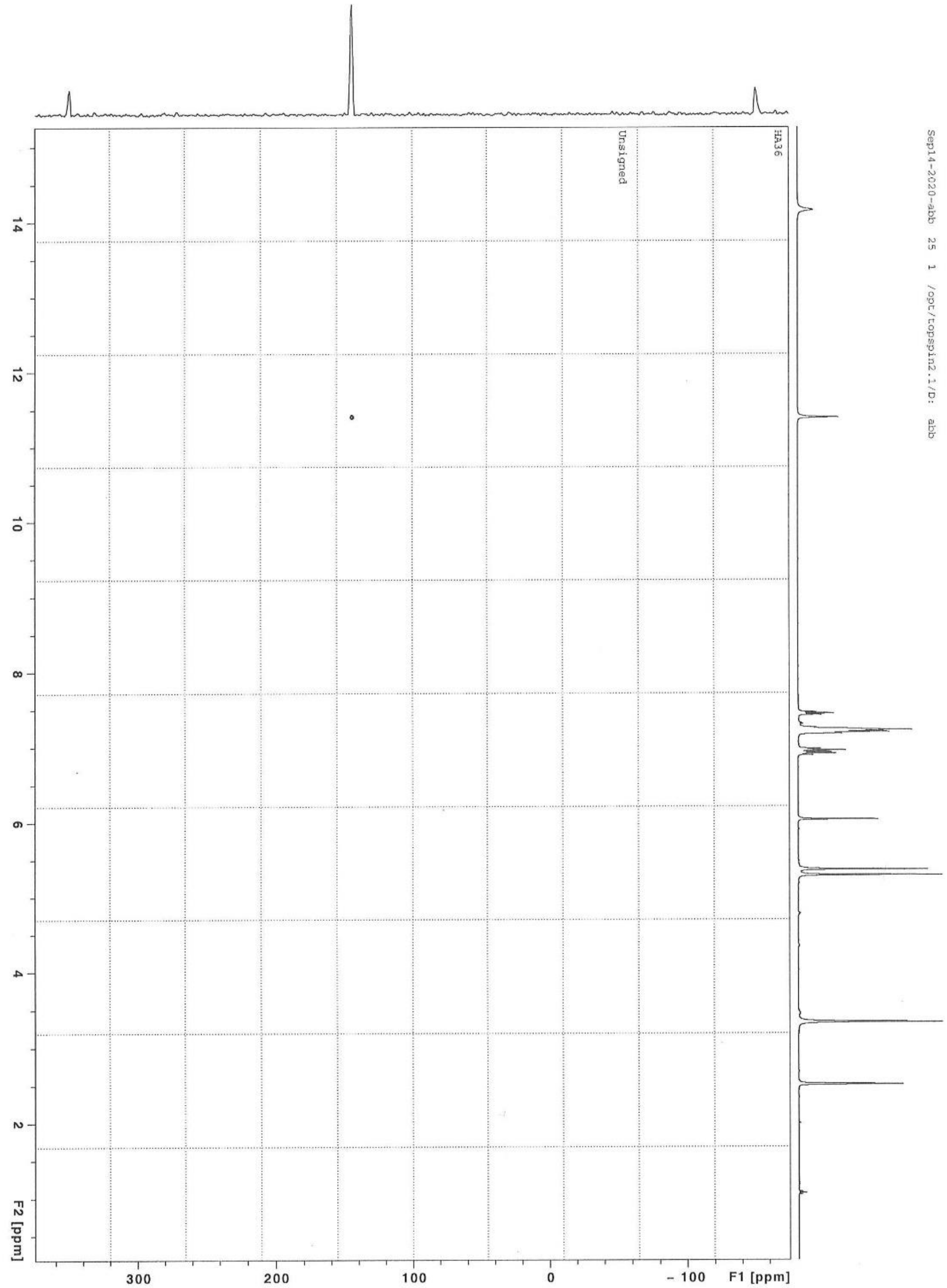

Figure S16. ^{1}H-^{15}N HSQC spectrum of compound **5g.**

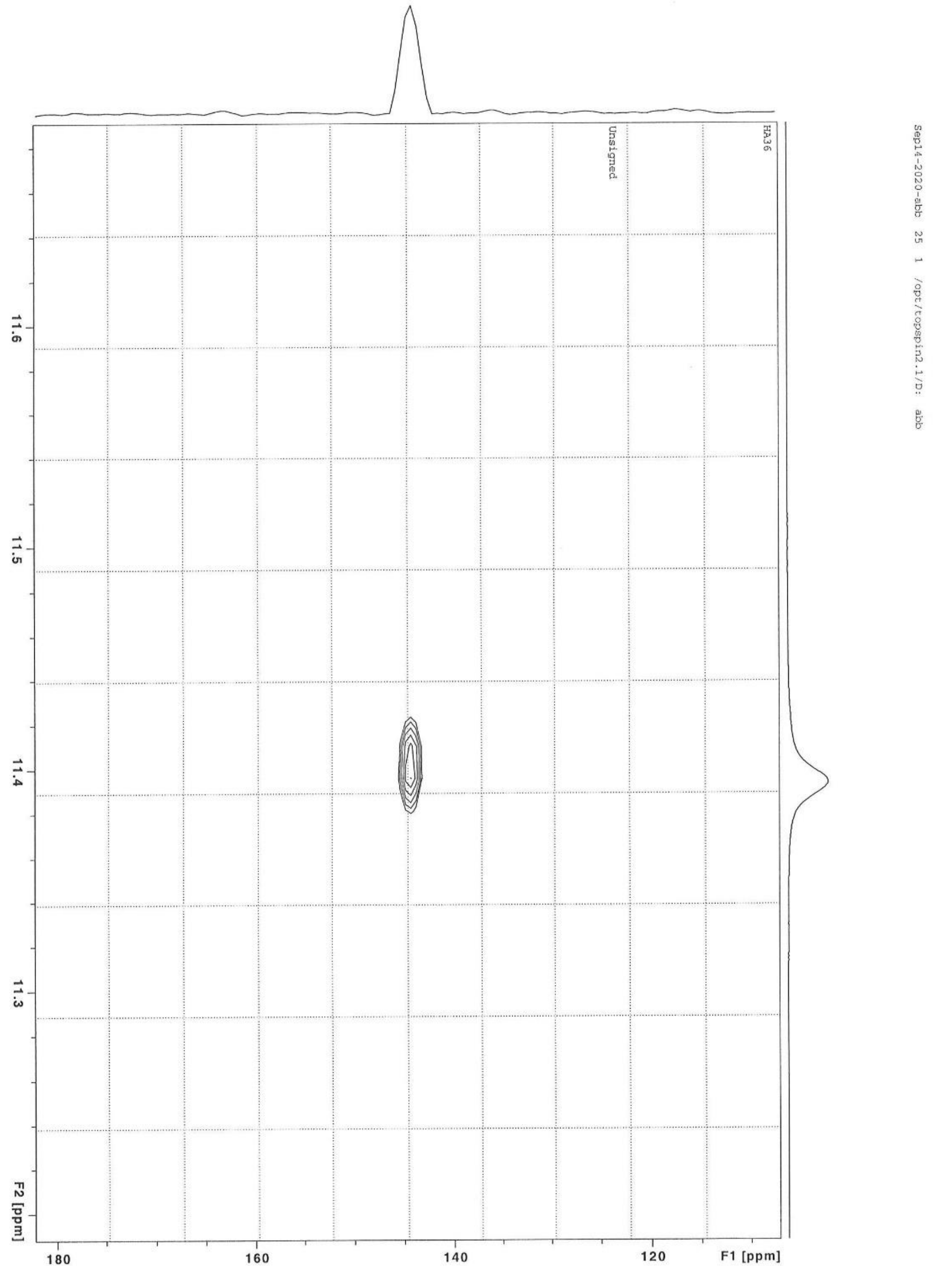

Figure S17. Detail of ^{1}H-^{15}N HSQC spectrum of compound **5g**.

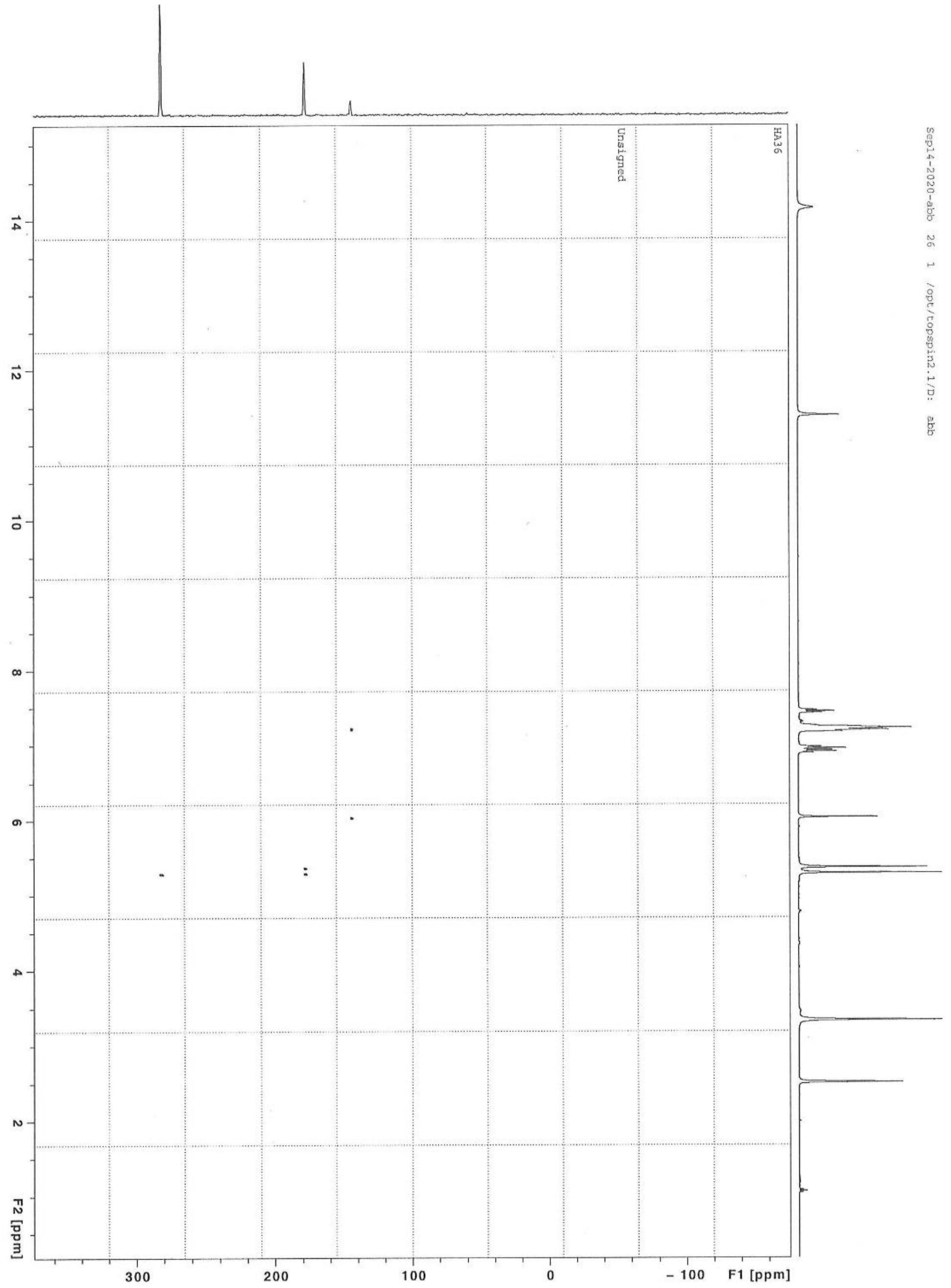

Figure S18. ^{1}H-^{15}N HMBC spectrum of compound **5g.**

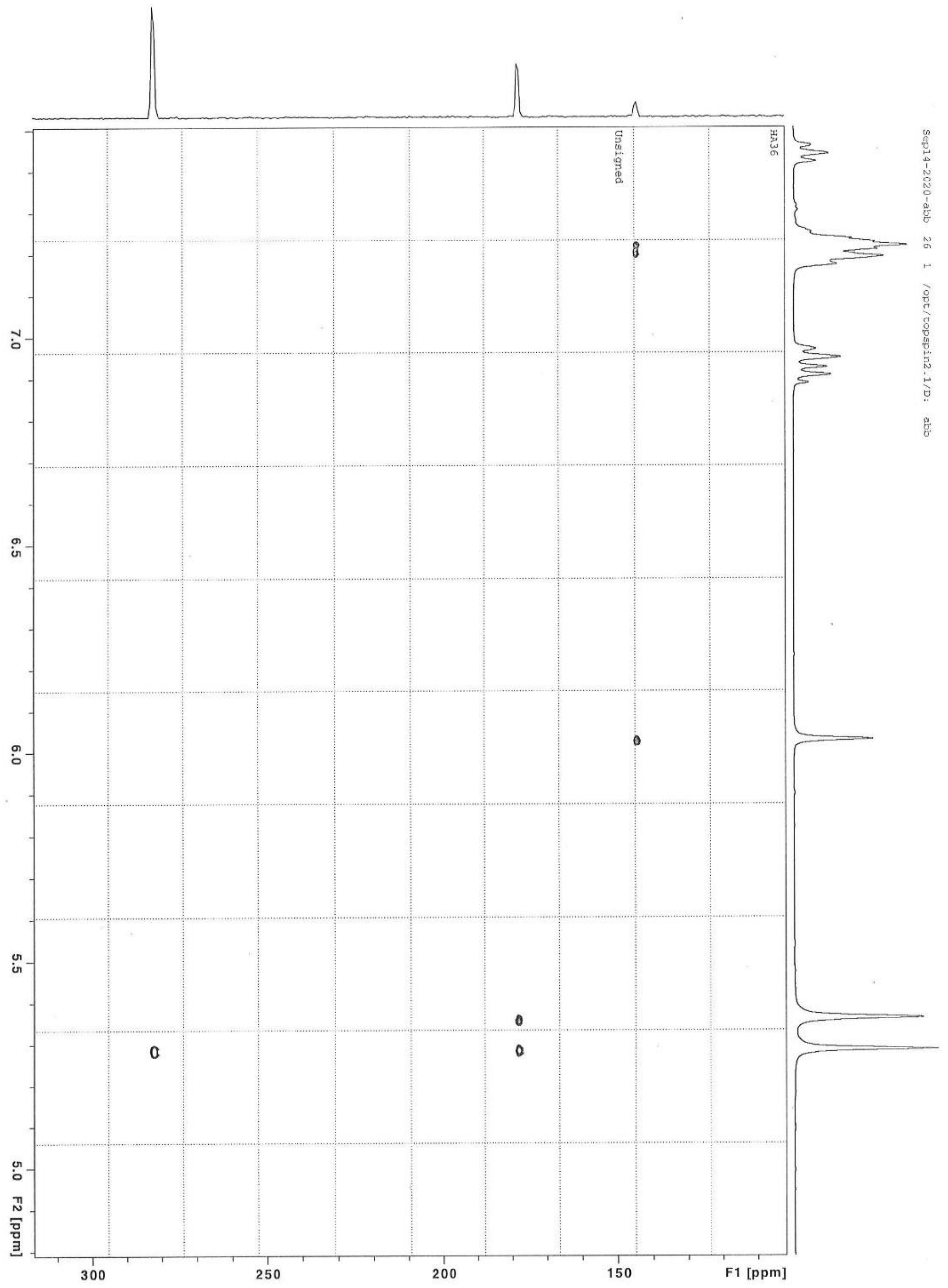

Figure S19. Detail of ^{1}H-^{15}N HMBC spectrum of compound **5g.**

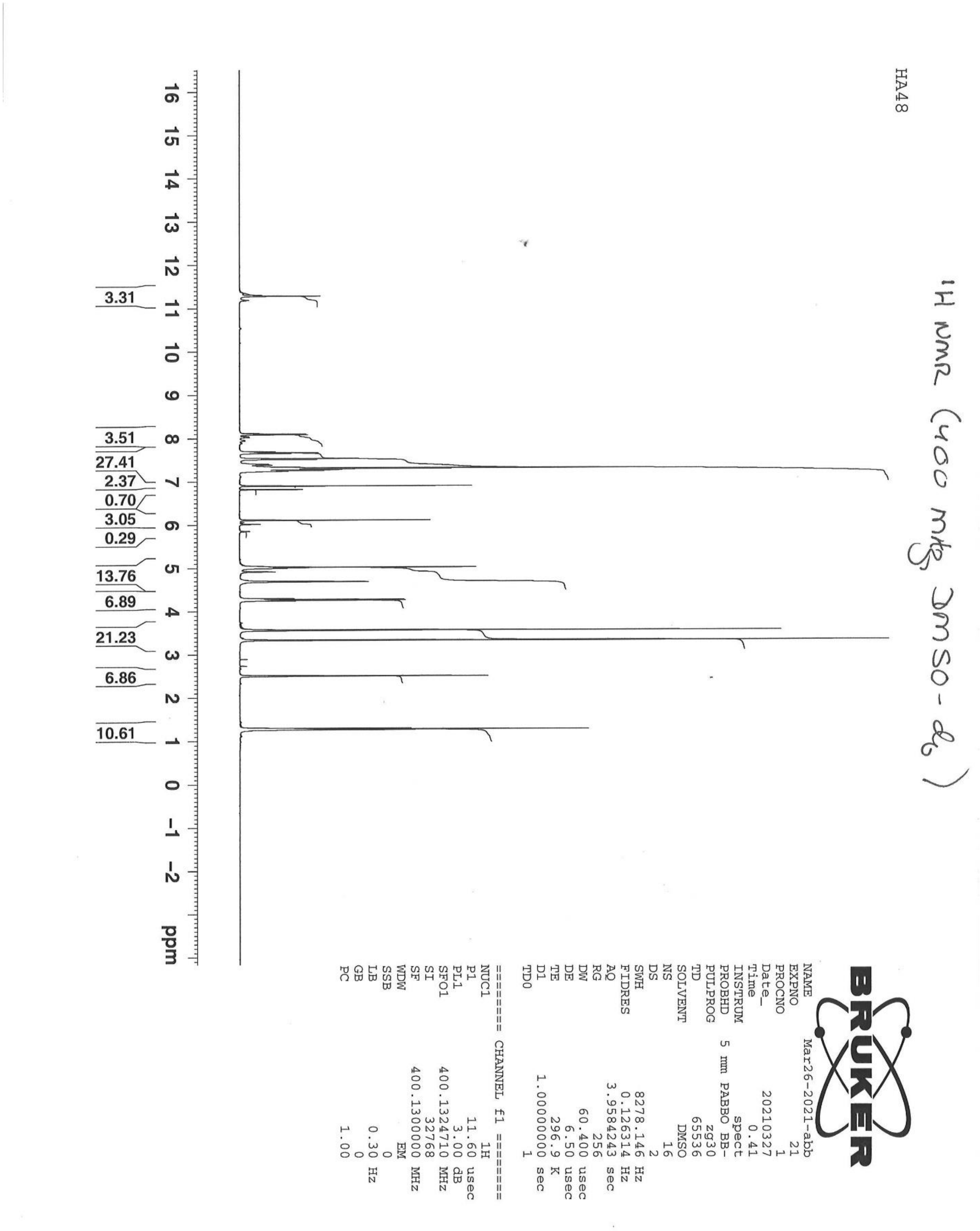

Figure S20. ^{1}H NMR spectrum of compound **7b** (400 MHz, DMSO-d_6).

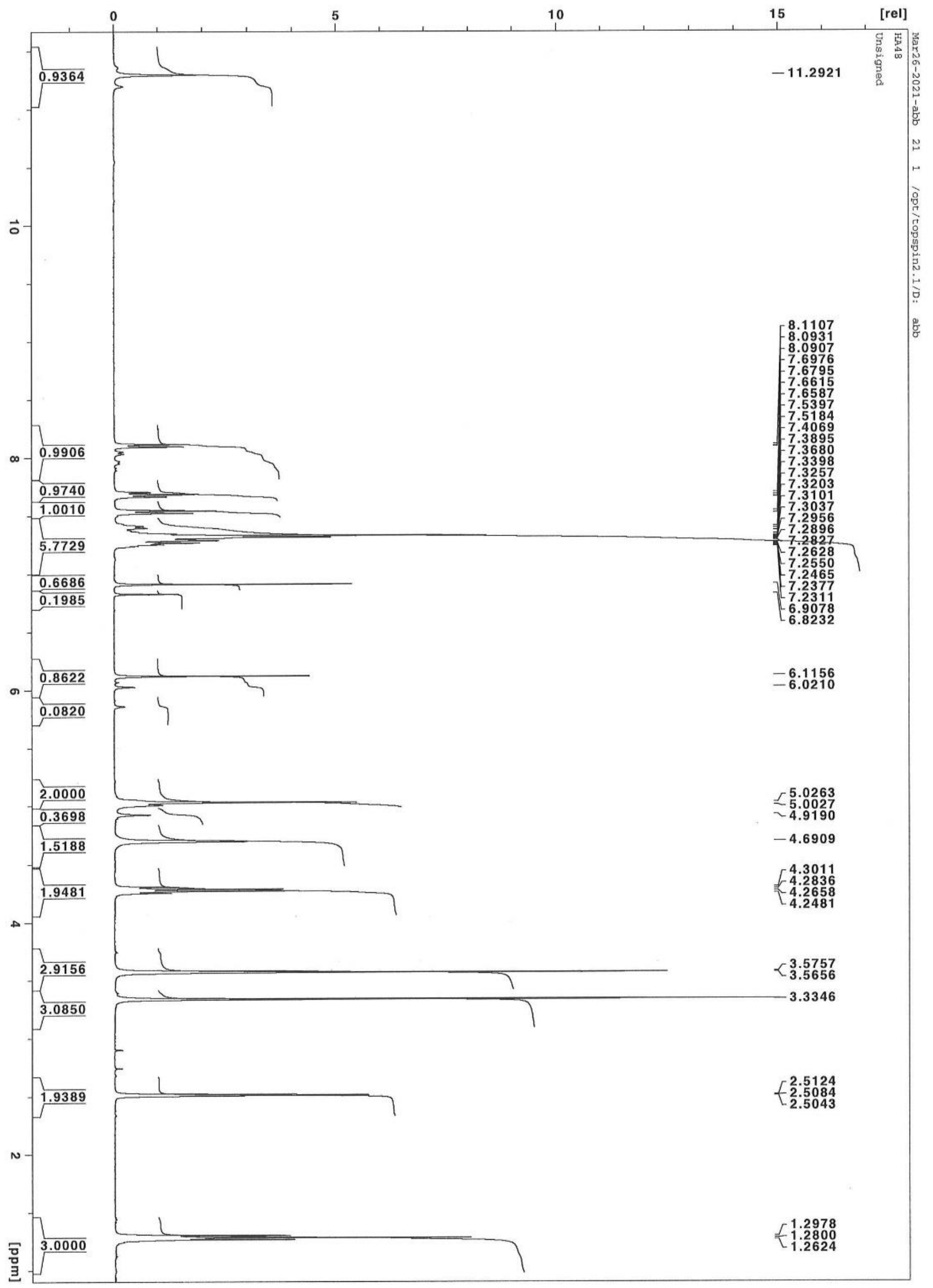

Figure S21. Detail of ^{1}H NMR spectrum of compound **7b.**

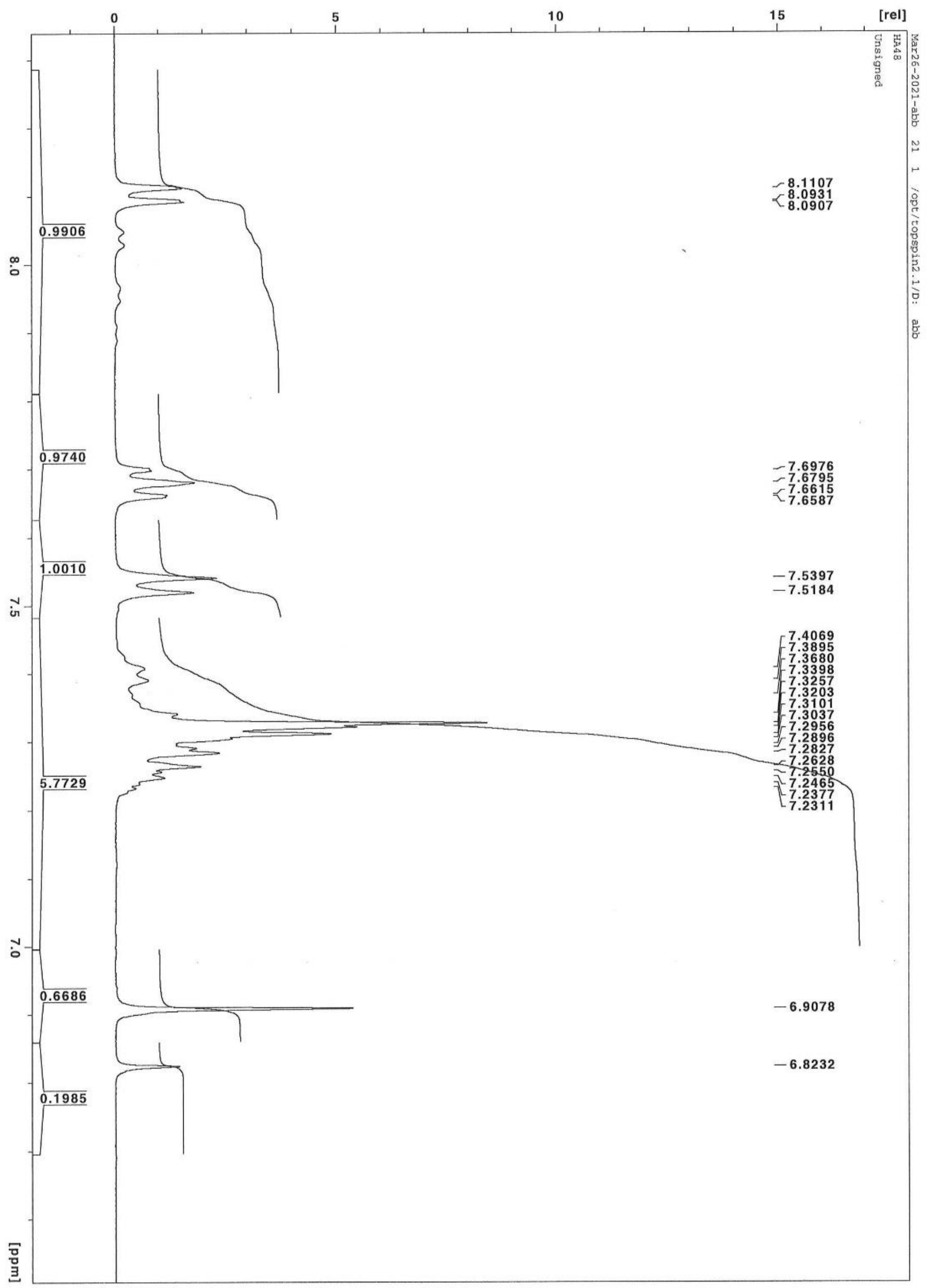

Figure S22. Detail of ^{1}H NMR spectrum of compound **7b**.

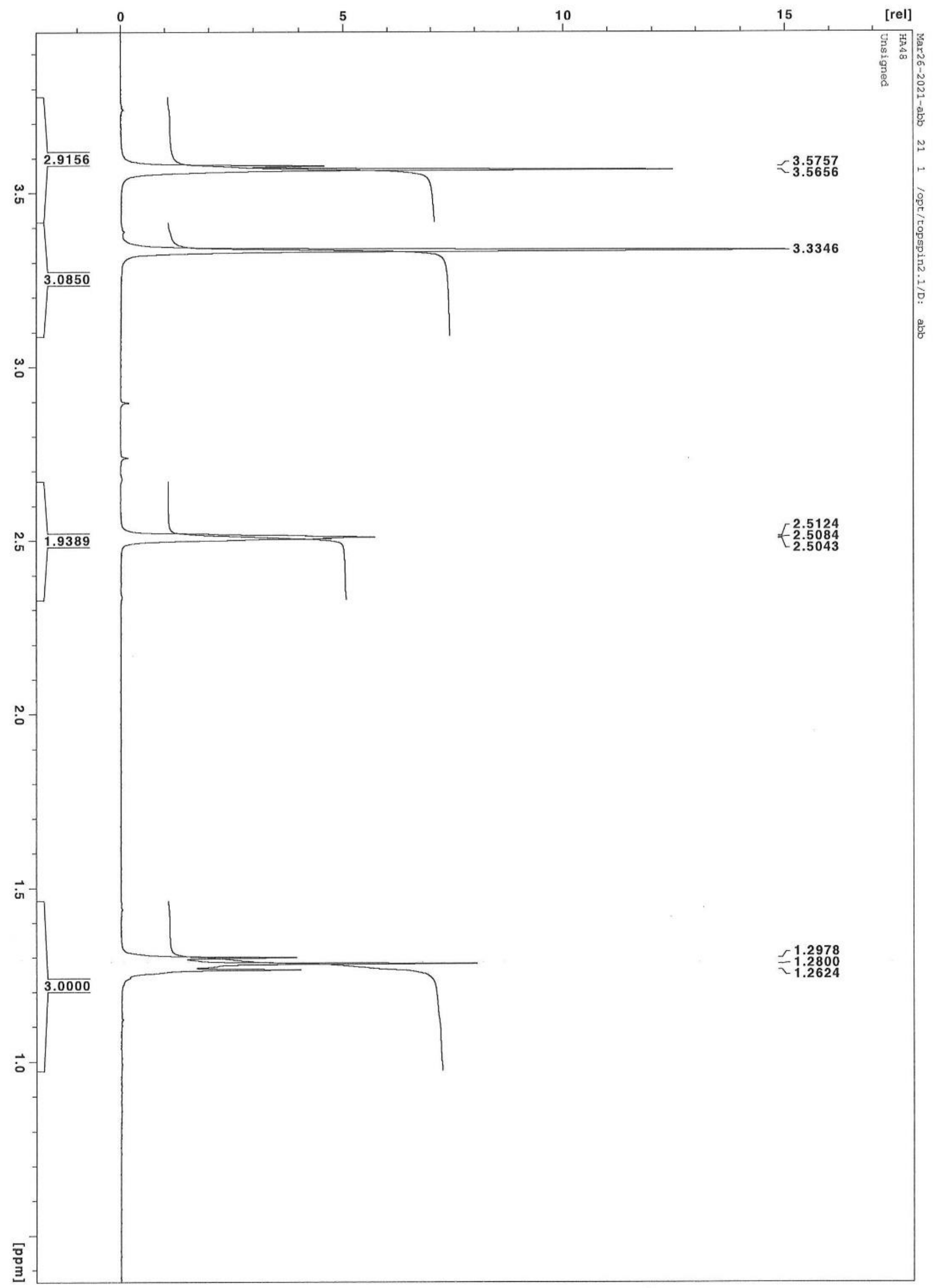

Figure S23. Detail of ^{1}H NMR spectrum of compound **7b.**

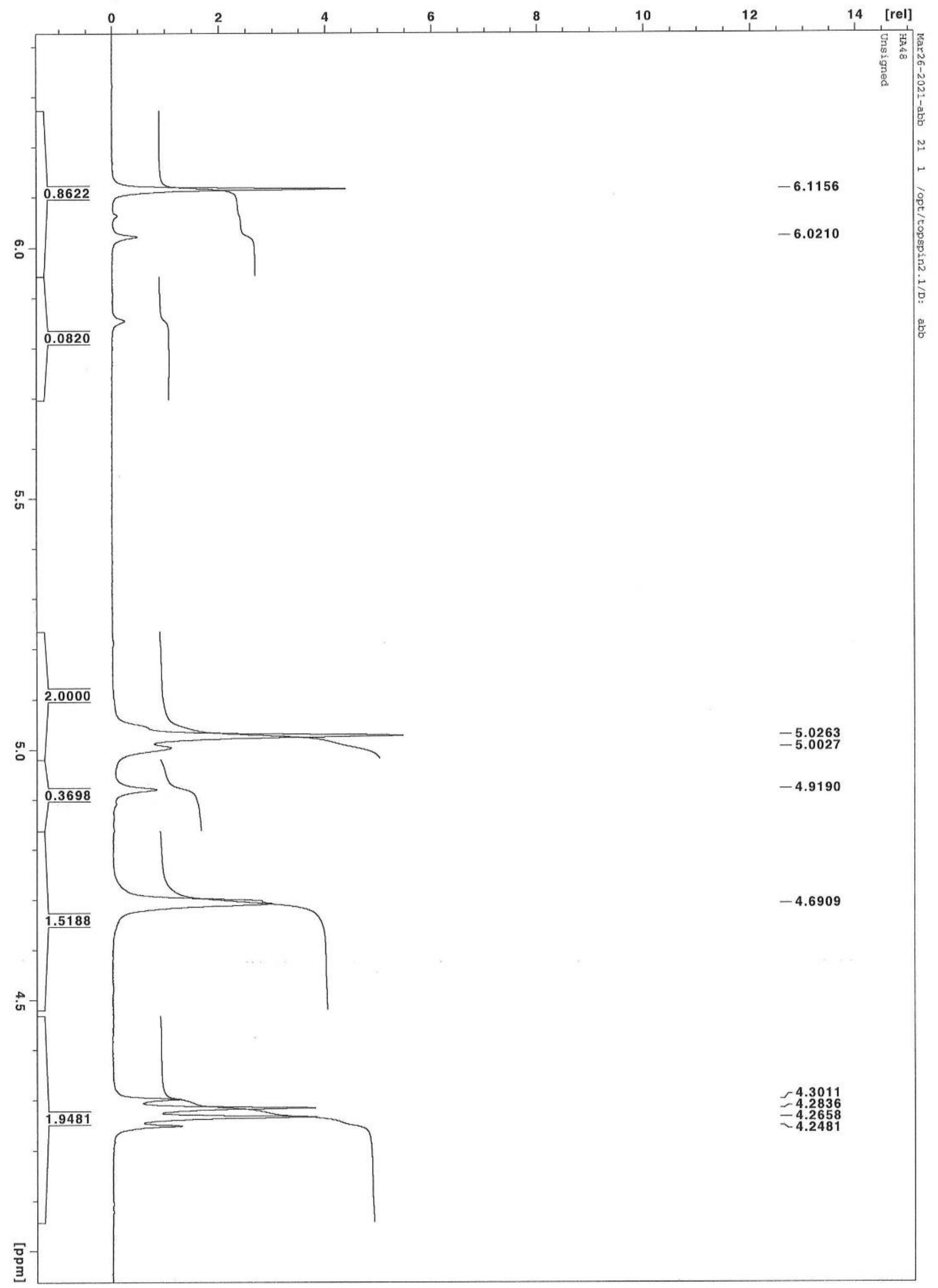

Figure S24. Detail of ^{1}H NMR spectrum of compound **7b**.

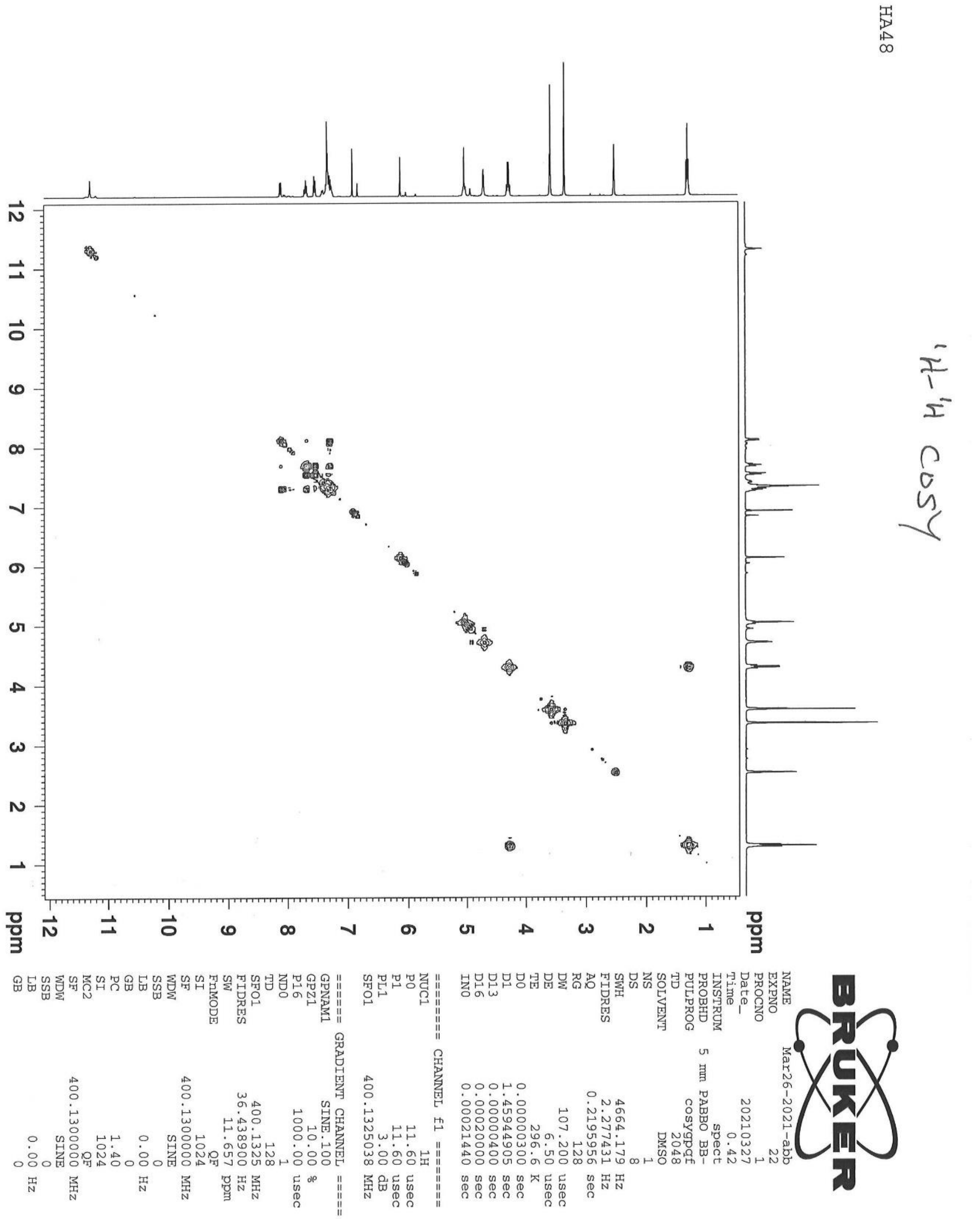

Figure S25. ^{1}H-^{1}H COSY spectrum of compound **7b.**

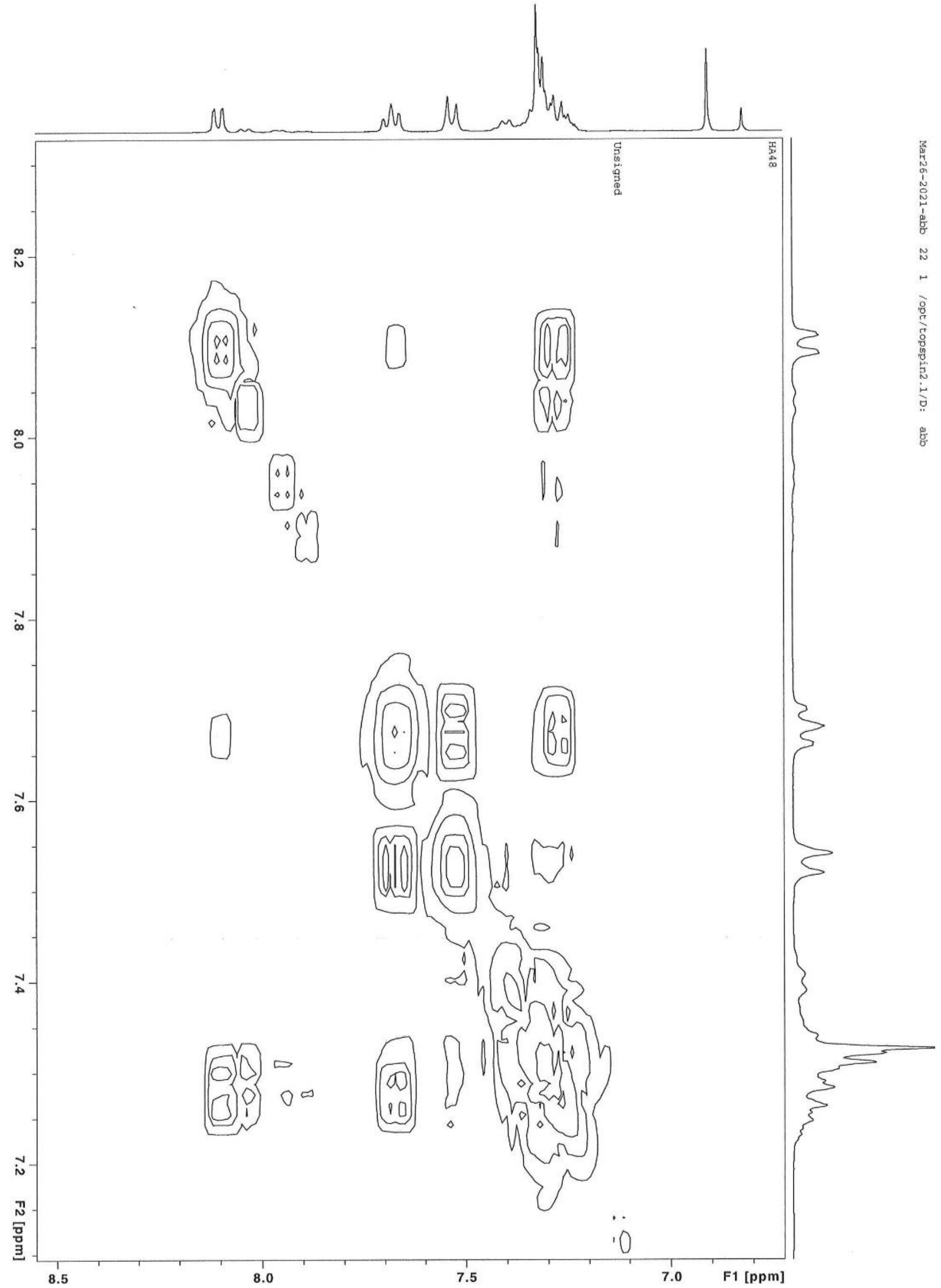

Figure S26. Detail of ^{1}H-^{1}H COSY spectrum of compound **7b.**

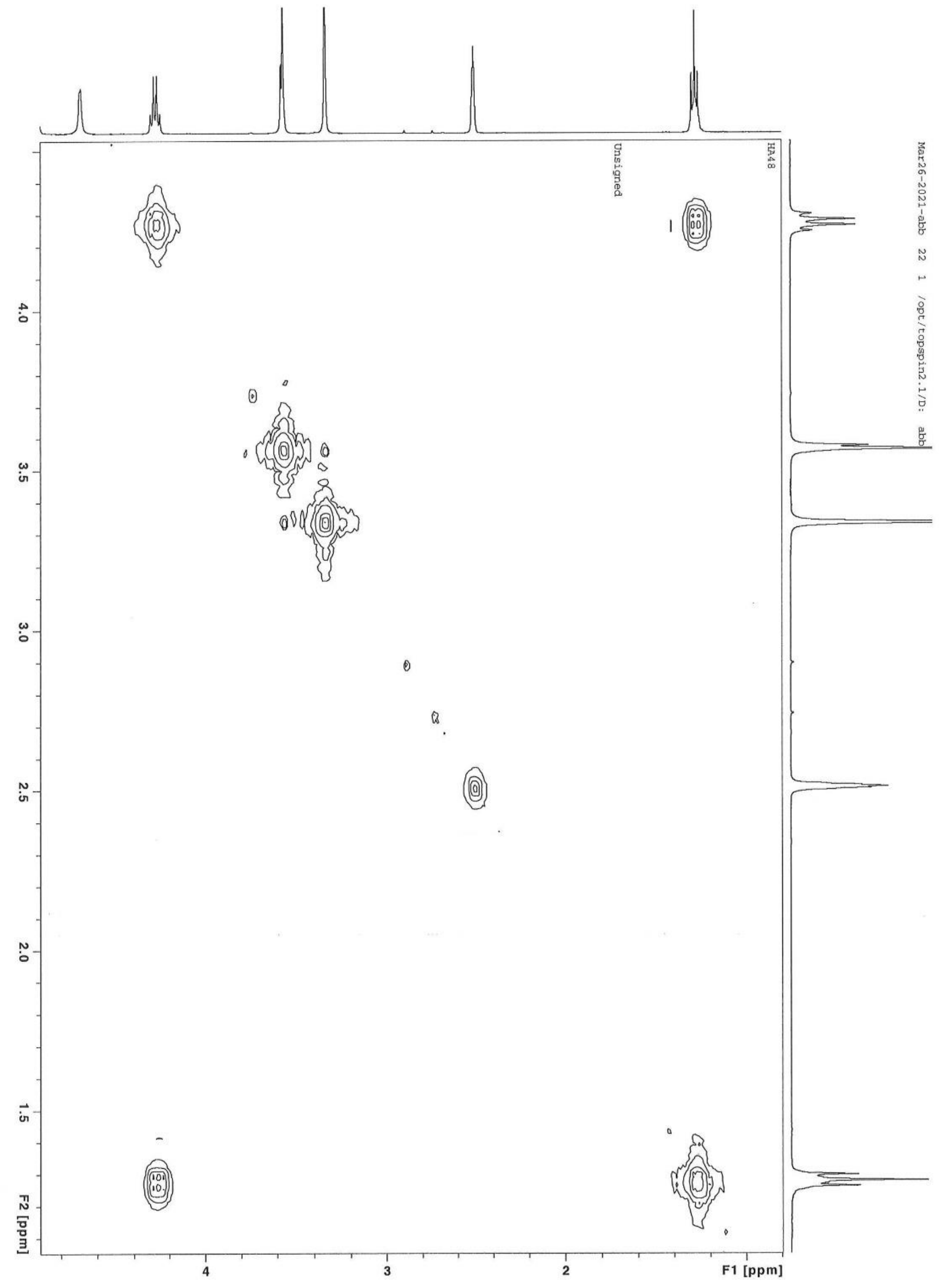

Figure S27. Detail of ^{1}H-^{1}H COSY spectrum of compound **7b**.

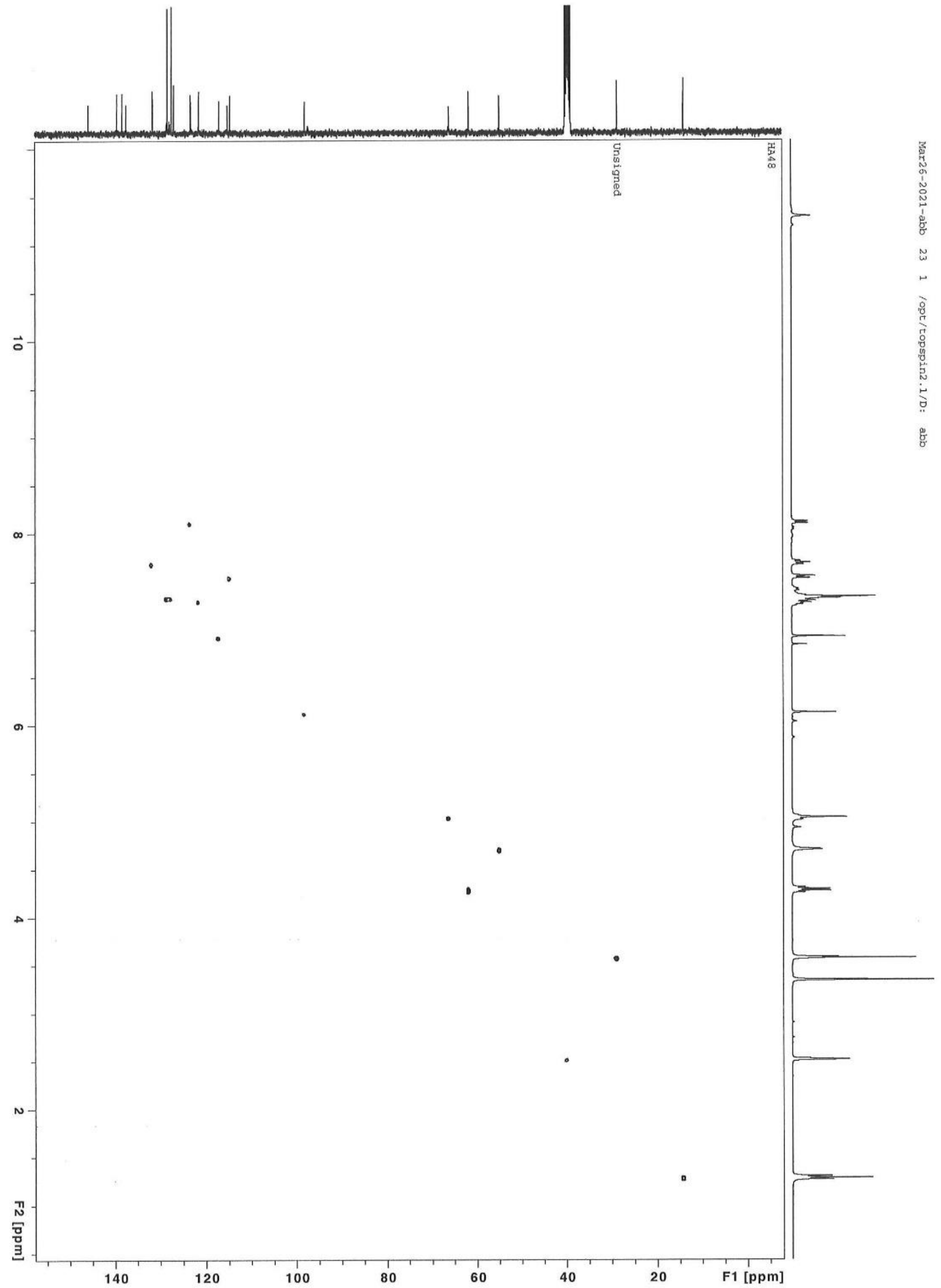

Figure S28. ^{1}H-^{13}C HSQC spectrum of compound **7b.**

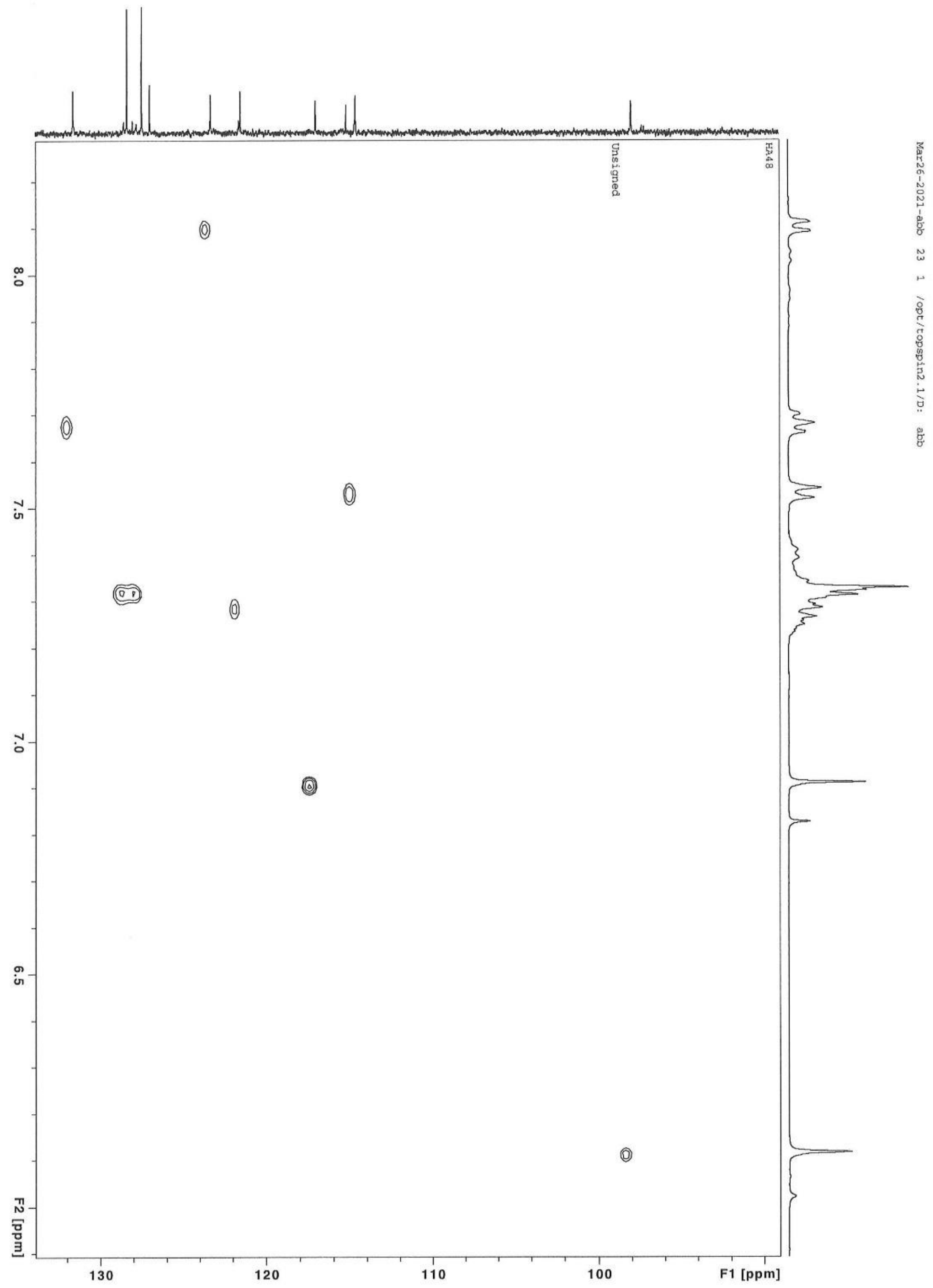

Figure S29. Detail of ^{1}H-^{13}C HSQC spectrum of compound **7b.**

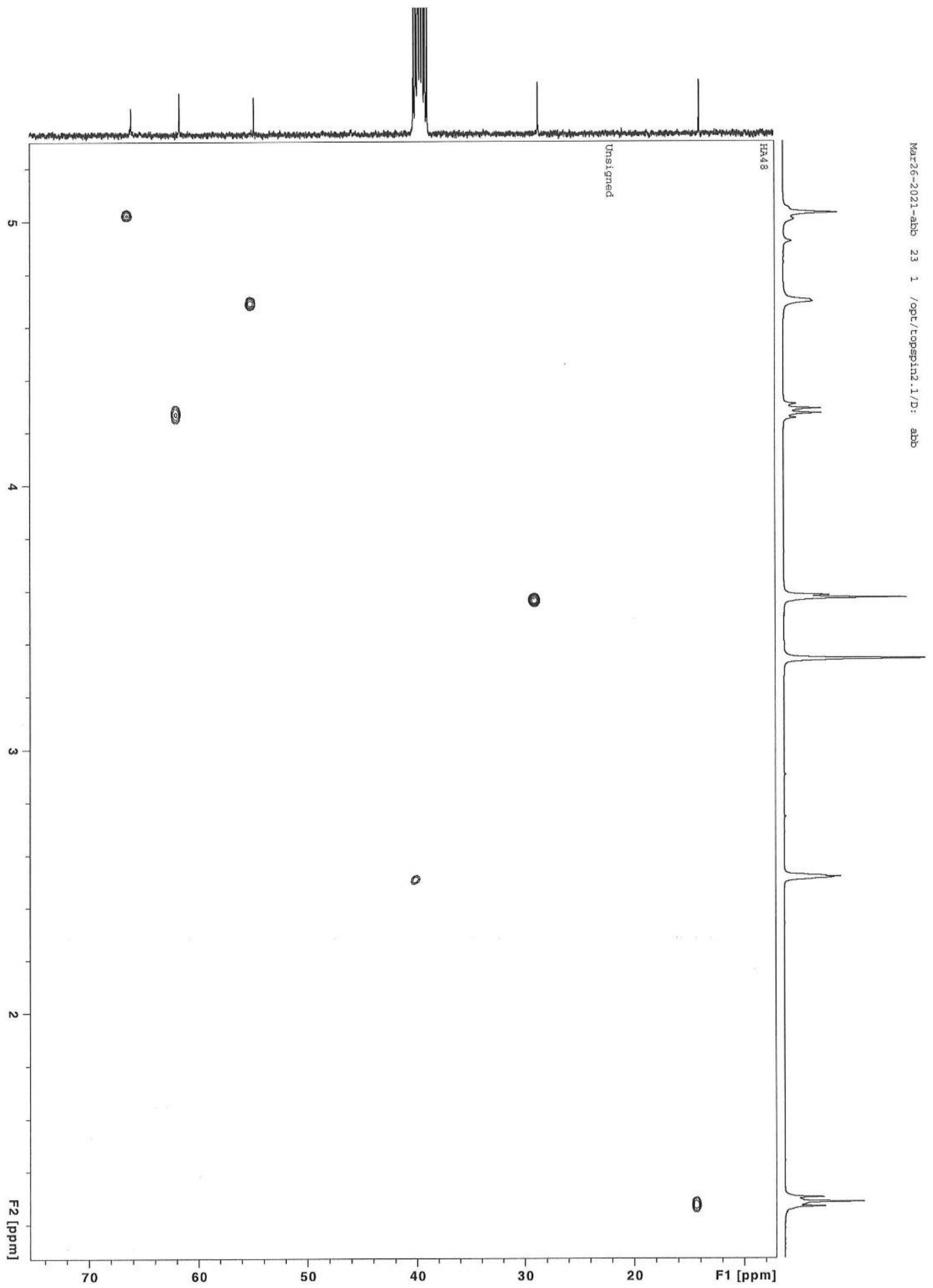

Figure S30. Detail of ^{1}H-^{13}C HSQC spectrum of compound **7b.**

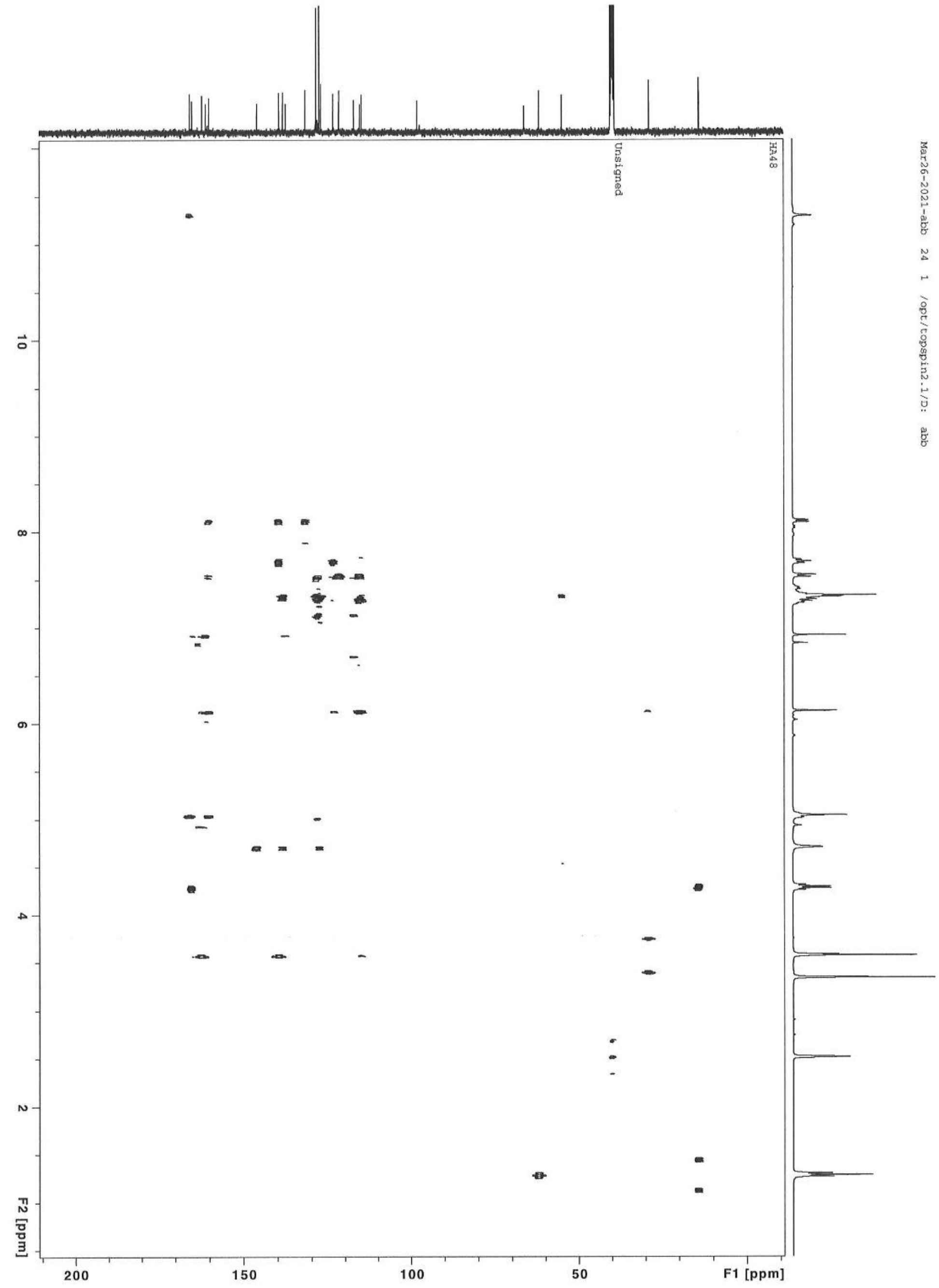

Figure S31. ^{1}H-^{13}C HMBC spectrum of compound **7b.**

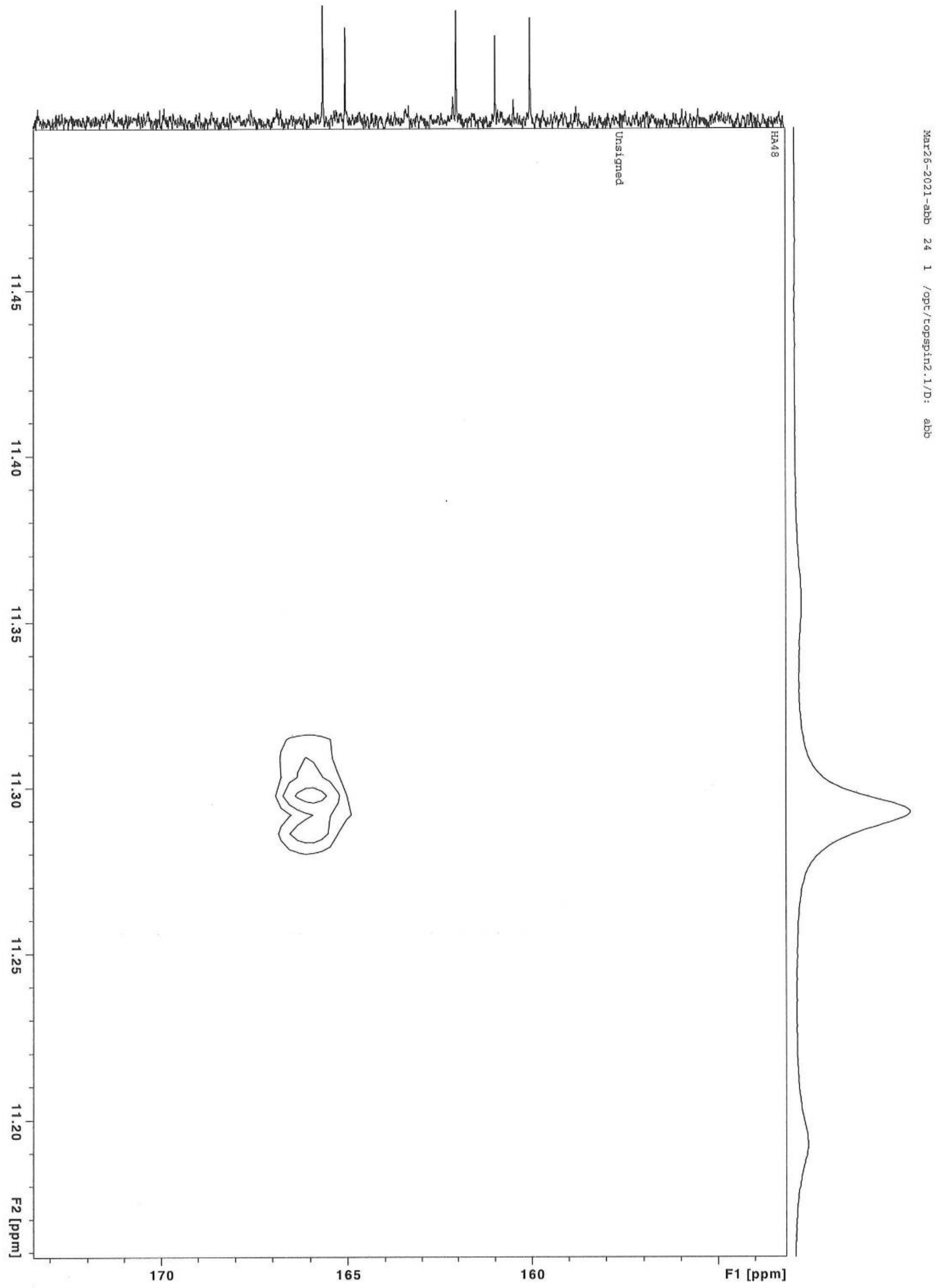

Figure S32. Detail of ^{1}H-^{13}C HMBC spectrum of compound **7b.**

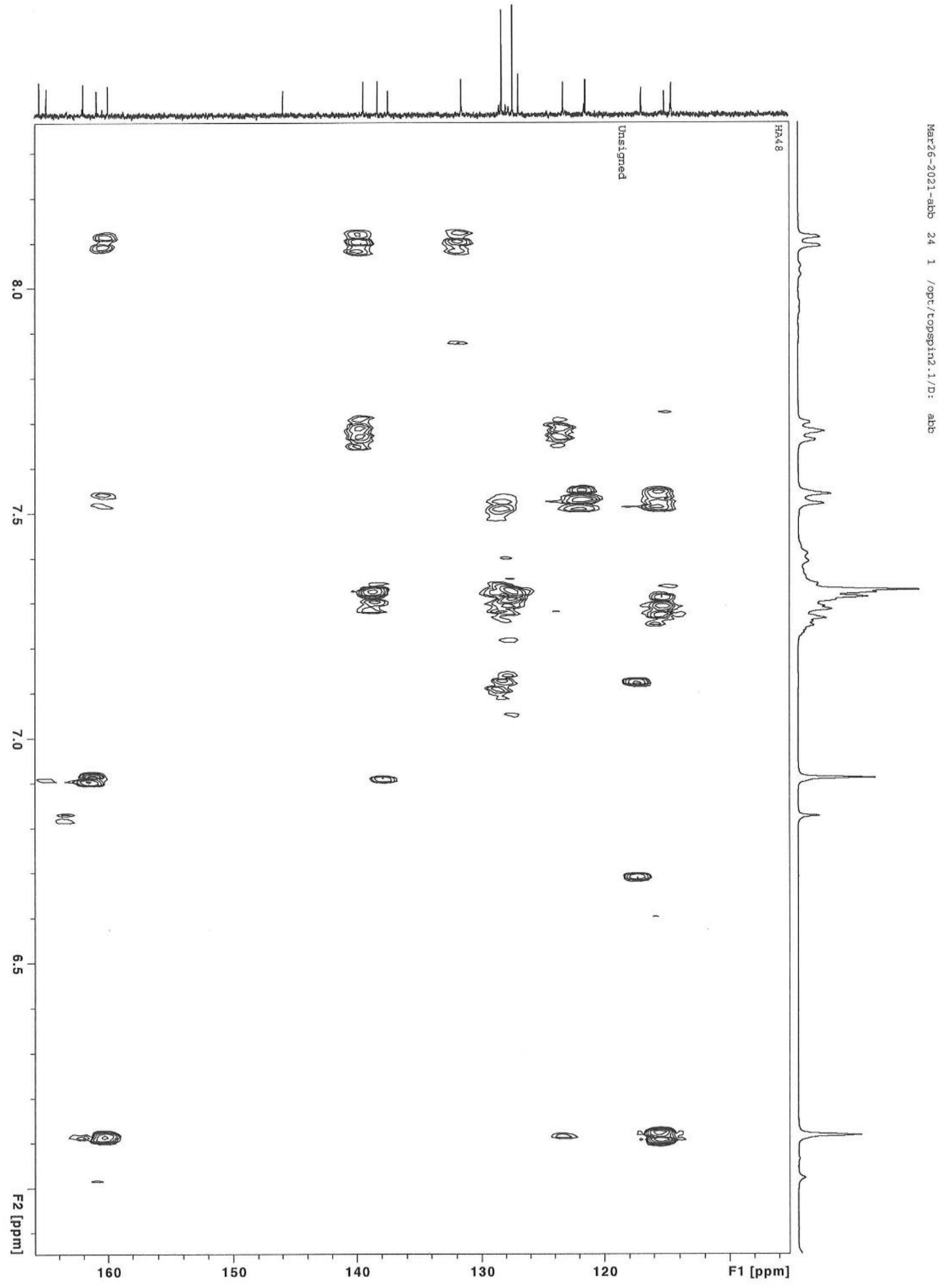

Figure S33. Detail of ^{1}H-^{13}C HMBC spectrum of compound **7b.**

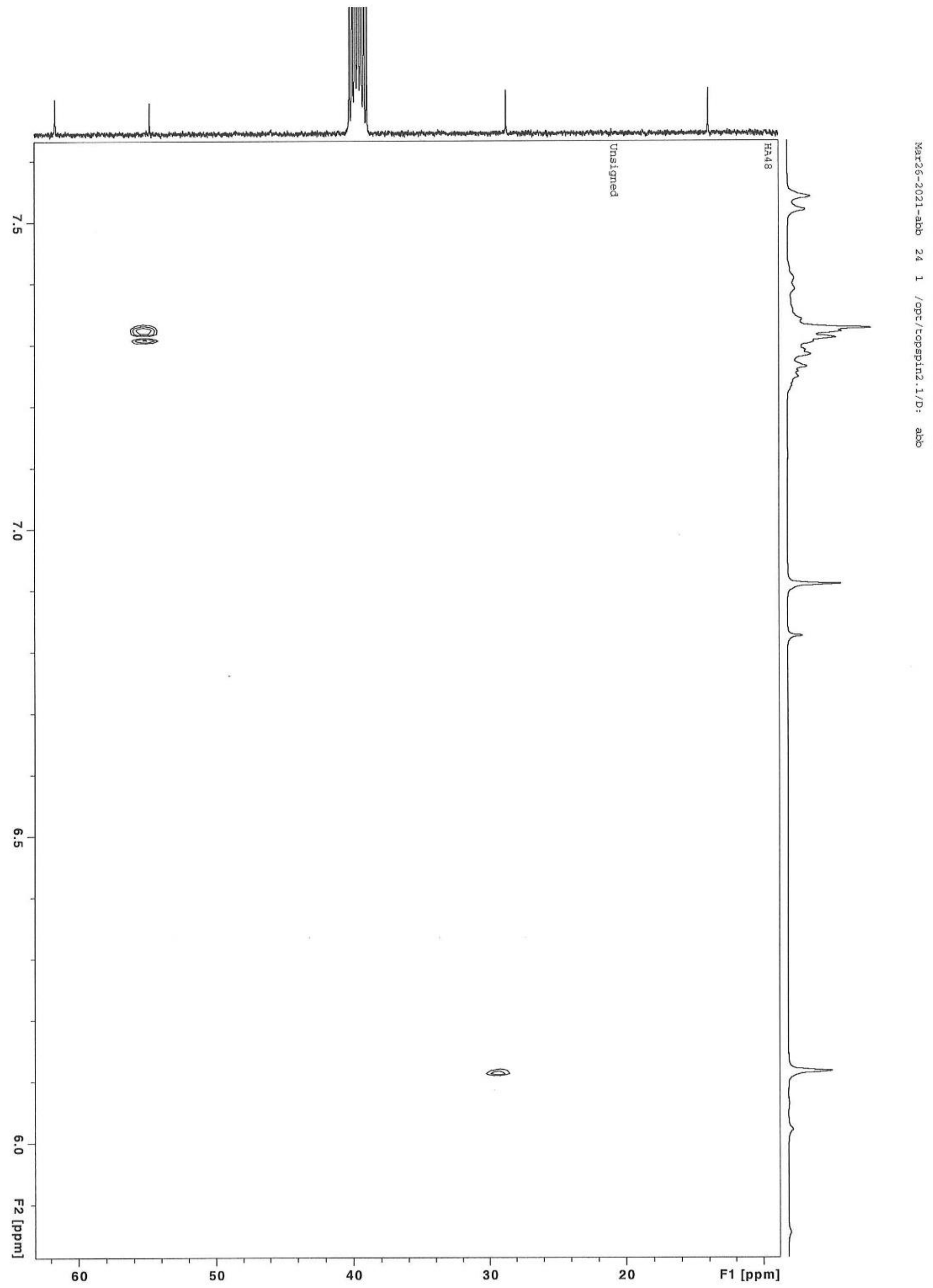

Figure S34. Detail of ^{1}H-^{13}C HMBC spectrum of compound **7b.**

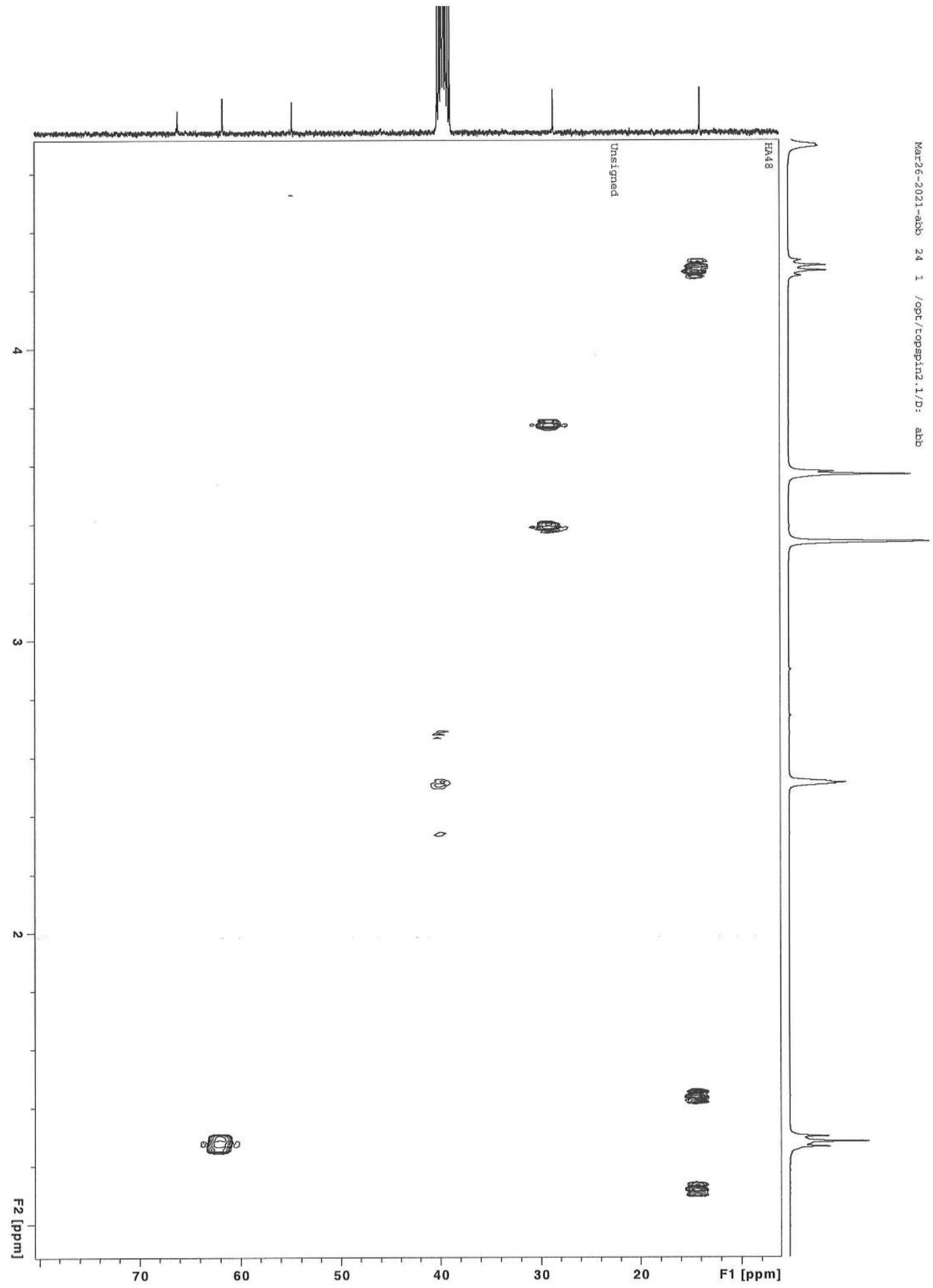

Figure S35. Detail of ^{1}H-^{13}C HMBC spectrum of compound **7b.**

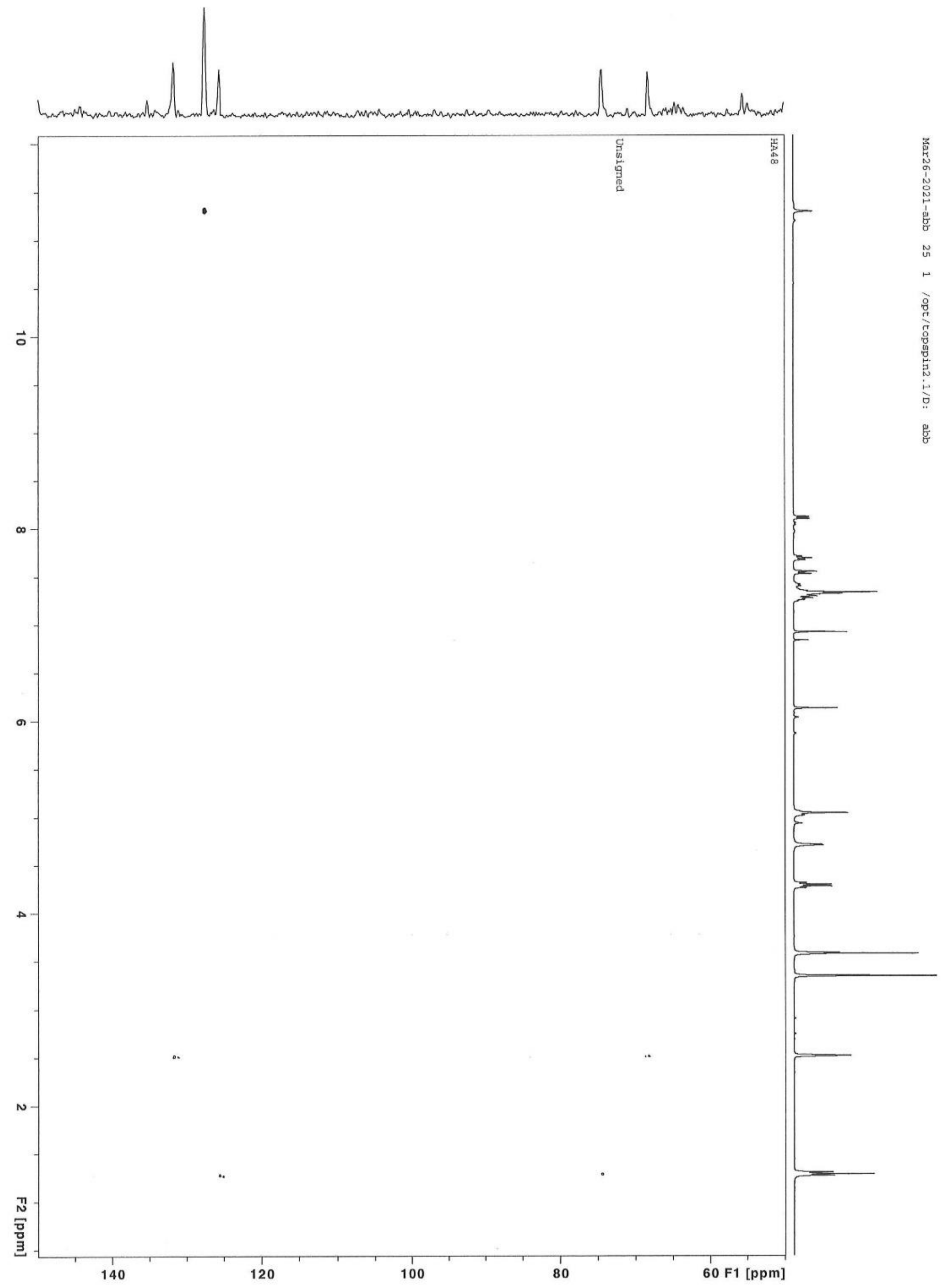

Figure S36. ^{1}H-^{15}N HSQC spectrum of compound **7b.**

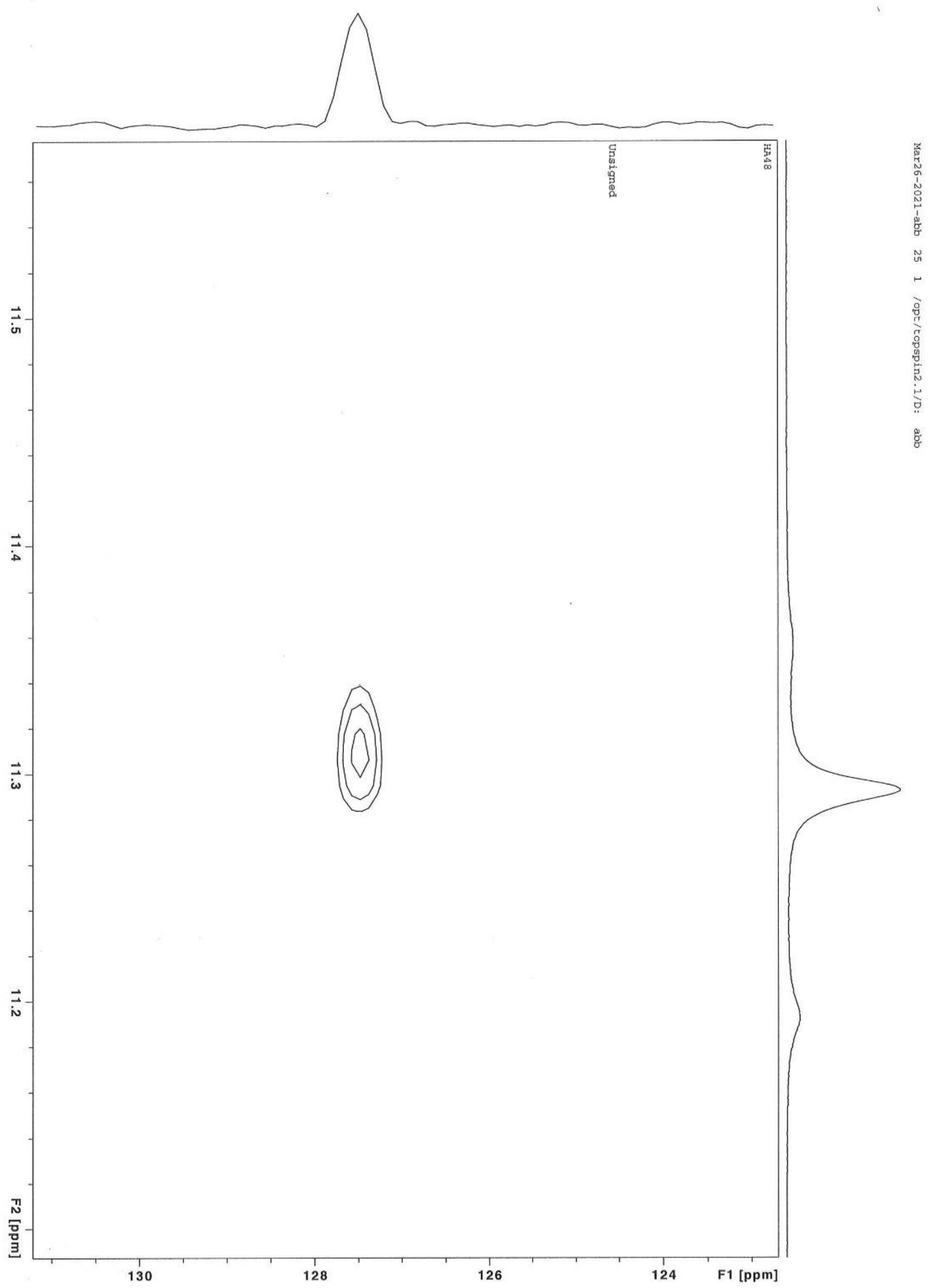

Figure S37. Detail of ^{1}H-^{15}N HSQC spectrum of compound **7b.**

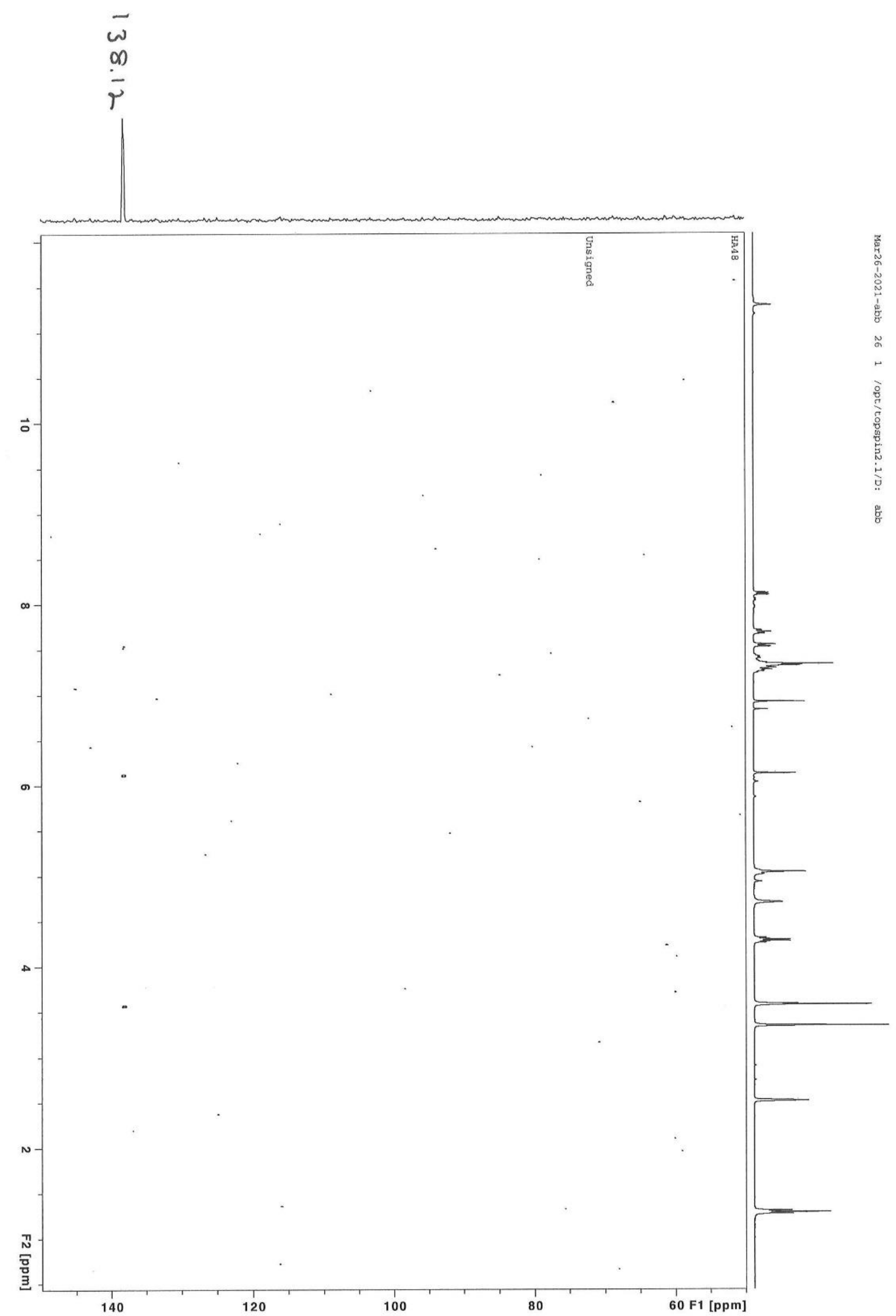

Figure S38. ^{1}H-^{15}N HMBC spectrum of compound **7b.**

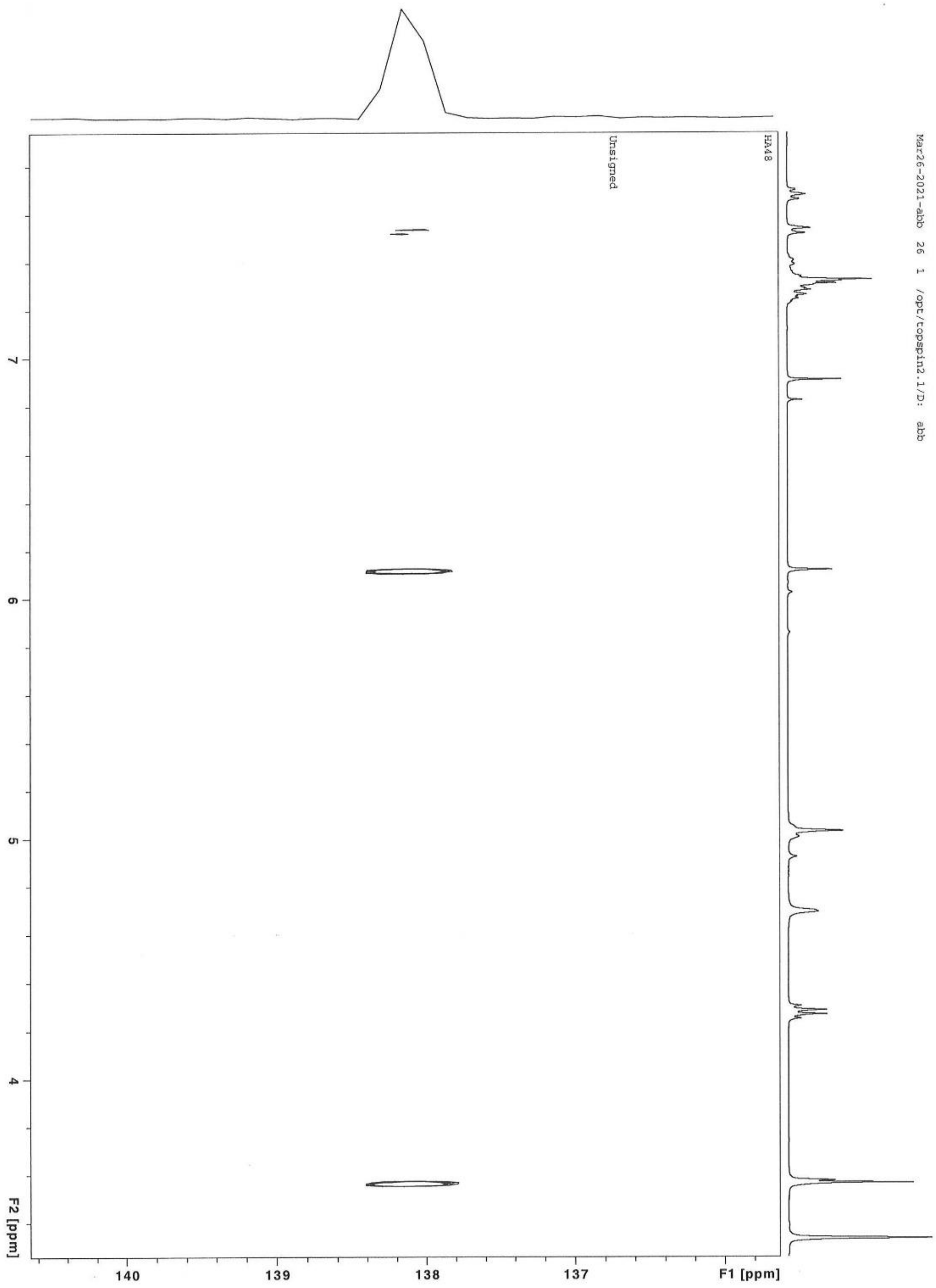

Figure S39. Detail of ^{1}H-^{15}N HMBC spectrum of compound **7b.**

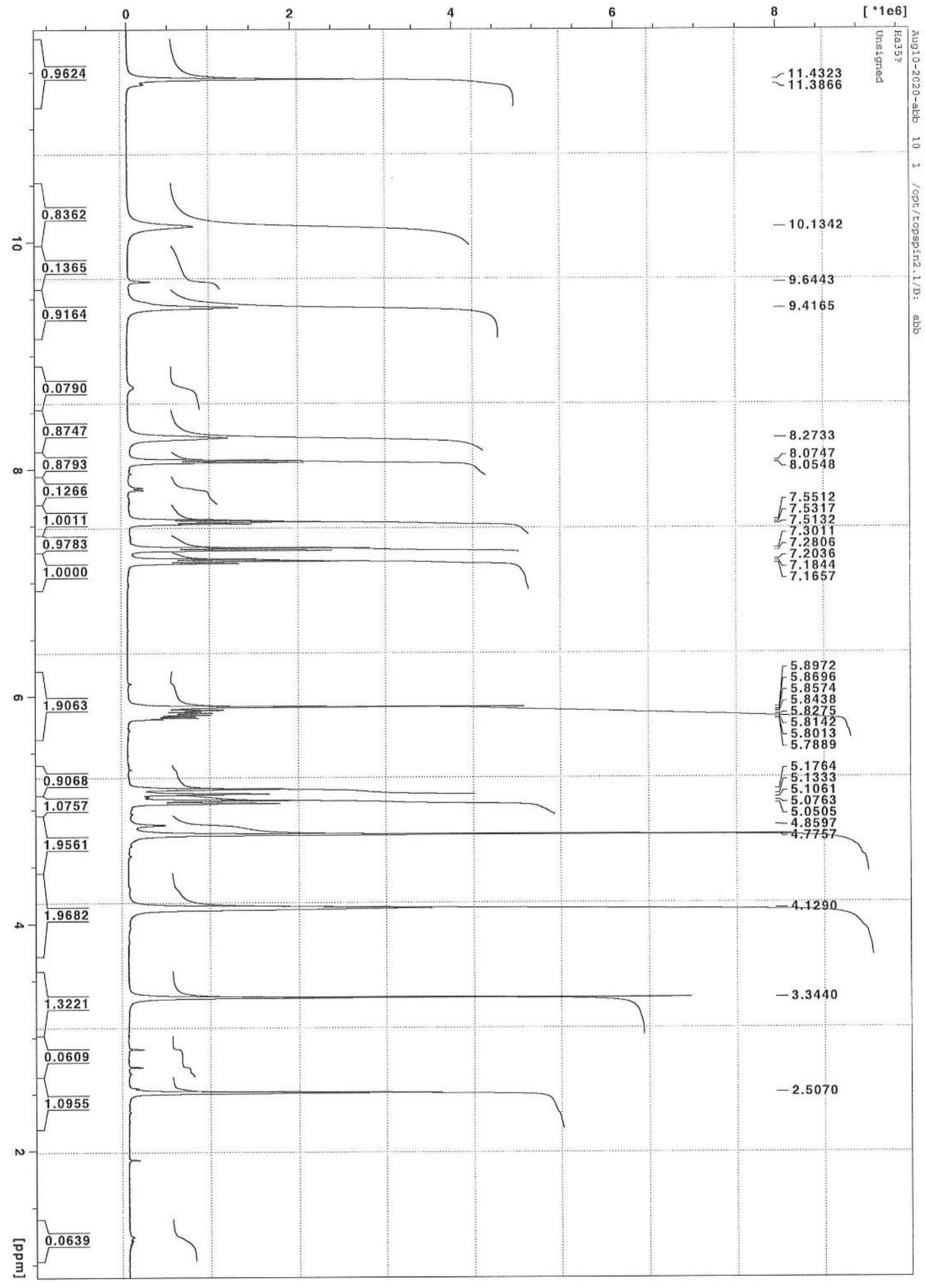

Figure S40. ^{1}H spectrum of compound **7a** (400 MHz, DMSO-d_6).

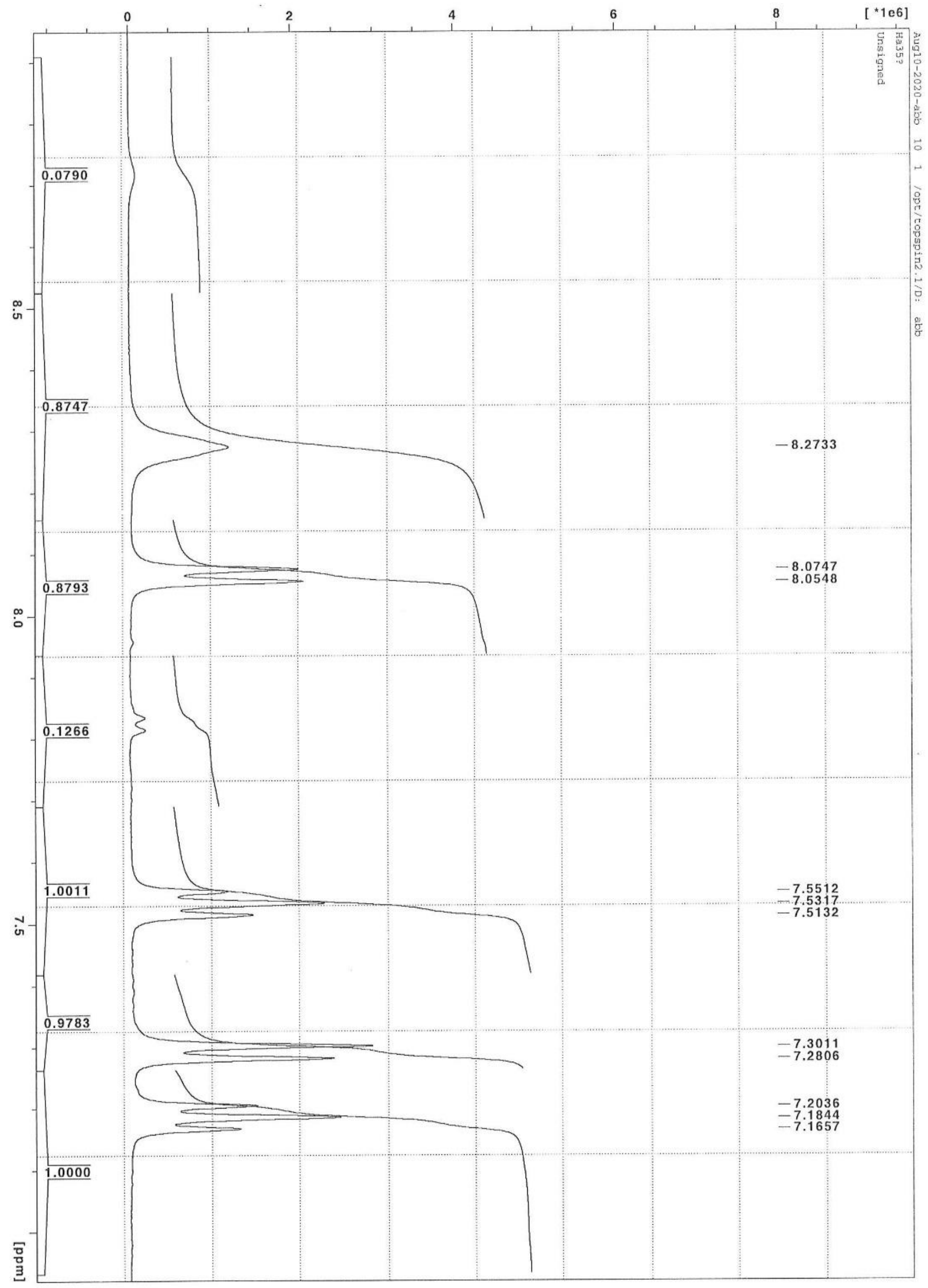

Figure S41. Detail of ^{1}H spectrum of compound **7a.**

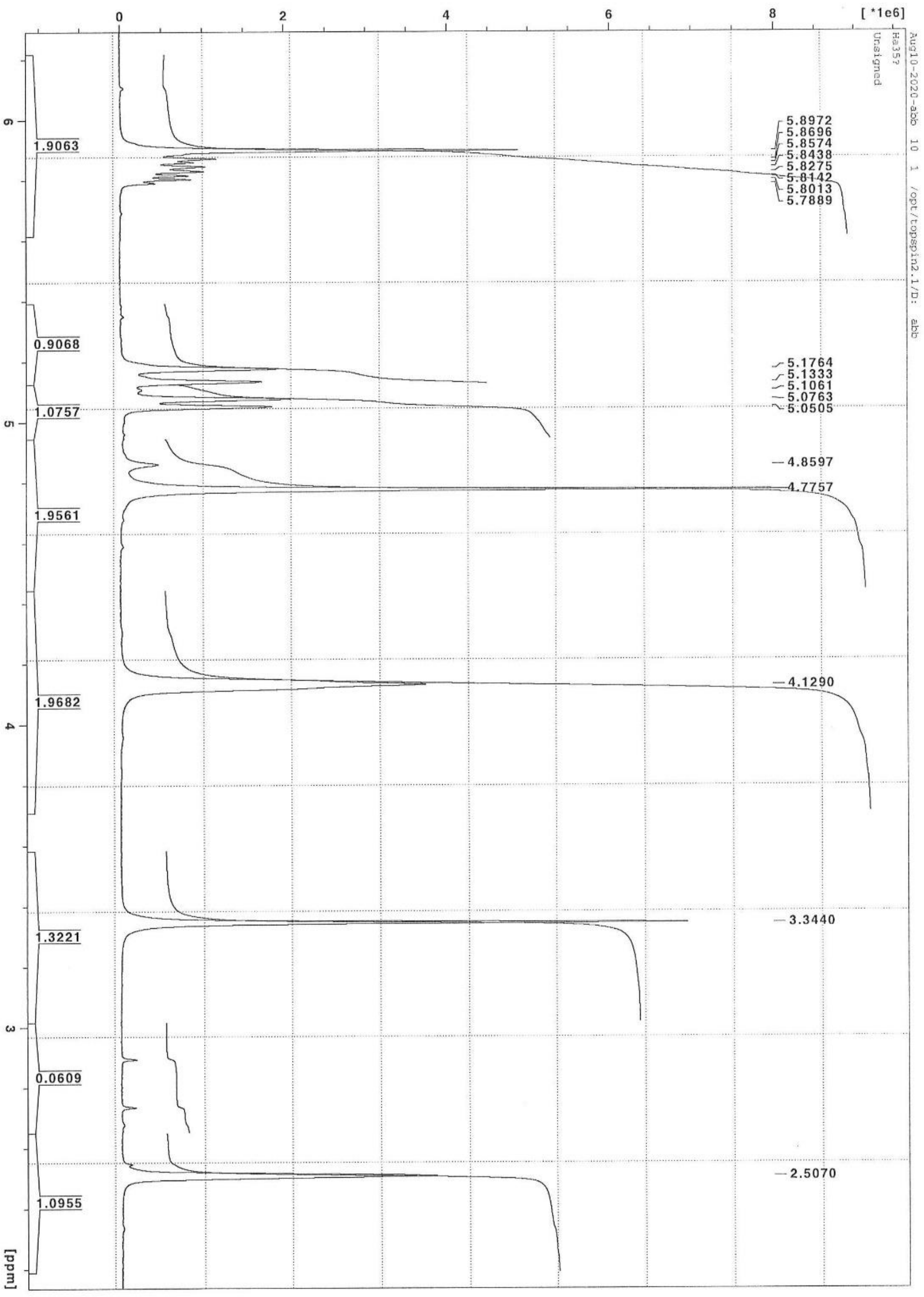

Figure S42. Detail of ^{1}H spectrum of compound **7a.**

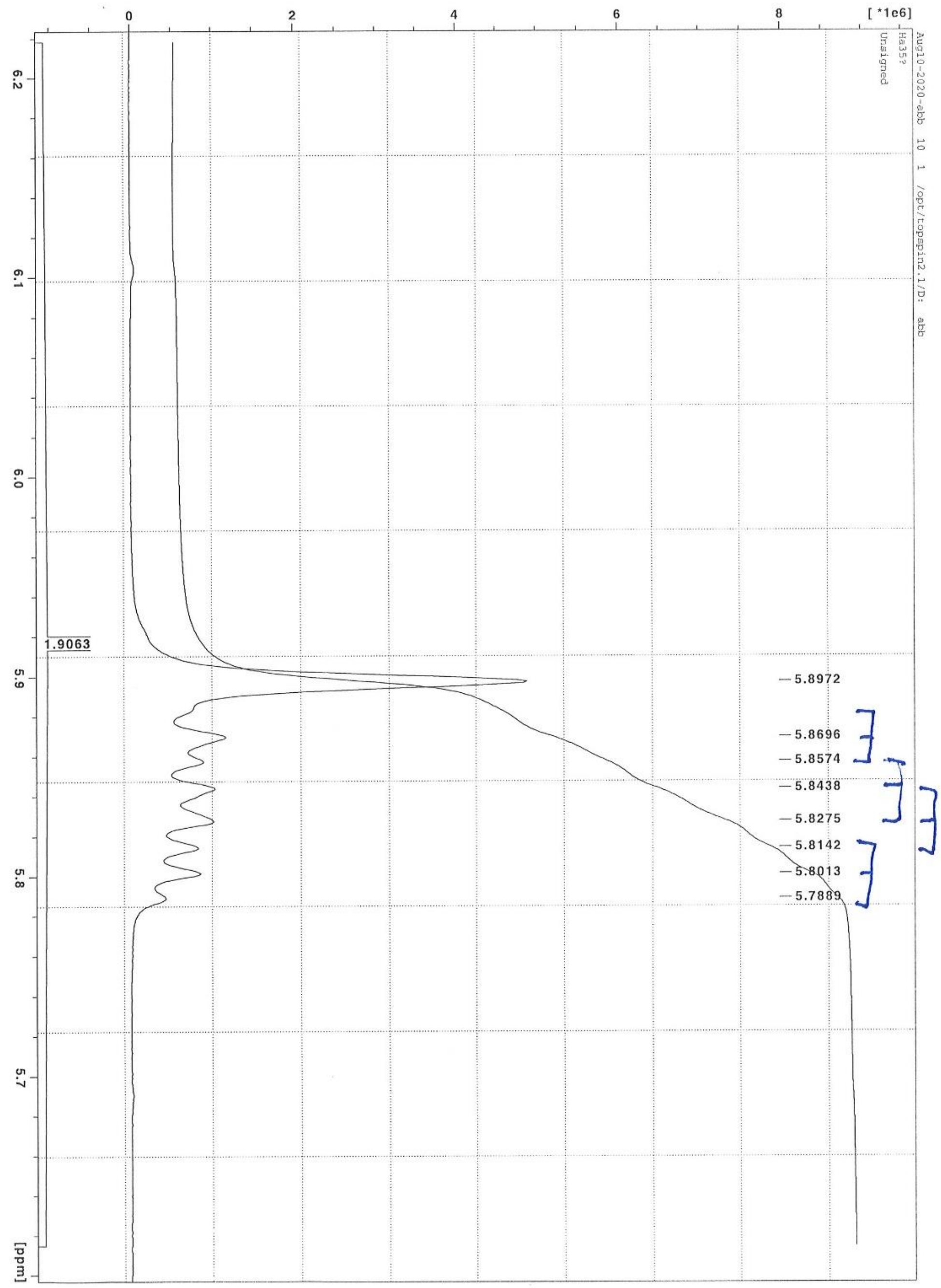

Figure S43. Detail of ^{1}H spectrum of compound **7a.**

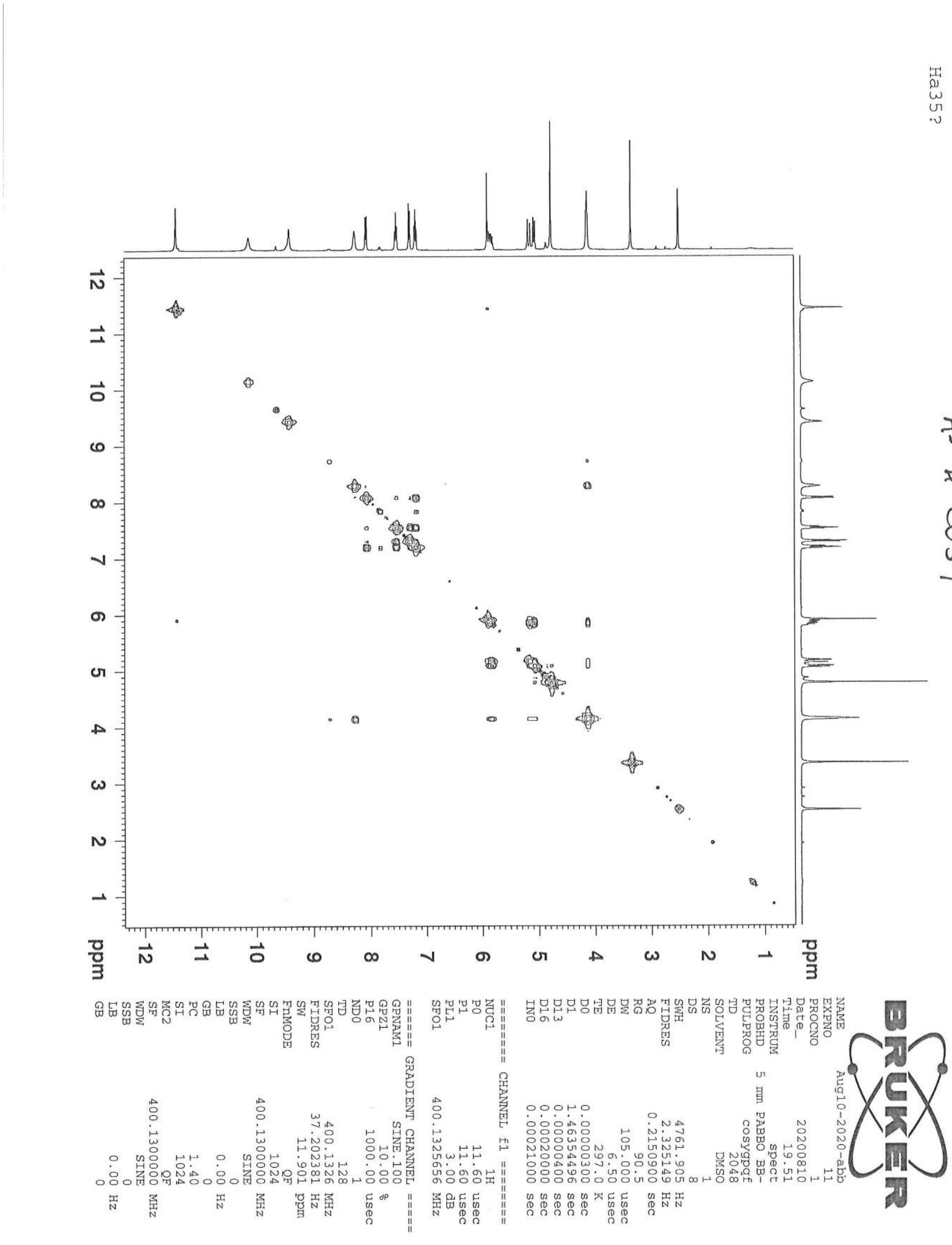

Figure S44. ^{1}H-^{1}H COSY spectrum of compound **7a.**

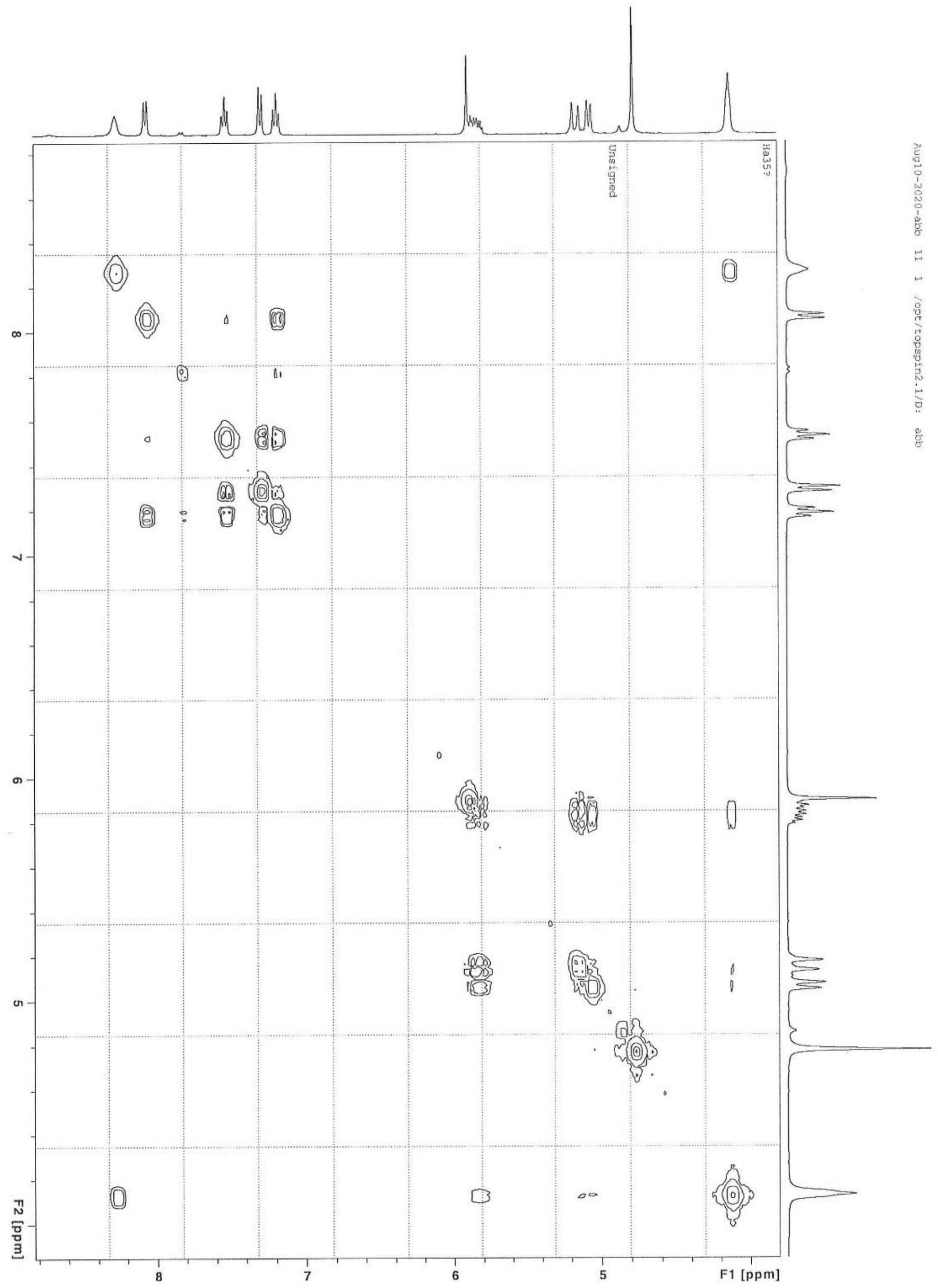

Figure S45. Detail of ^{1}H-^{1}H COSY spectrum of compound **7a.**

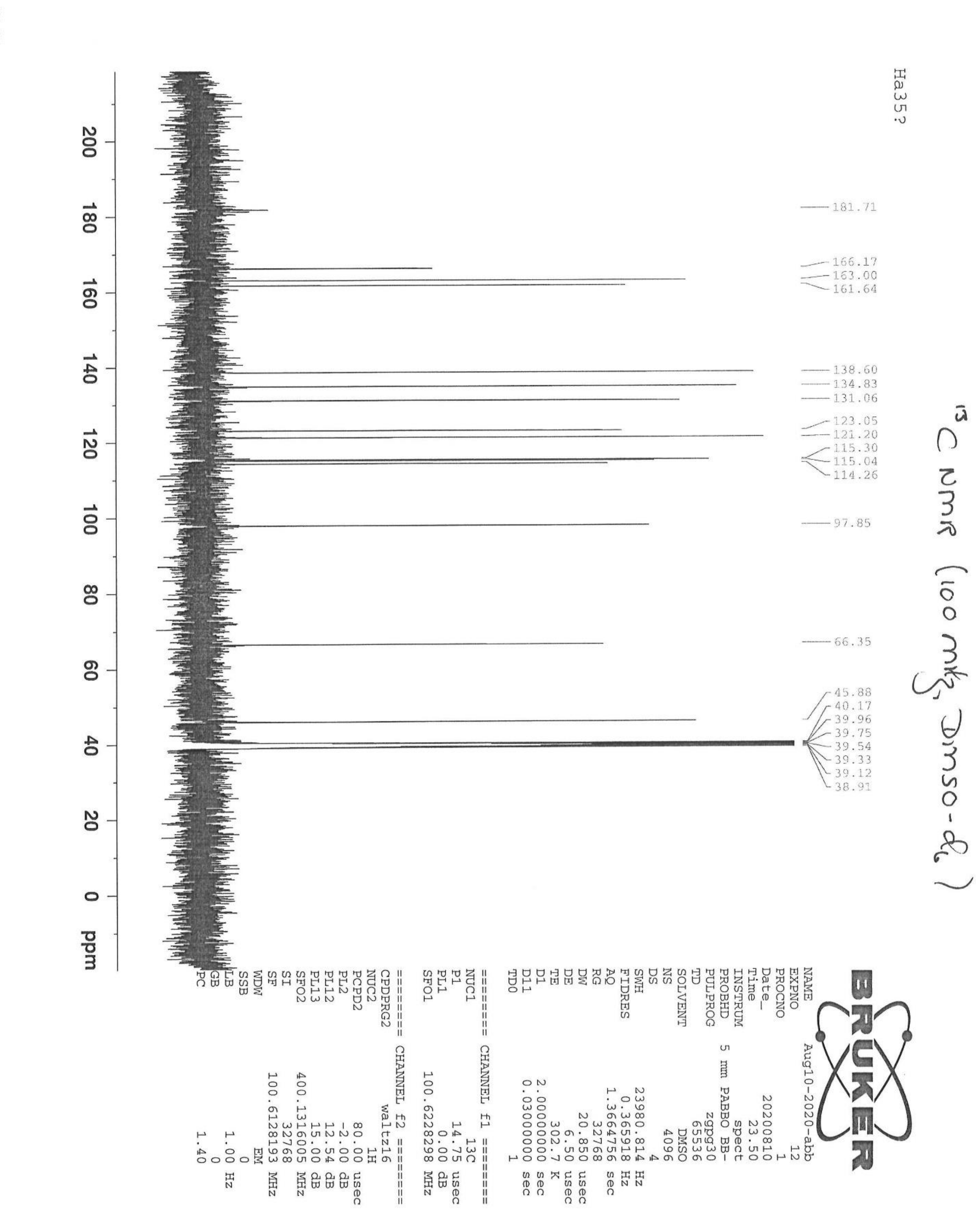

Figure S46. ^{13}C spectrum of compound **7a** (100 MHz, DMSO-d_6).

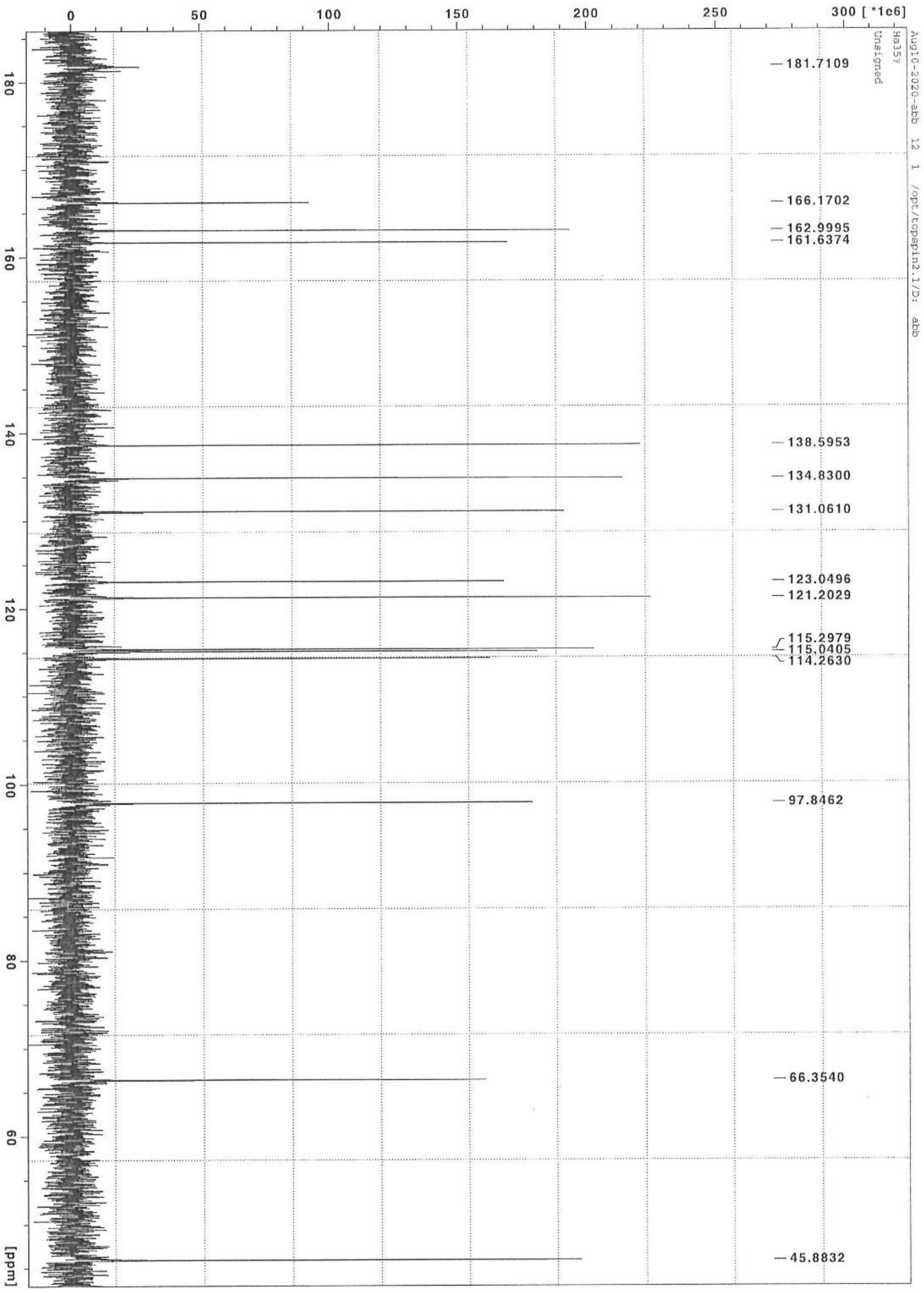

Figure S47. Detail of ^{13}C spectrum of compound **7a.**

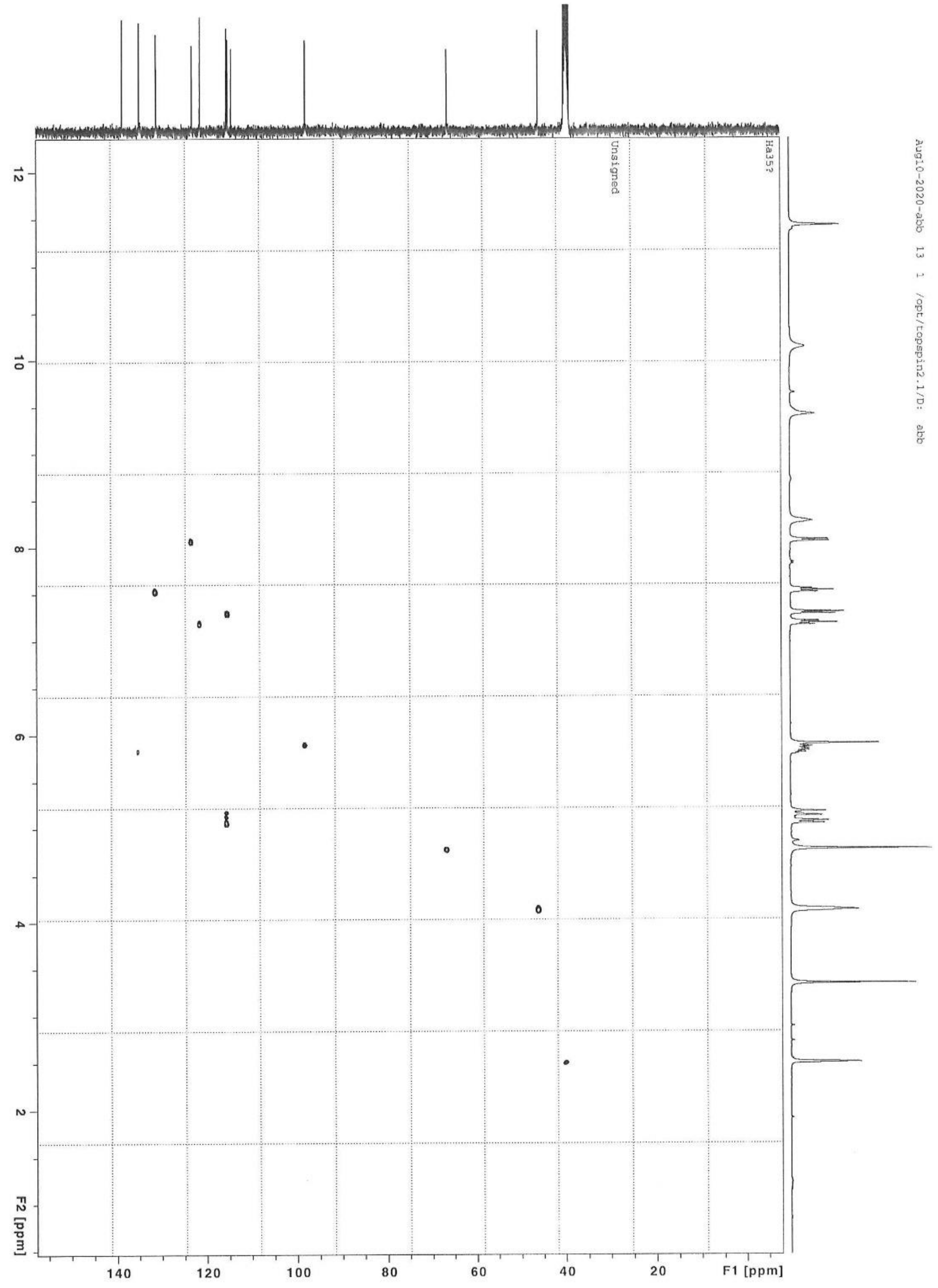

Figure S48. ^{1}H-^{13}C HSQC spectrum of compound **7a.**

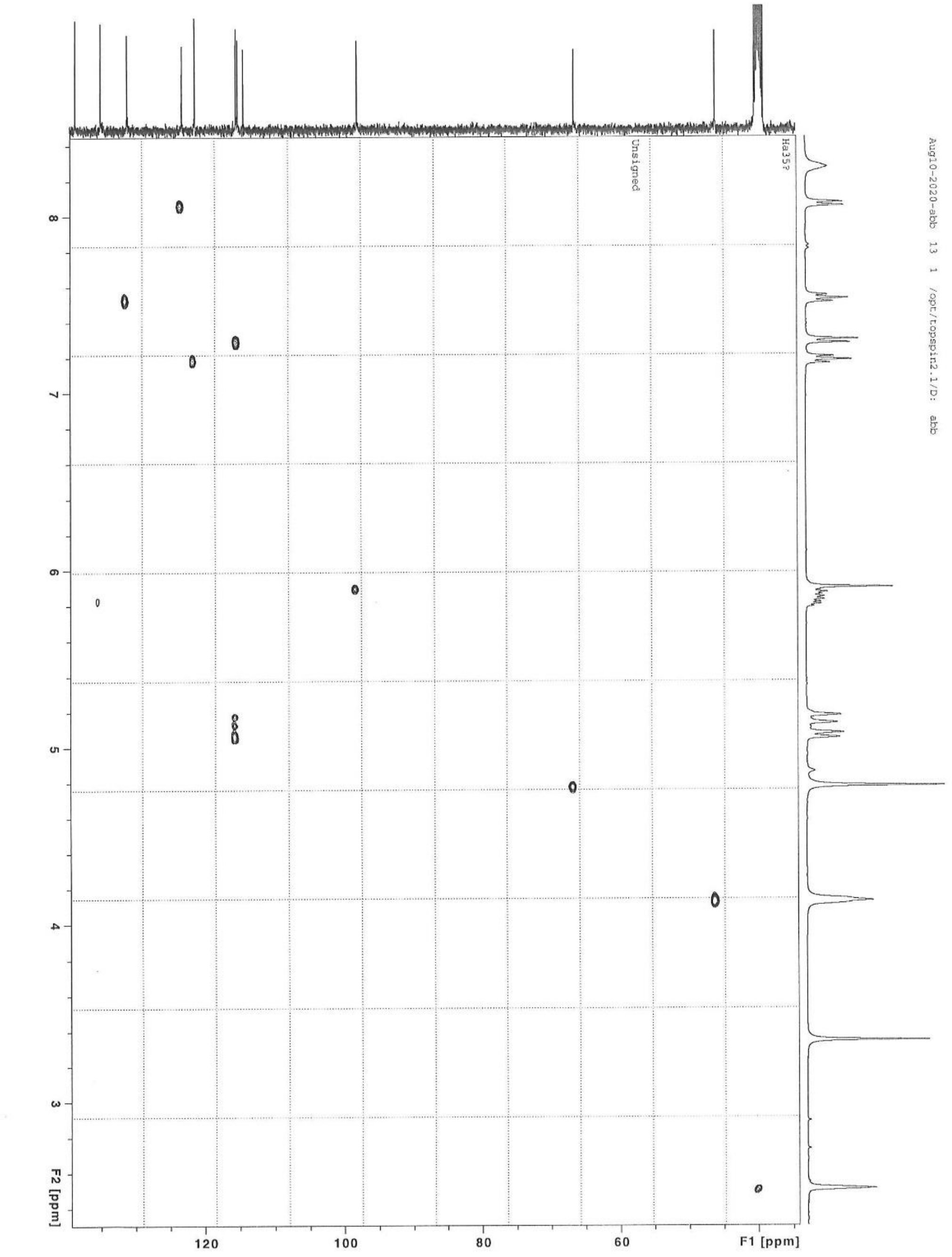

Figure S49. Detail of ^{1}H-^{13}C HSQC spectrum of compound **7a.**

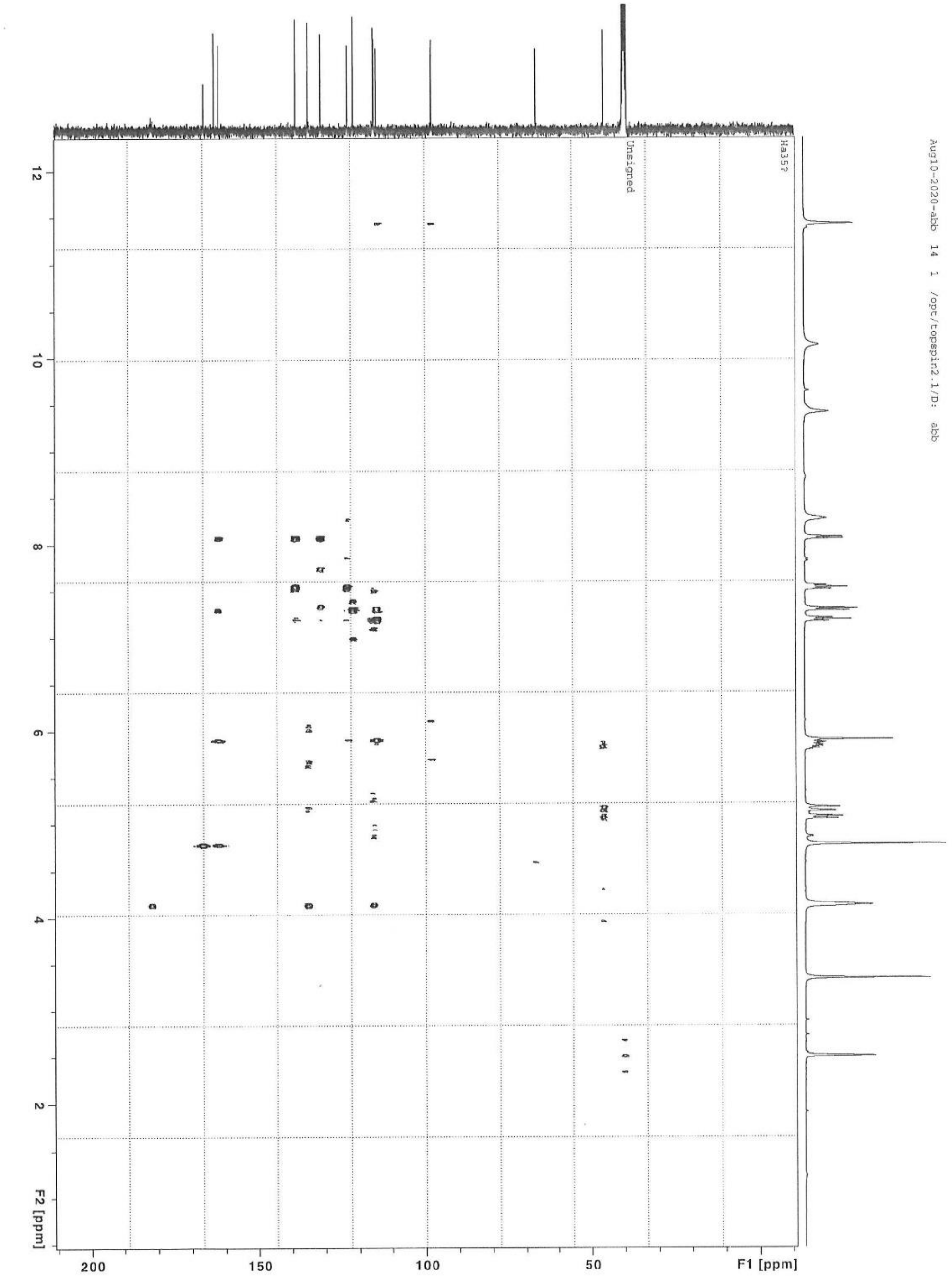

Figure S50. ^{1}H-^{13}C HMBC spectrum of compound **7a.**

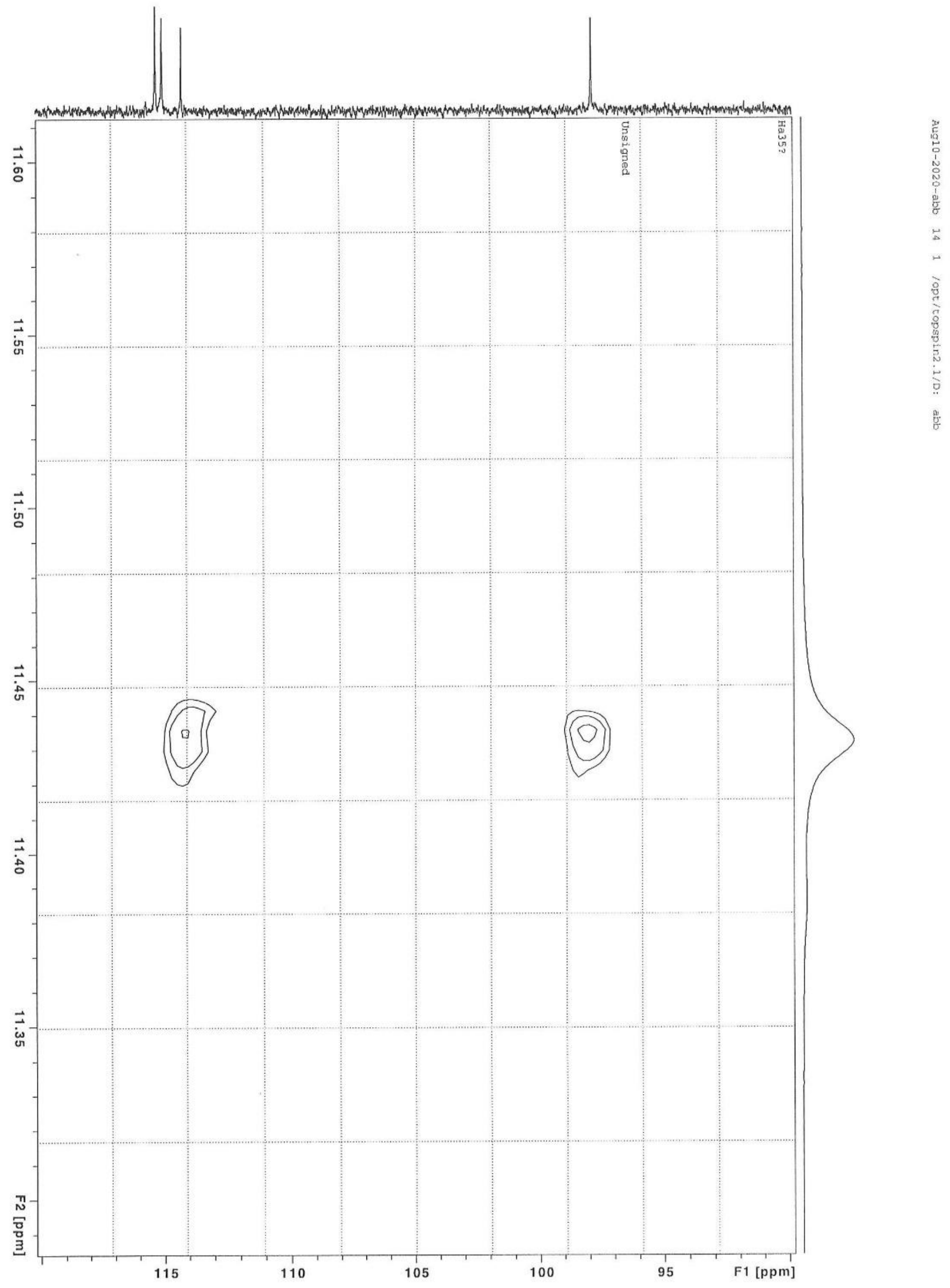

Figure S51. Detail of ^{1}H-^{13}C HMBC spectrum of compound **7a.**

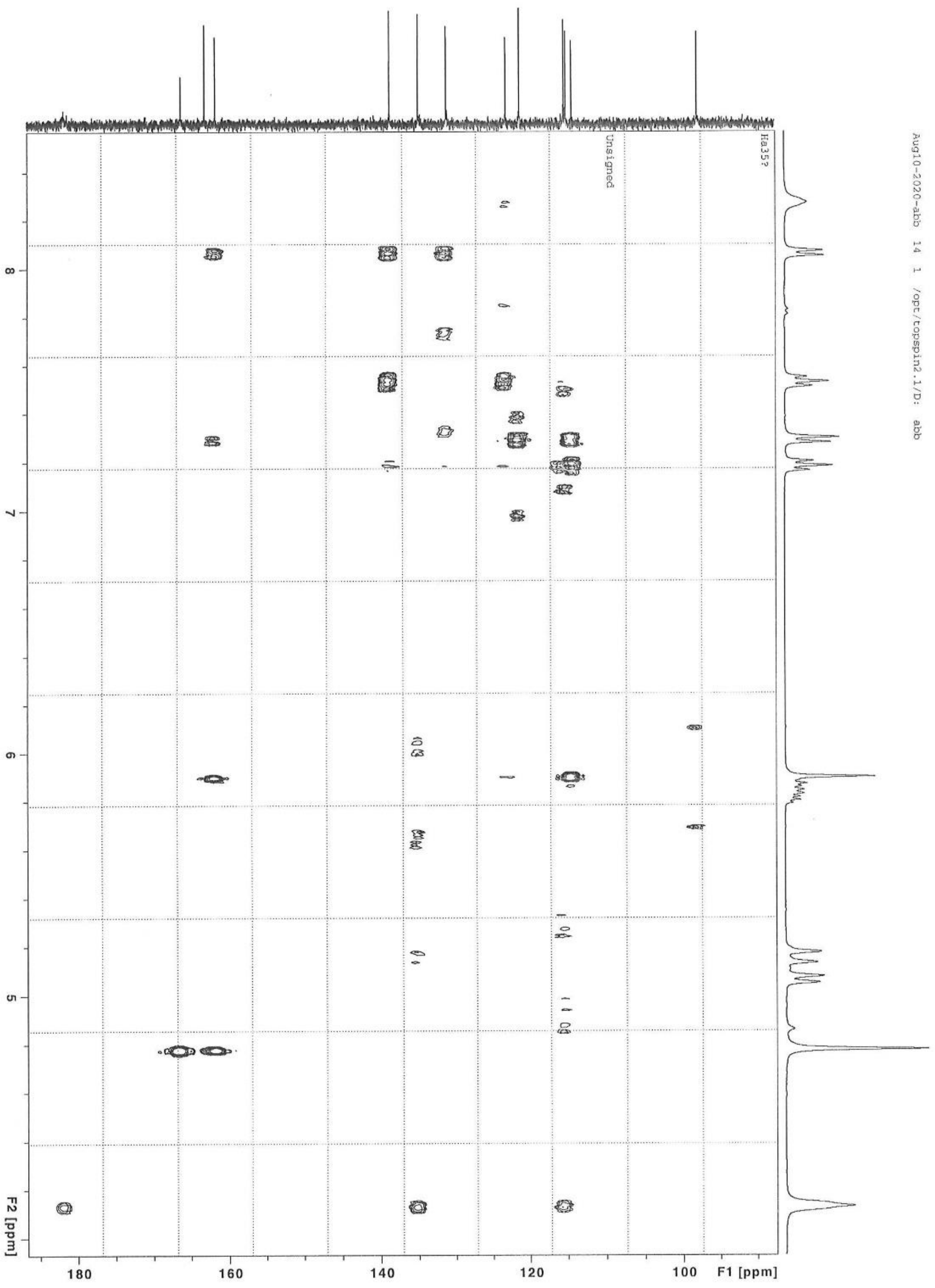

Figure S52. Detail of ^{1}H-^{13}C HMBC spectrum of compound **7a**

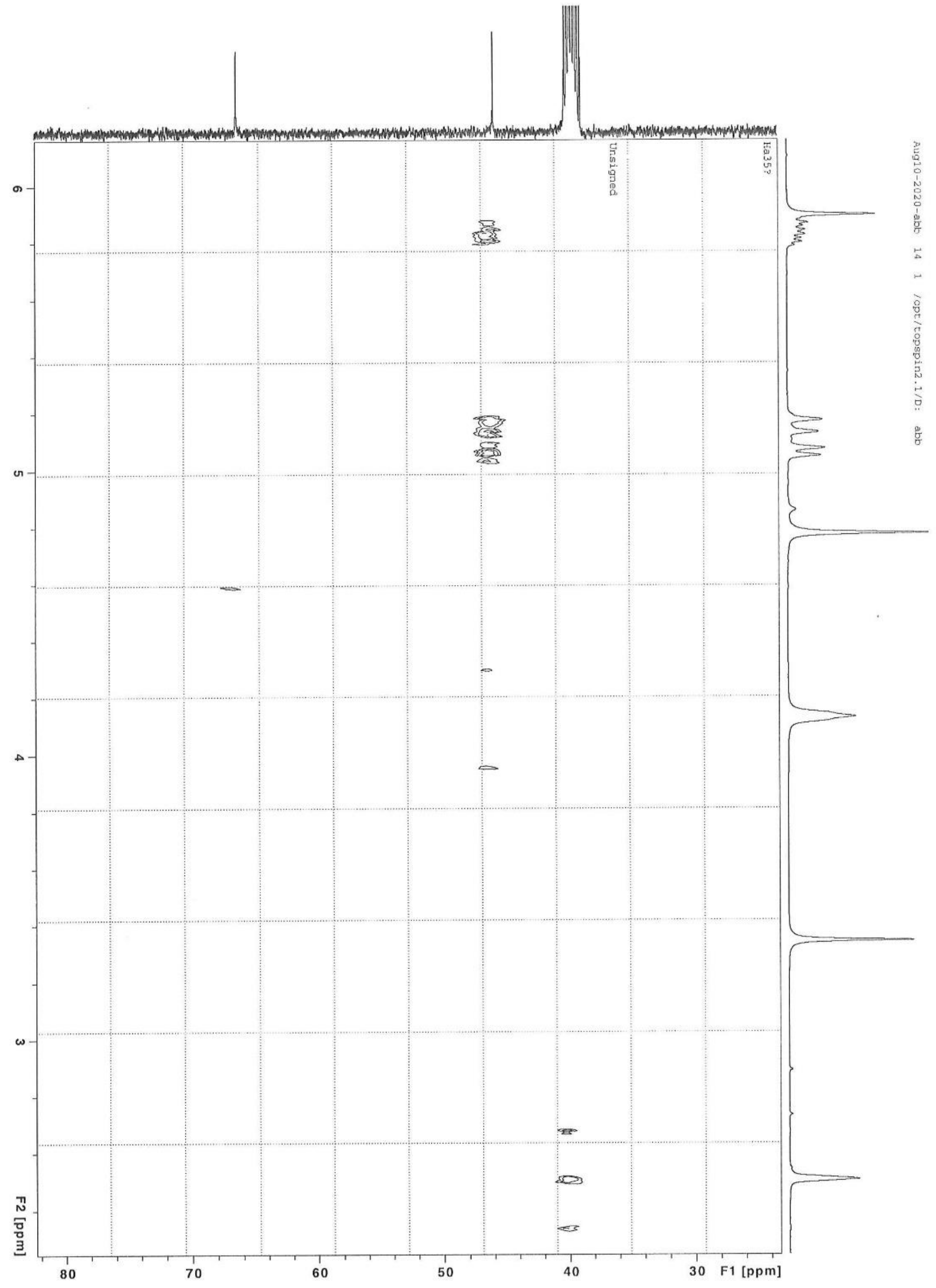

Figure S53. Detail of ^{1}H-^{13}C HMBC spectrum of compound **7a.**

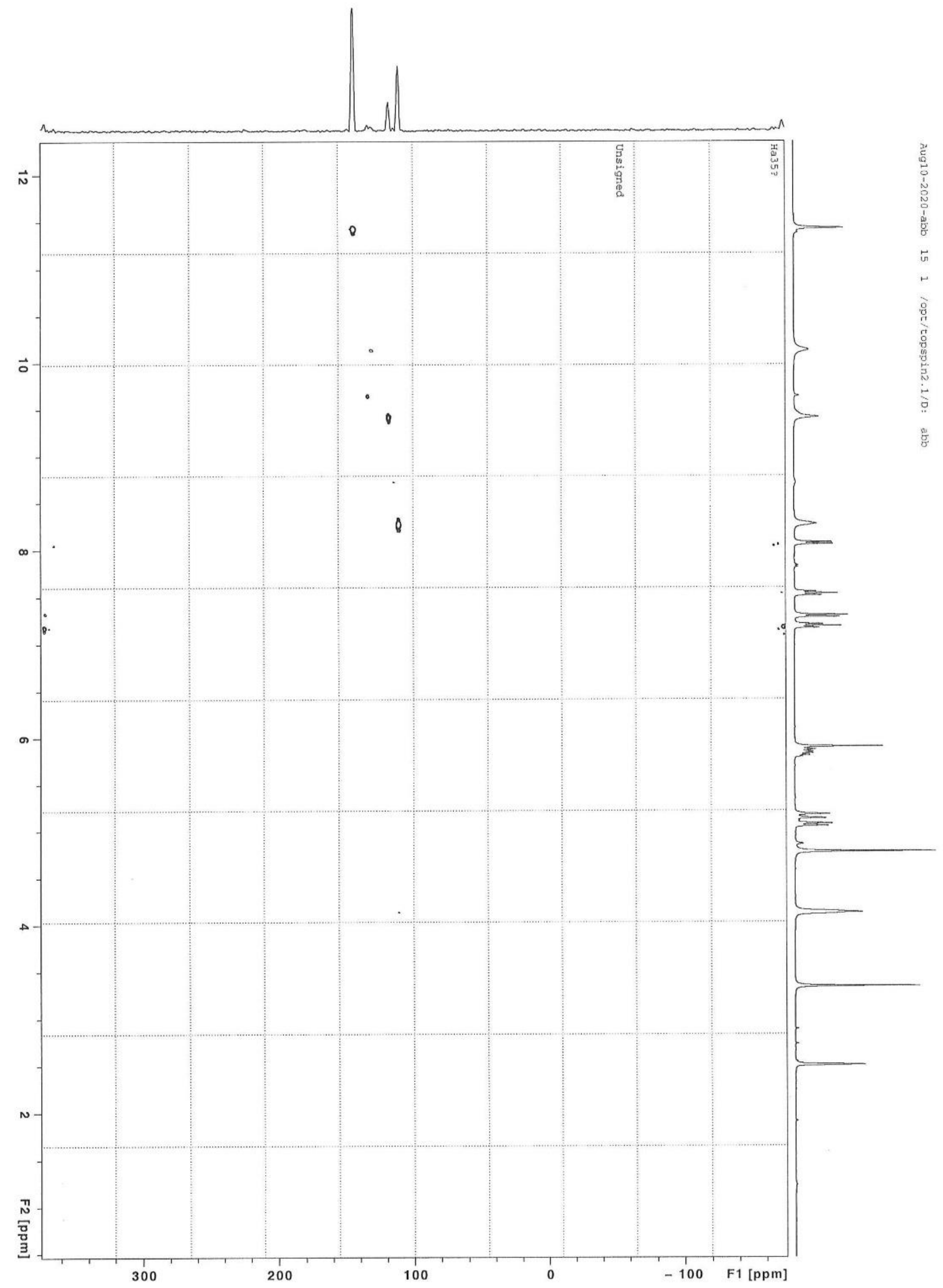

Figure S54. ^{1}H-^{15}N HSQC spectrum of compound **7a.**

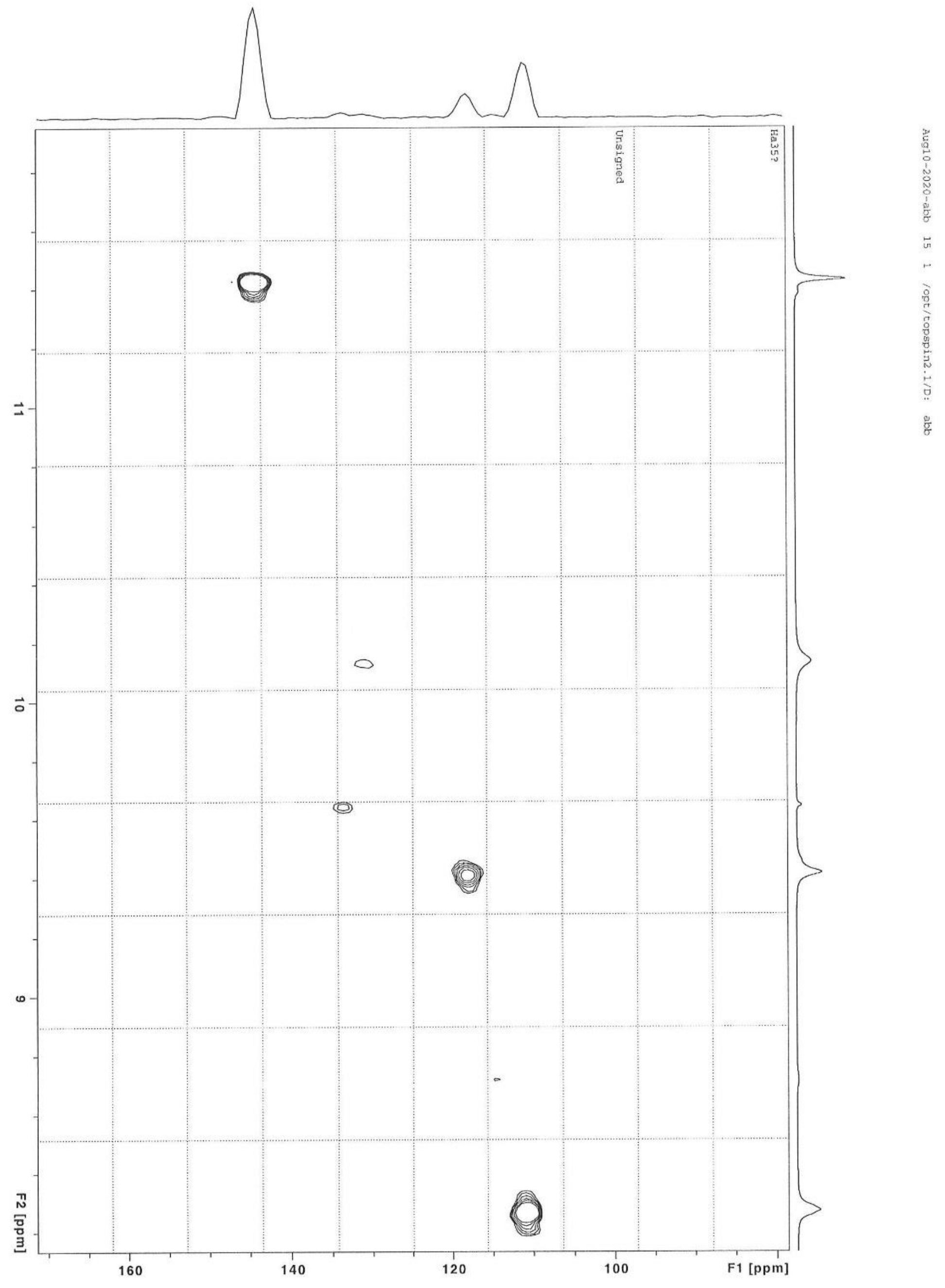

Figure S55. Detail of ^{1}H-^{15}N HSQC spectrum of compound **7a.**

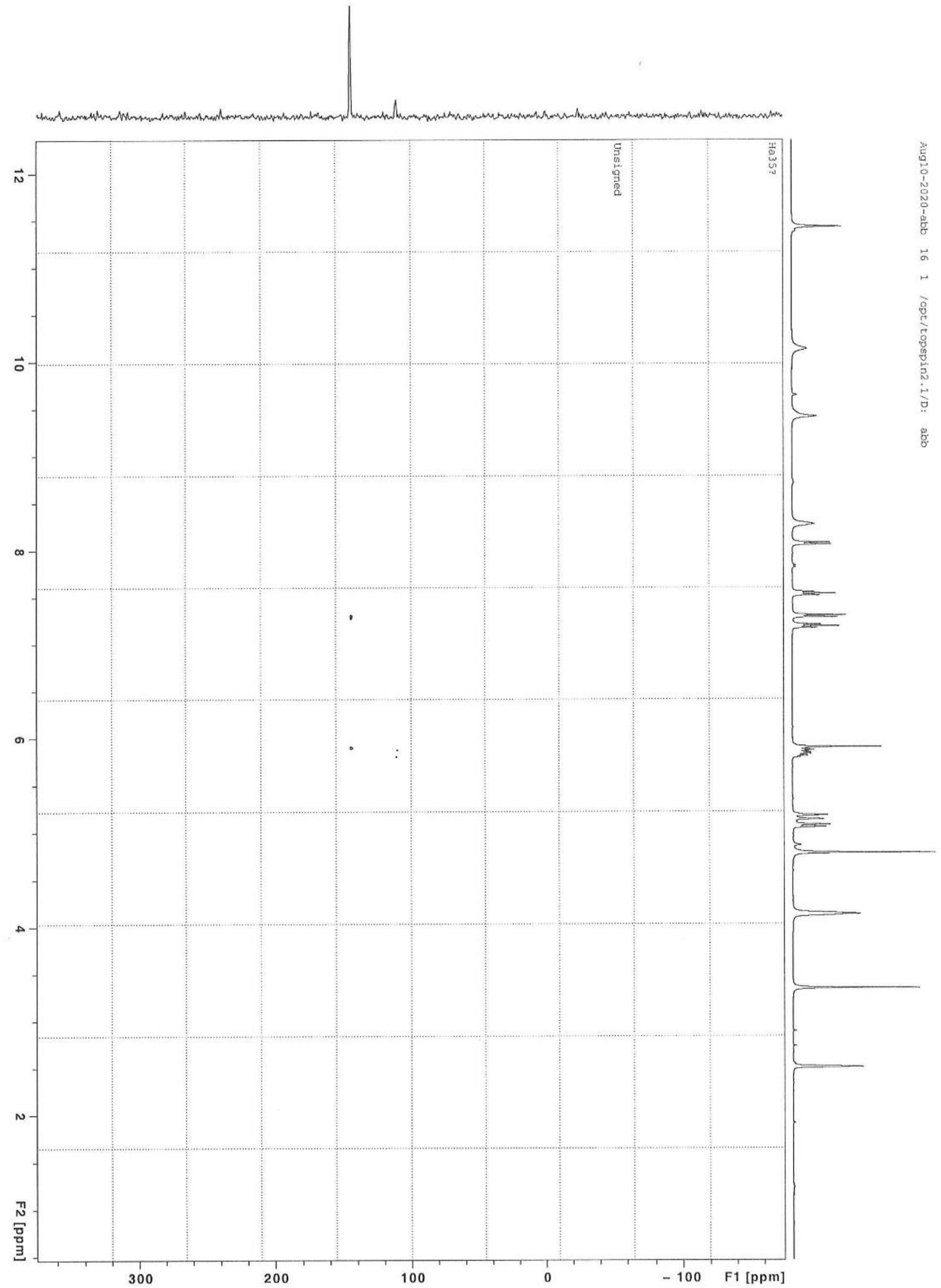

Figure S56. ^{1}H-^{15}N HMBC spectrum of compound **7a.**

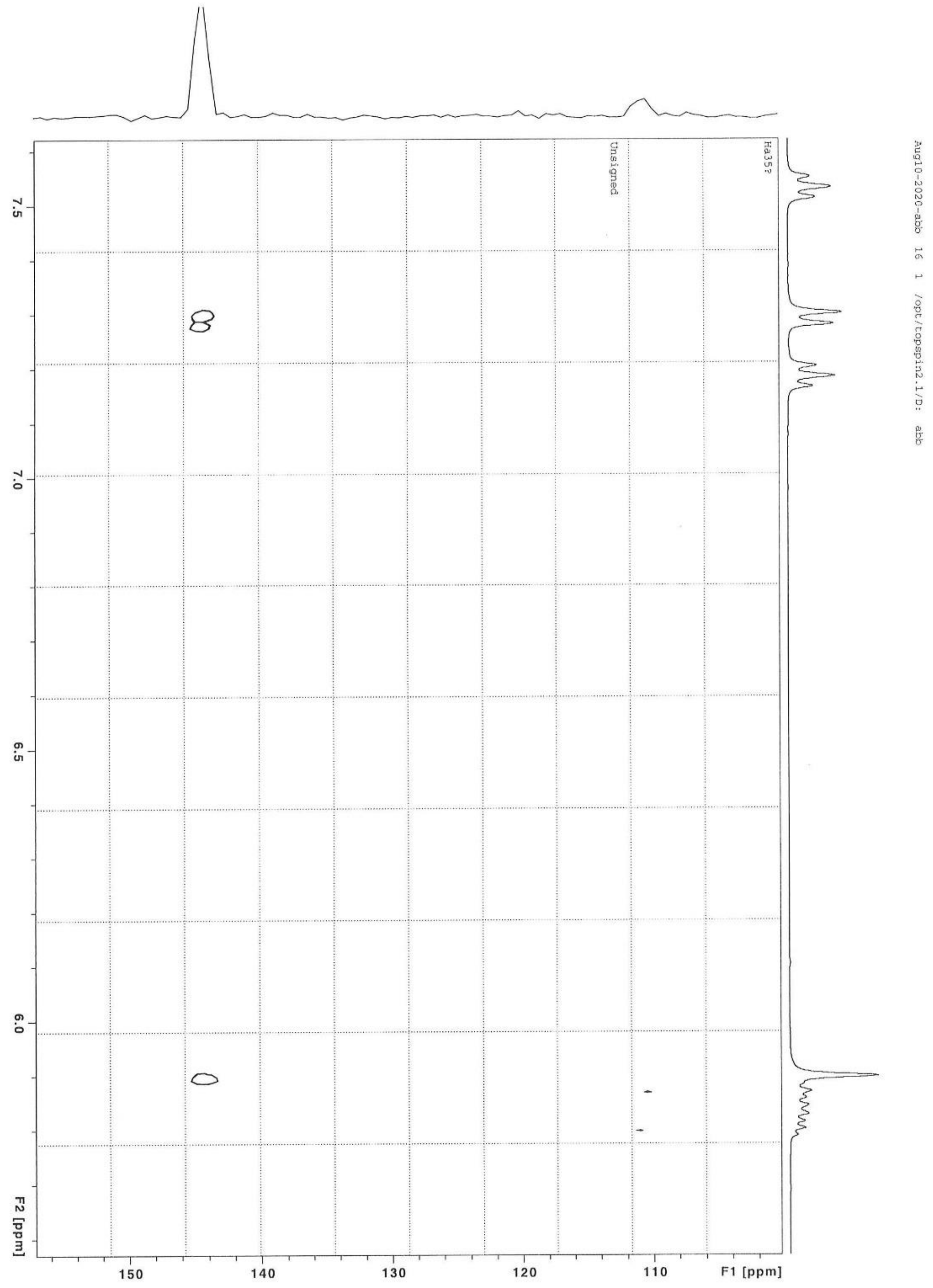

Figure S57. Detail of ${}^{1}H$-${}^{15}N$ HMBC spectrum of compound **7a.**

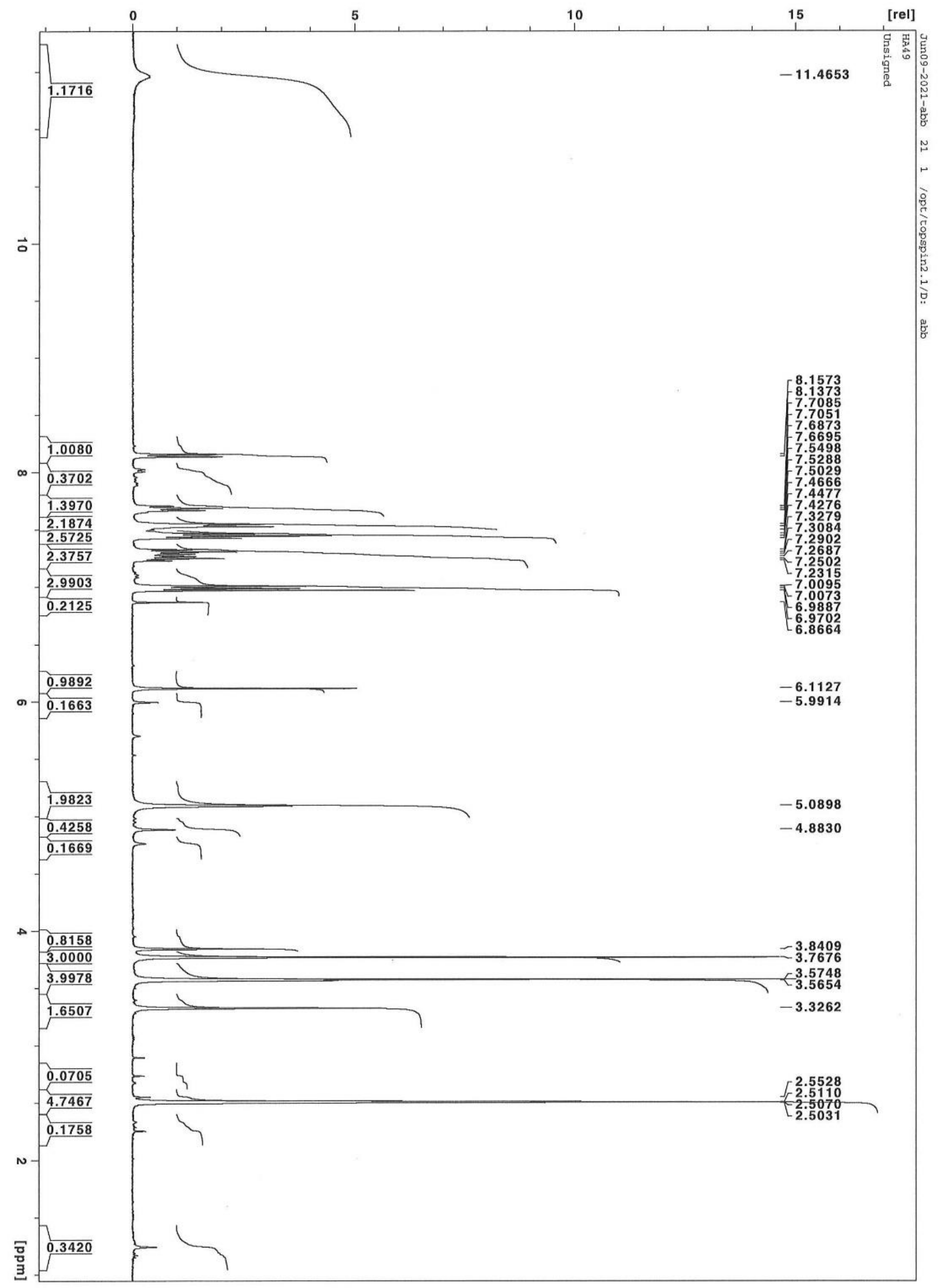

Figure S58. ^{1}H spectrum of compound **7c** (400 MHz, DMSO-d_6).

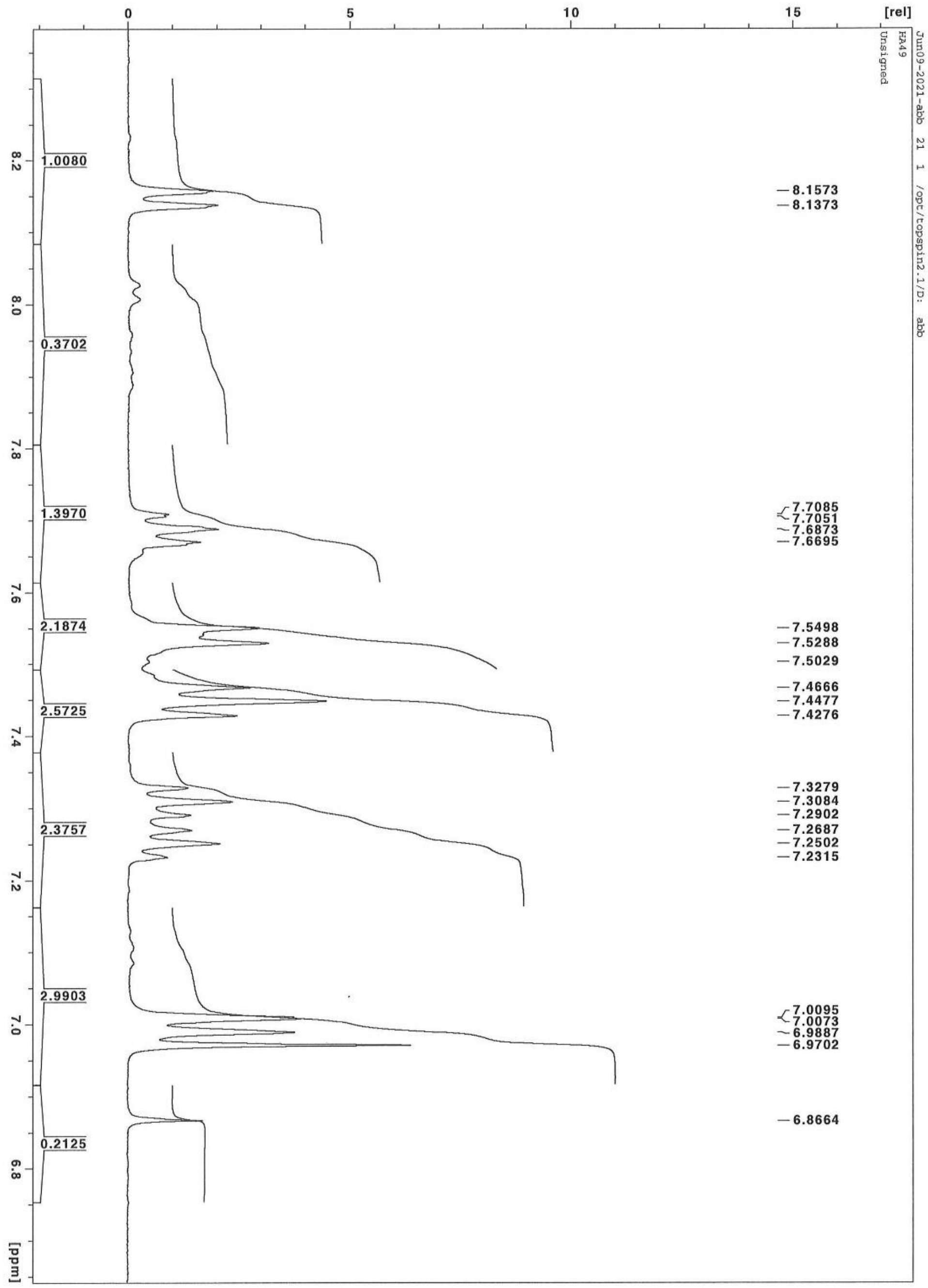

Figure S59. Detail of ^{1}H NMR spectrum of compound **7c.**

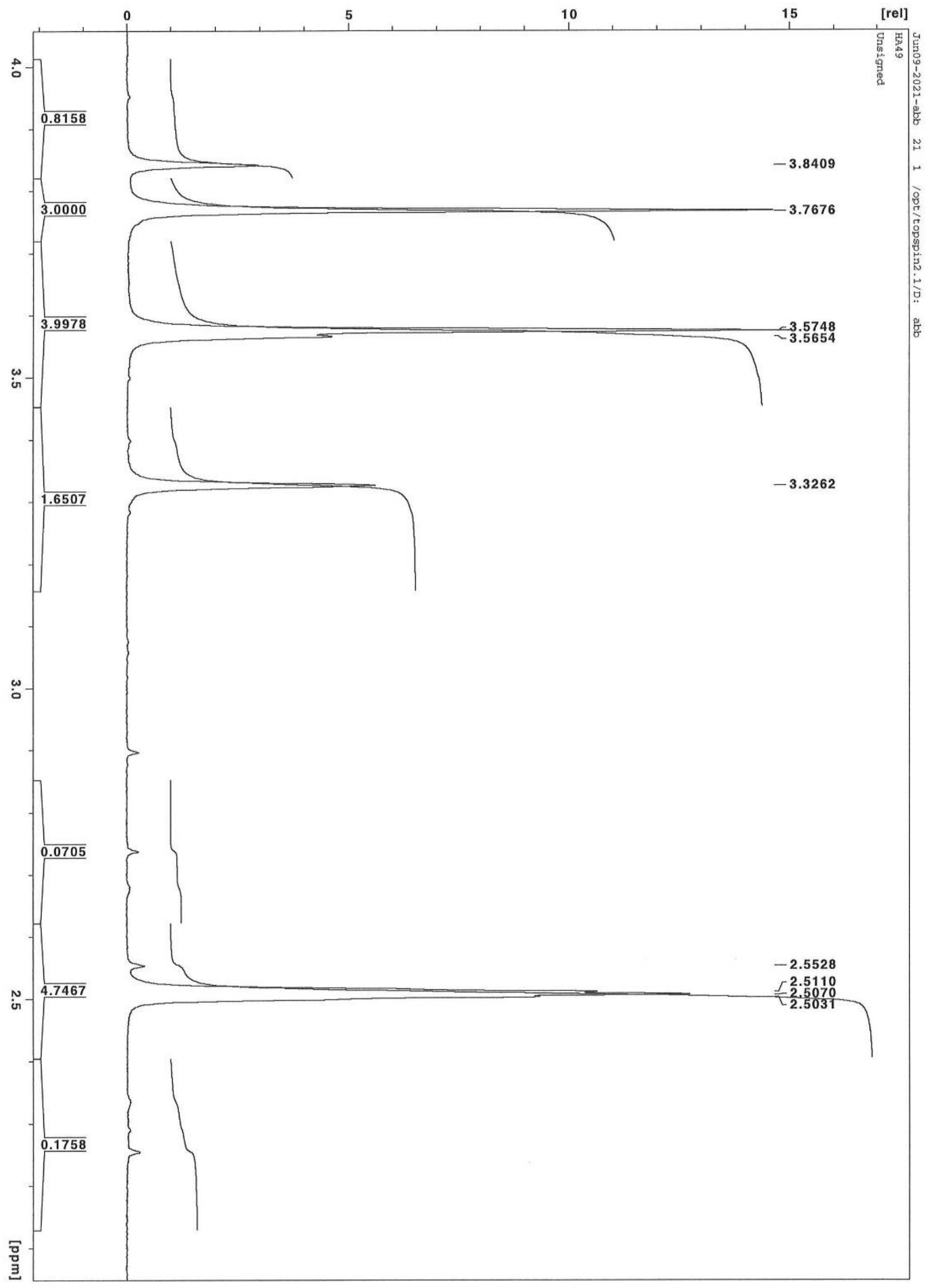

Figure S60. Detail of ^{1}H NMR spectrum of compound **7c.**

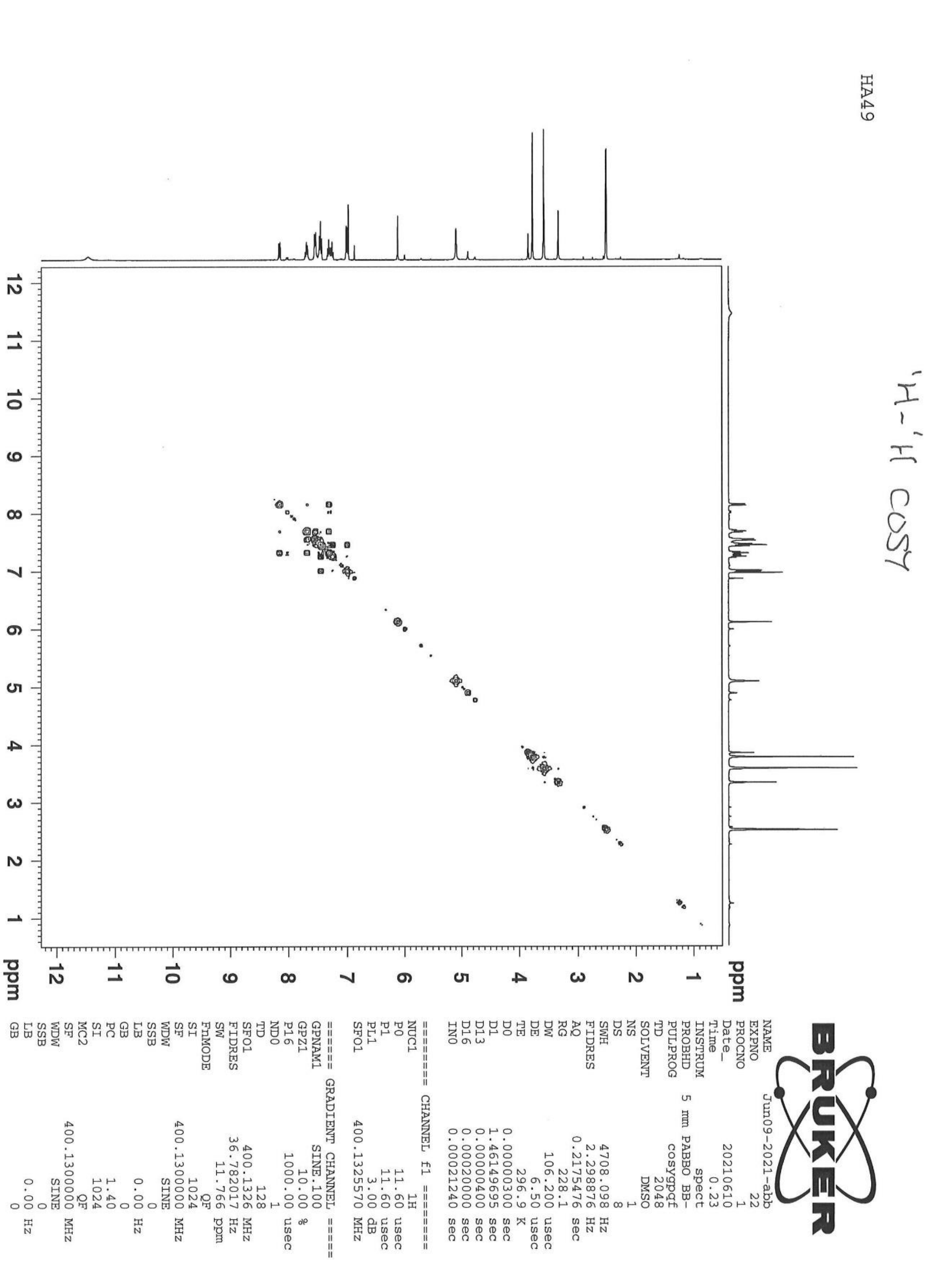

Figure S61. ^{1}H-^{1}H COSY spectrum of compound **7c.**

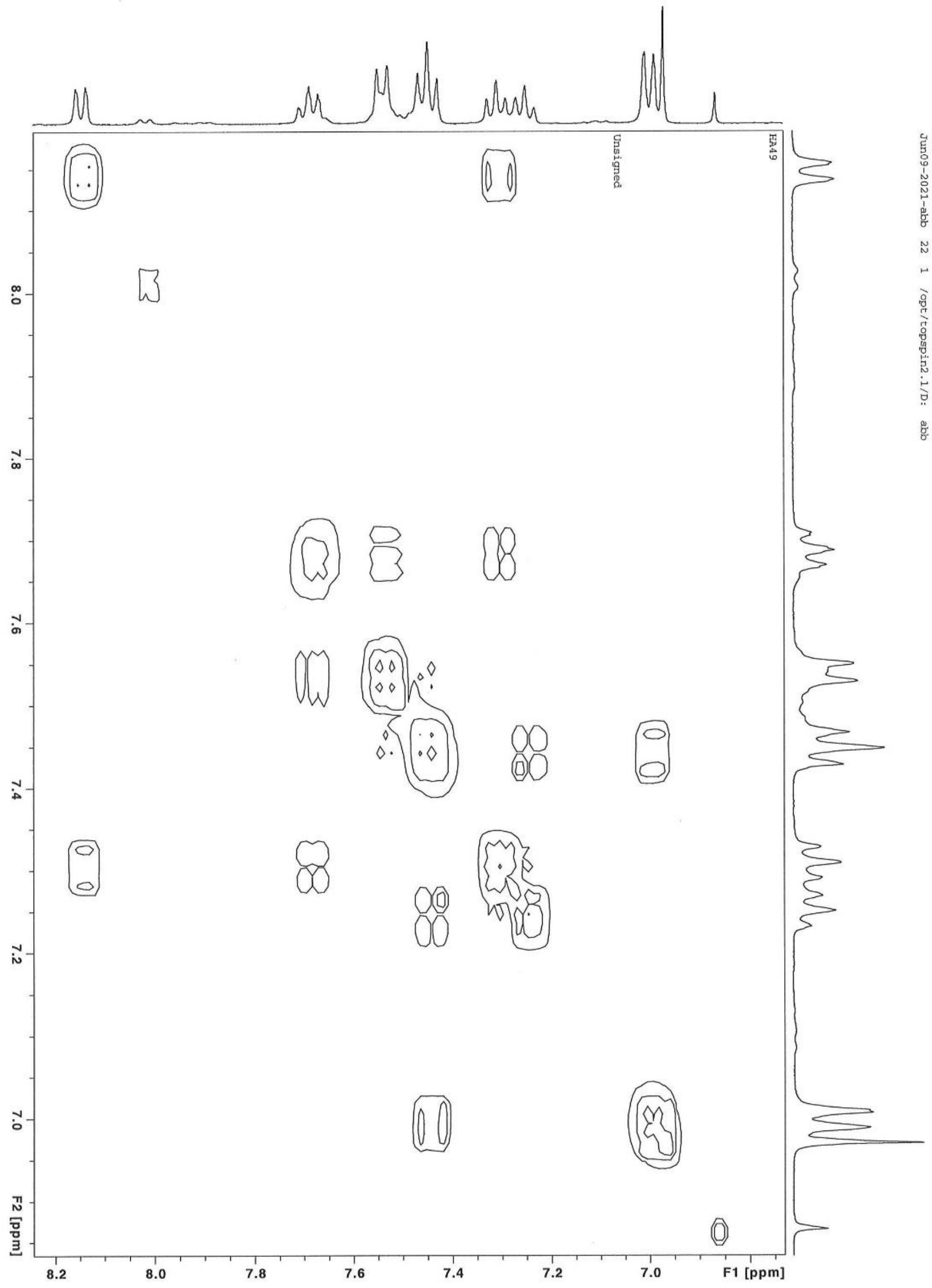

Figure S62. Detail of ^{1}H-^{1}H COSY spectrum of compound **7c.**

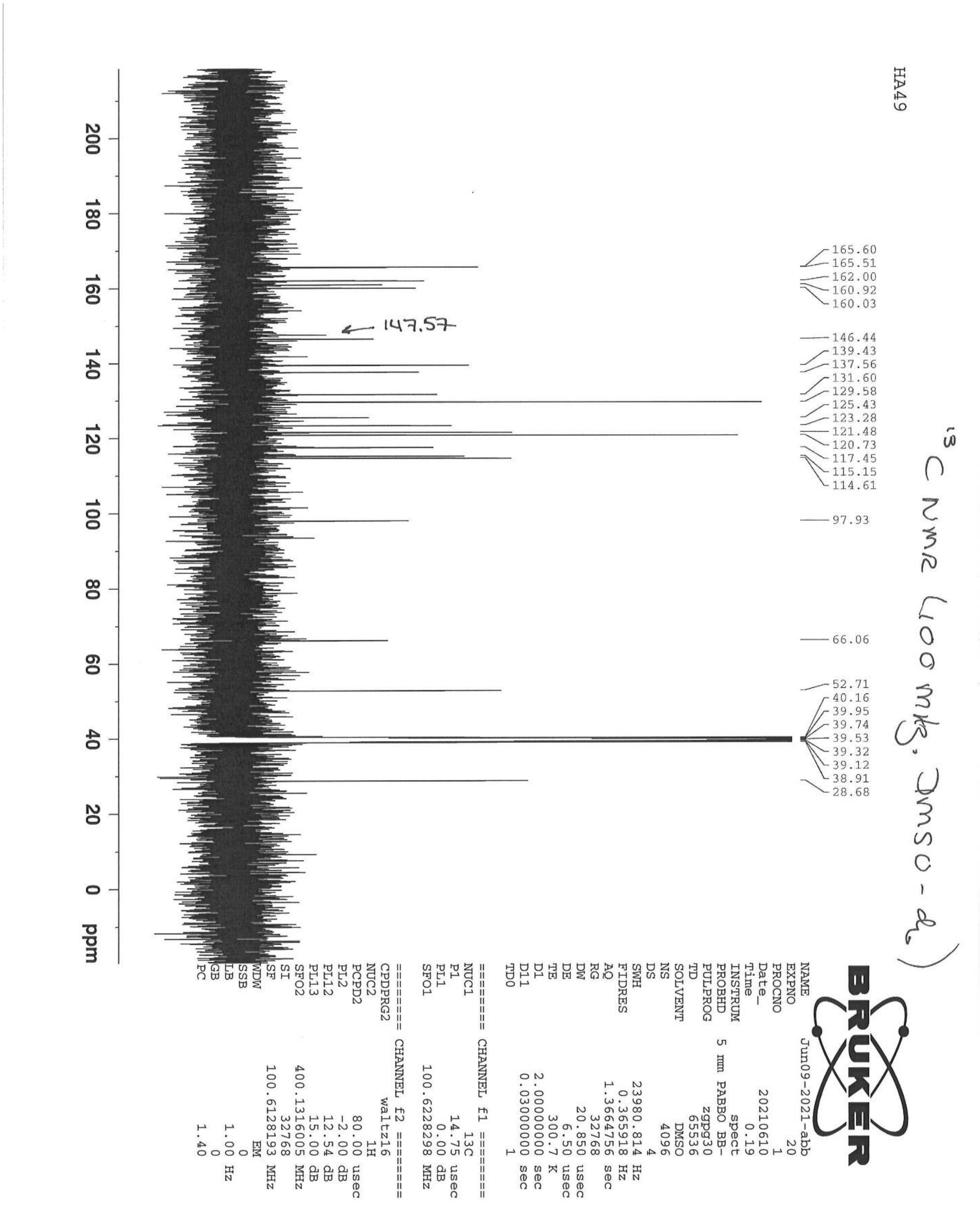

Figure S63. ^{13}C NMR spectrum of compound **7c** (100 MHz, DMSO-d_6).

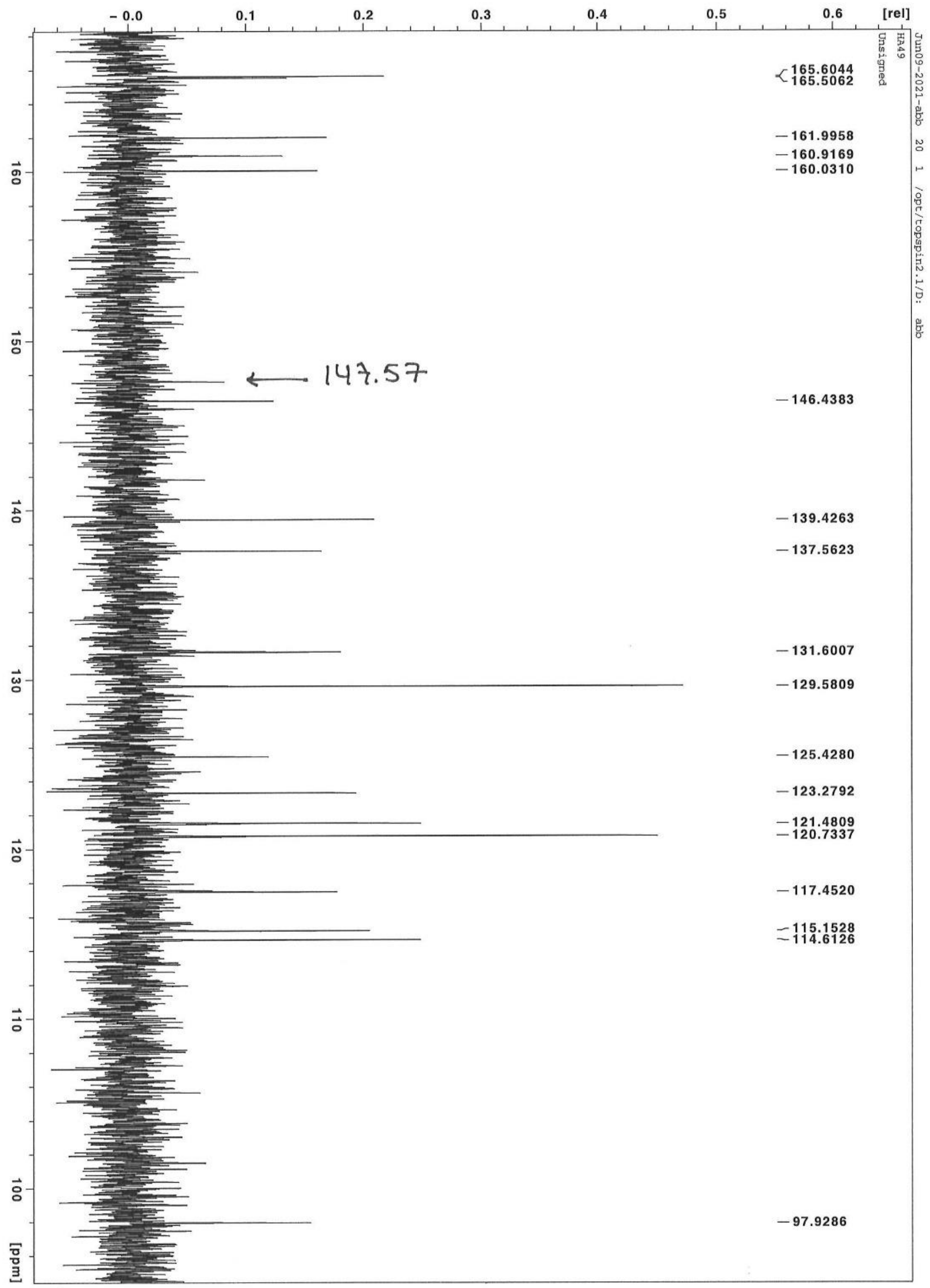

Figure S64. Detail of ^{13}C NMR spectrum of compound **7c.**

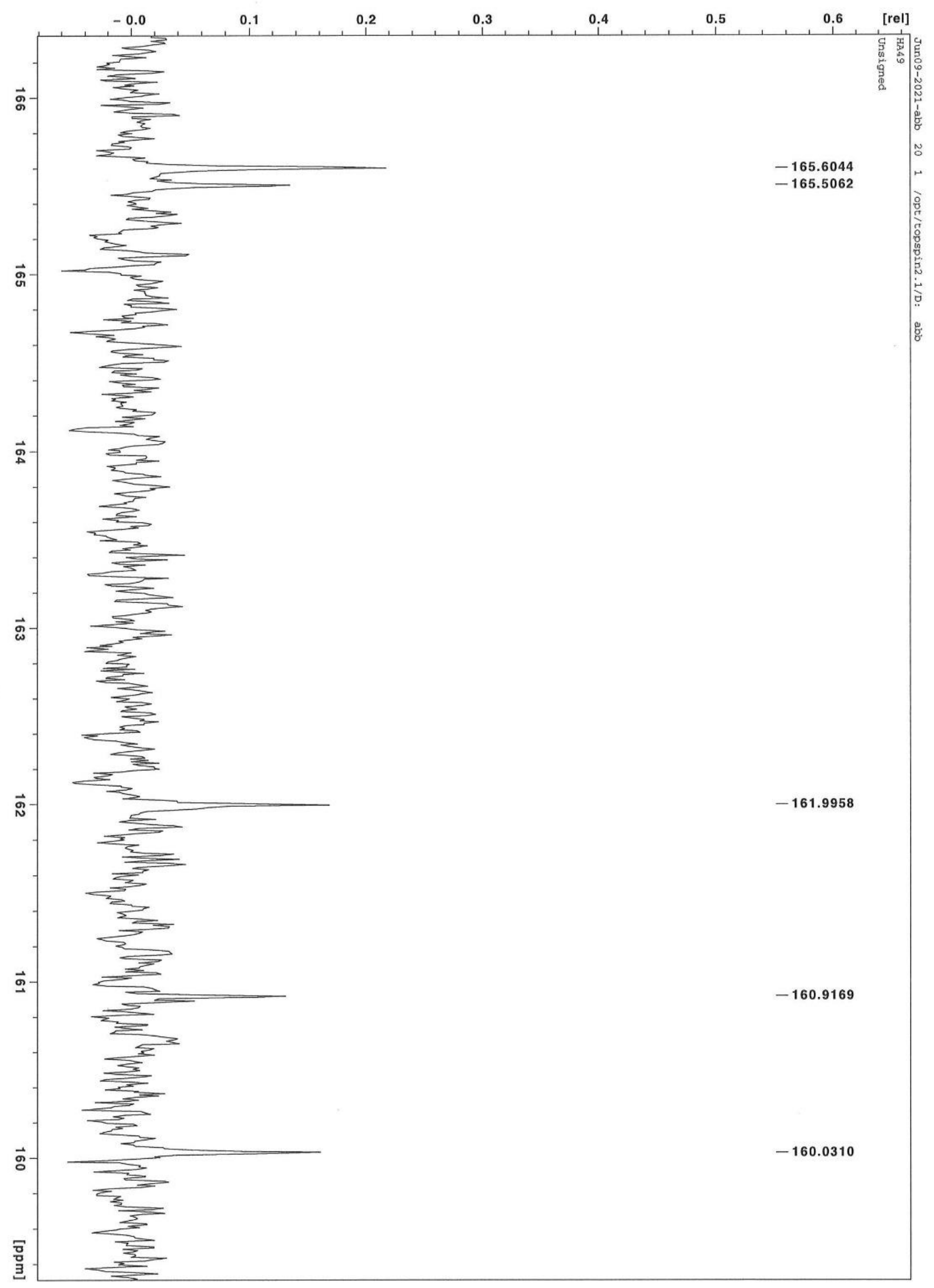

Figure S65. Detail of ^{13}C NMR spectrum of compound **7c.**

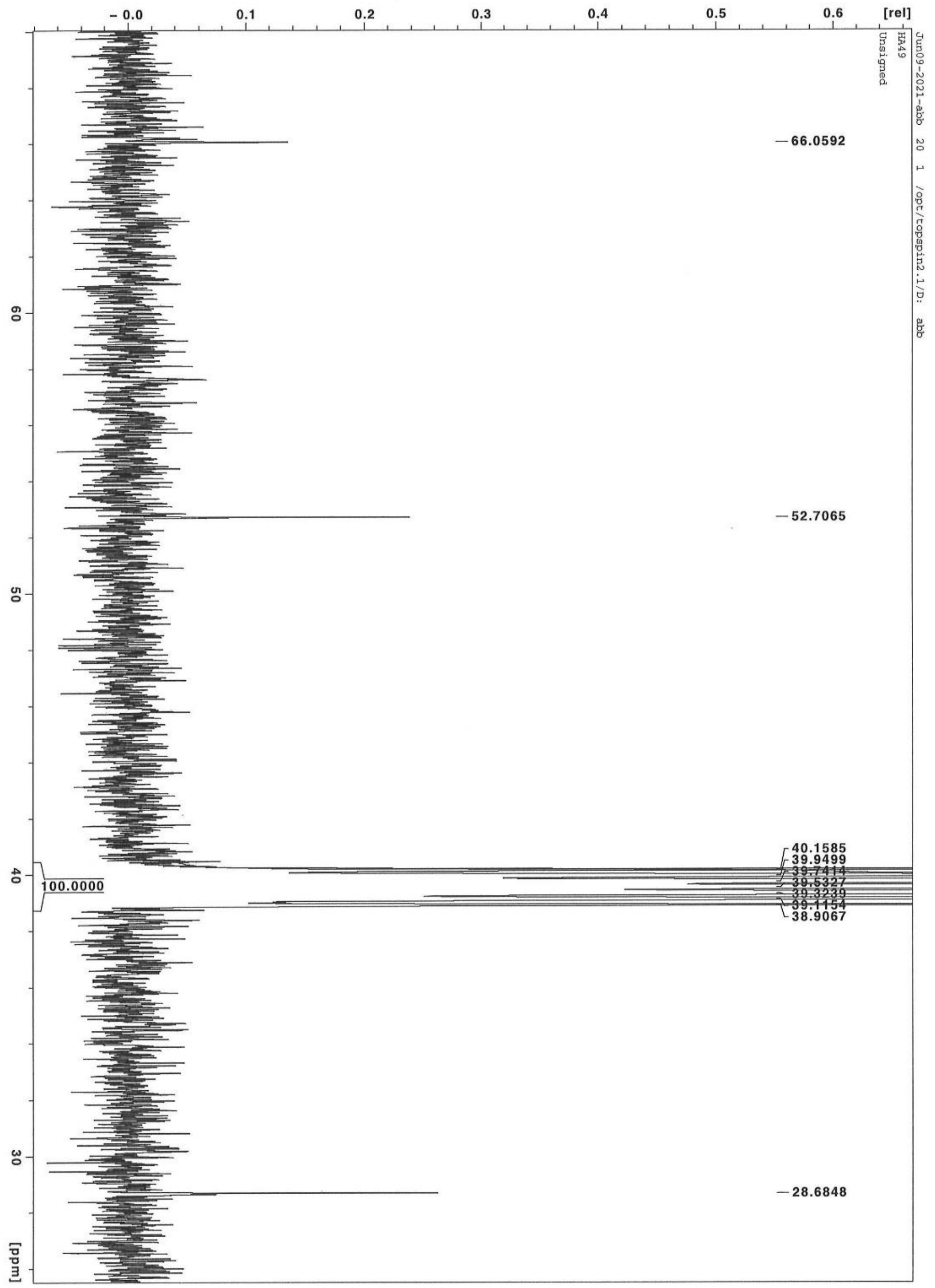

Figure S66. Detail of ^{13}C NMR spectrum of compound **7c.**

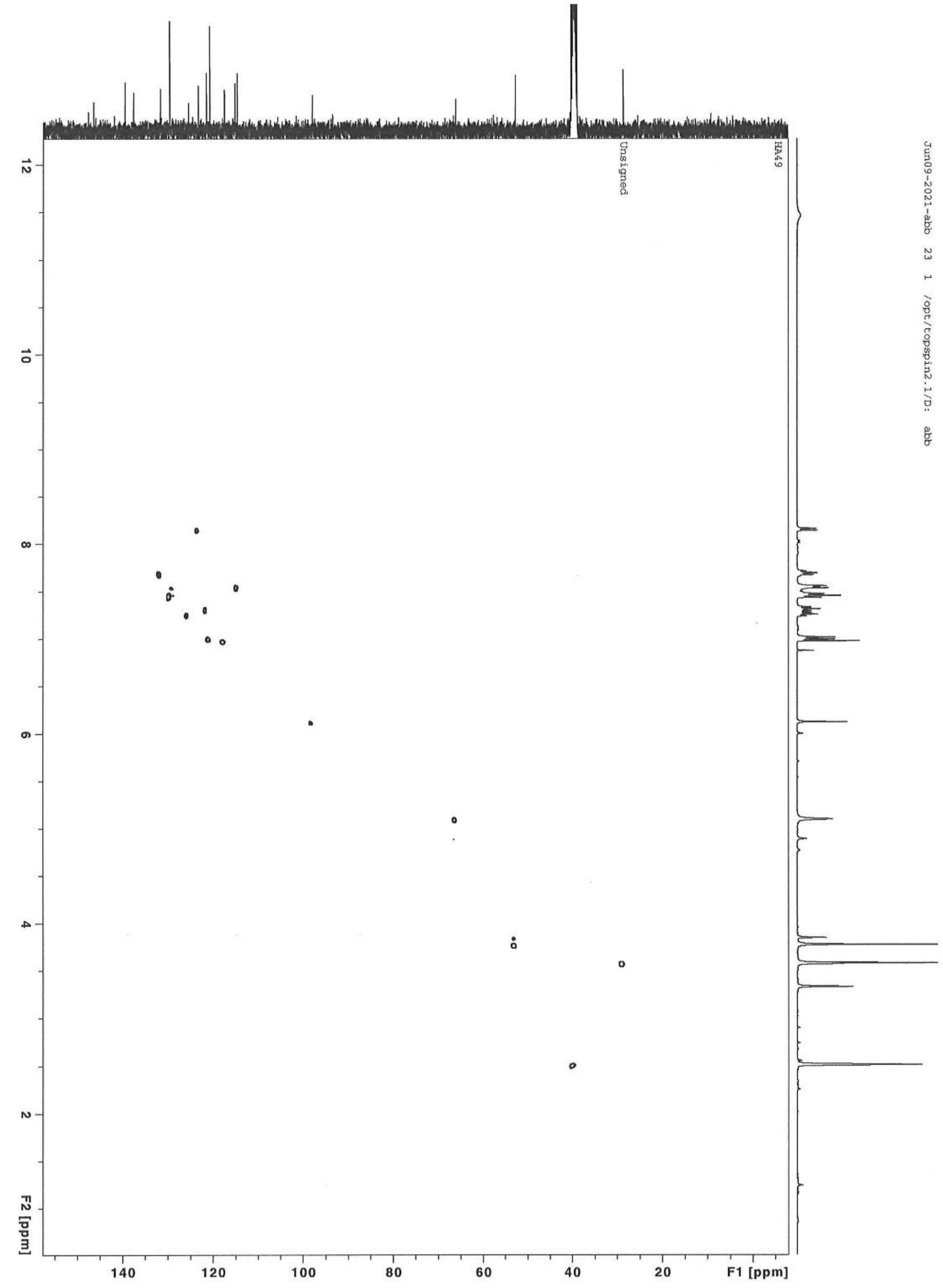

Figure S67. ^{1}H-^{13}C HSQC spectrum of compound **7c.**

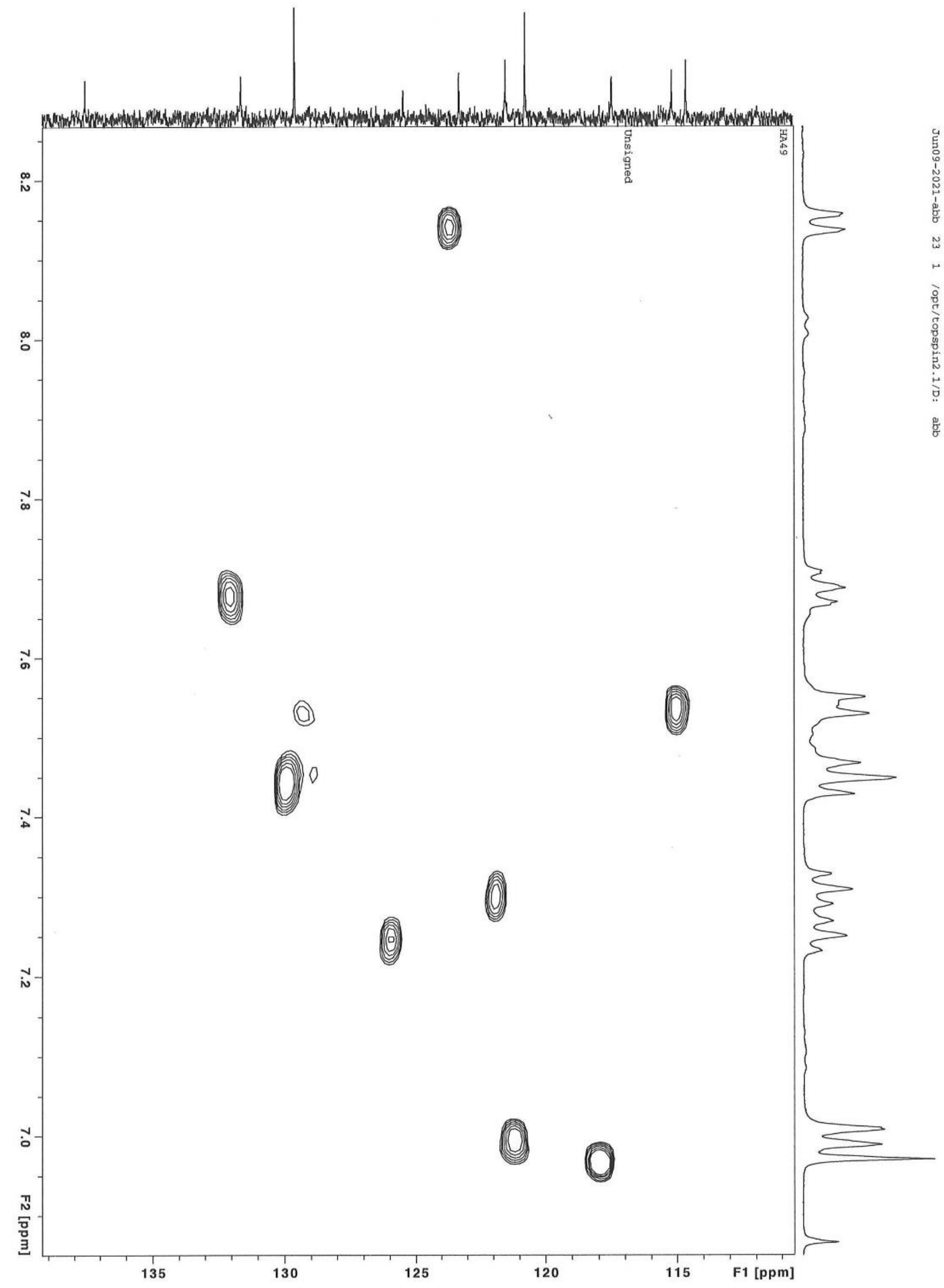

Figure S68. Detail of ^{1}H-^{13}C HSQC spectrum of compound **7c.**

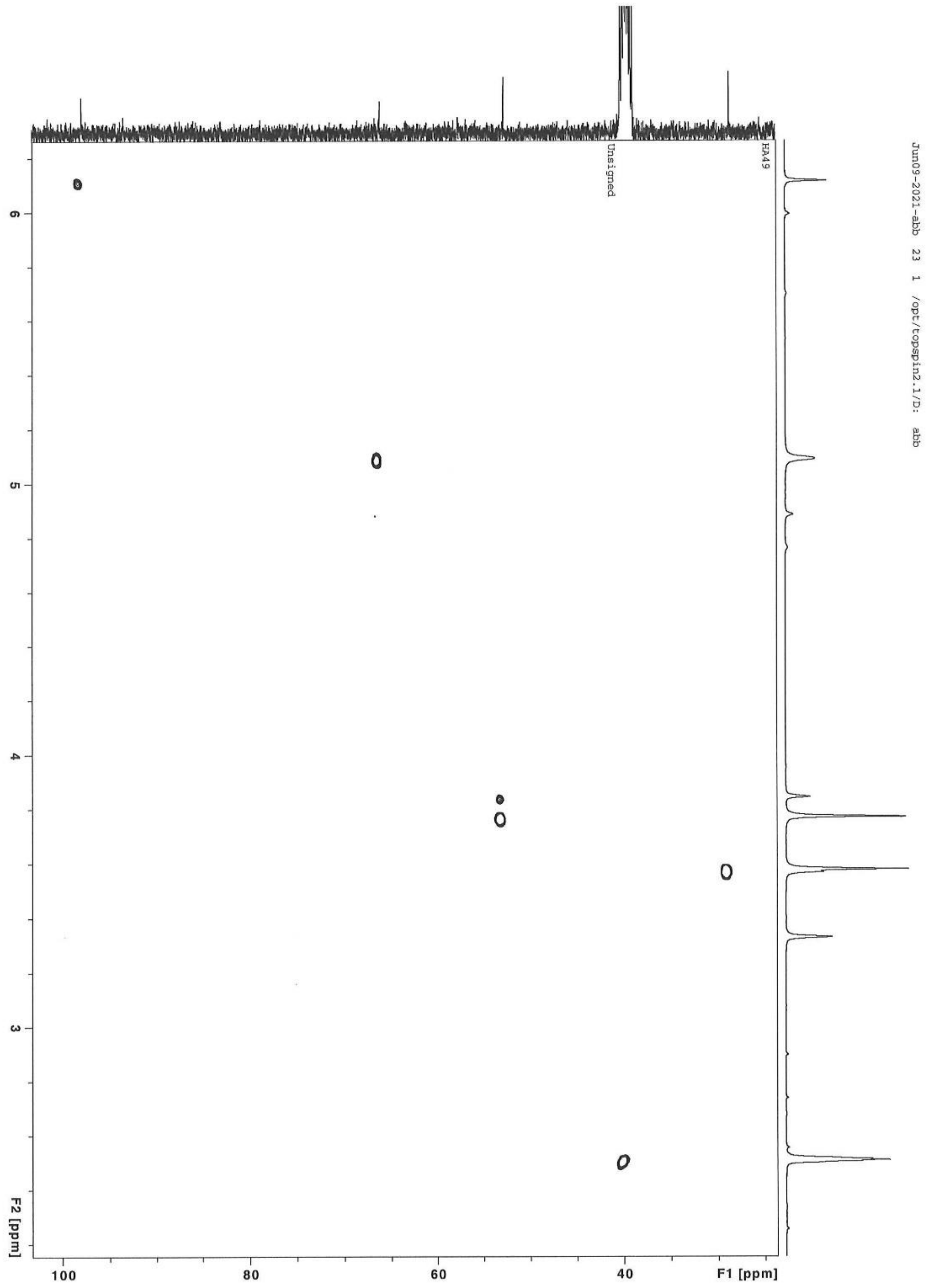

Figure S69. Detail of ^{1}H-^{13}C HSQC spectrum of compound **7c.**

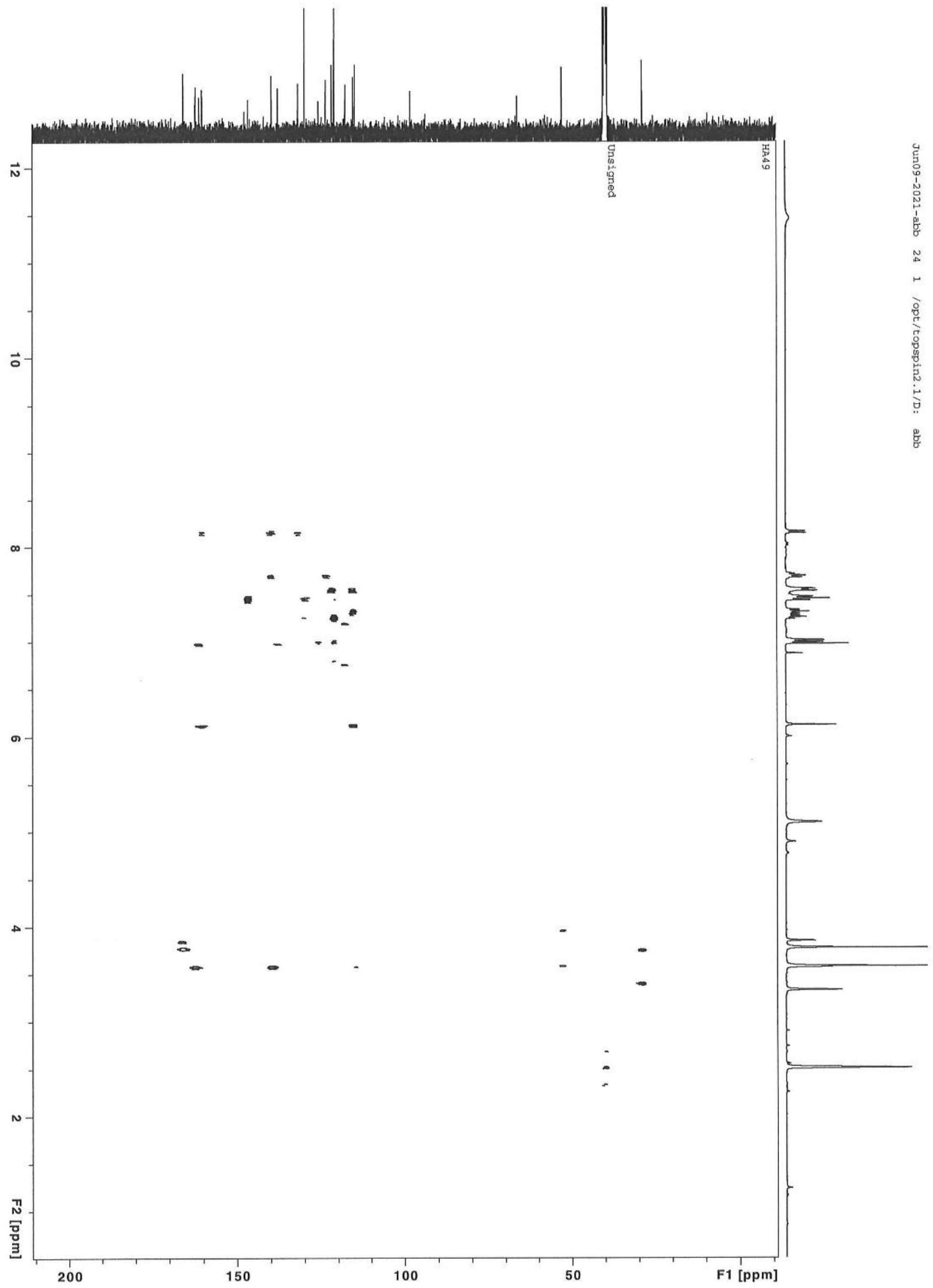

Figure S70. ^{1}H-^{13}C HMBC spectrum of compound **7c.**

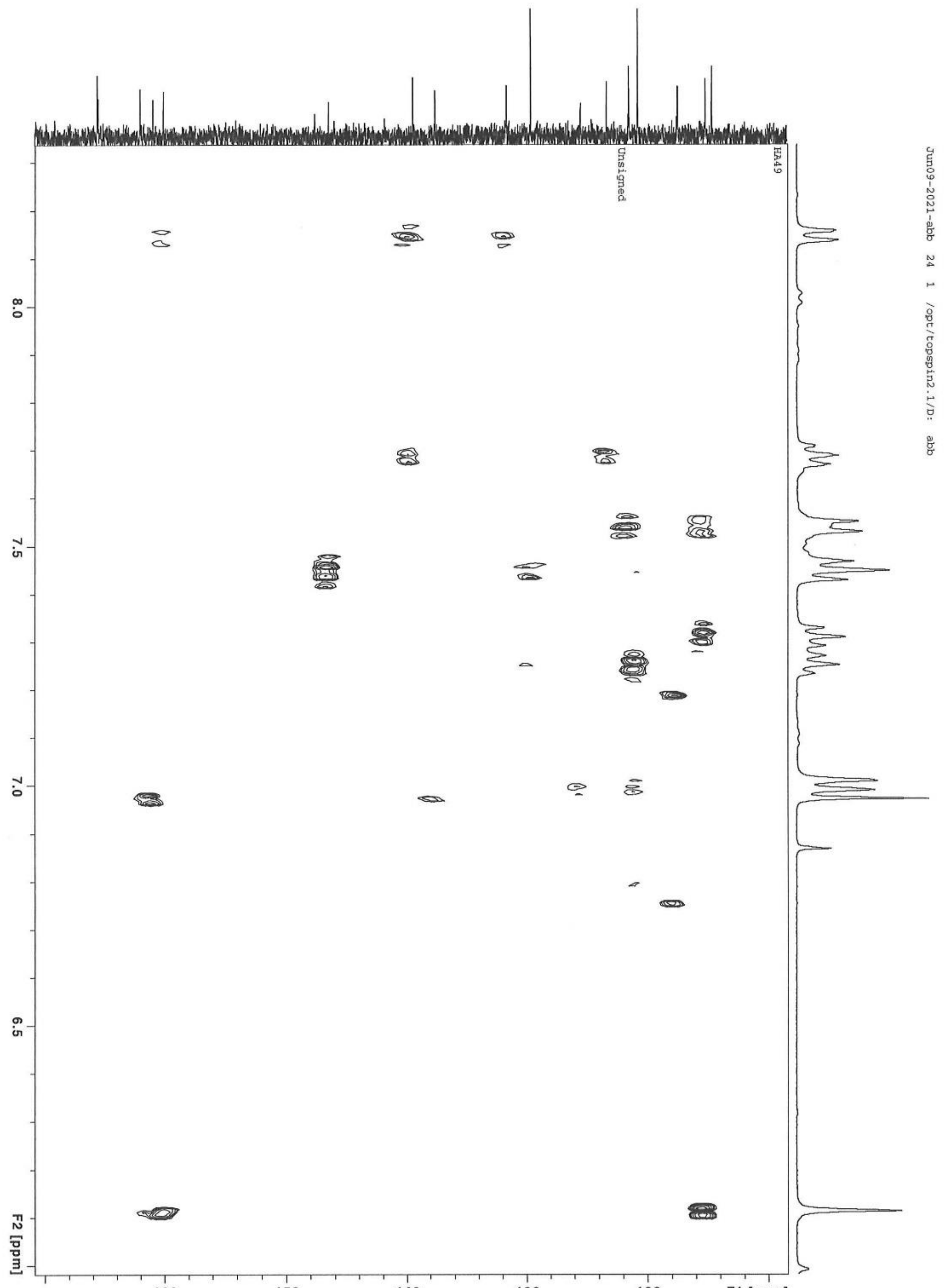

Figure S71. Detail of ^{1}H-^{13}C HMBC spectrum of compound **7c.**

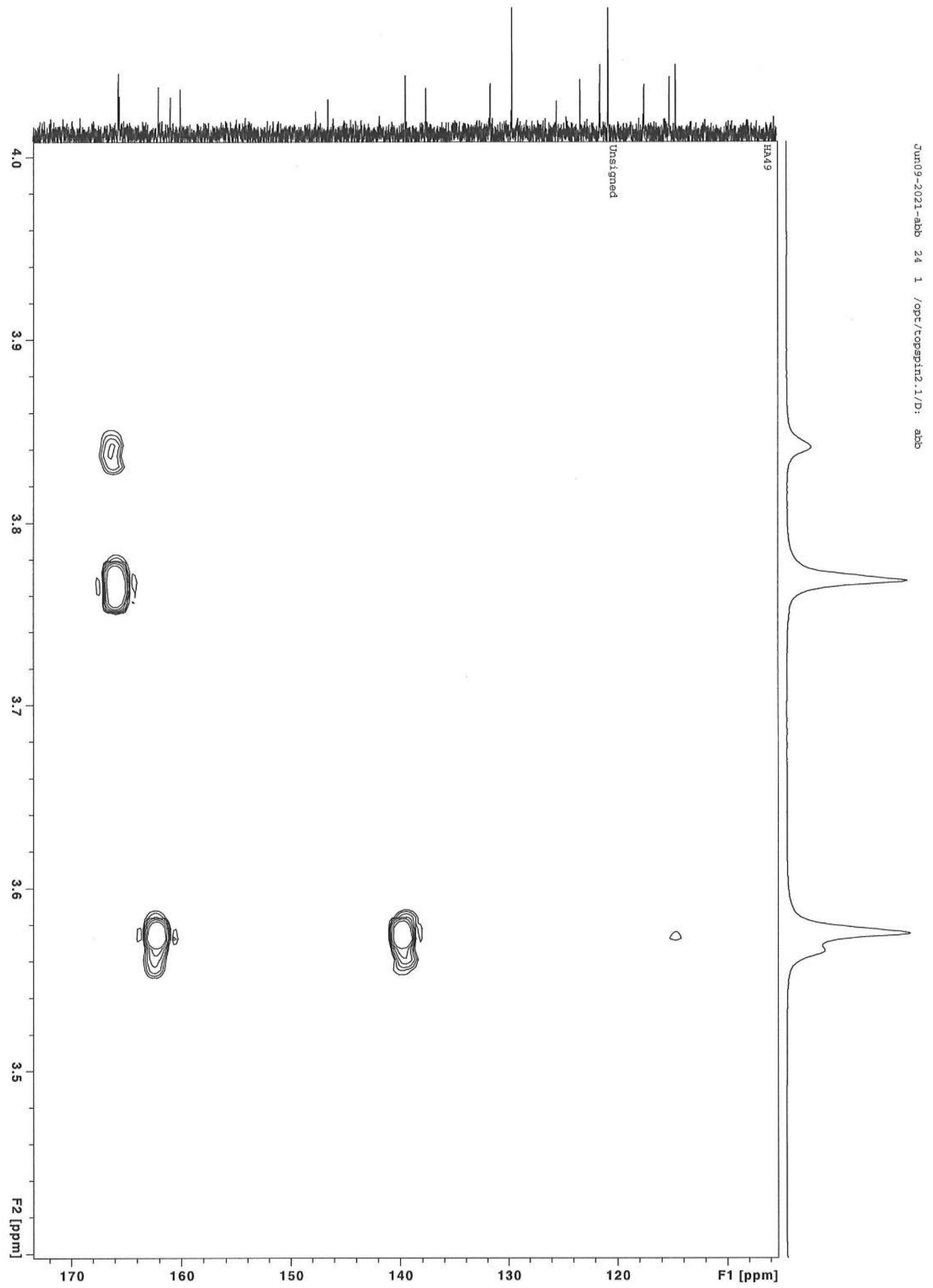

Figure S72. Detail of ^{1}H-^{13}C HMBC spectrum of compound **7c.**

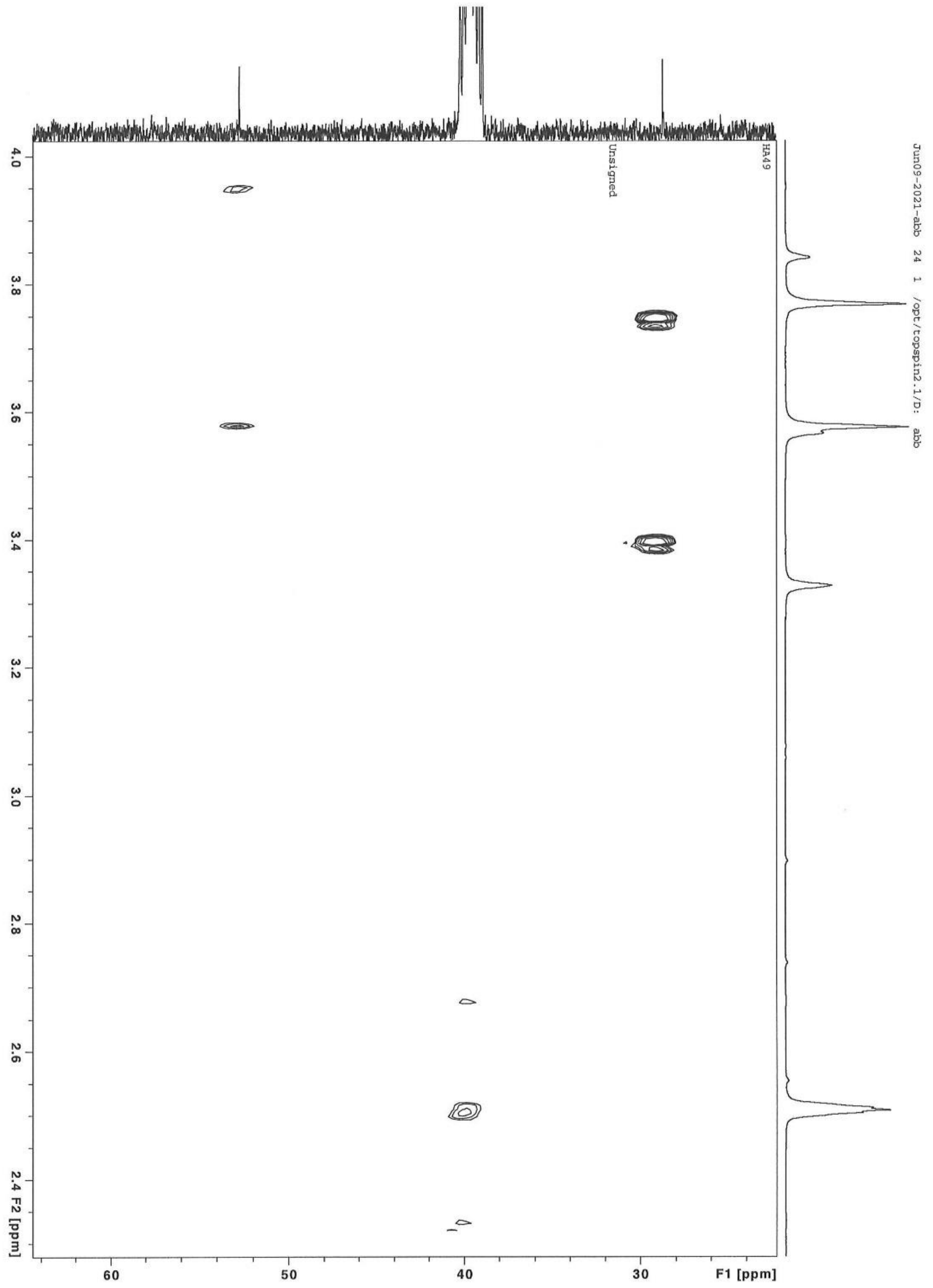

Figure S73. Detail of ^{1}H-^{13}C HMBC spectrum of compound **7c.**

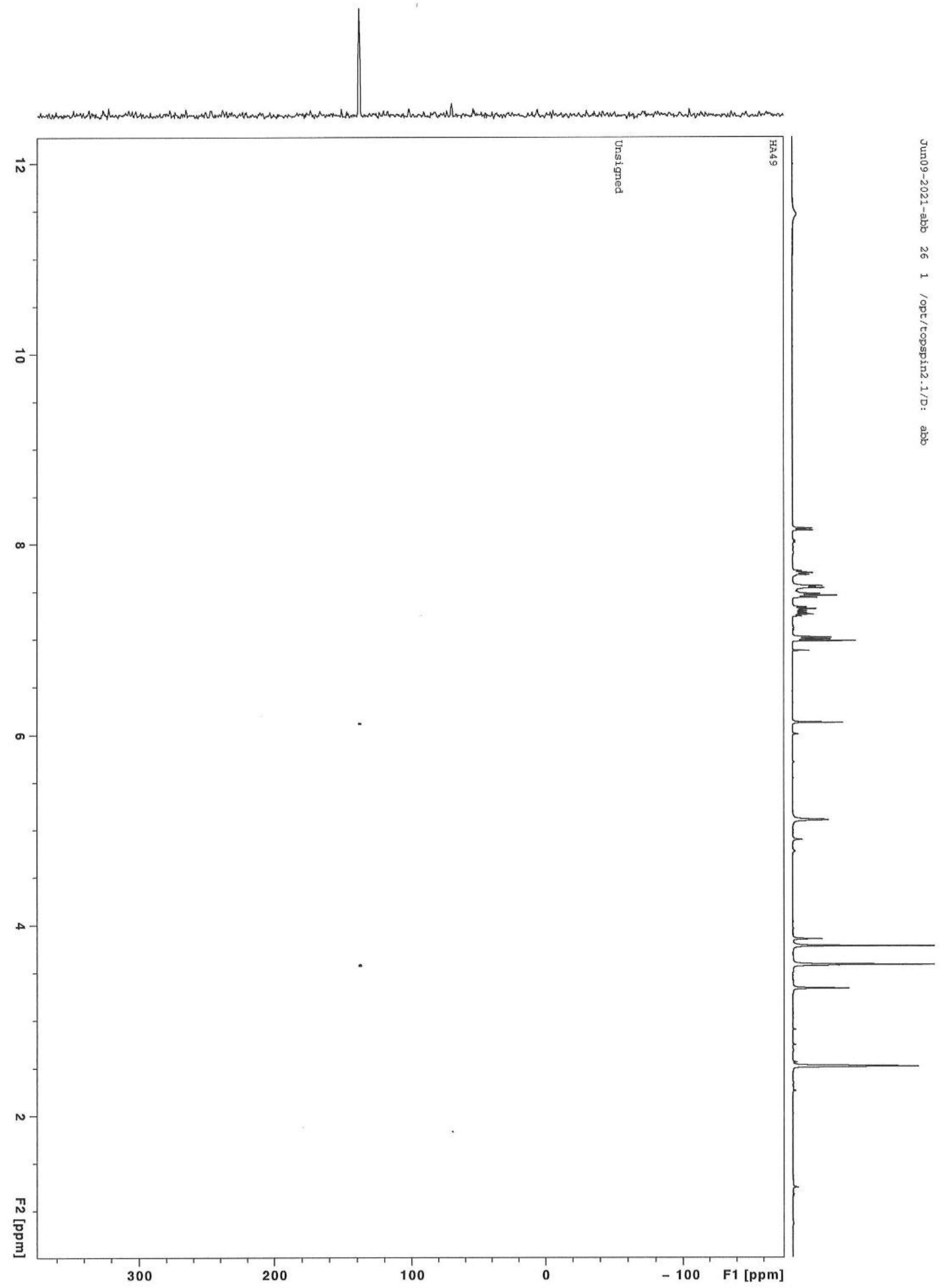

Figure S74. ^{1}H-^{15}N HMBC spectrum of compound **7c** (^{1}H-^{15}N experiment gave no cross peaks).

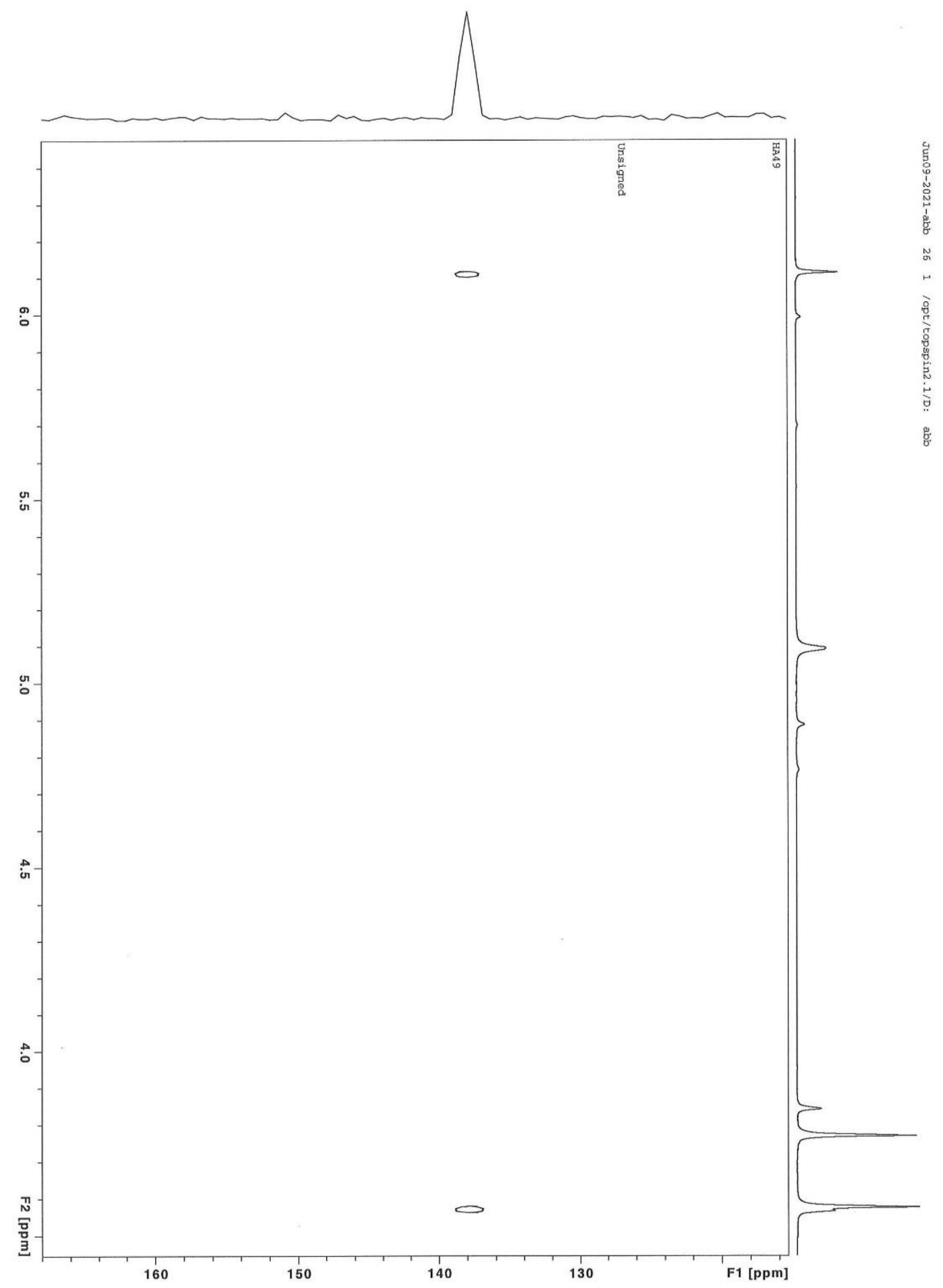

Figure S75. Detail of ^{1}H-^{15}N HMBC spectrum of compound **7c.**

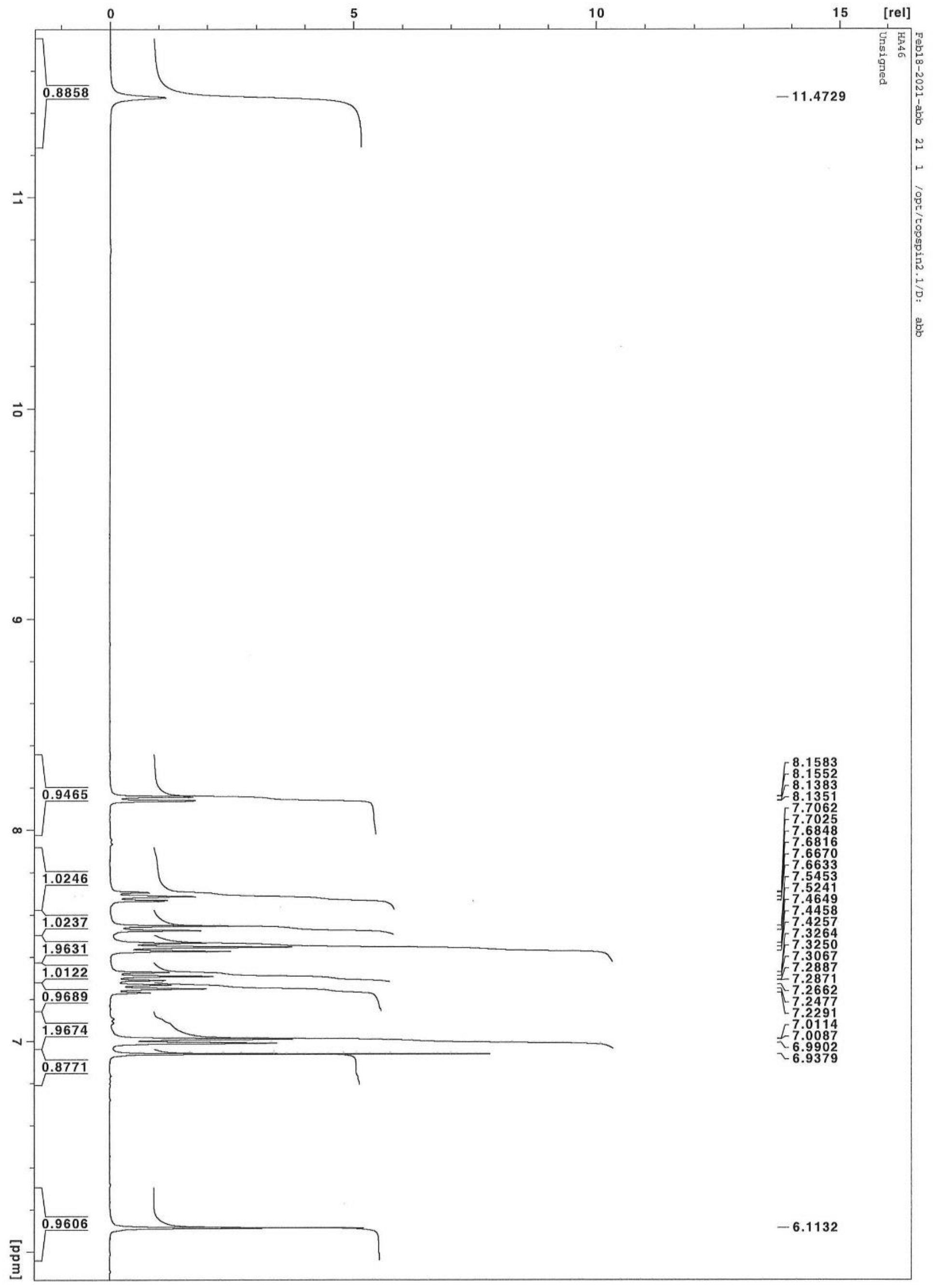

Figure S76. ^{1}H spectrum of compound **7d** (400 MHz, DMSO-d_6).

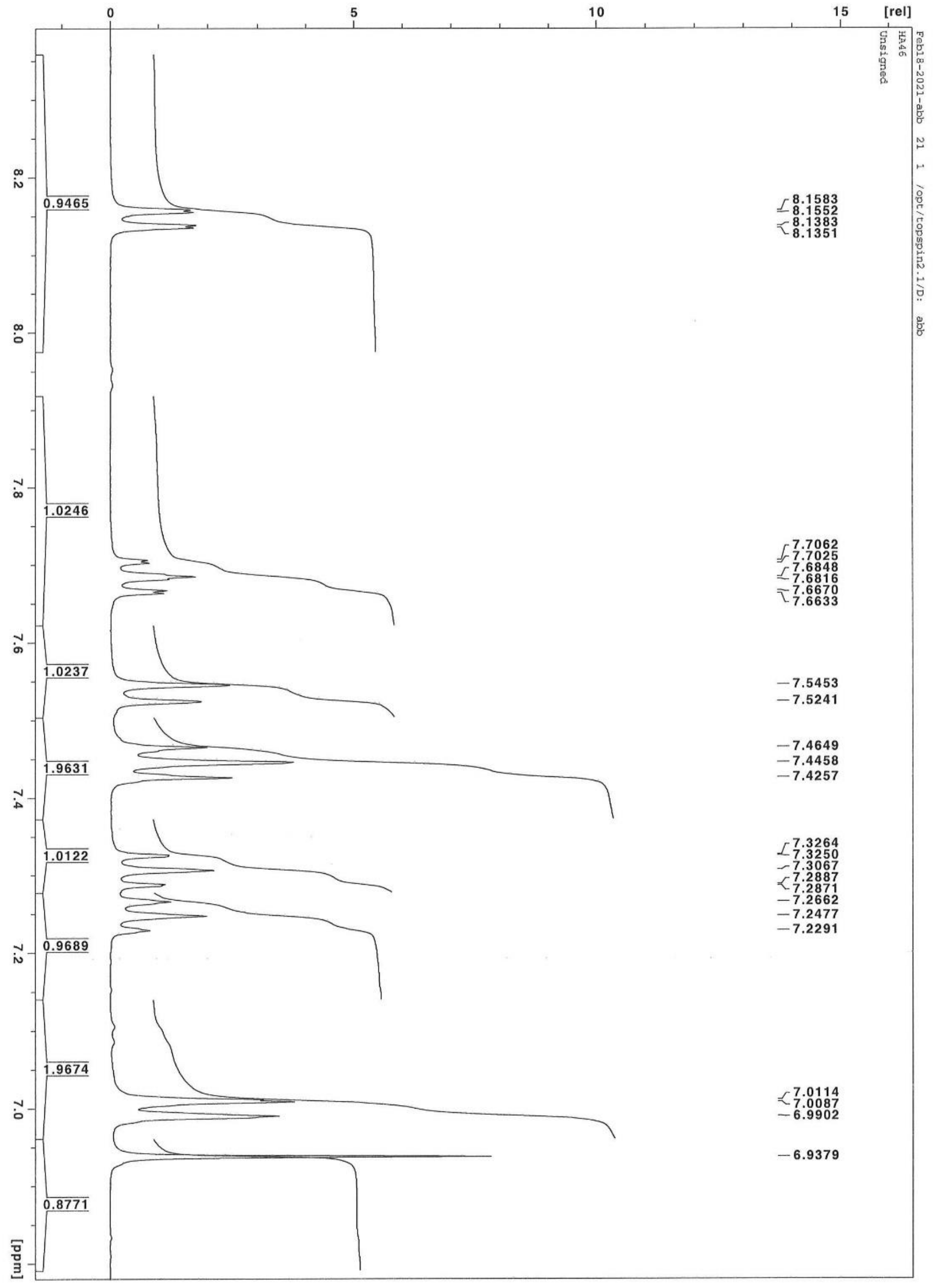

Figure S77. Detail of ^{1}H spectrum of compound **7d.**

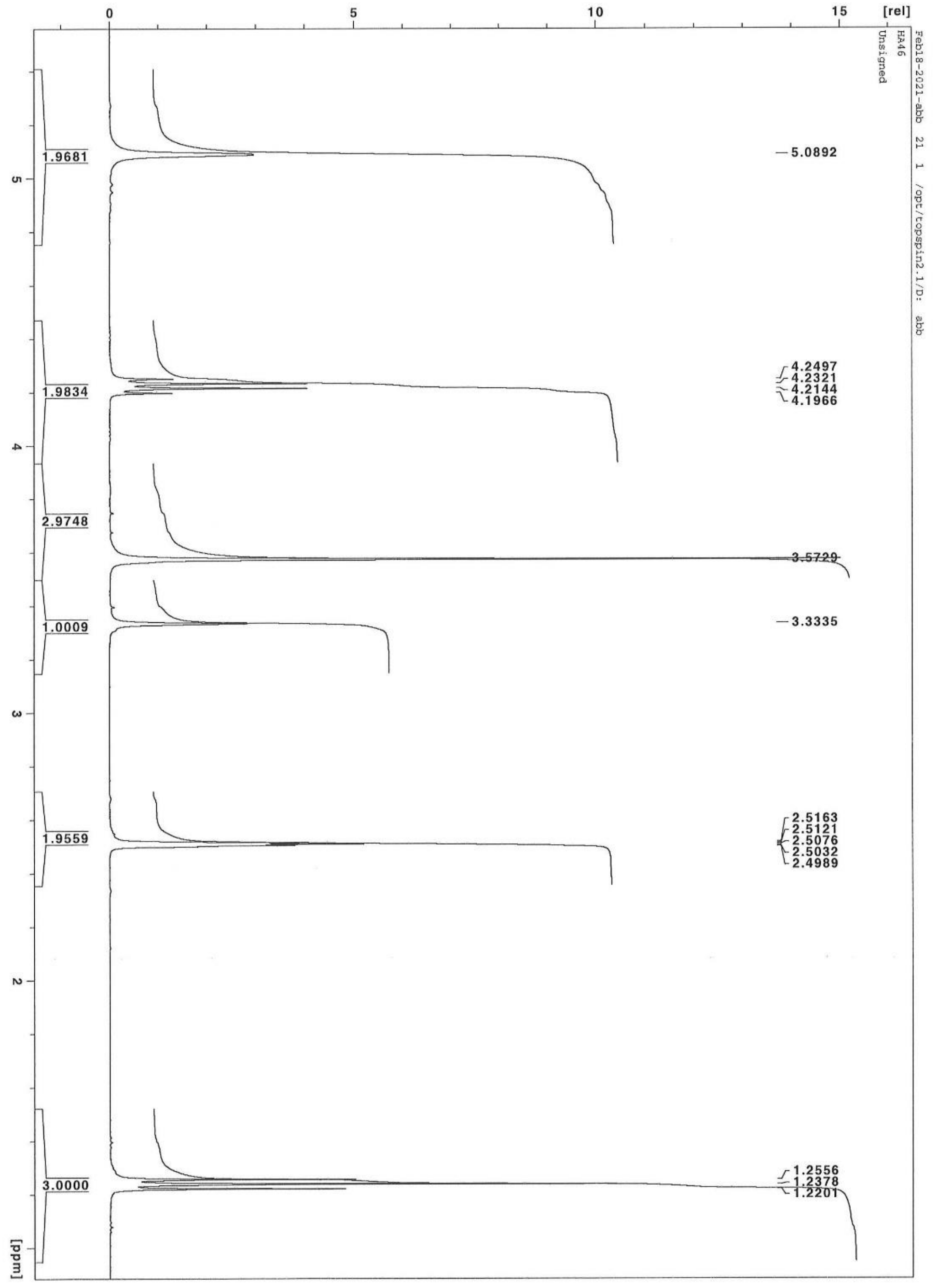

Figure S78. Detail of ^{1}H spectrum of compound **7d.**

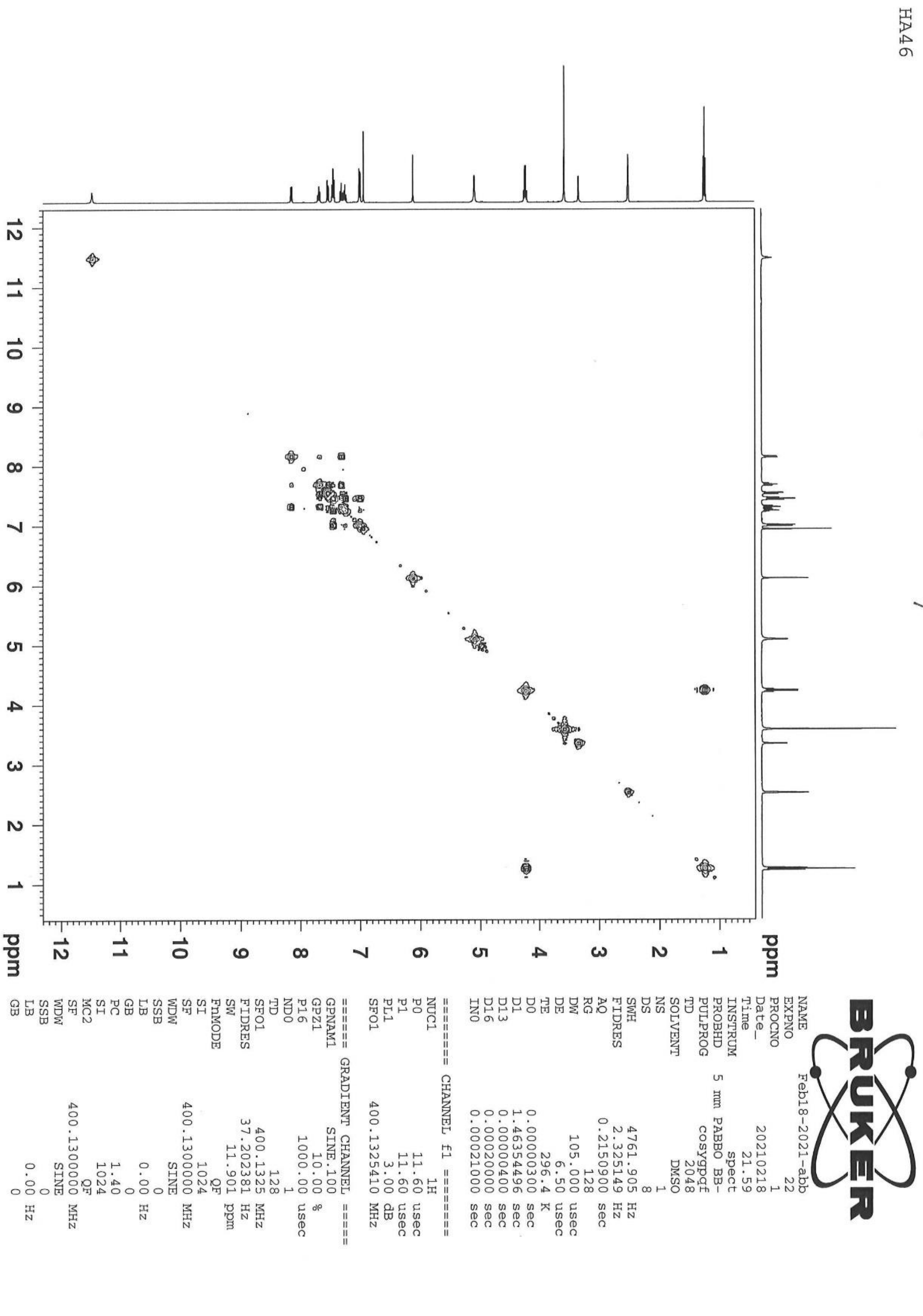

Figure S79. ^{1}H-^{1}H COSY spectrum of compound **7d.**

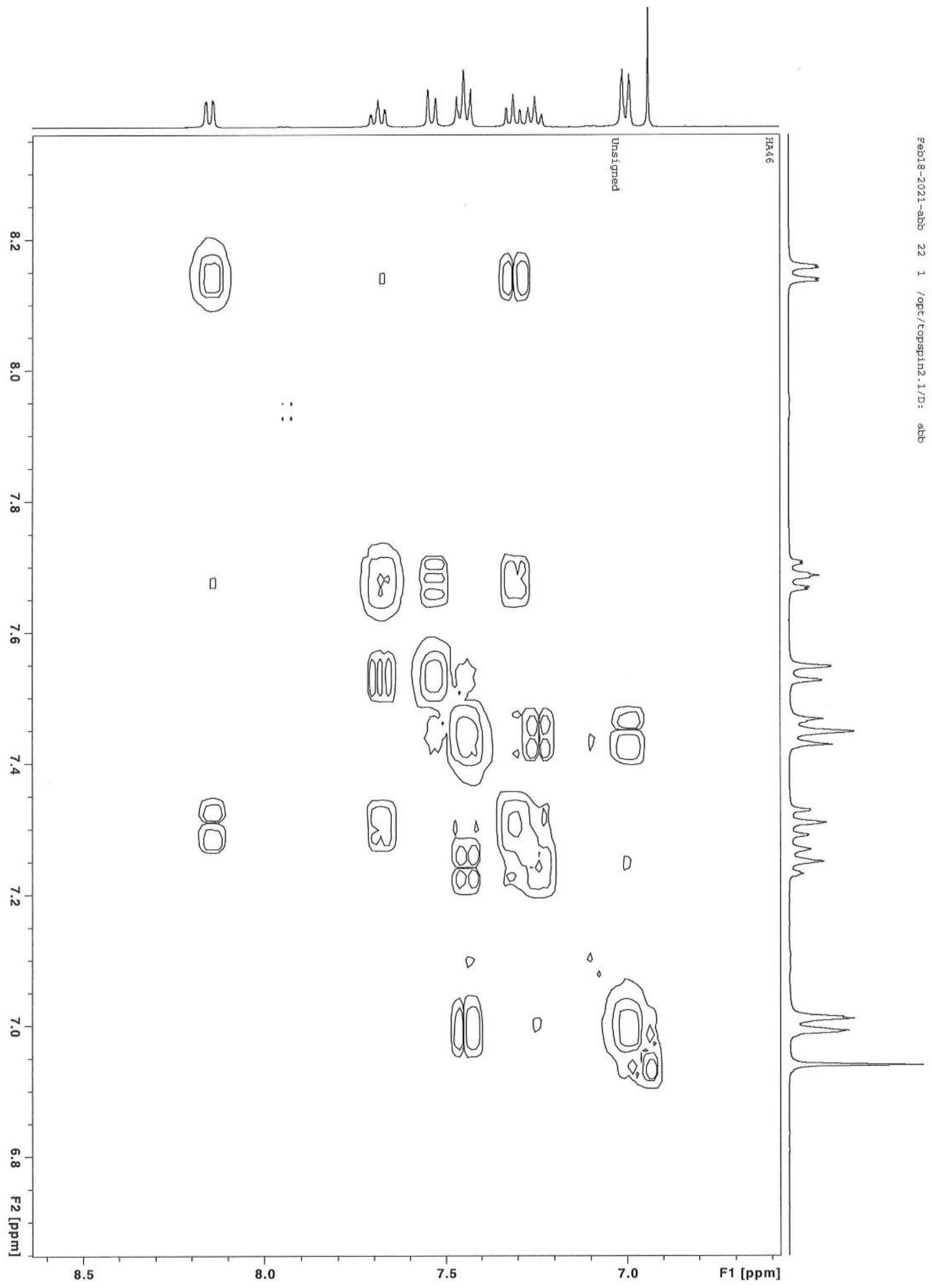

Figure S80. Detail of ^{1}H-^{1}H COSY spectrum of compound **7d.**

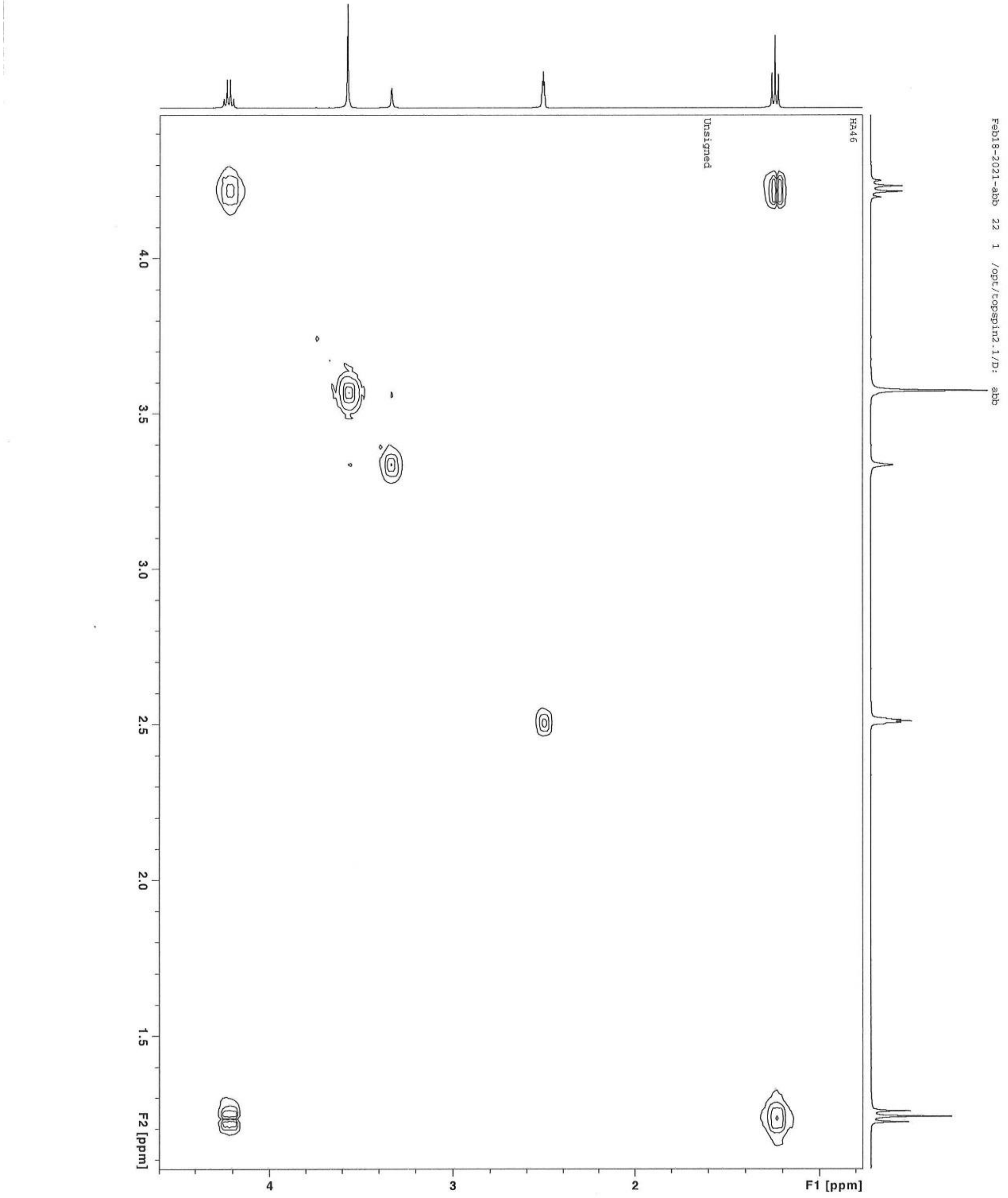

Figure S81. Detail of ^{1}H-^{1}H COSY spectrum of compound **7d.**

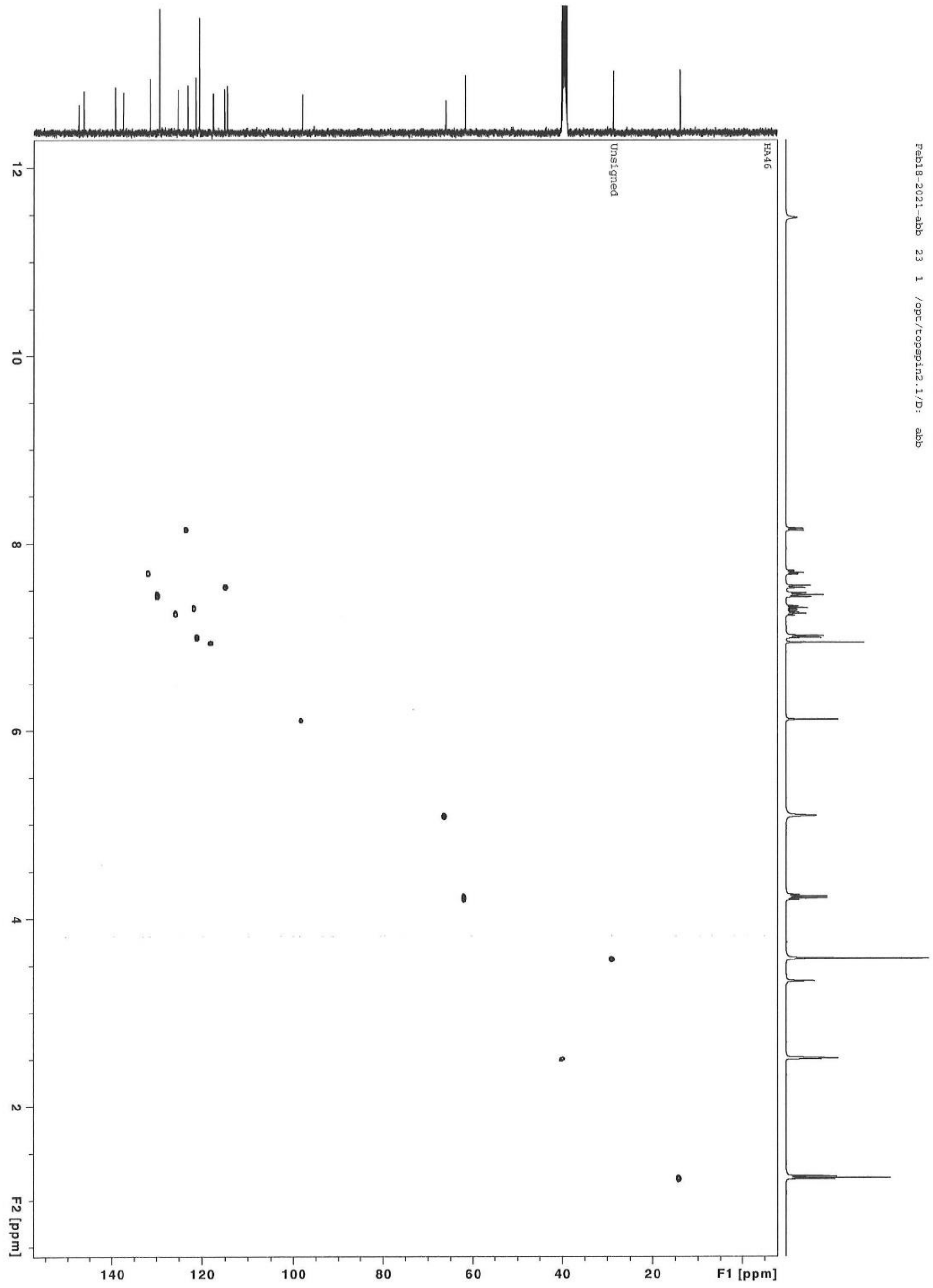

Figure S82. ^{1}H-^{13}C HSQC spectrum of compound **7d.**

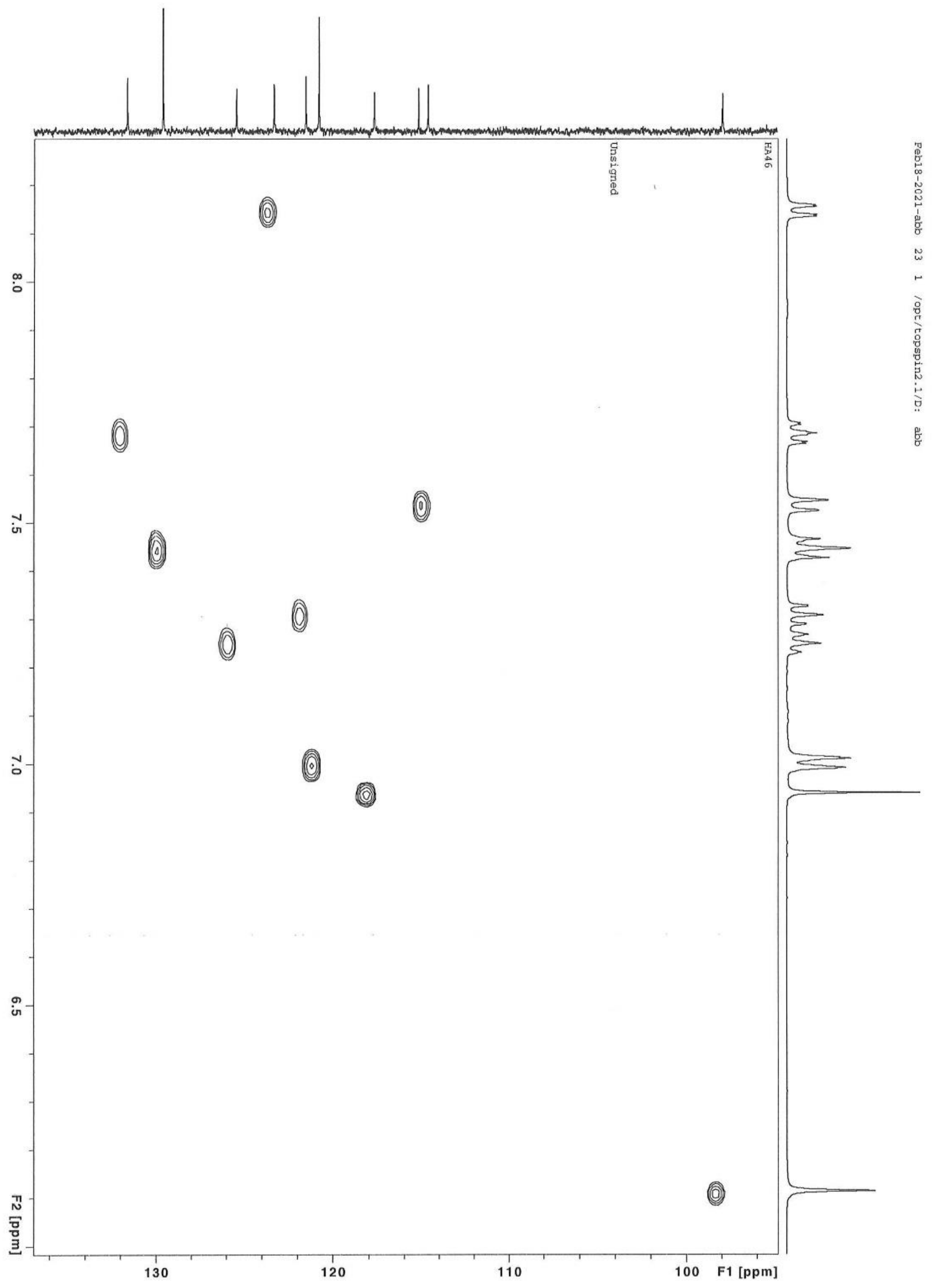

Figure S83. Detail of ^{1}H-^{13}C HSQC spectrum of compound **7d.**

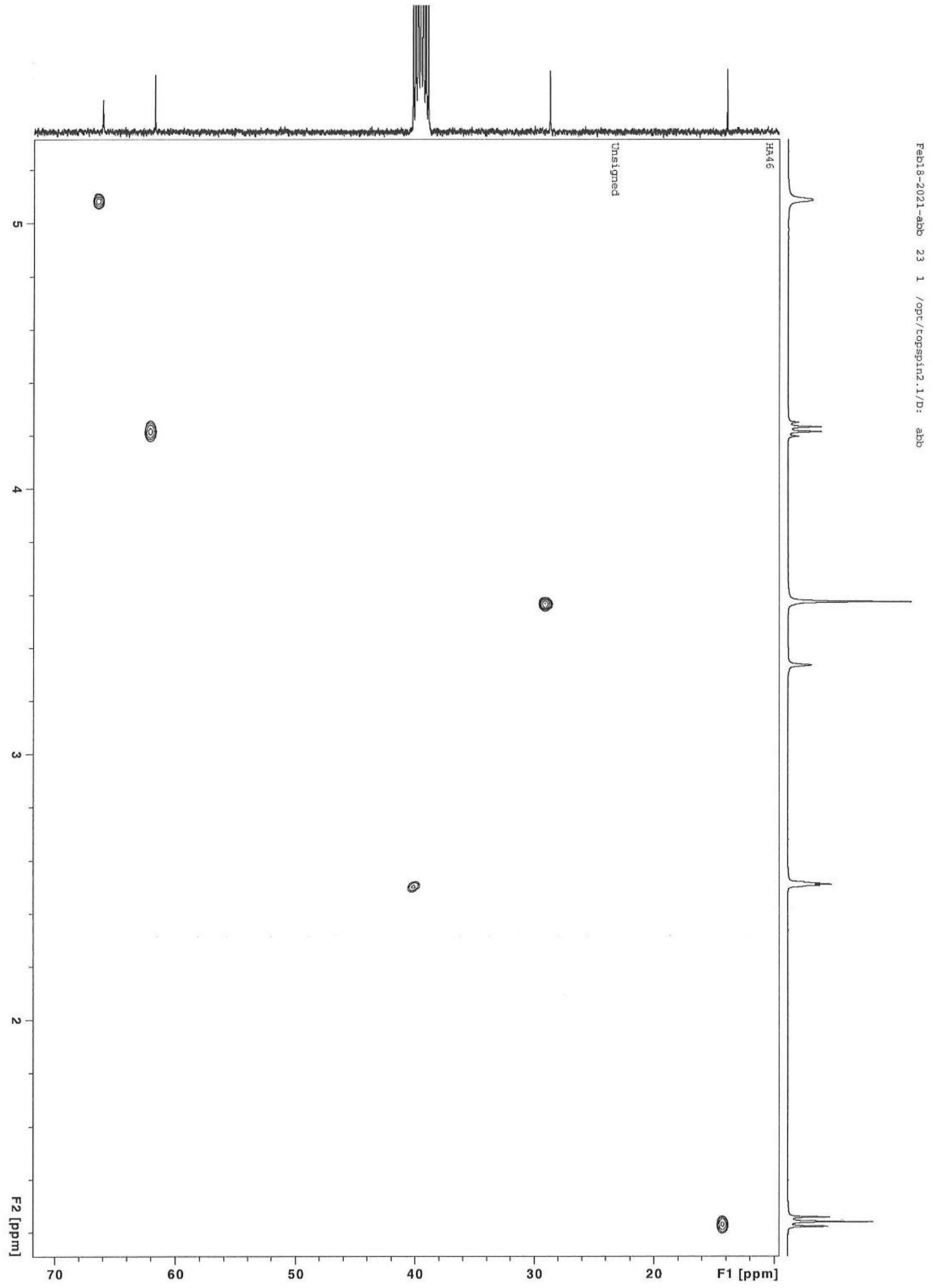

Figure S84. Detail of ^{1}H-^{13}C HSQC spectrum of compound **7d.**

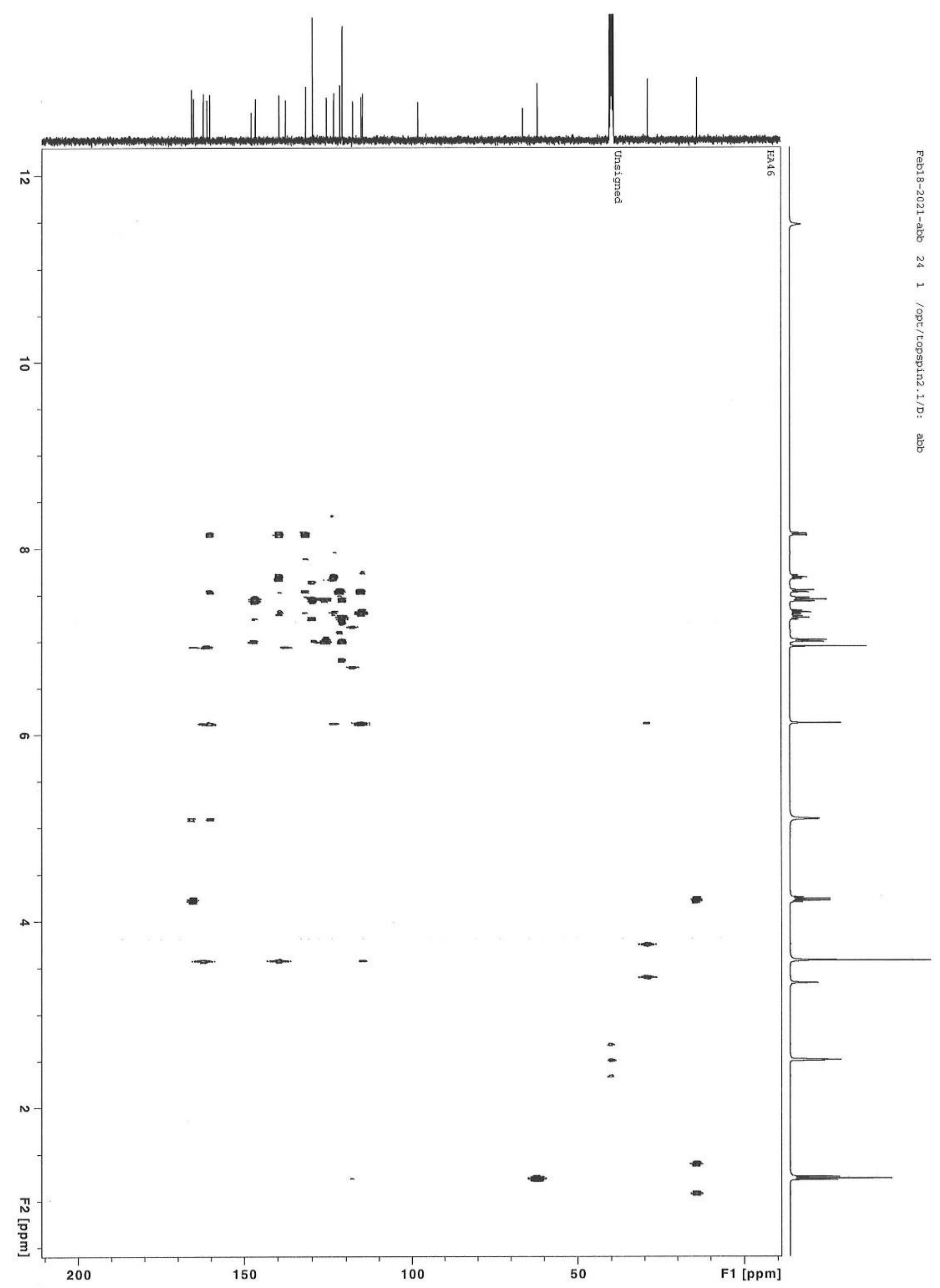

Figure S85. ^{1}H-^{13}C HMBC spectrum of compound **7d.**

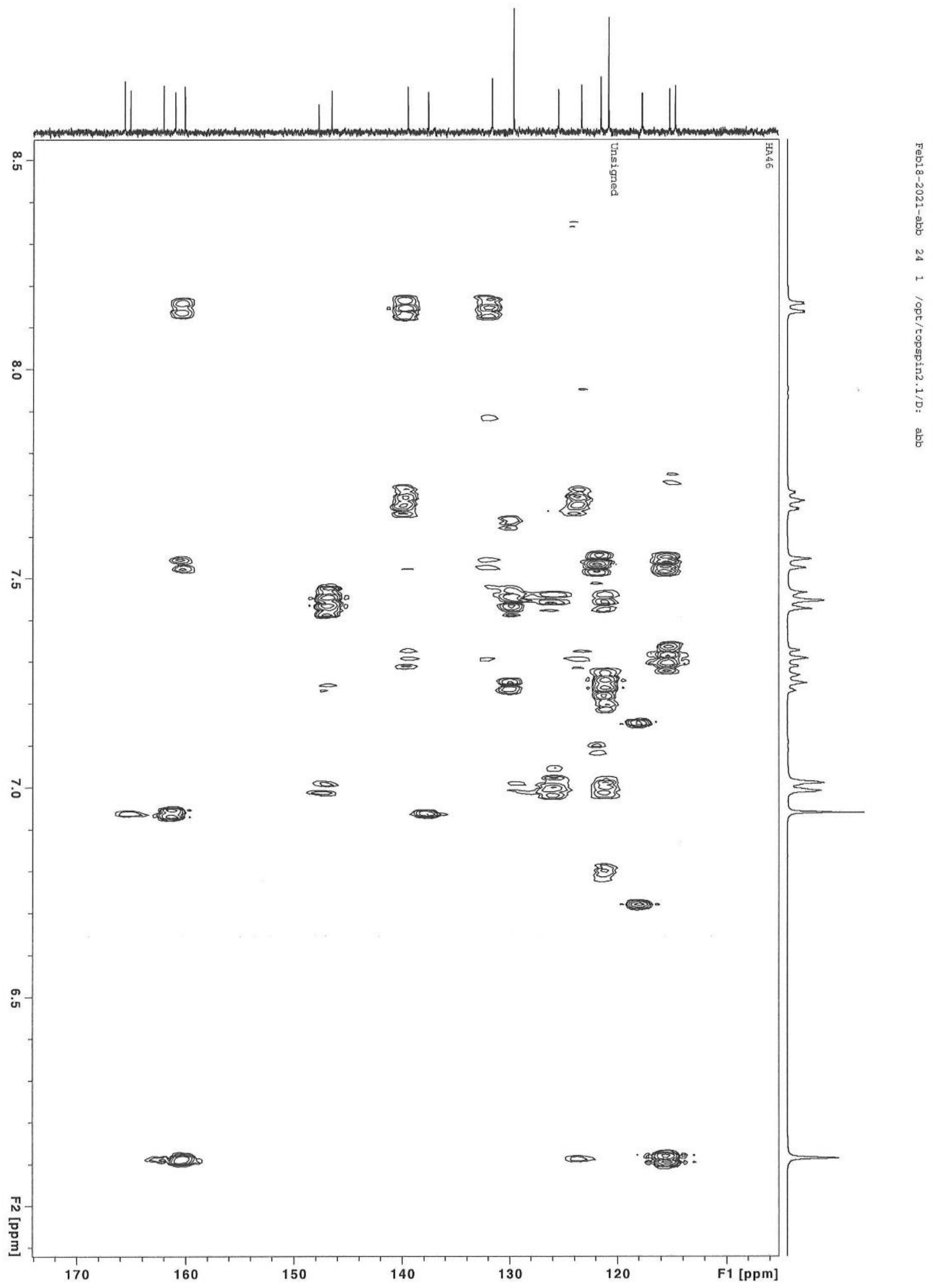

Figure S86. Detail of ^{1}H-^{13}C HMBC spectrum of compound **7d.**

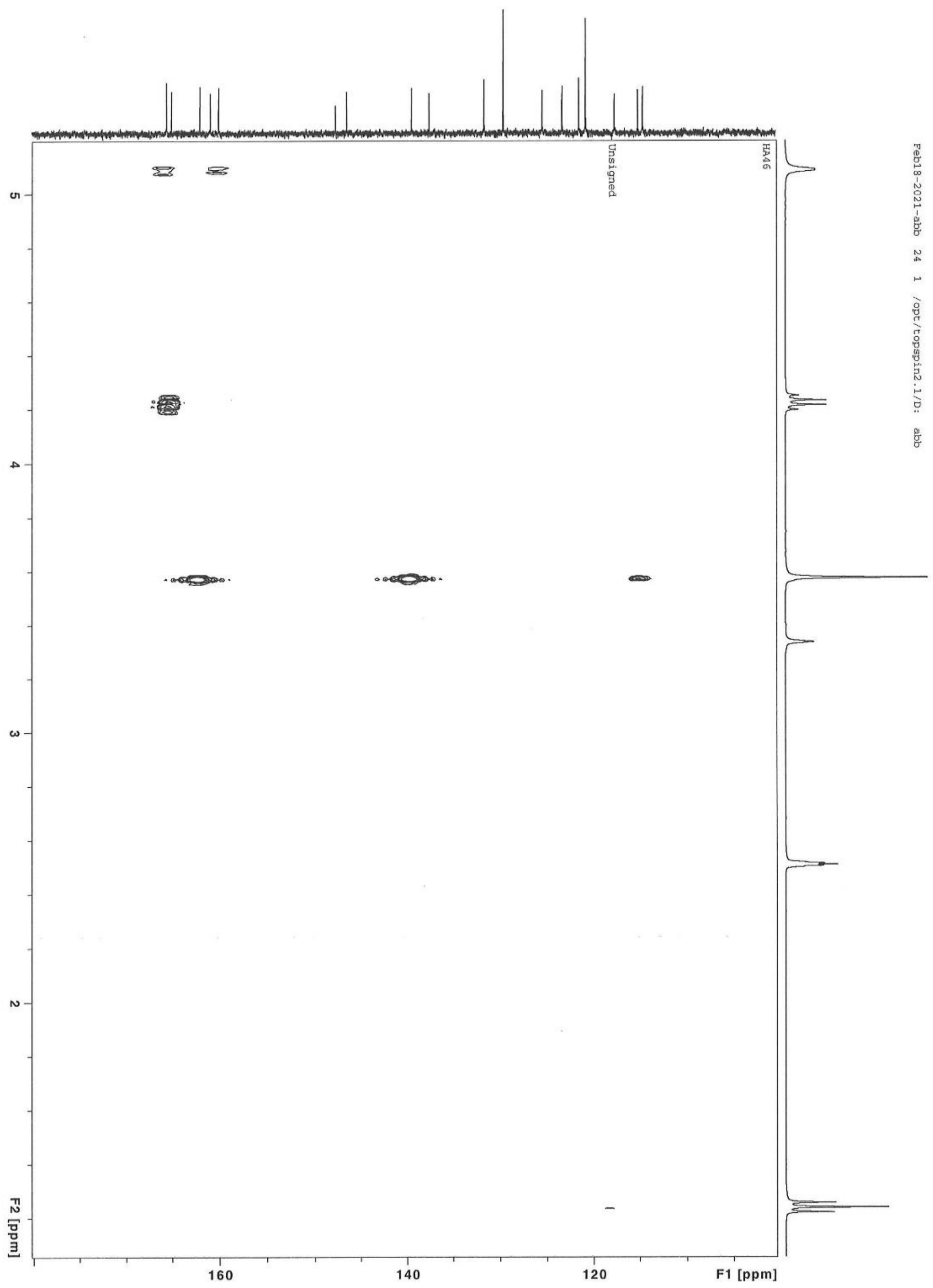

Figure S87. Detail of ^{1}H-^{13}C HMBC spectrum of compound **7d.**

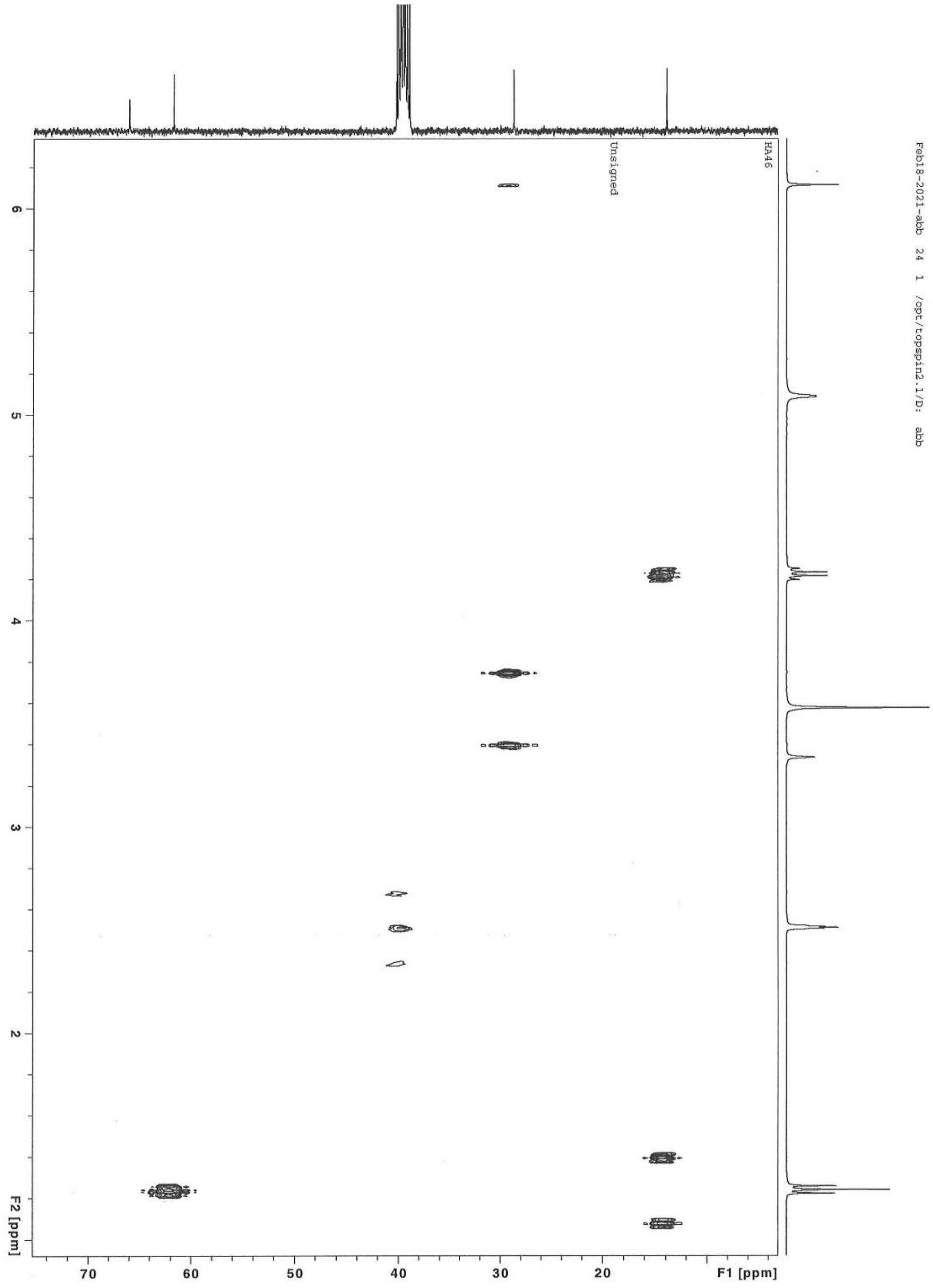

Figure S88. Detail of ^{1}H-^{13}C HMBC spectrum of compound **7d.**

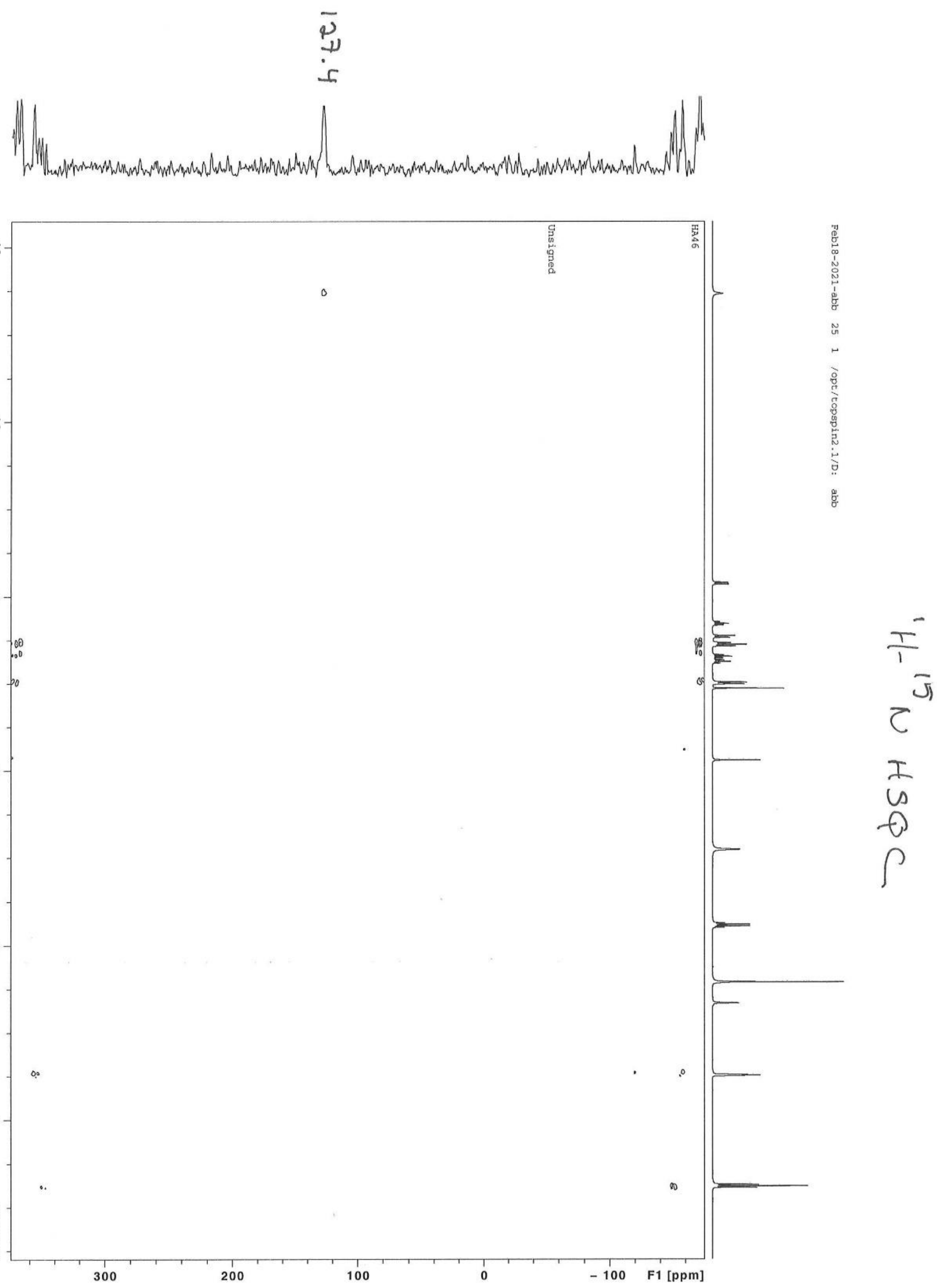

Figure S89. ^{1}H-^{15}N HSQC spectrum of compound **7d.**

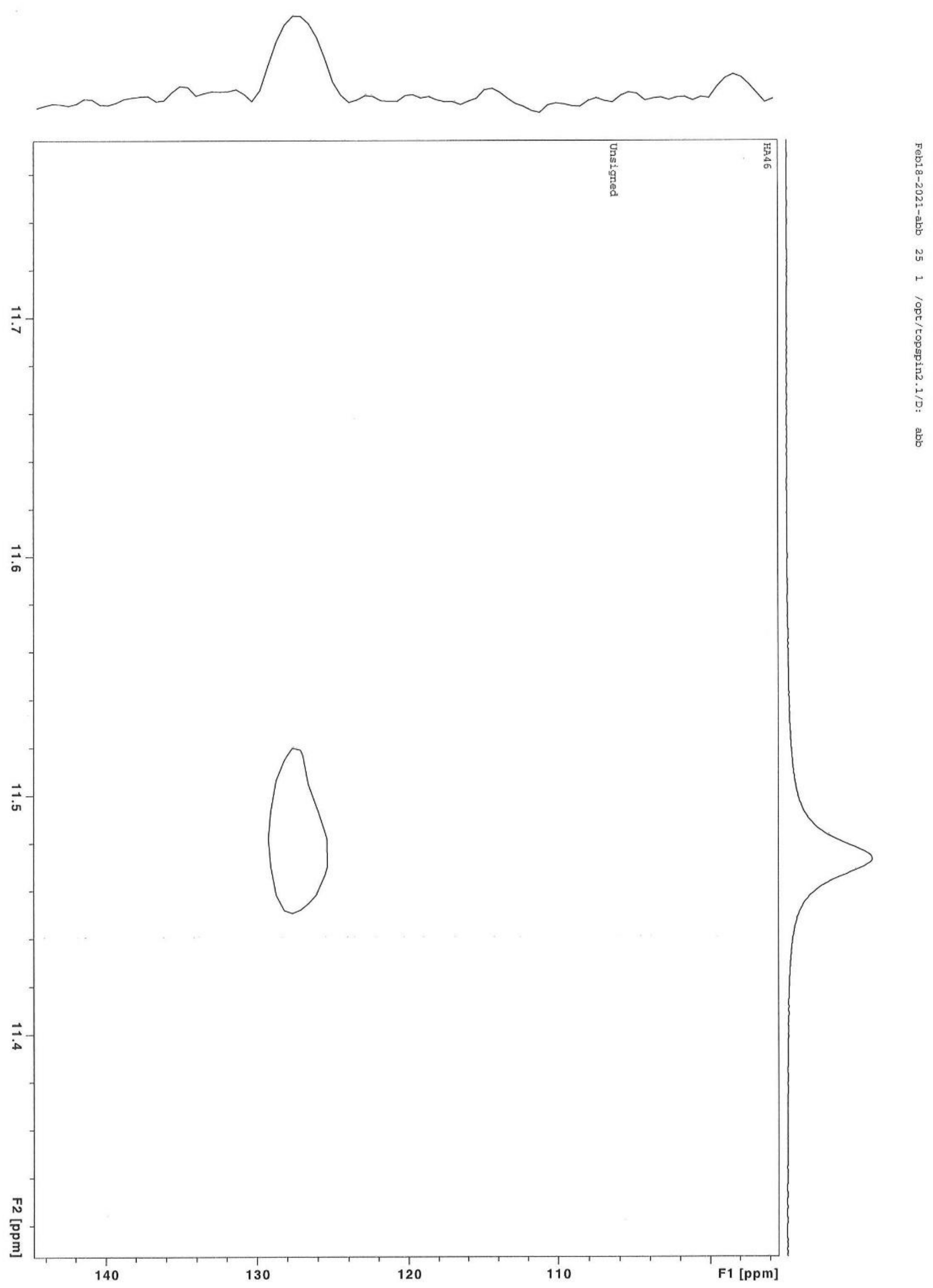

Figure S90. Detail of ^{1}H-^{15}N HSQC spectrum of compound **7d.**

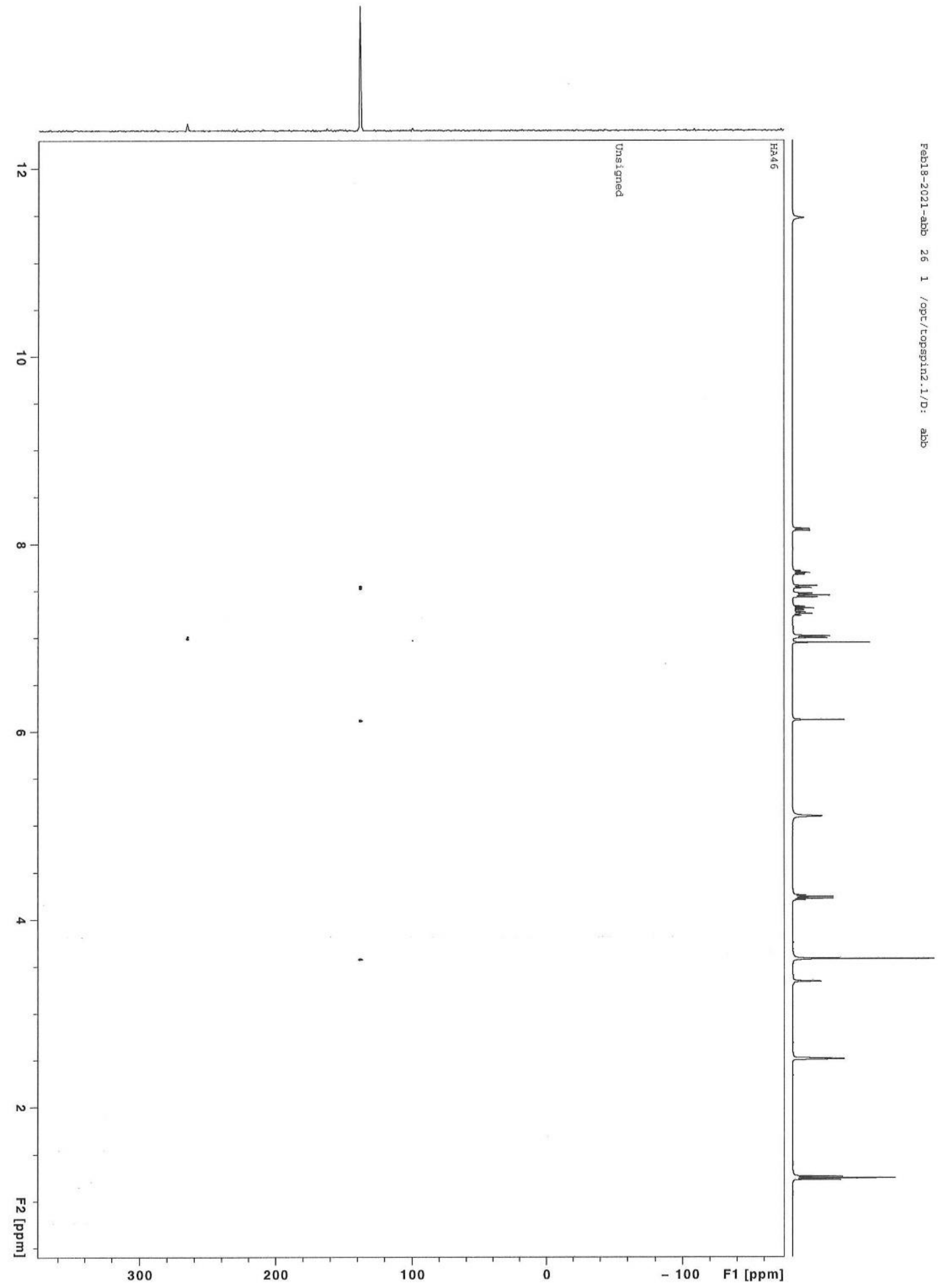

Figure S91. ^{1}H-^{15}N HMBC spectrum of compound **7d.**

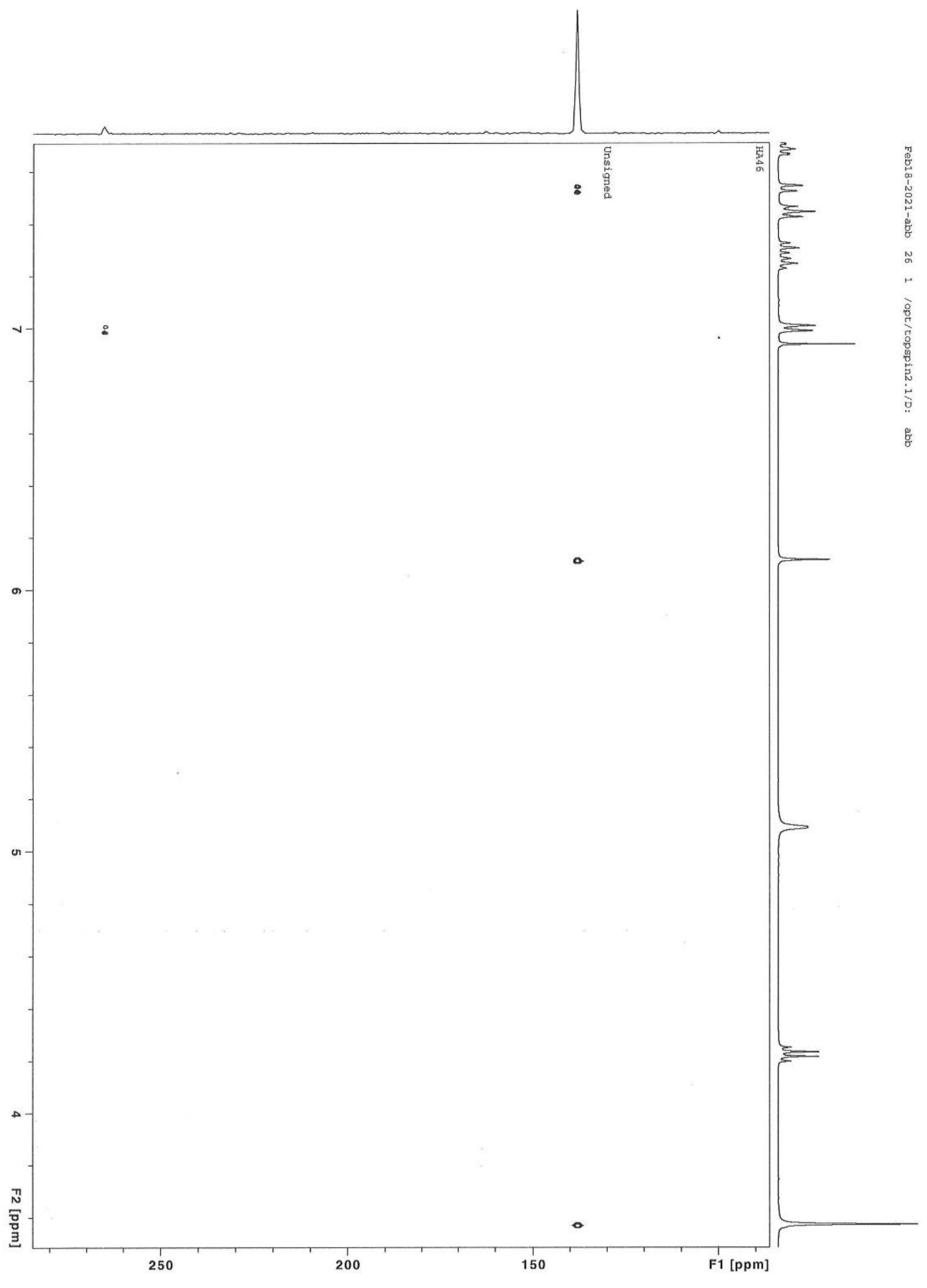

Figure S92. Detail of ^{1}H-^{15}N HMBC spectrum of compound **7d.**

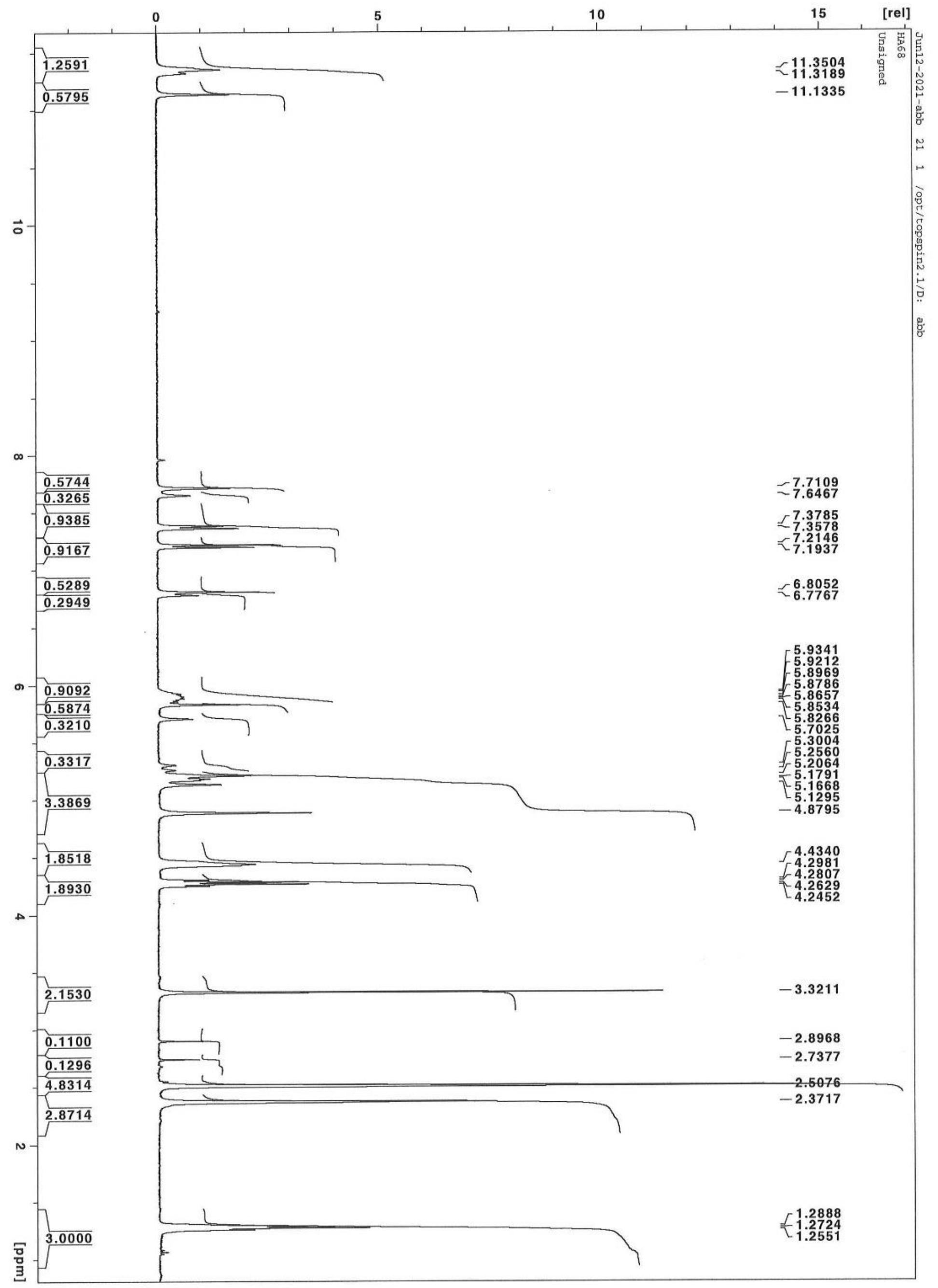

Figure S93. ^{1}H spectrum of compound **7e** (400 MHz, DMSO-d_6). Solubility was low, and sample contains a small amount of DMF.

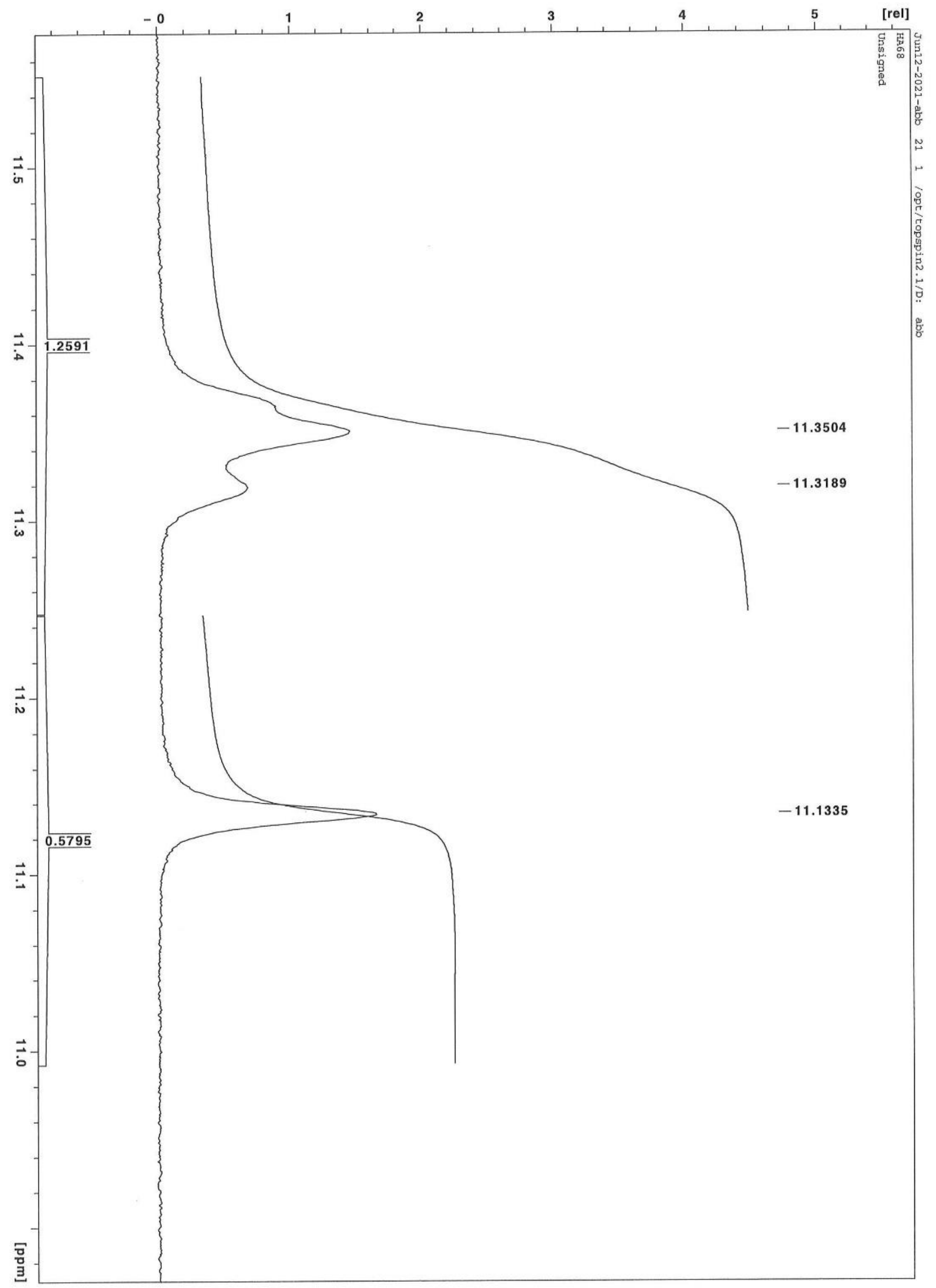

Figure S94. Detail of ^{1}H spectrum of compound **7e.**

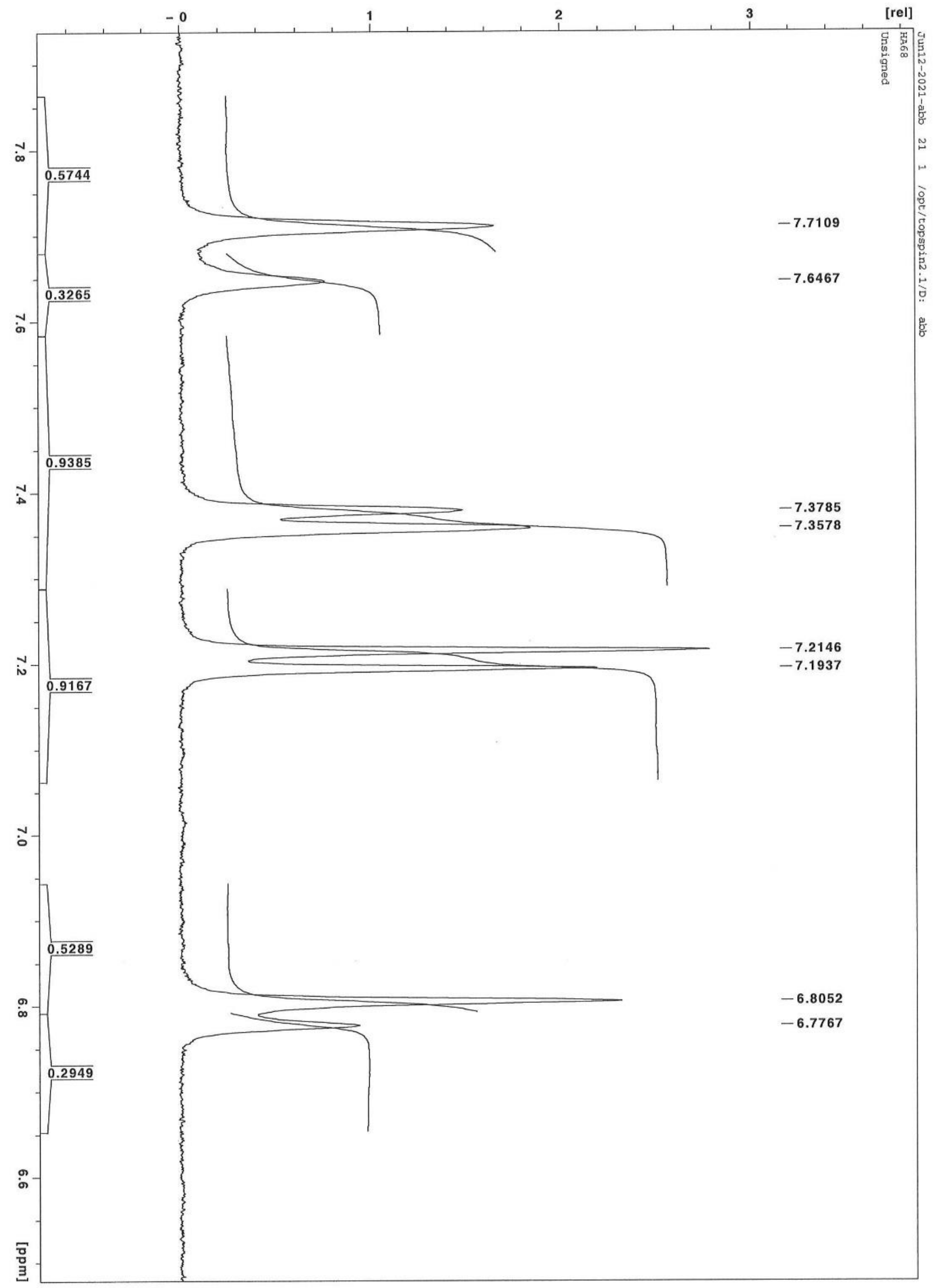

Figure S95. Detail of ^{1}H spectrum of compound **7e.**

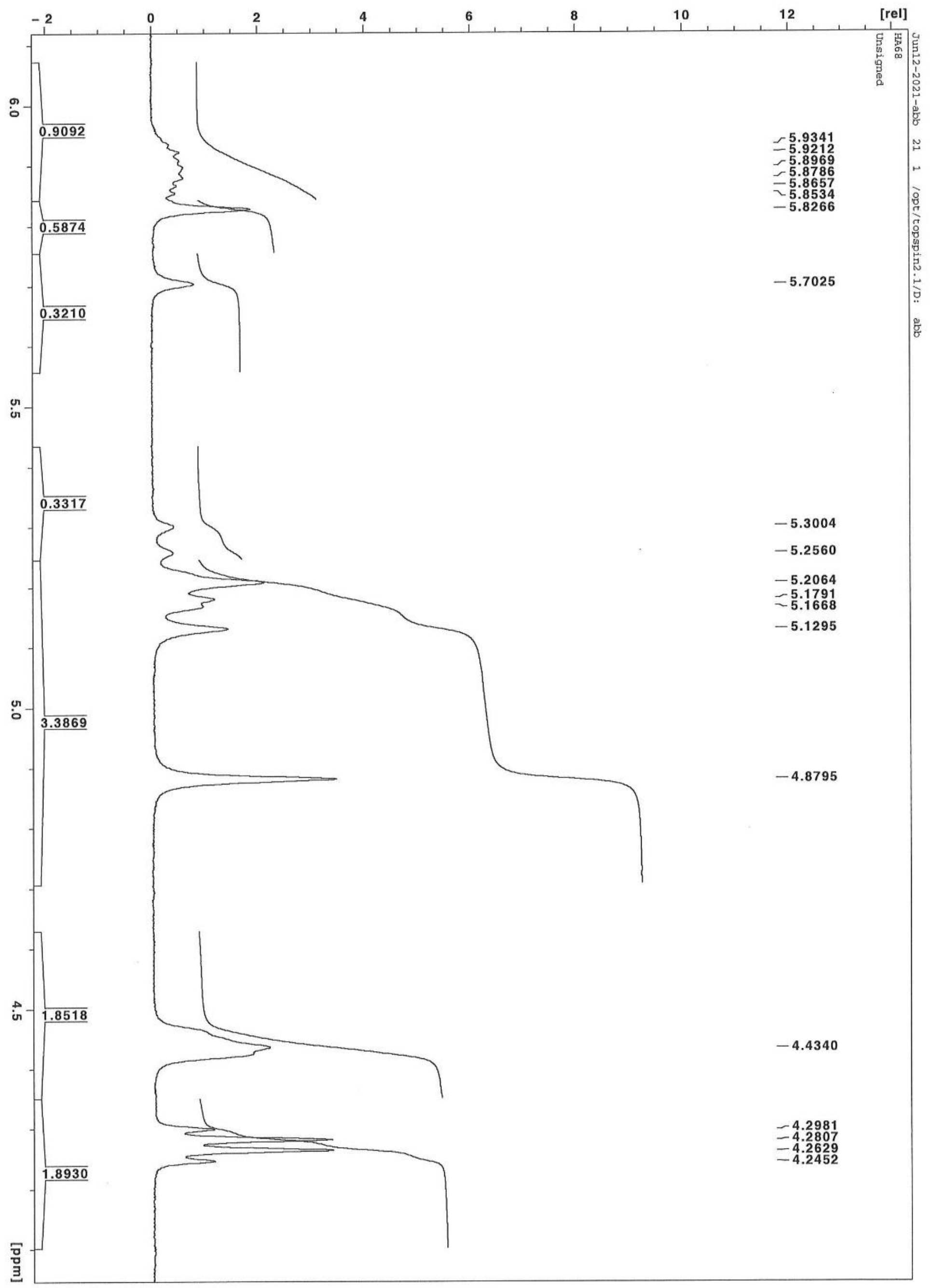

Figure S96. Detail of ^{1}H spectrum of compound **7e.**

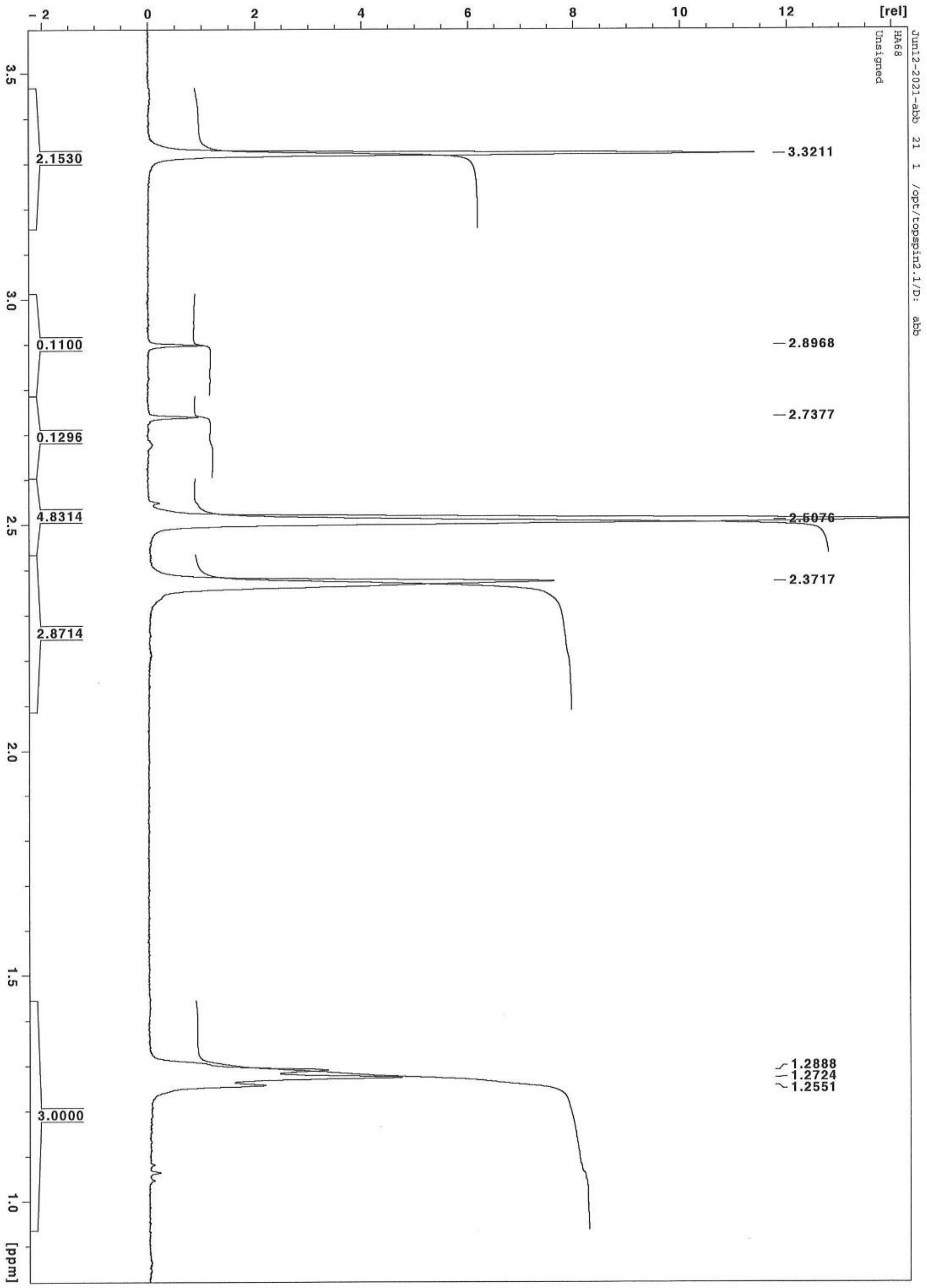

Figure S97. Detail of ^{1}H spectrum of compound **7e.**

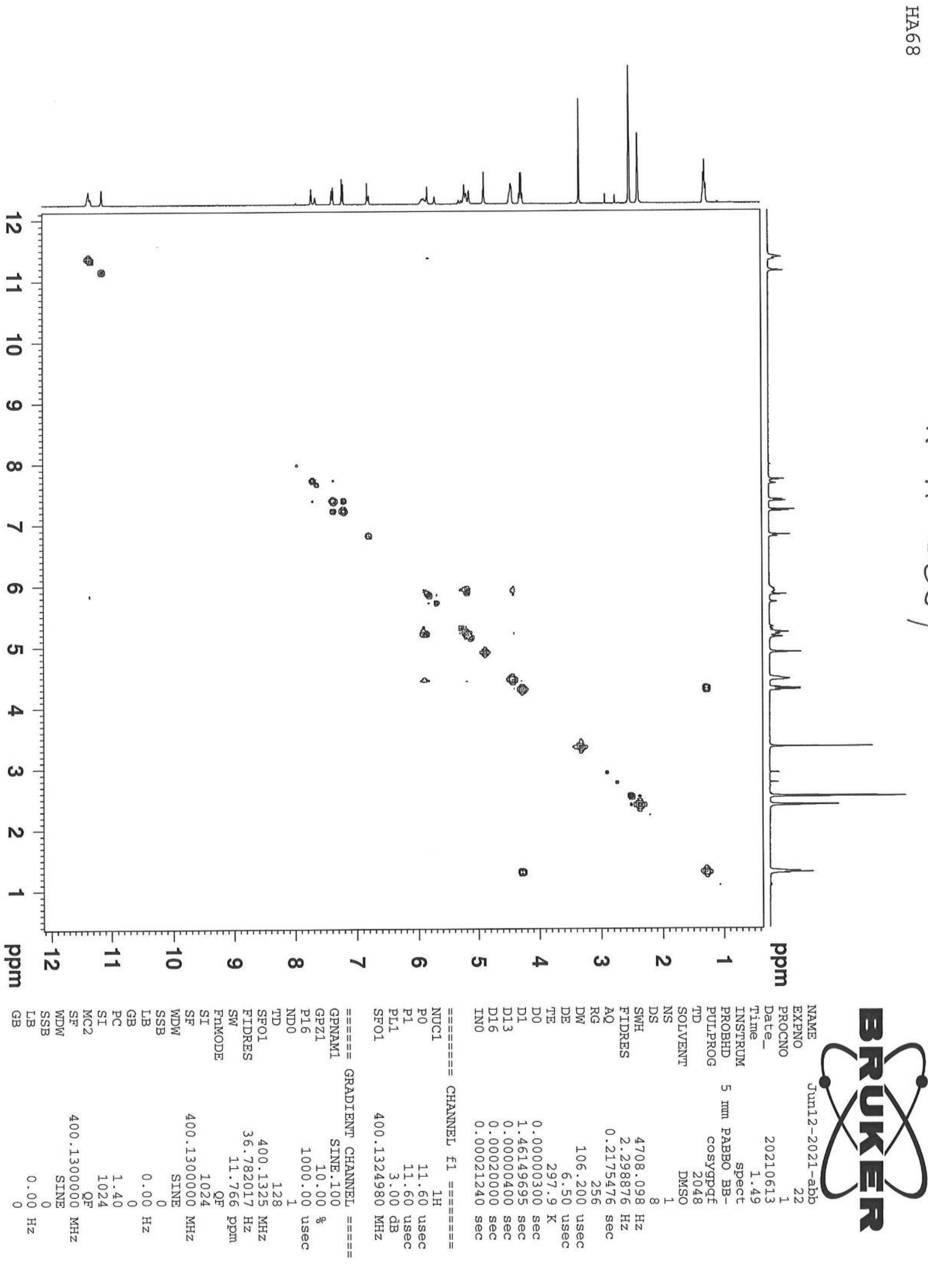

Figure S98. ^{1}H-^{1}H COSY spectrum of compound **7e.**

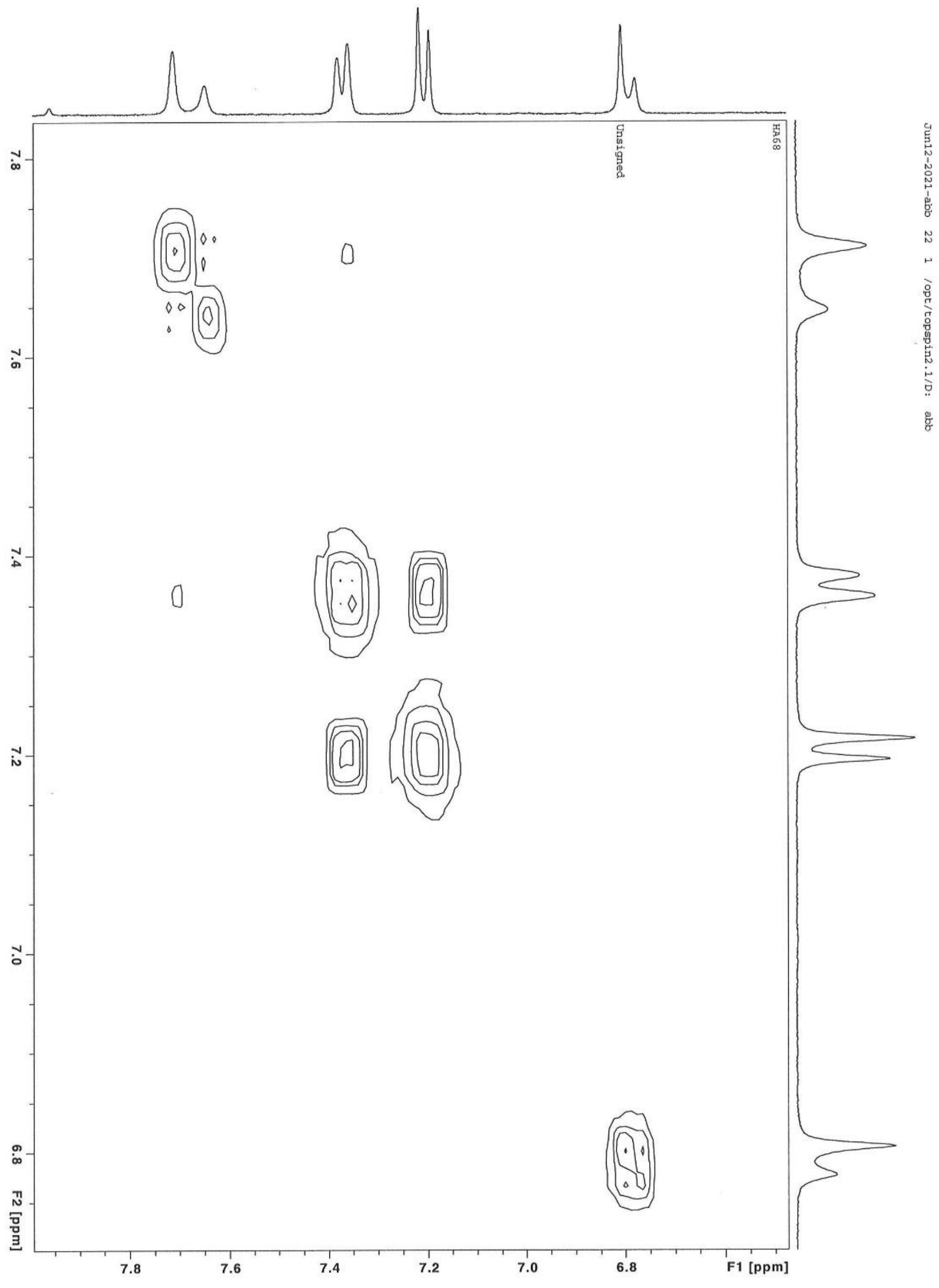

Figure S99. Detail of ^{1}H-^{1}H COSY spectrum of compound **7e.**

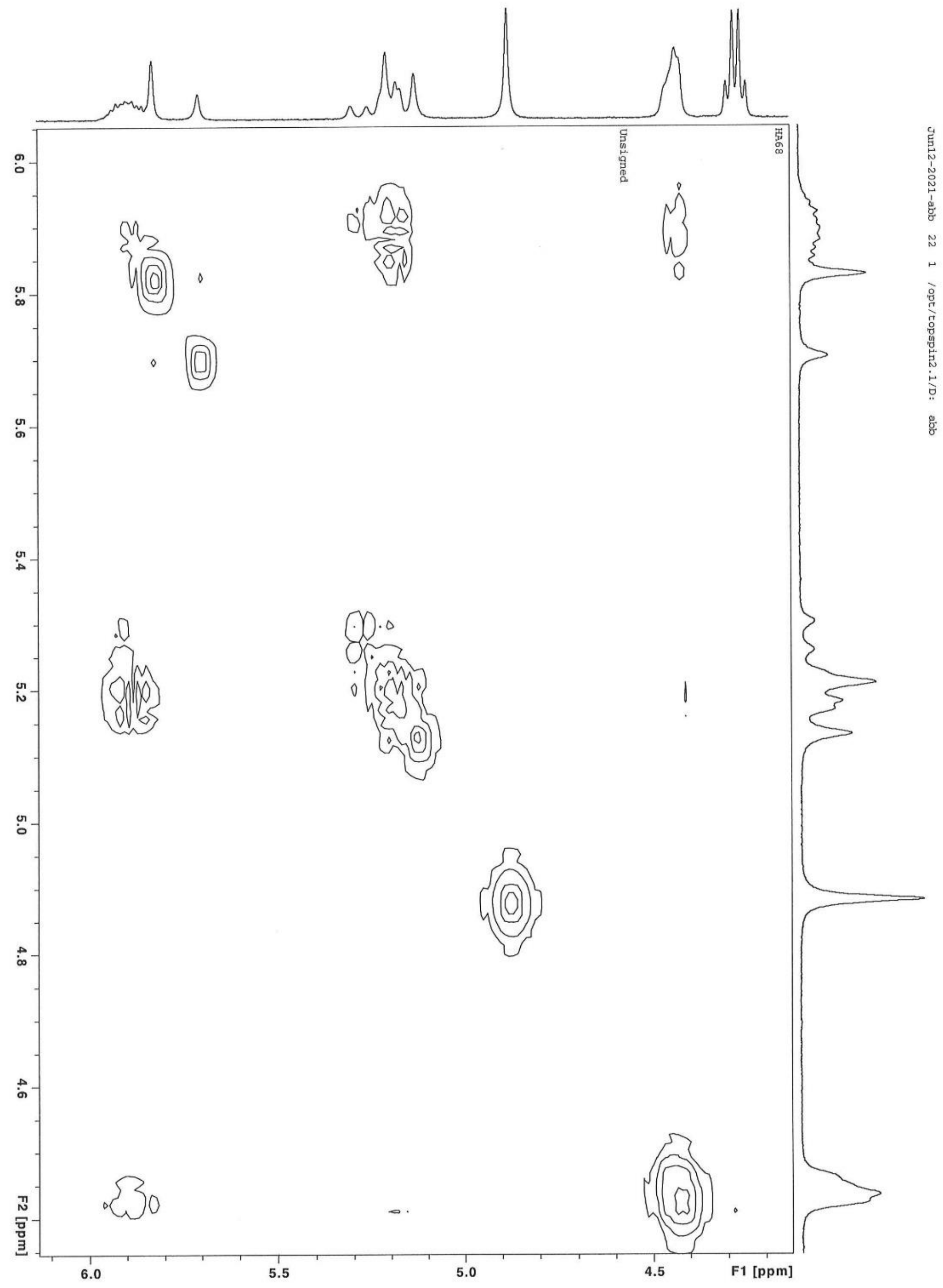

Figure S100. Detail of ^{1}H-^{1}H COSY spectrum of compound **7e.**

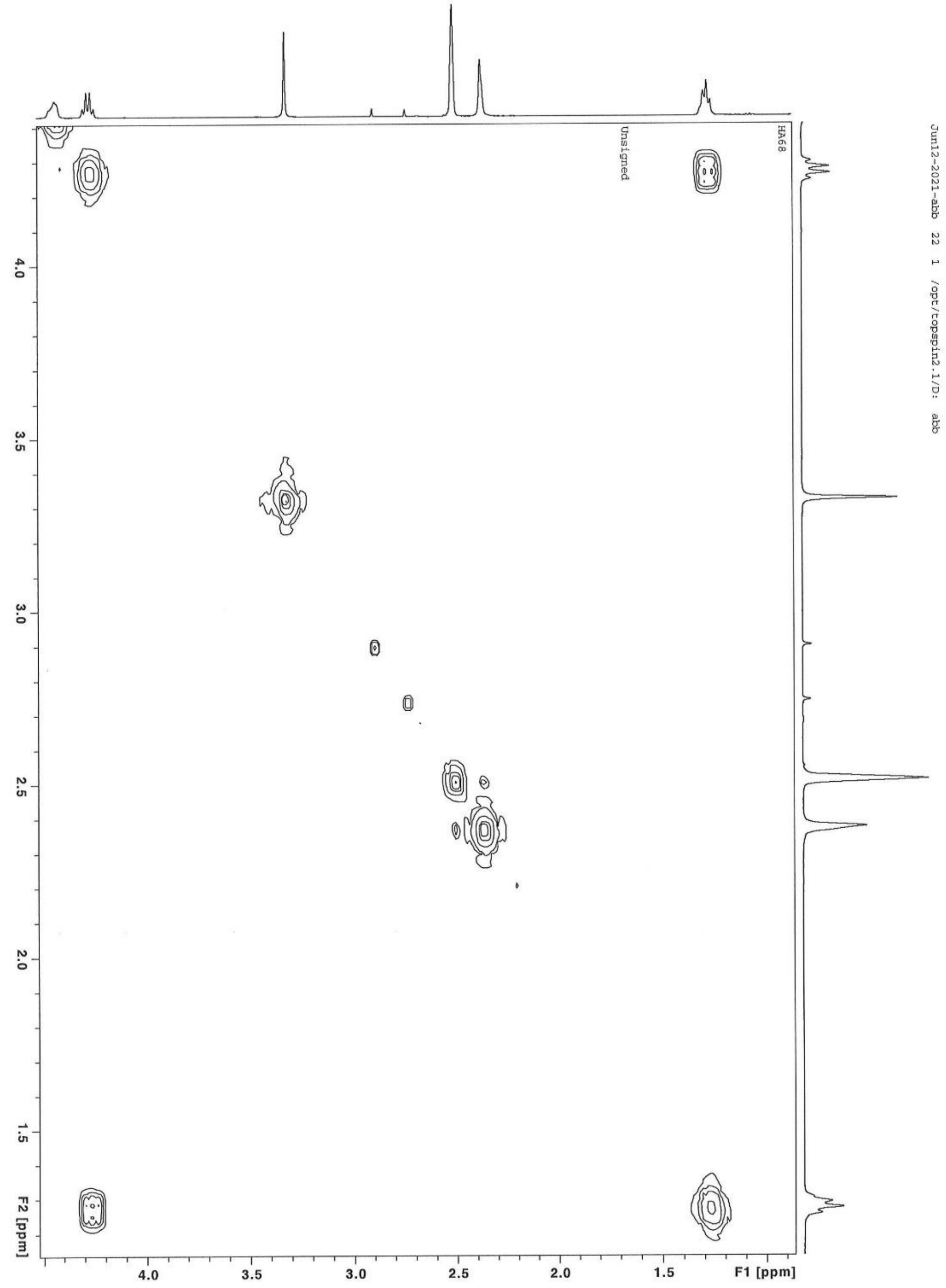

Figure S101. Detail of ^{1}H-^{1}H COSY spectrum of compound **7e.**

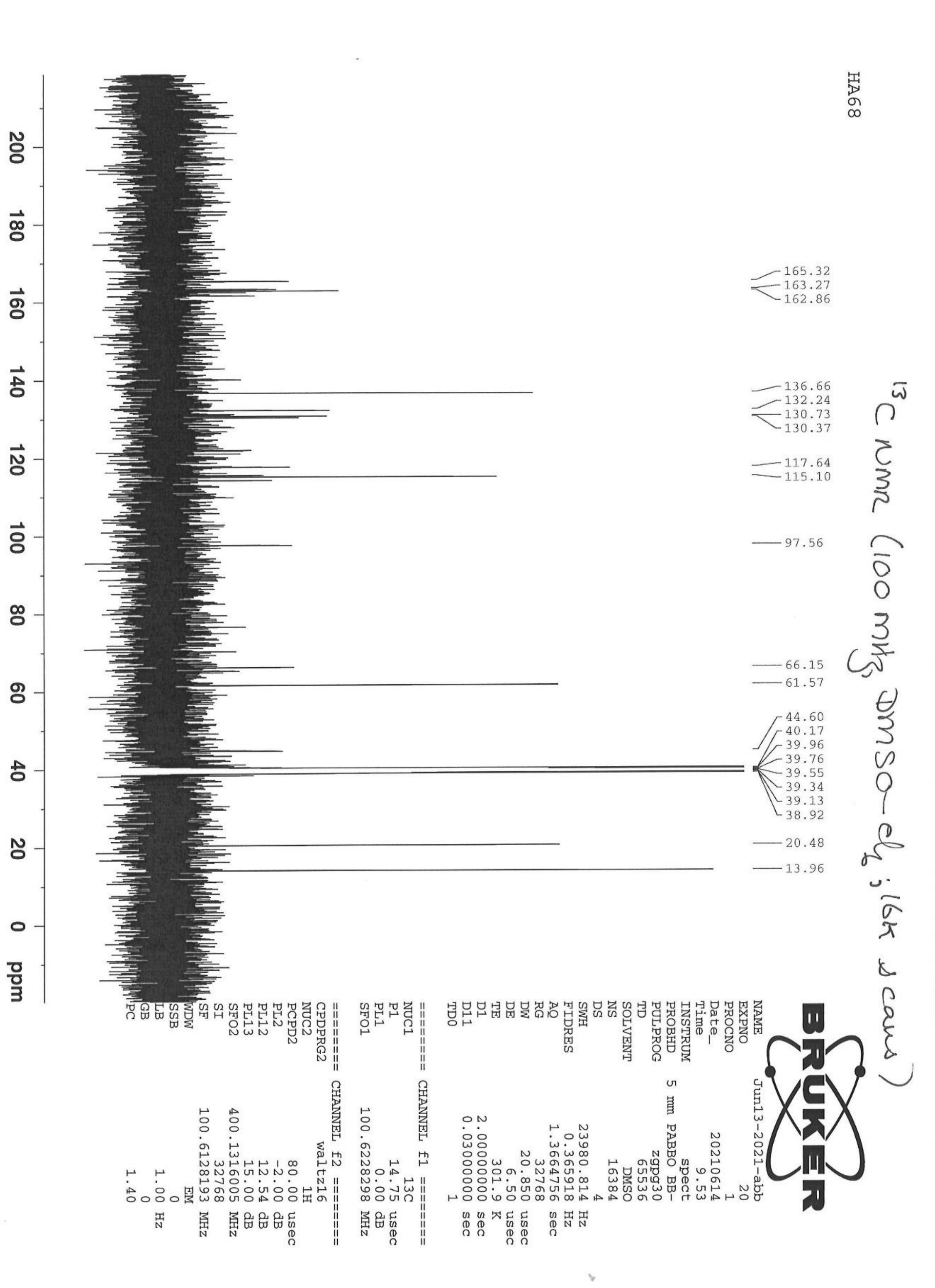

Figure S102. ^{13}C spectrum of compound **7e** (100 MHz, DMSO-d_6).

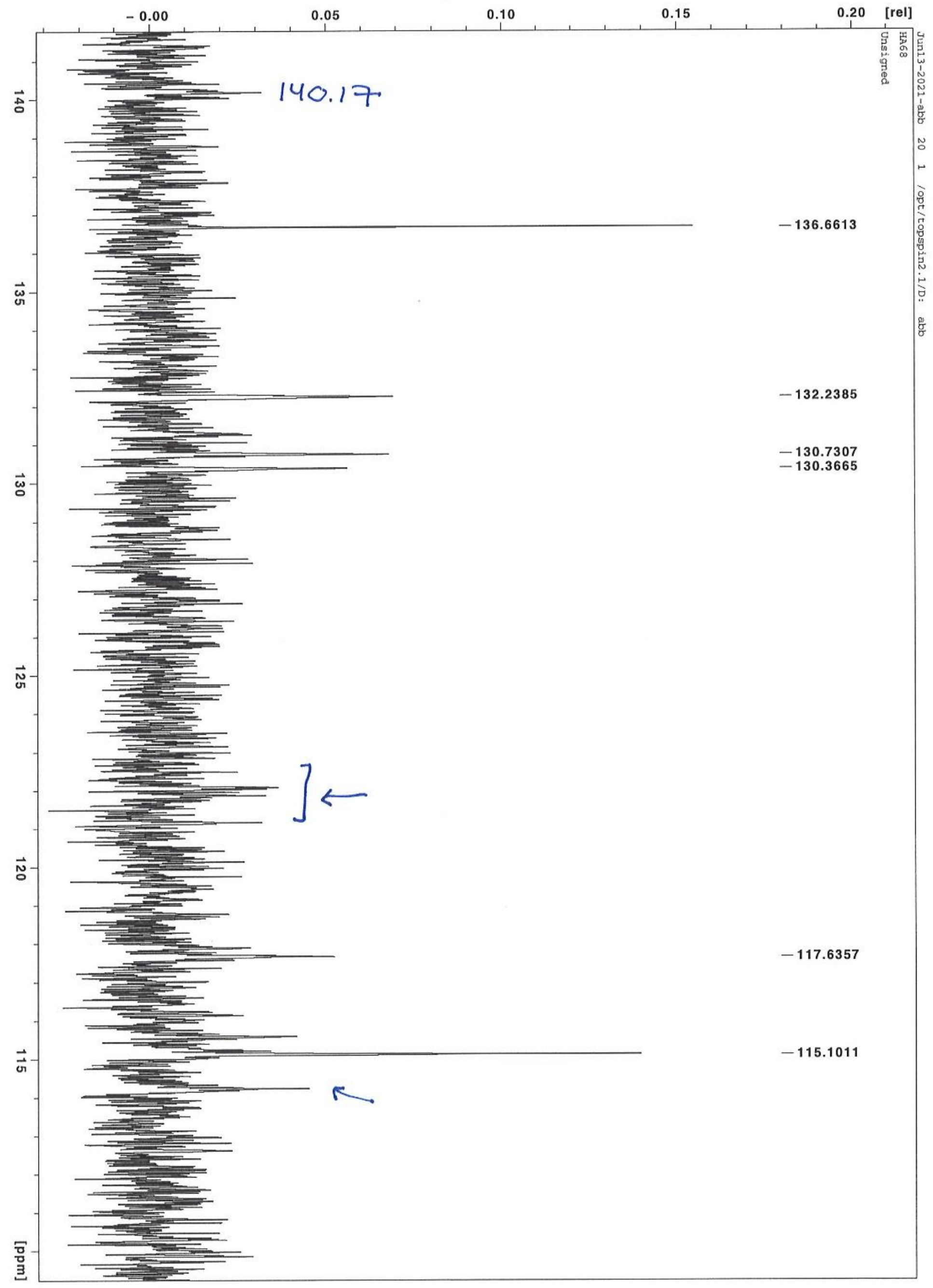

Figure S103. Detail of ^{13}C spectrum of compound **7e.**

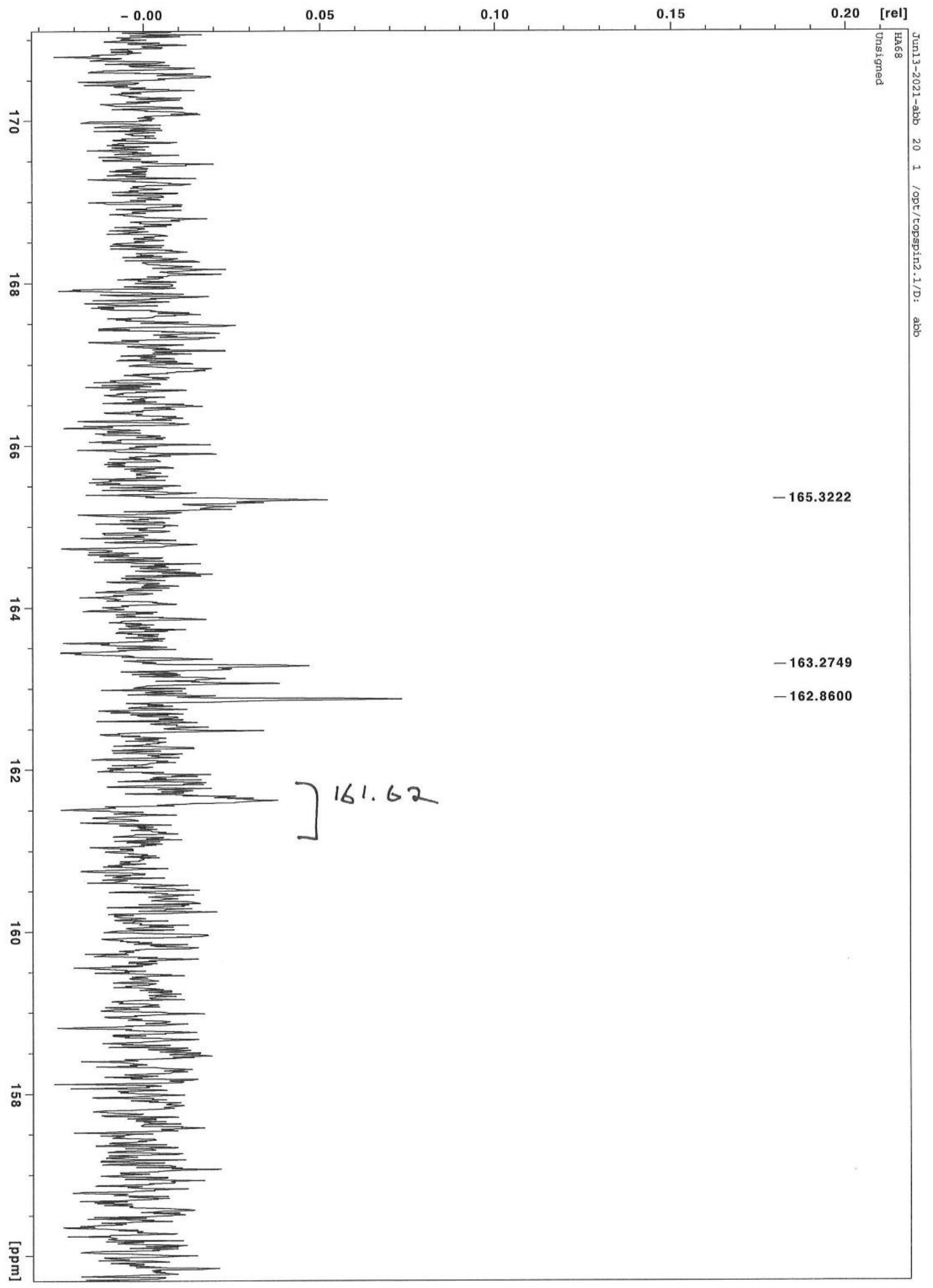

Figure S104. Detail of ^{13}C spectrum of compound **7e.**

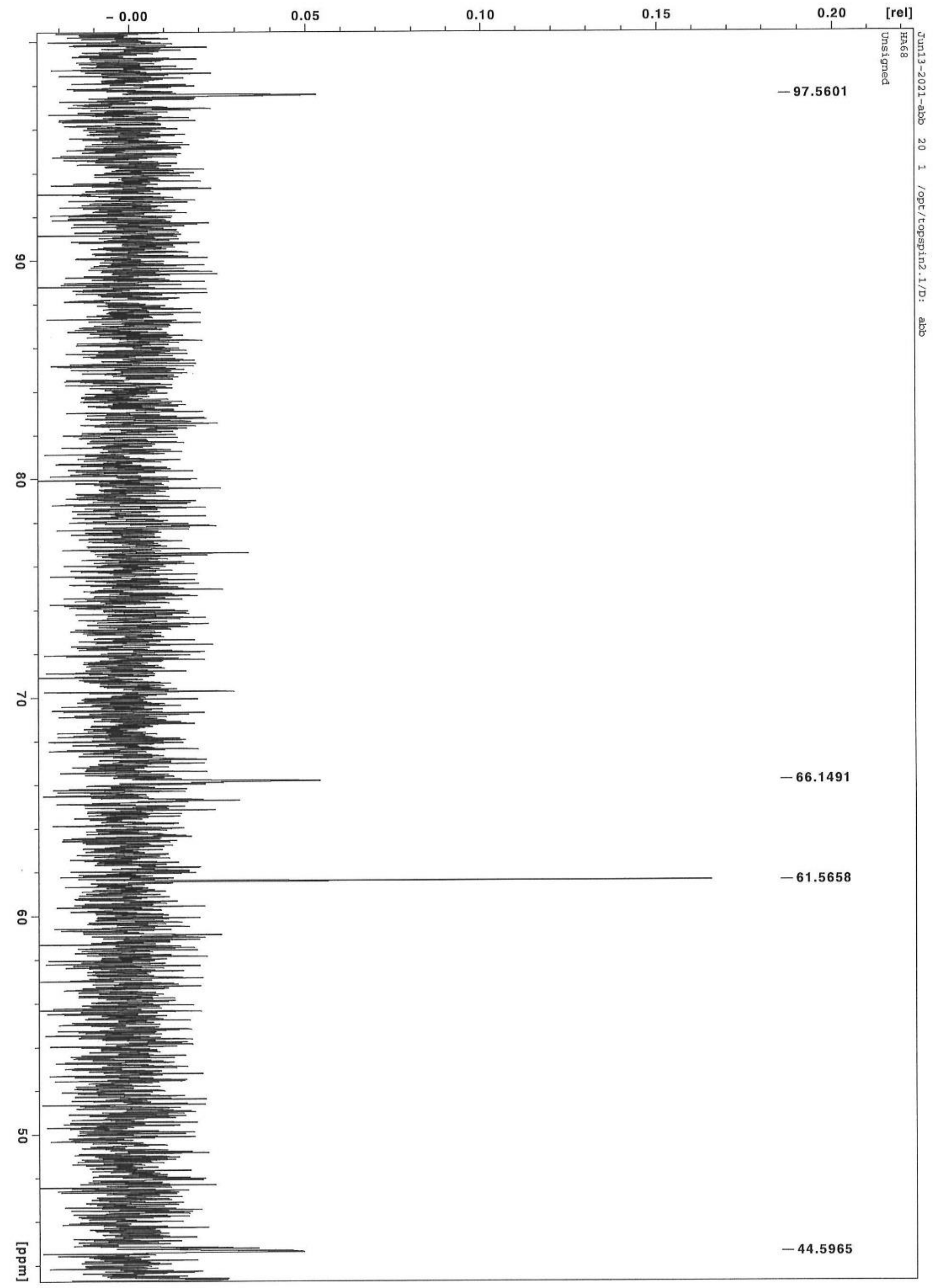

Figure S105. Detail of ^{13}C spectrum of compound **7e.**

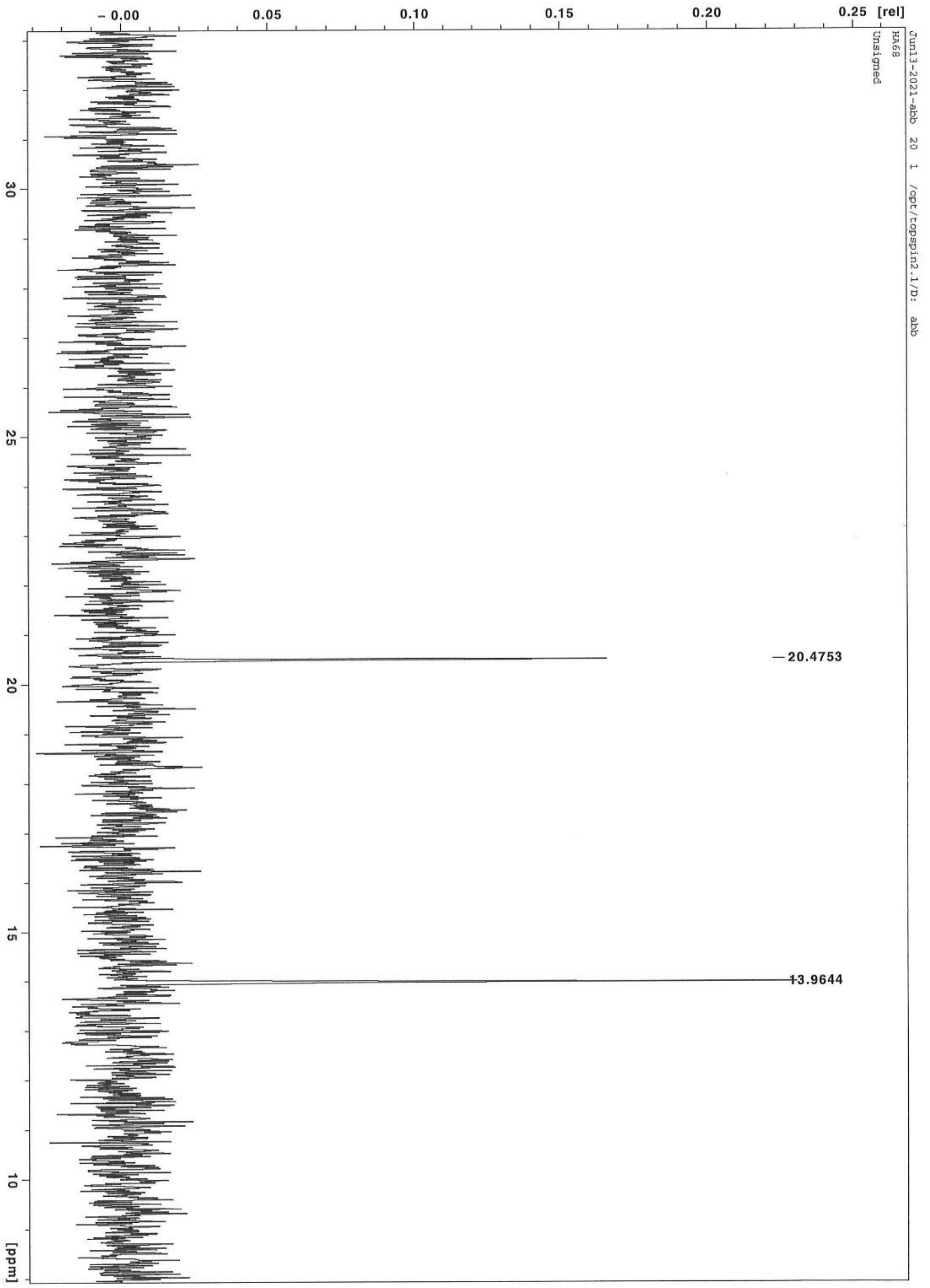

Figure S106. Detail of ^{13}C spectrum of compound **7e.**

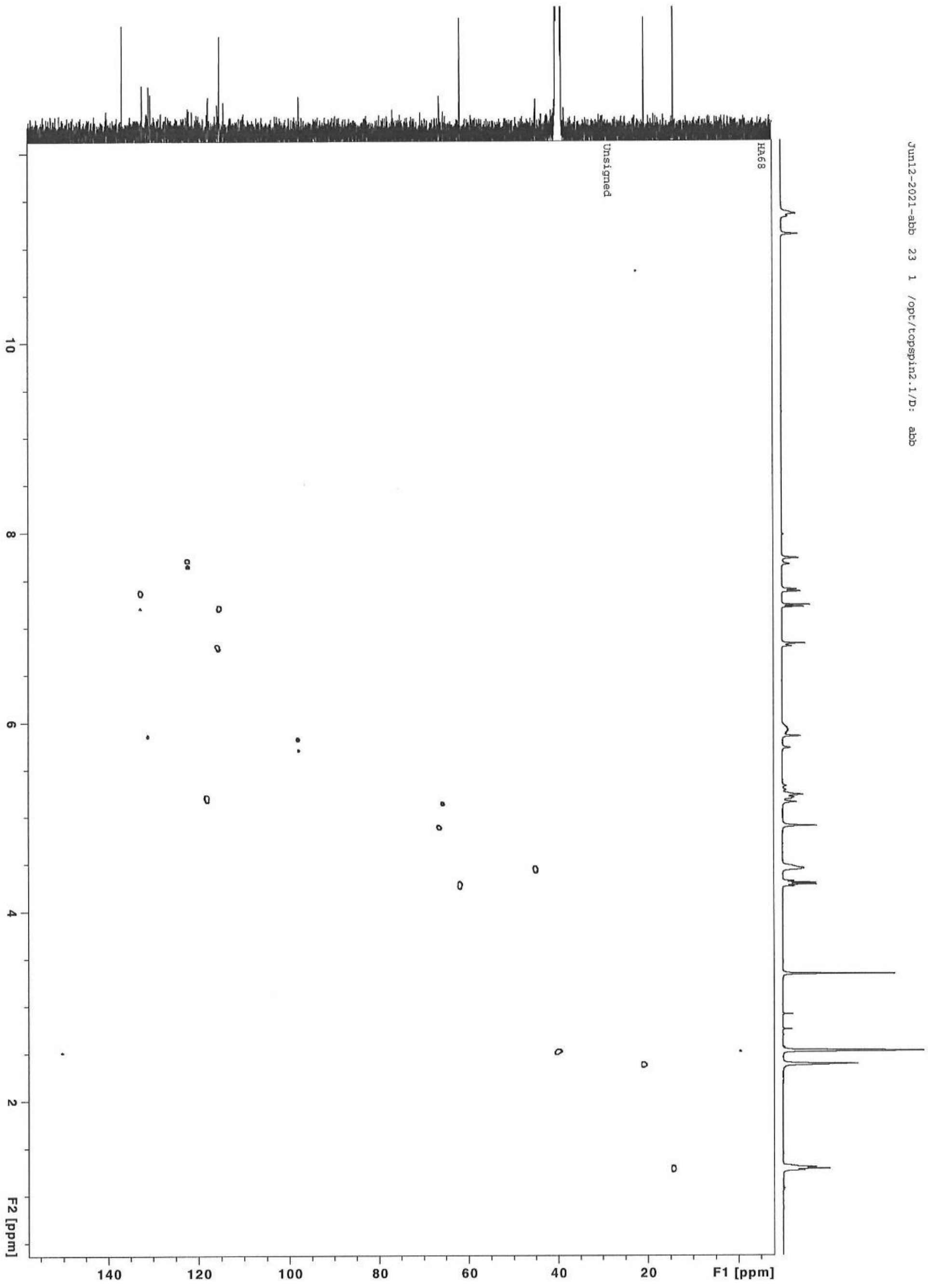

Figure S107. ^{1}H-^{13}C HSQC spectrum of compound **7e.**

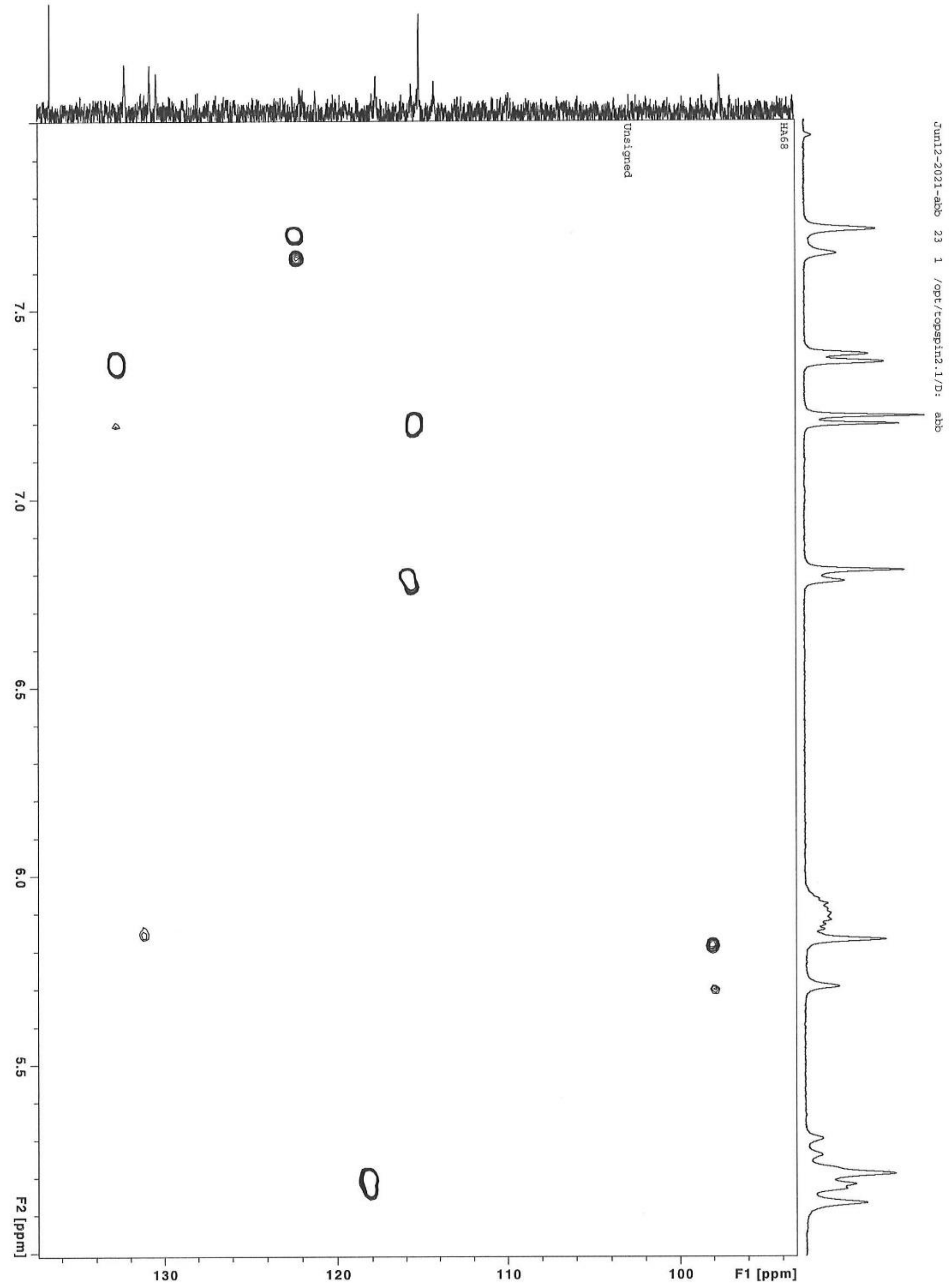

Figure S108. Detail of ^{1}H-^{13}C HSQC spectrum of compound **7e.**

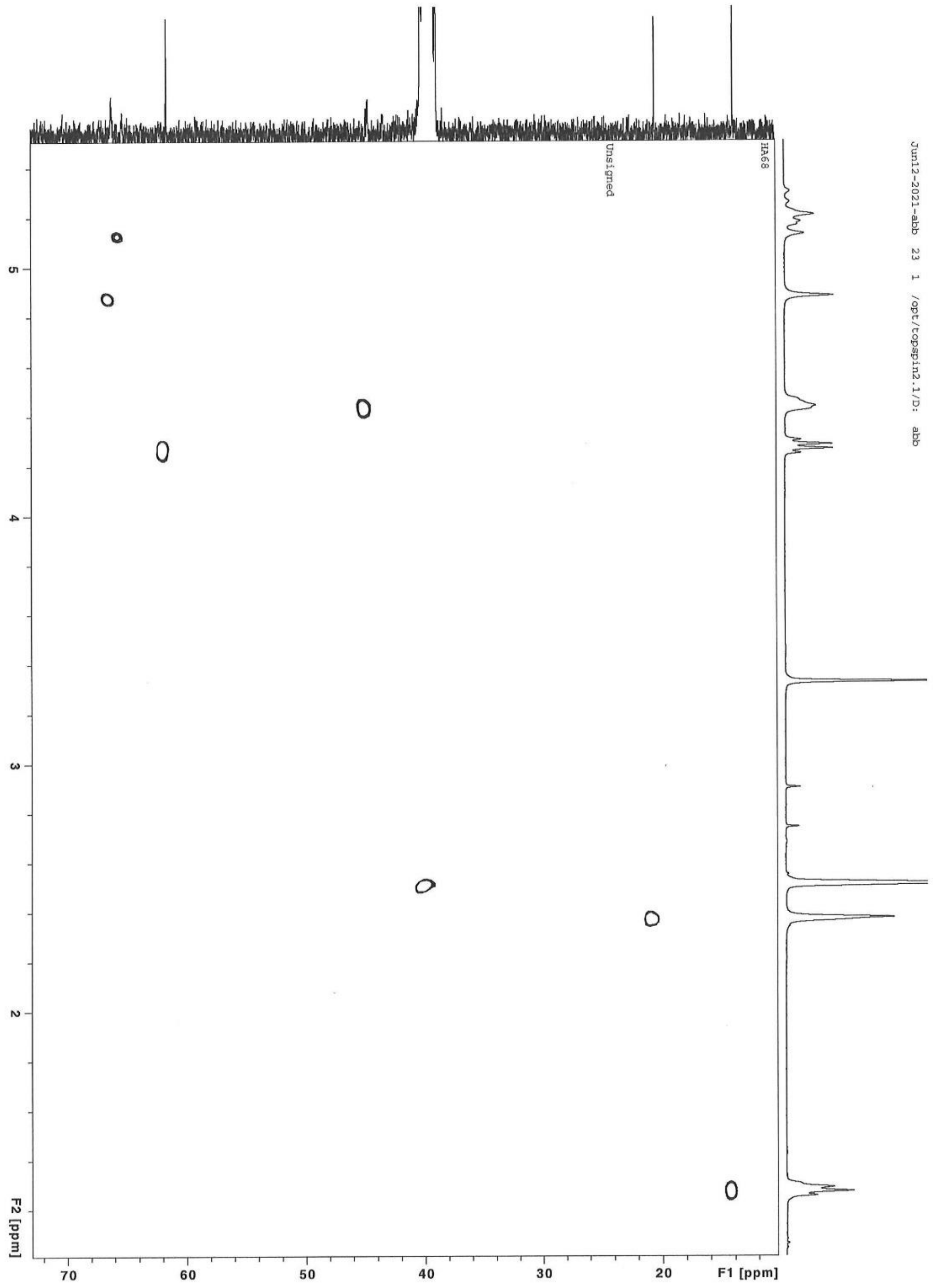

Figure S109. Detail of ^{1}H-^{13}C HSQC spectrum of compound **7e.**

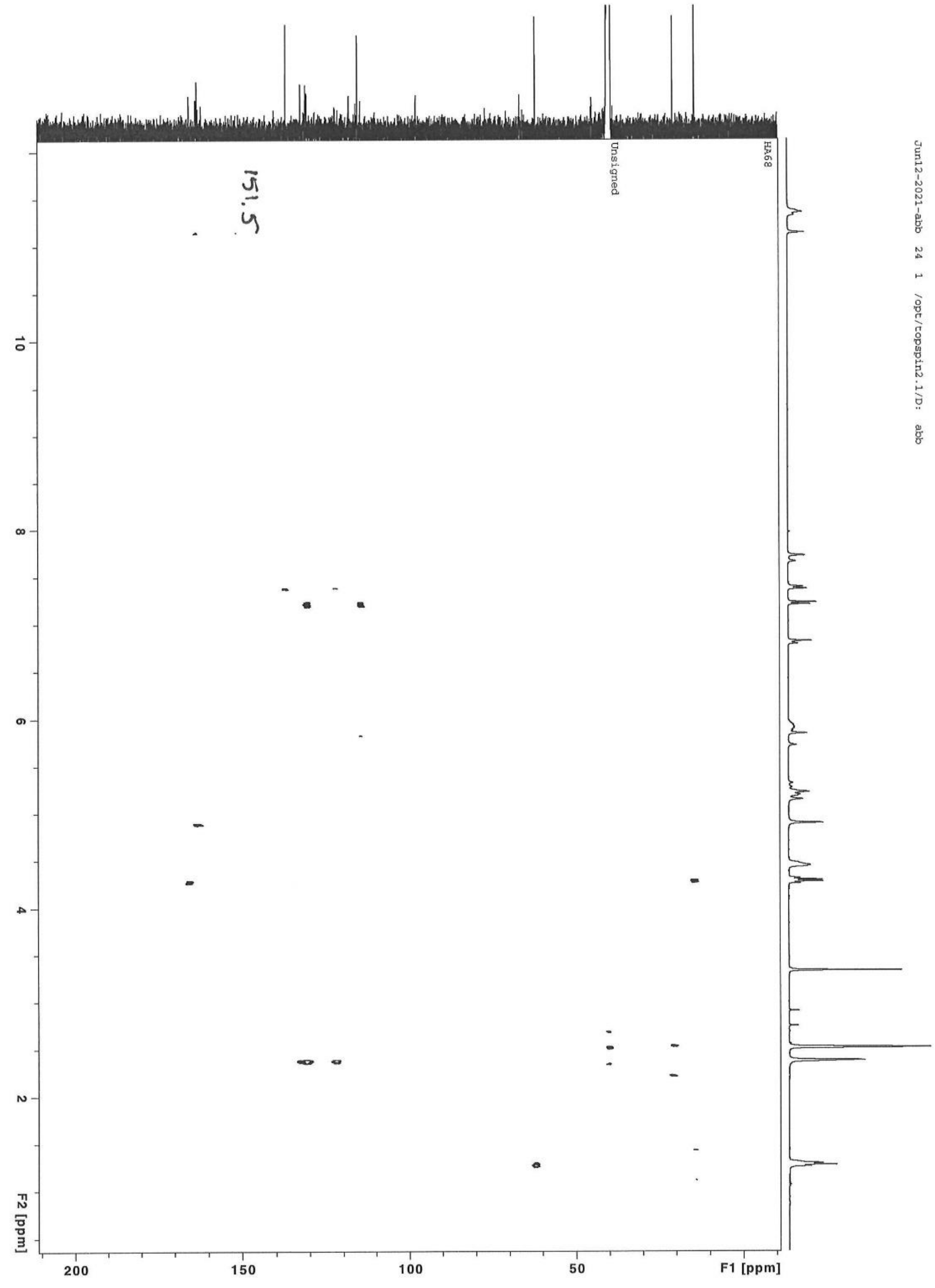

Figure S110. ^{1}H-^{13}C HMBC spectrum of compound **7e.**

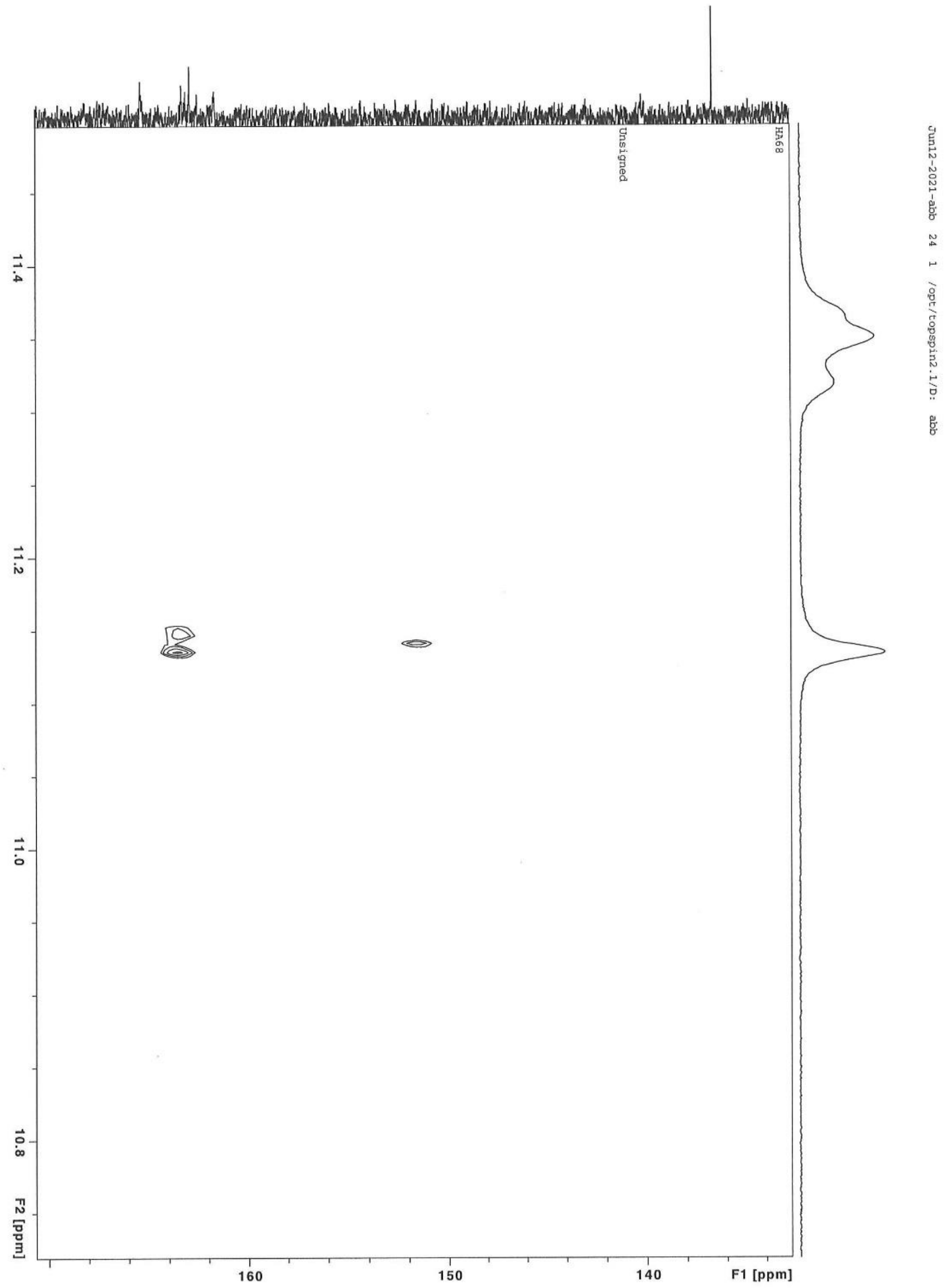

Figure S111. Detail of ^{1}H-^{13}C HMBC spectrum of compound **7e.**

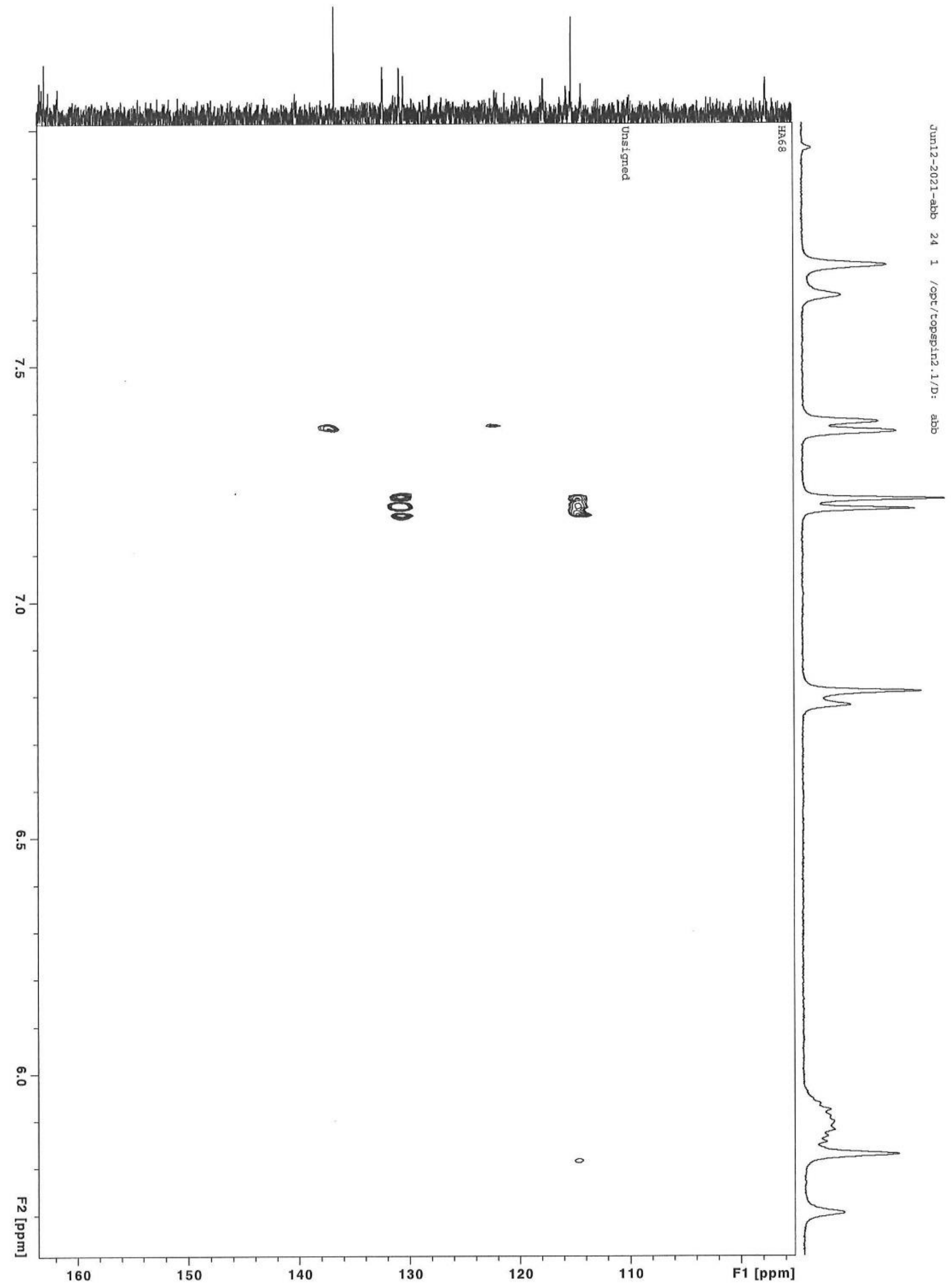

Figure S112. Detail of ^{1}H-^{13}C HMBC spectrum of compound **7e.**

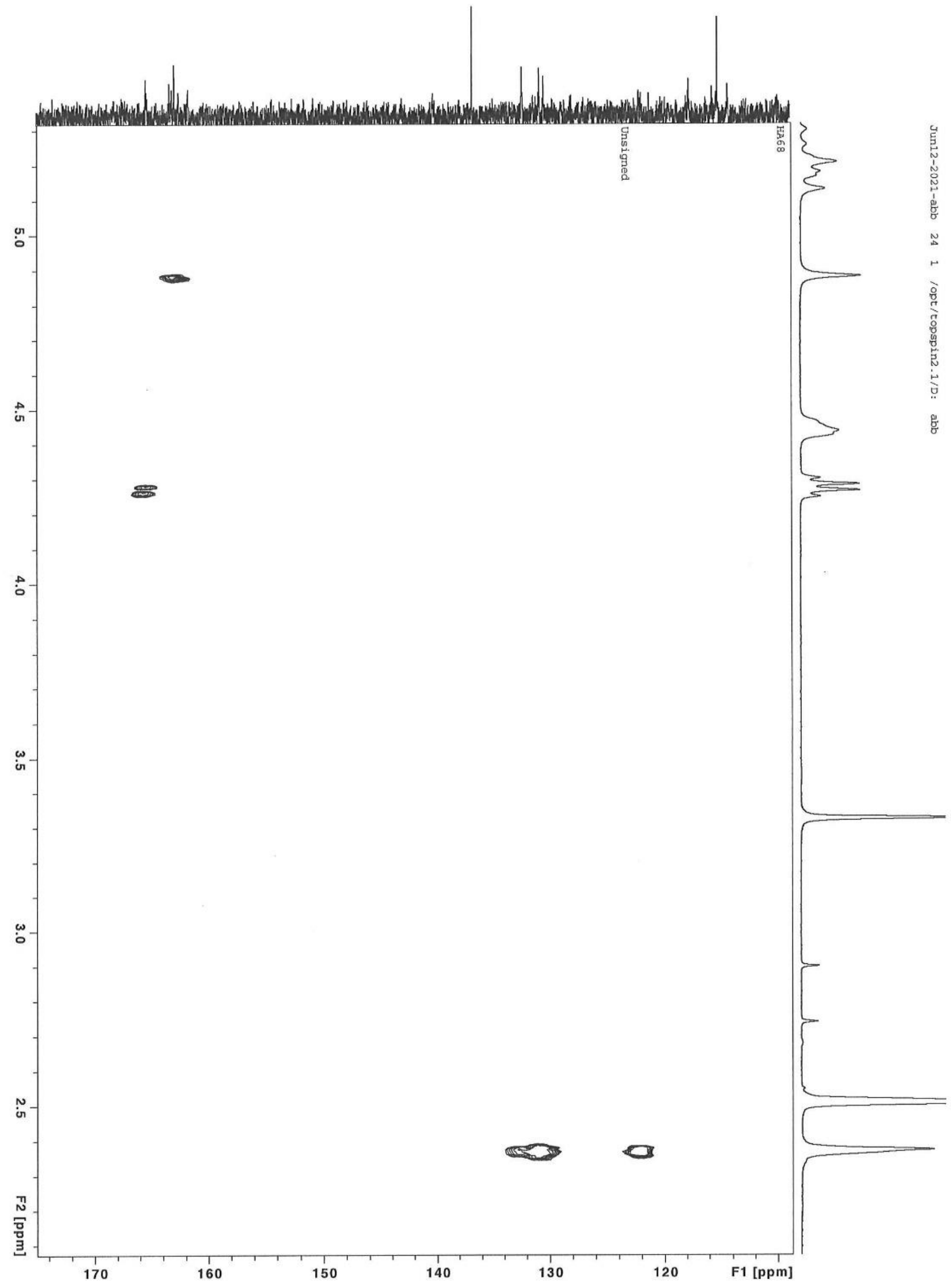

Figure S113. Detail of ^{1}H-^{13}C HMBC spectrum of compound **7e.**

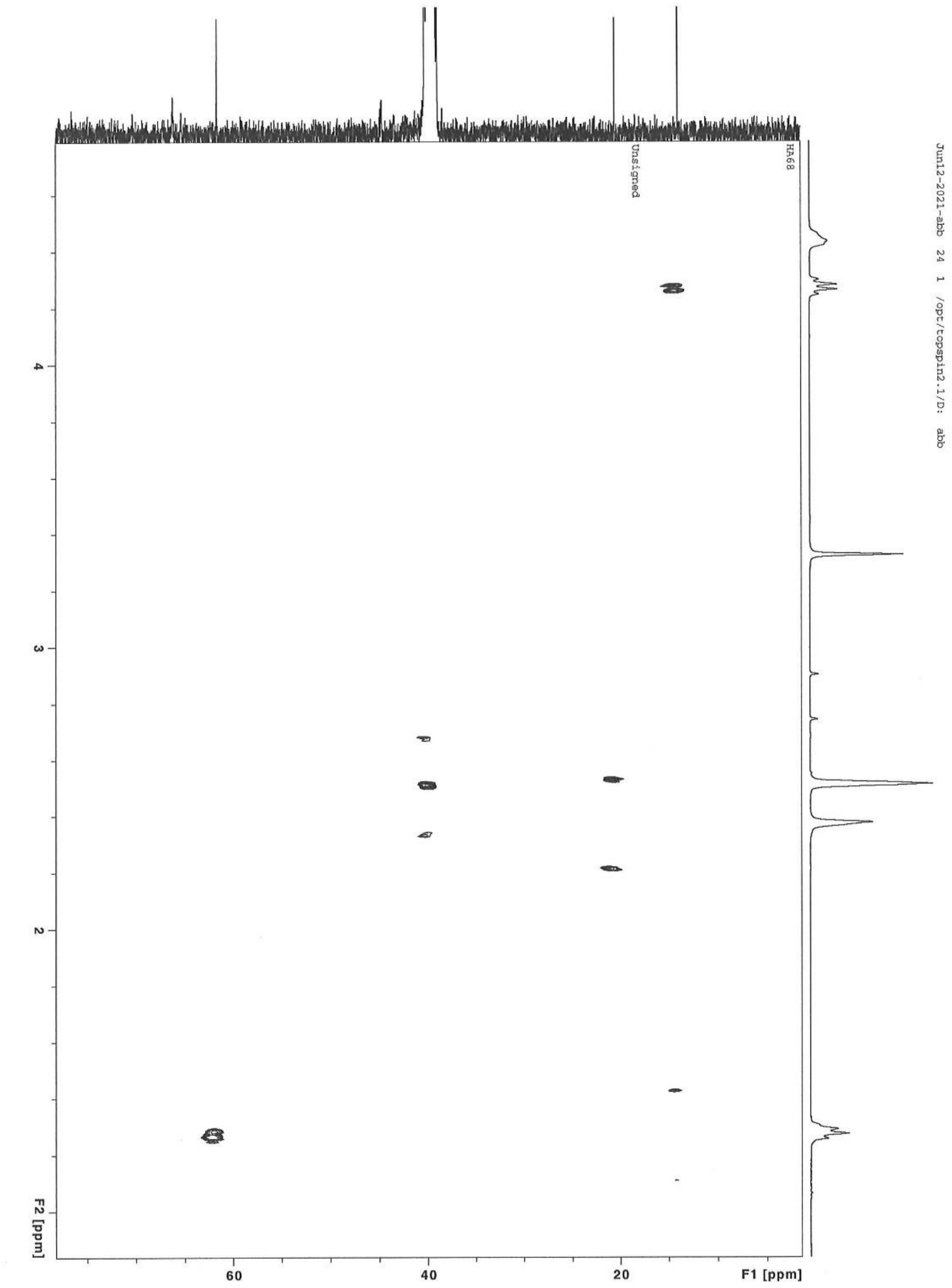

Figure S114. Detail of ^{1}H-^{13}C HMBC spectrum of compound **7e.**

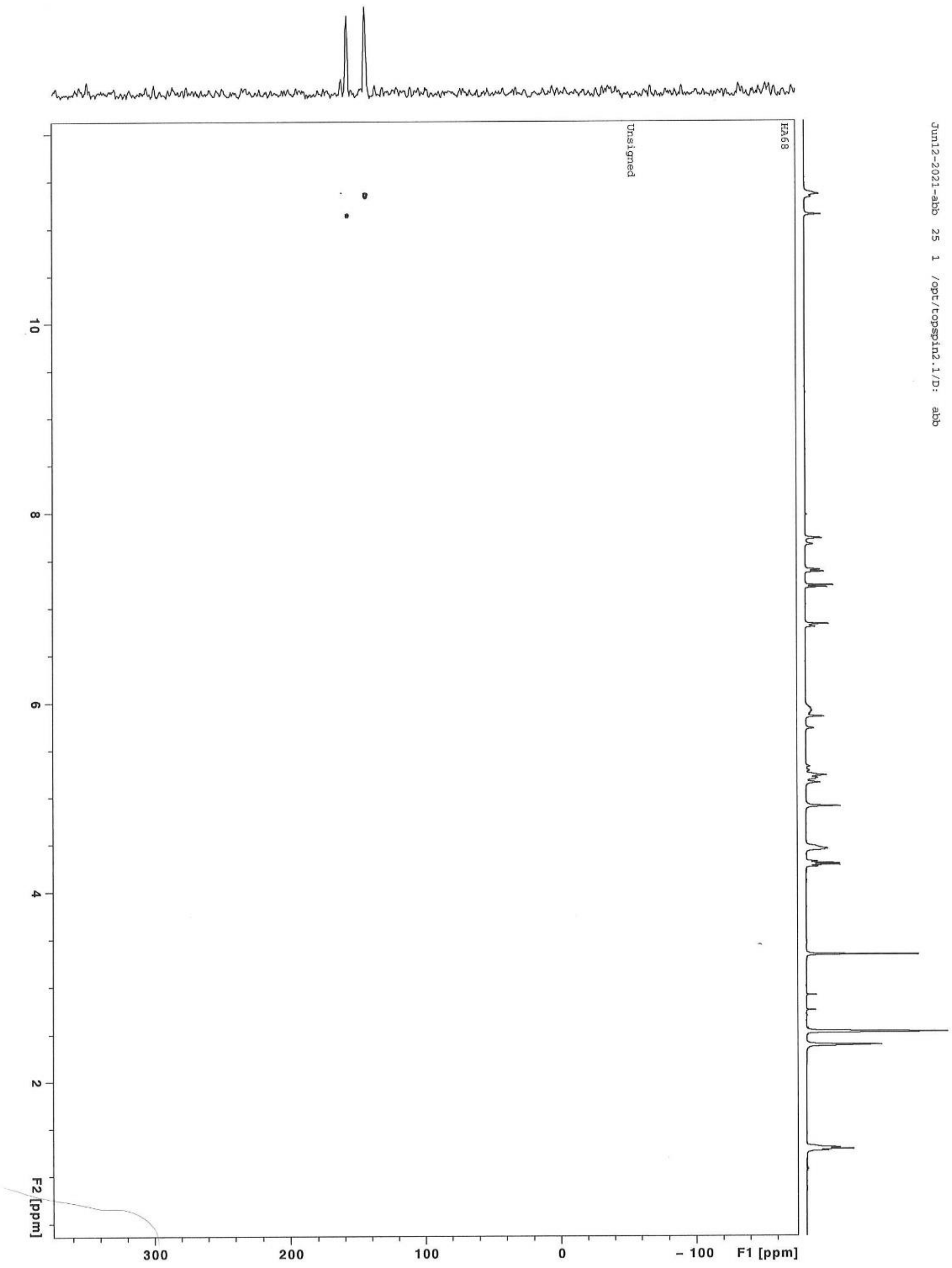

Figure S115. ^{1}H-^{15}N HSQC spectrum of compound **7e** (^{1}H-^{15}N HMBC spectrum gave no cross peaks).

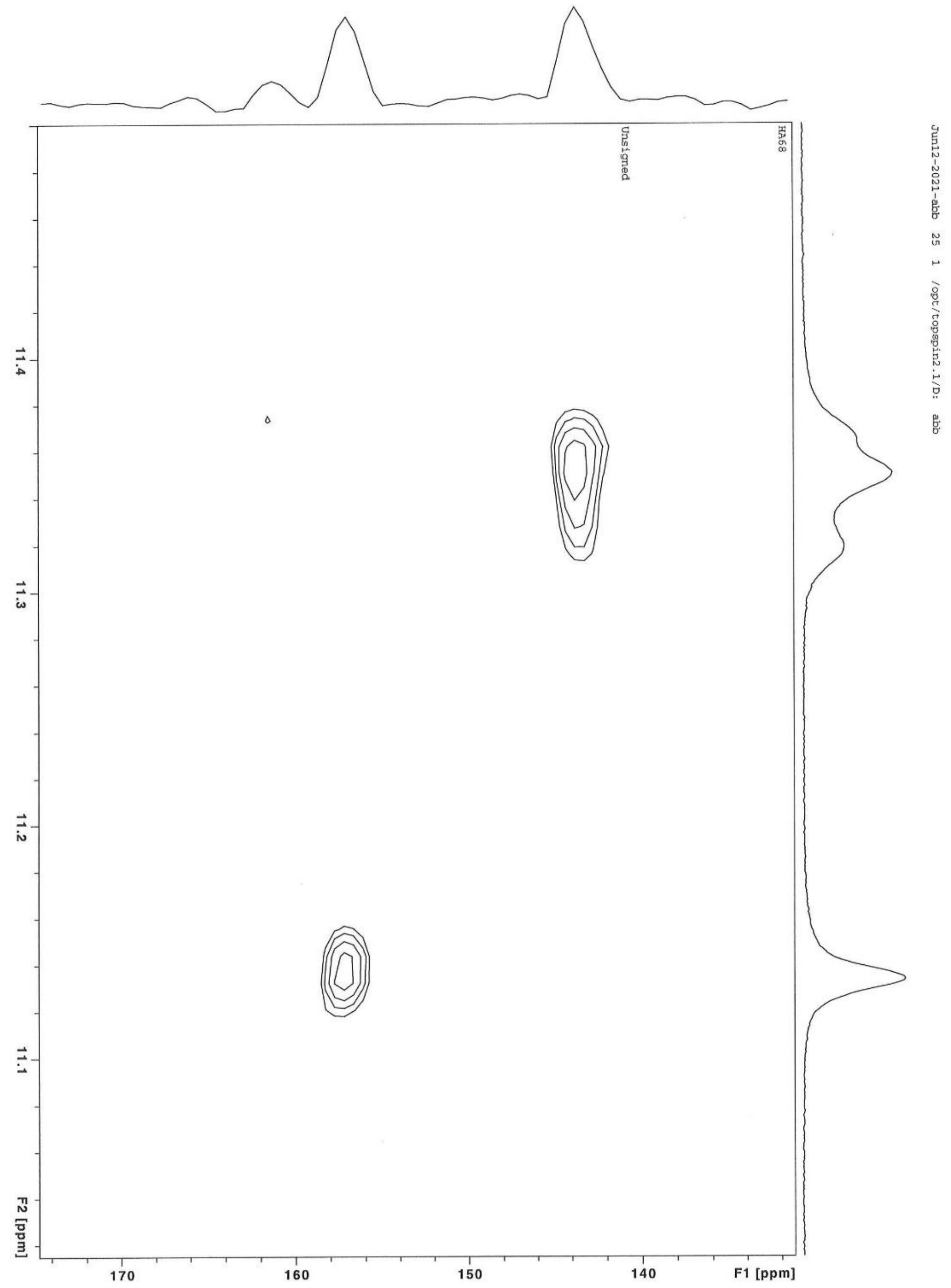

Figure S116. Detail of ^{1}H-^{15}N HSQC spectrum of compound **7e.**

Supplementary Material, part 2

Y.A.M.M. Elshaier, A.A. Aly,* M. Abd El-Aziz, H.M. Fathy, A.B. Brown, S. Bräse, and M. Ramadan

"Synthesis and identification of new *N*,*N*-disubstituted thiourea and thiazolidinone scaffolds based on quinolone moiety as urease inhibitors"

Contents

Compound **7f** **S117-S139**

Compound **7g** **S140-S159**

Compound **7h** **S160-S171**

Compound **7j** **S172-S189**

Compound **7k** **S190-S205**

Compound **7l** **S206-S228**

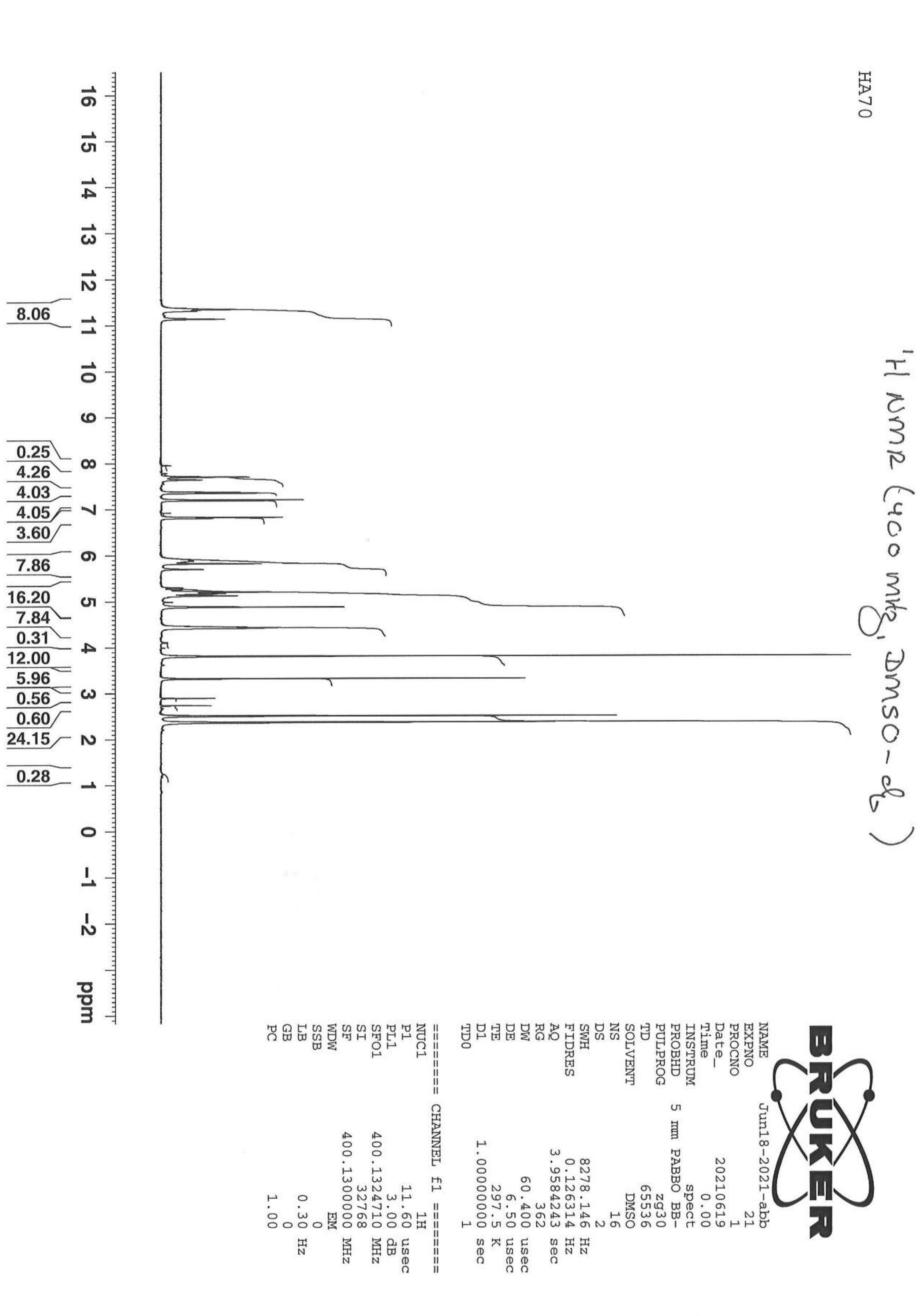

Figure S117. ^{1}H NMR spectrum of compound **7f** (400 MHz, DMSO-d_6).

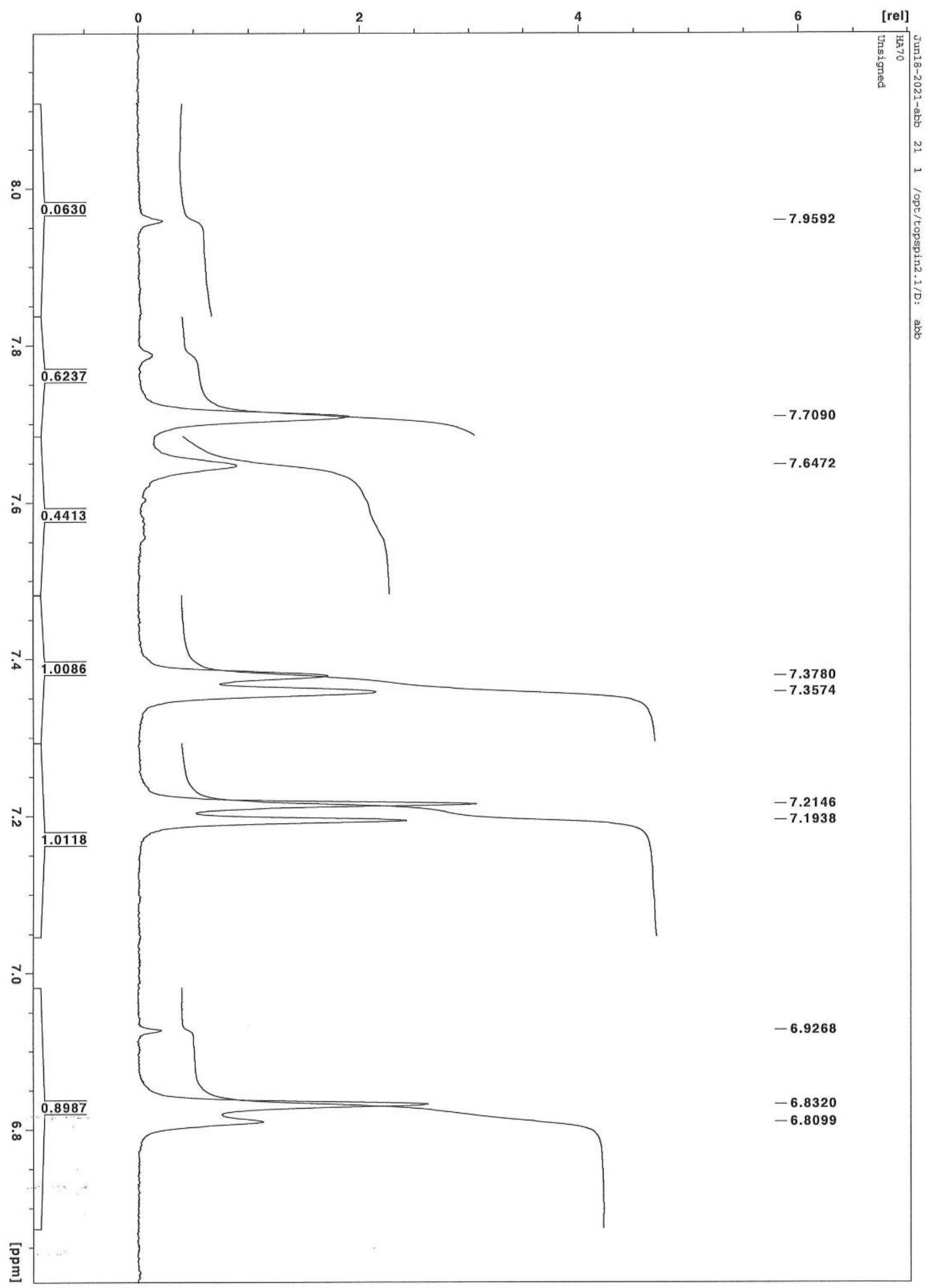

Figure S118. Detail of ^{1}H NMR spectrum of compound **7f.**

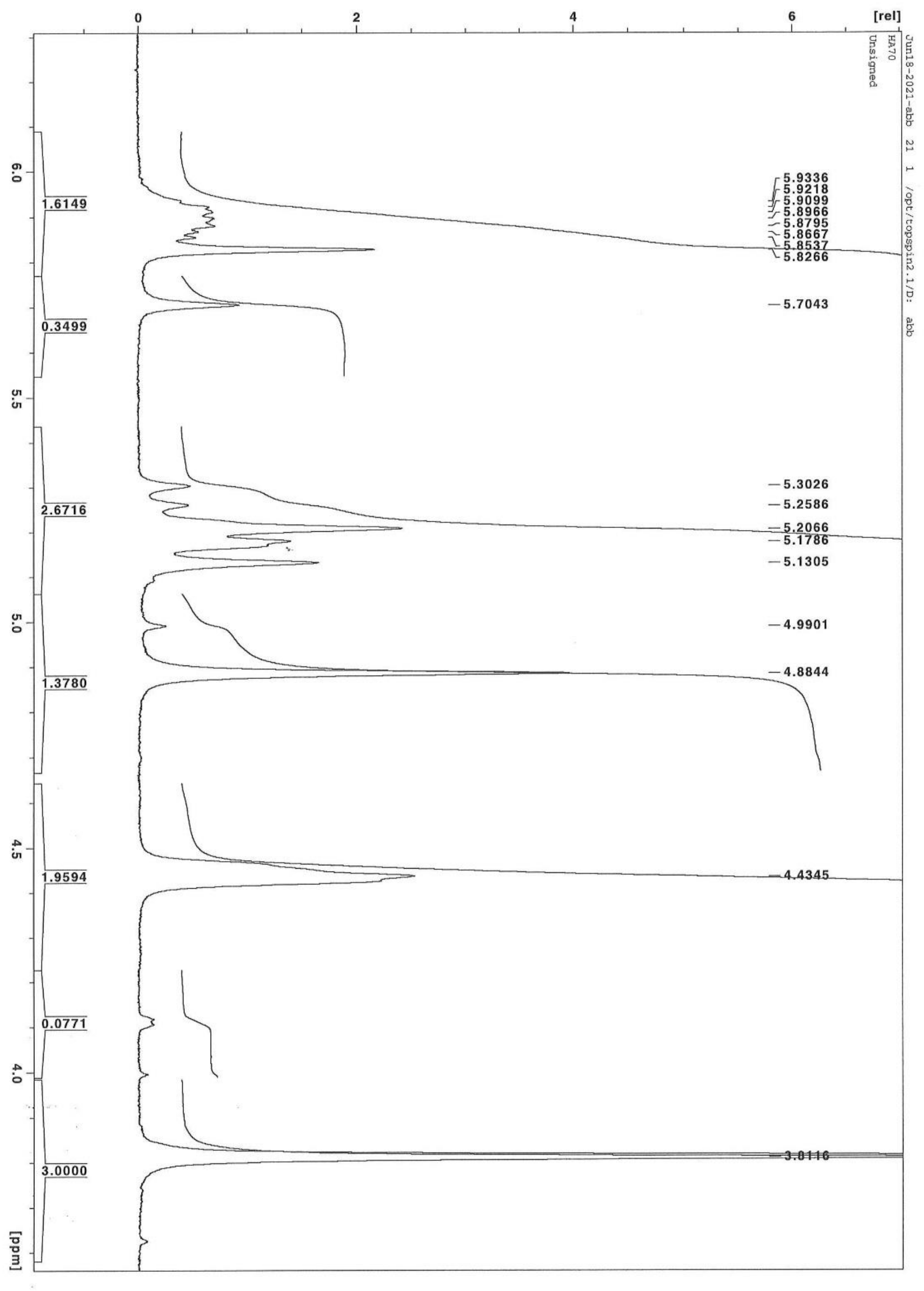

Figure S119. Detail of ^{1}H NMR spectrum of compound **7f.**

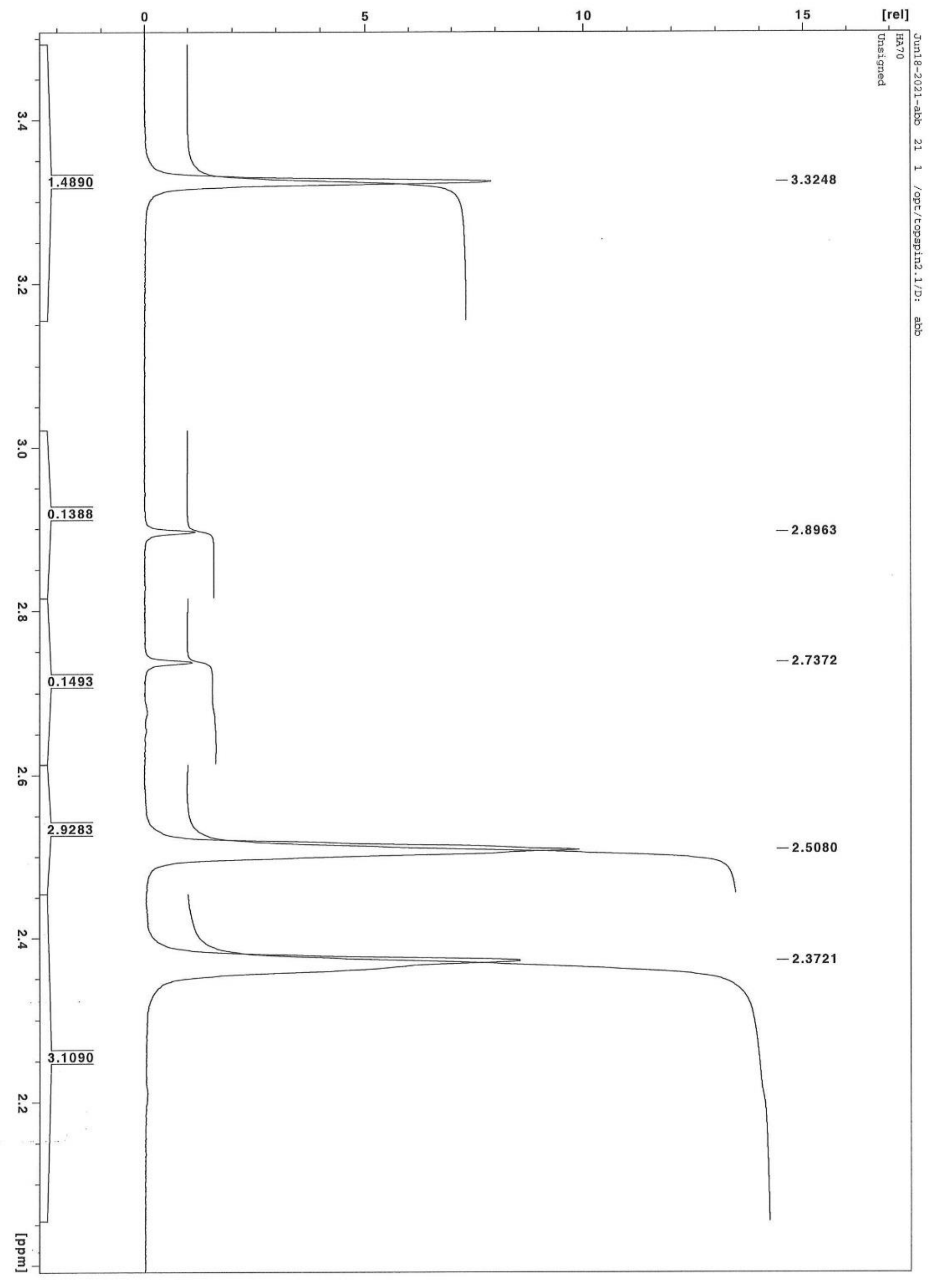

Figure S120. Detail of ^{1}H NMR spectrum of compound **7f.**

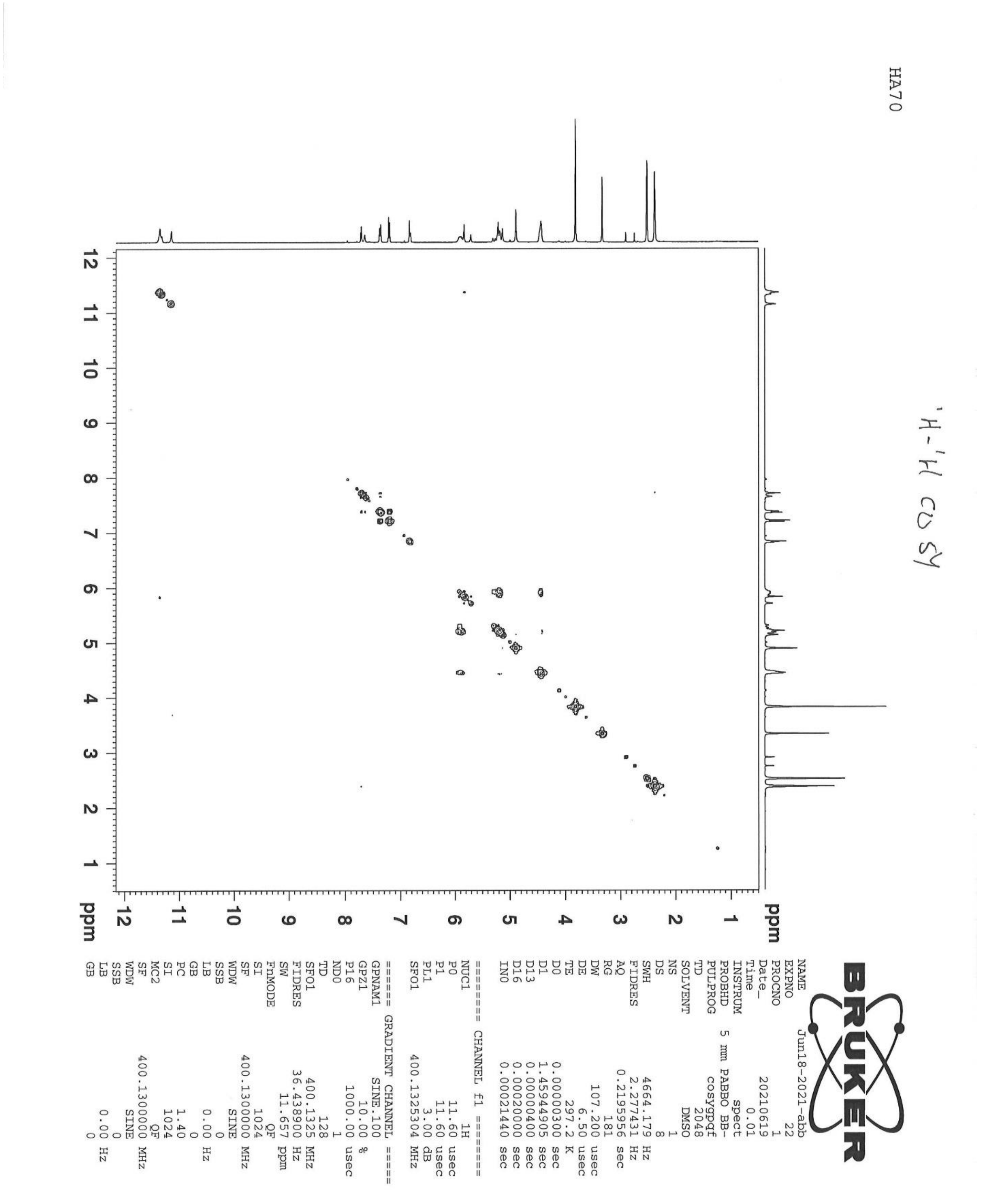

Figure S121. ^{1}H-^{1}H COSY spectrum of compound **7f.**

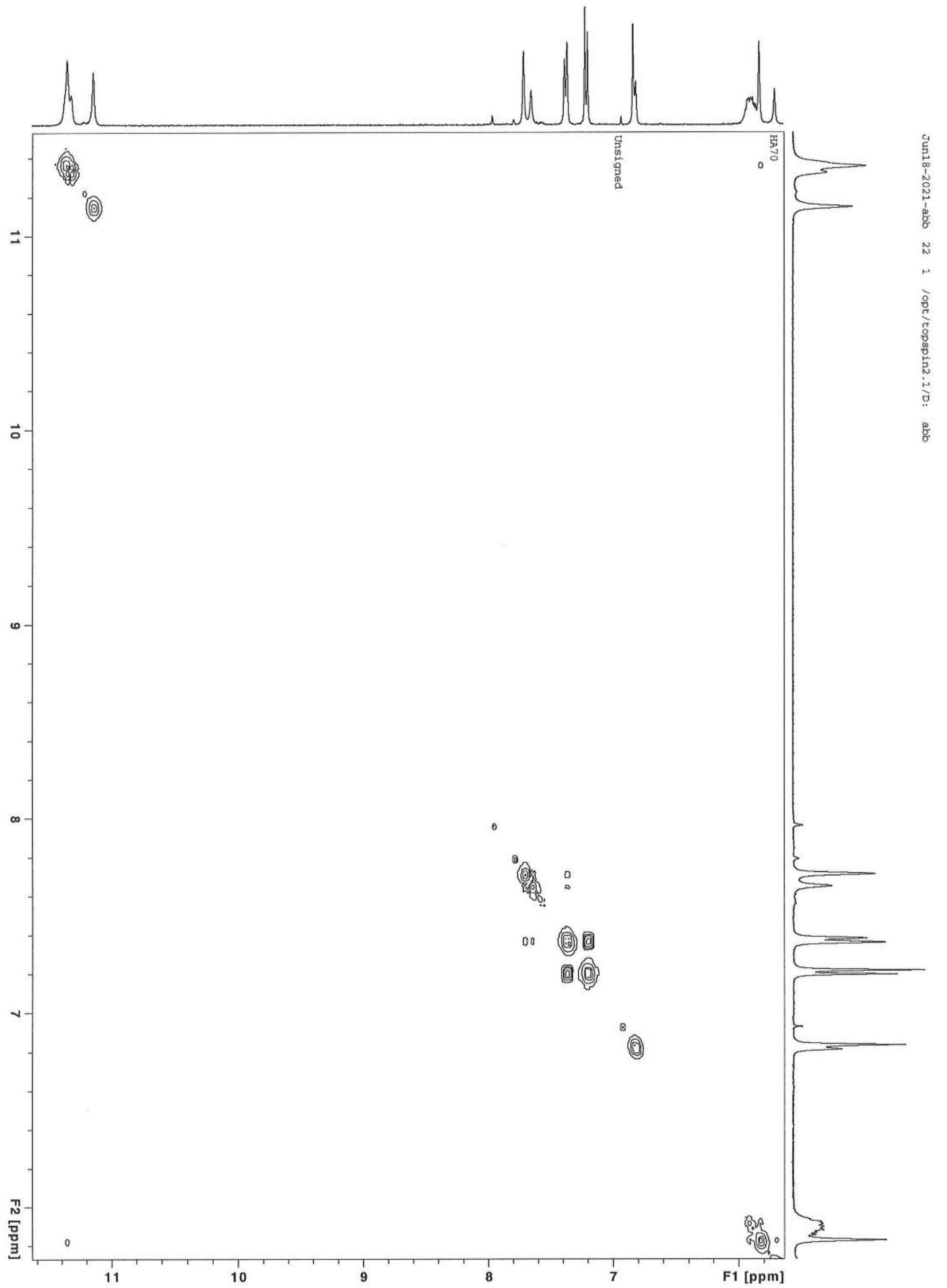

Figure S122. Detail of ^{1}H-^{1}H COSY spectrum of compound **7f.**

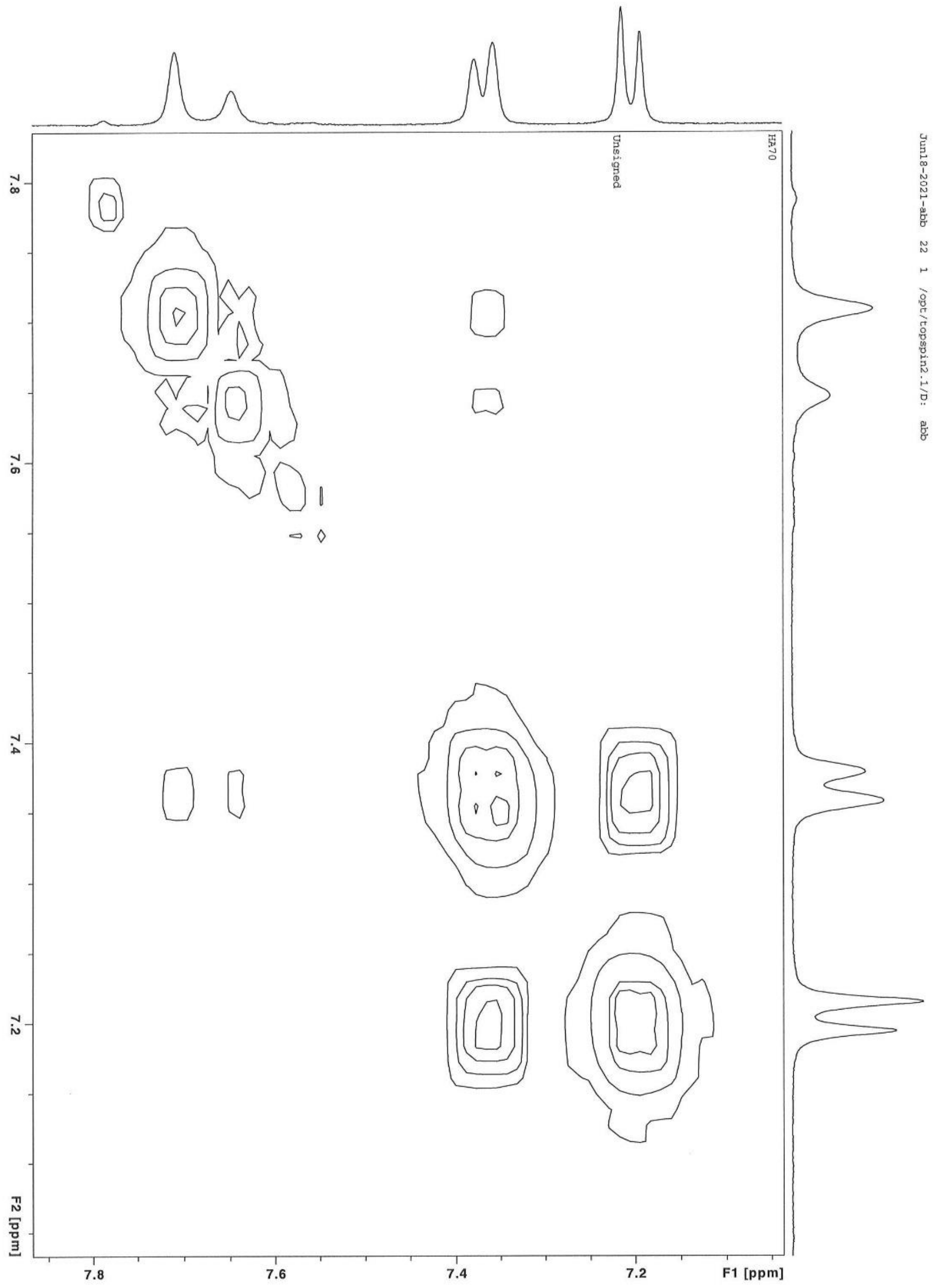

Figure S123. Detail of ^{1}H-^{1}H COSY spectrum of compound **7f.**

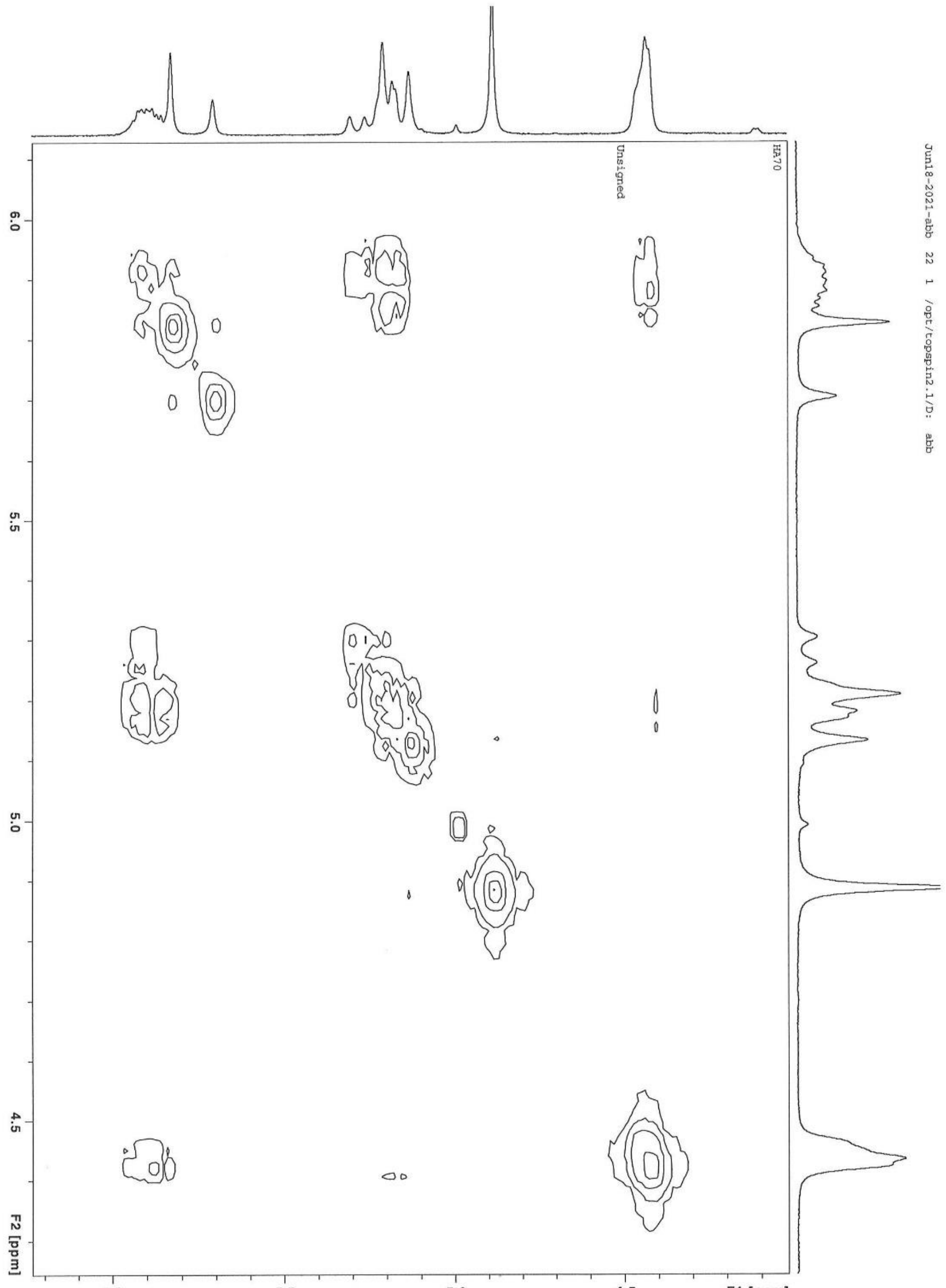

Figure S124. Detail of ^{1}H-^{1}H spectrum of compound **7f.**

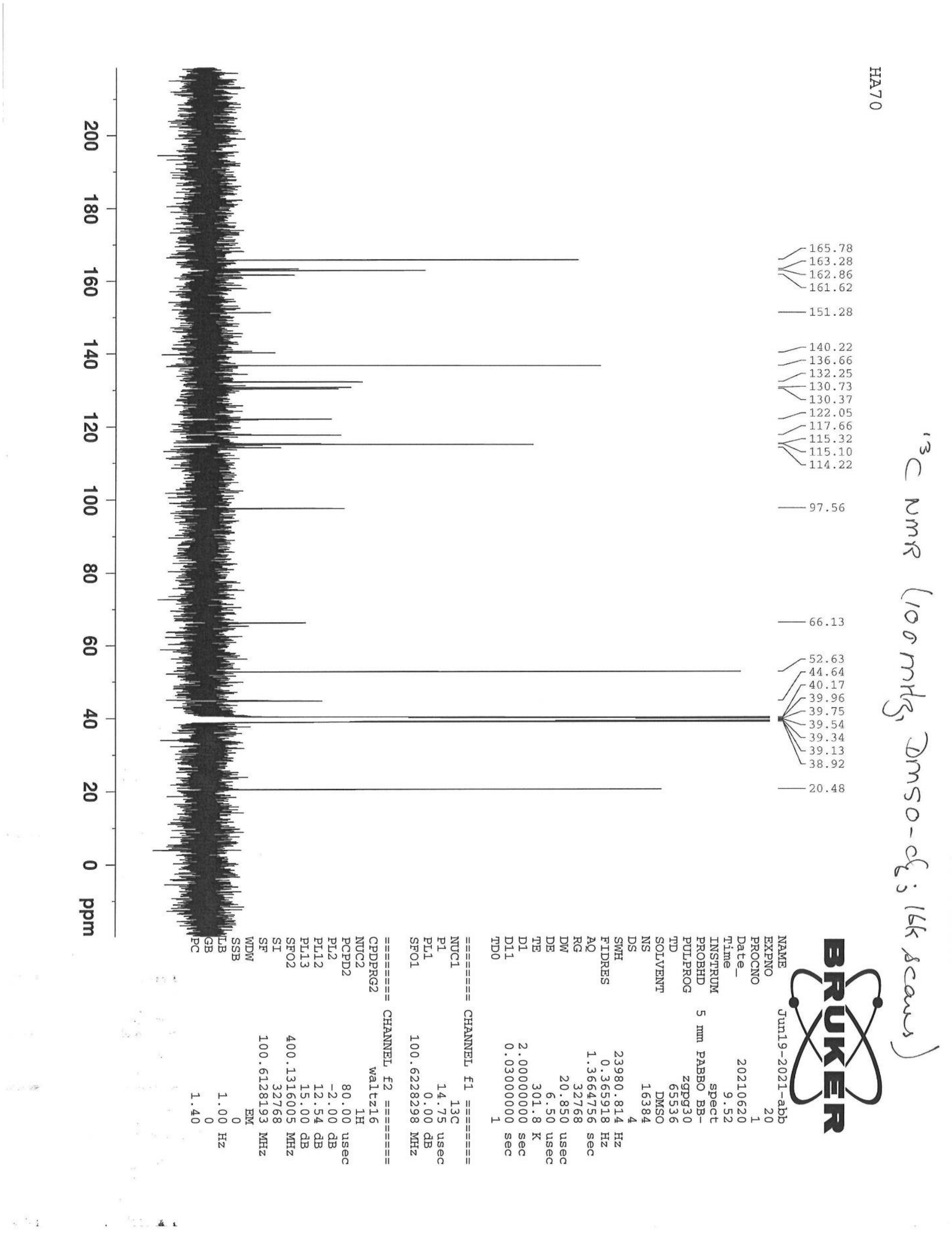

Figure S125. ^{13}C NMR spectrum of compound **7f** (100 MHz, DMSO-d_6).

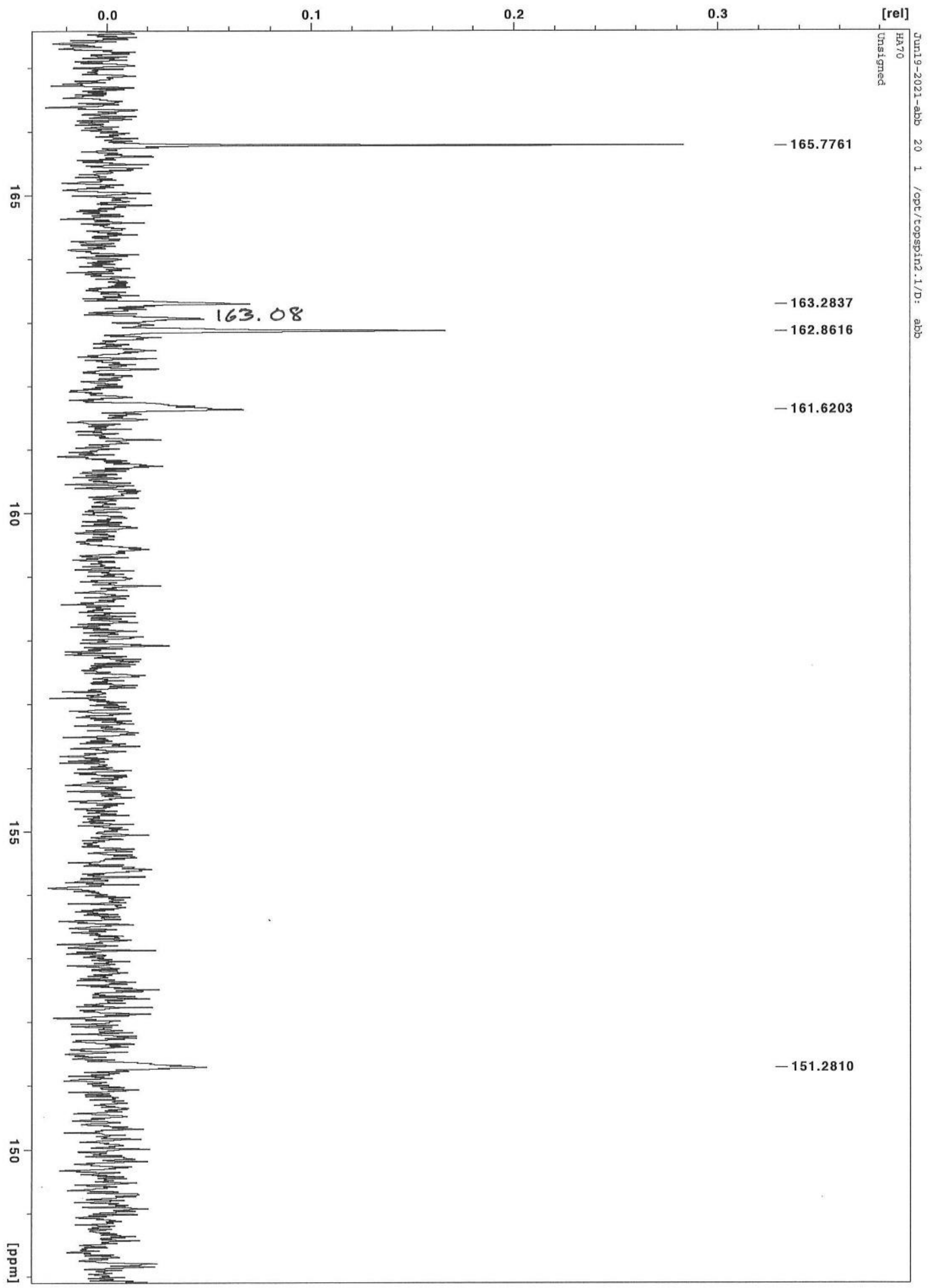

Figure S126. Detail of ^{13}C NMR spectrum of compound **7f.**

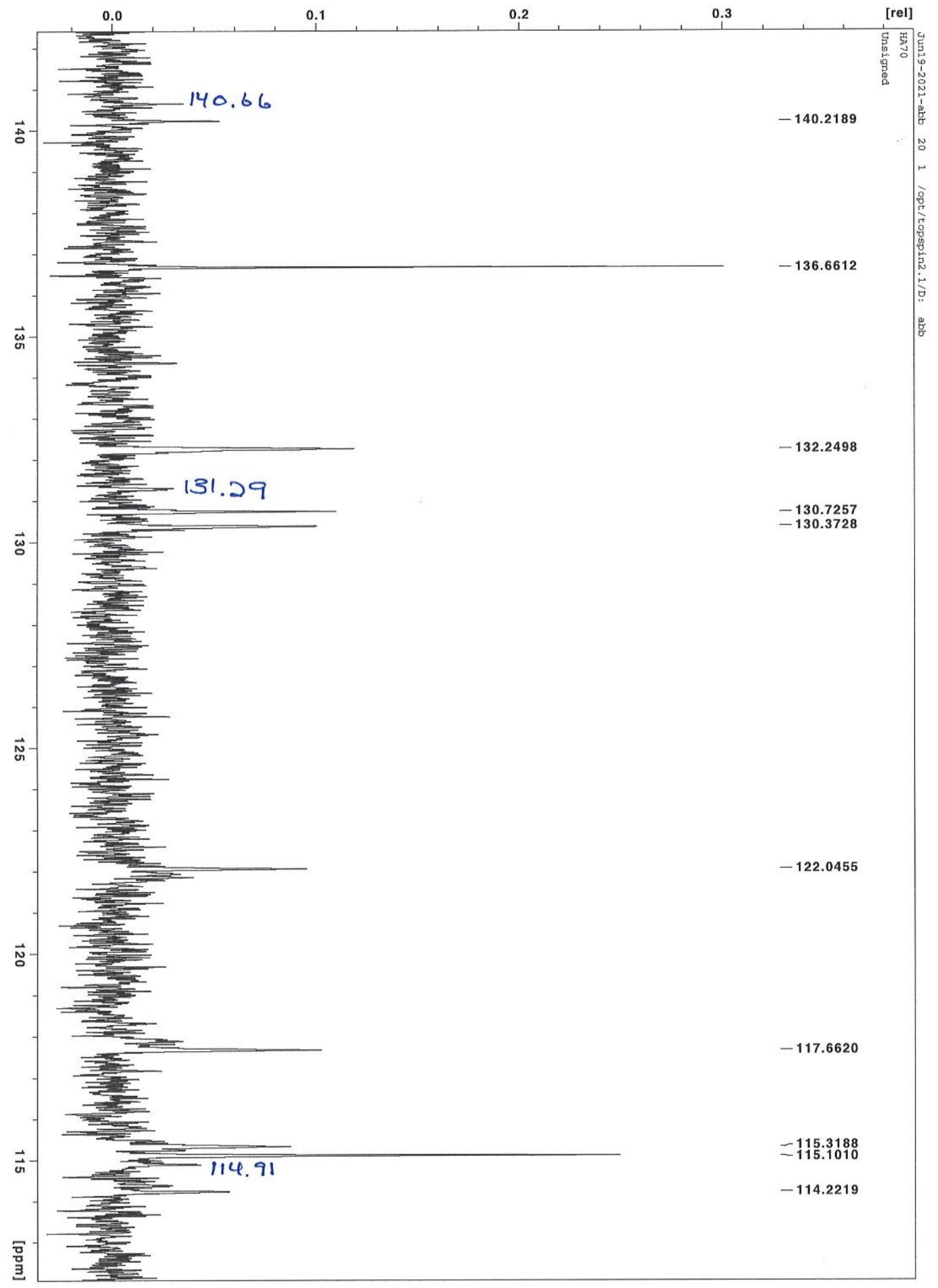

Figure S127. Detail of ^{13}C NMR spectrum of compound **7f.**

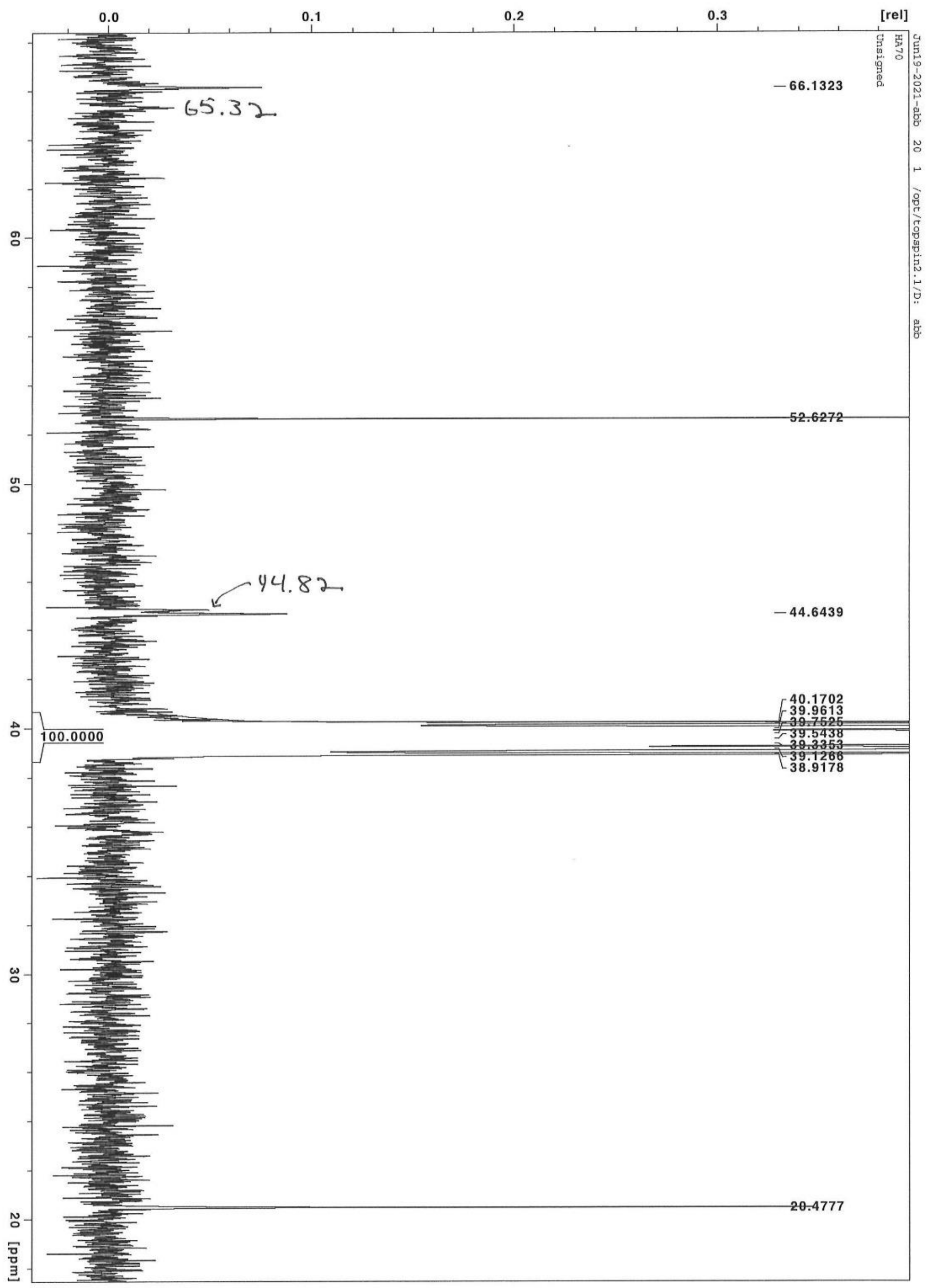

Figure S128. Detail of ^{13}C NMR spectrum of compound **7f.**

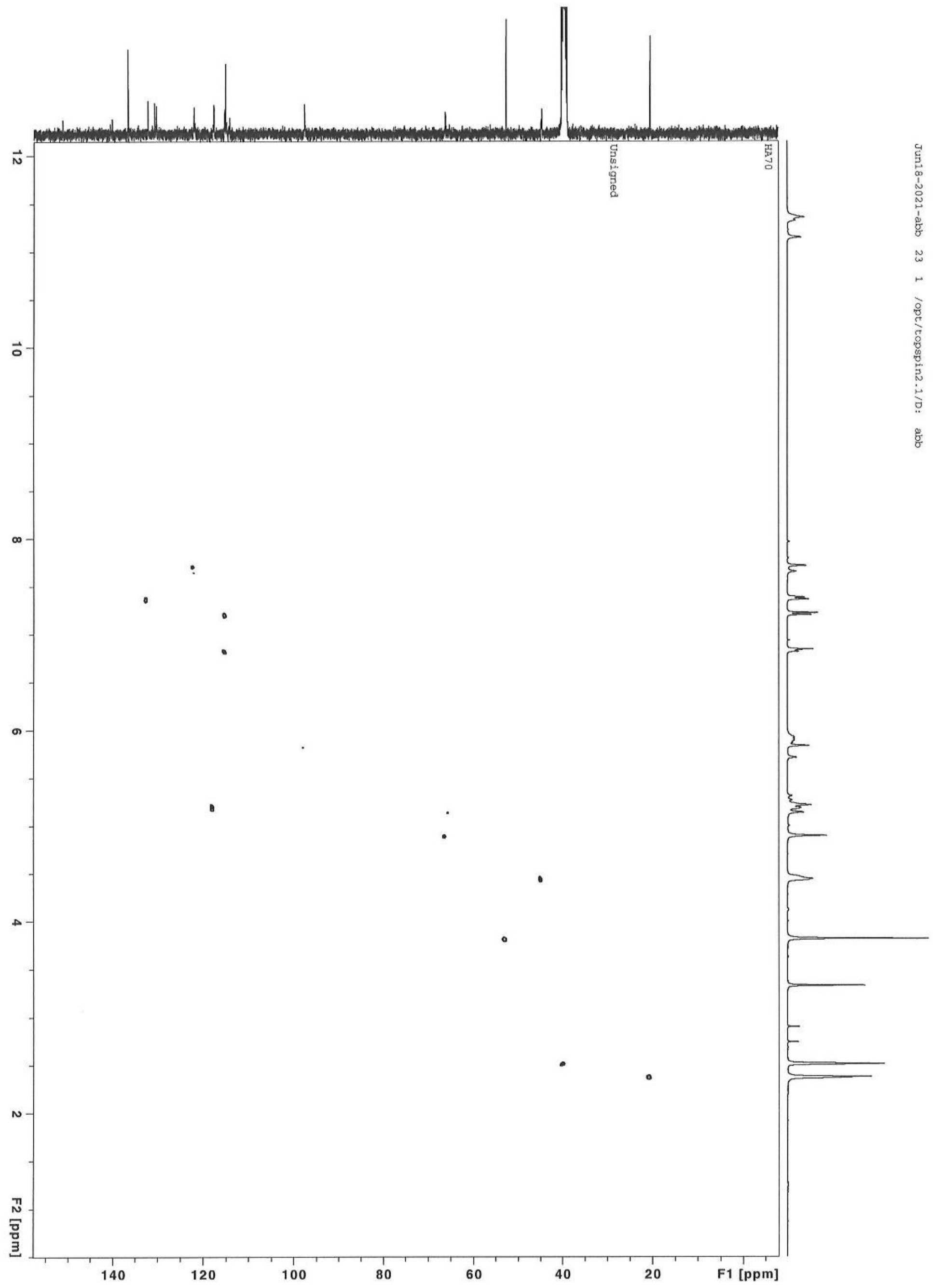

Figure S129. ^{1}H-^{13}C HSQC spectrum of compound **7f.**

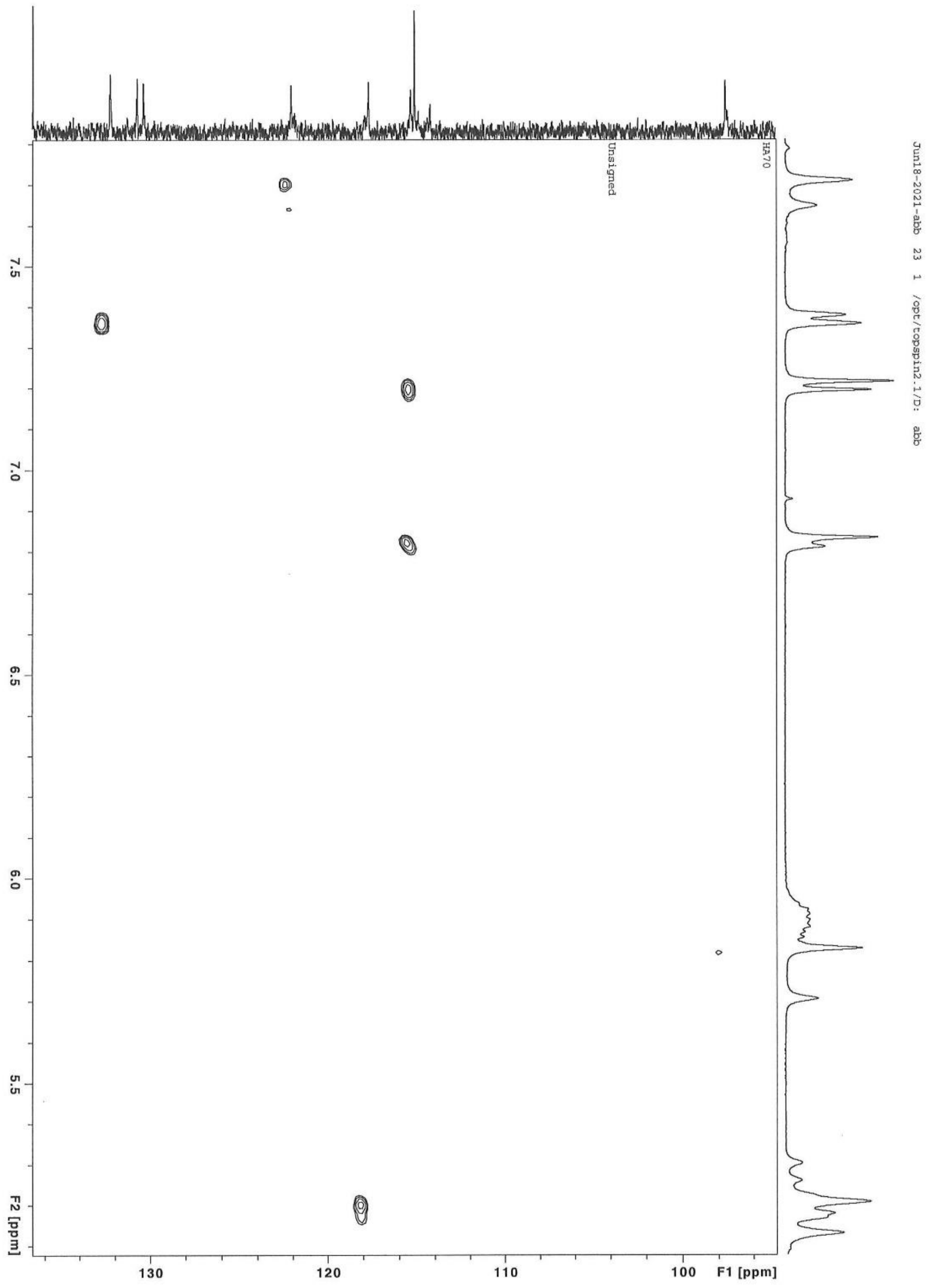

Figure S130. Detail of ^{1}H-^{13}C HSQC spectrum of compound **7f.**

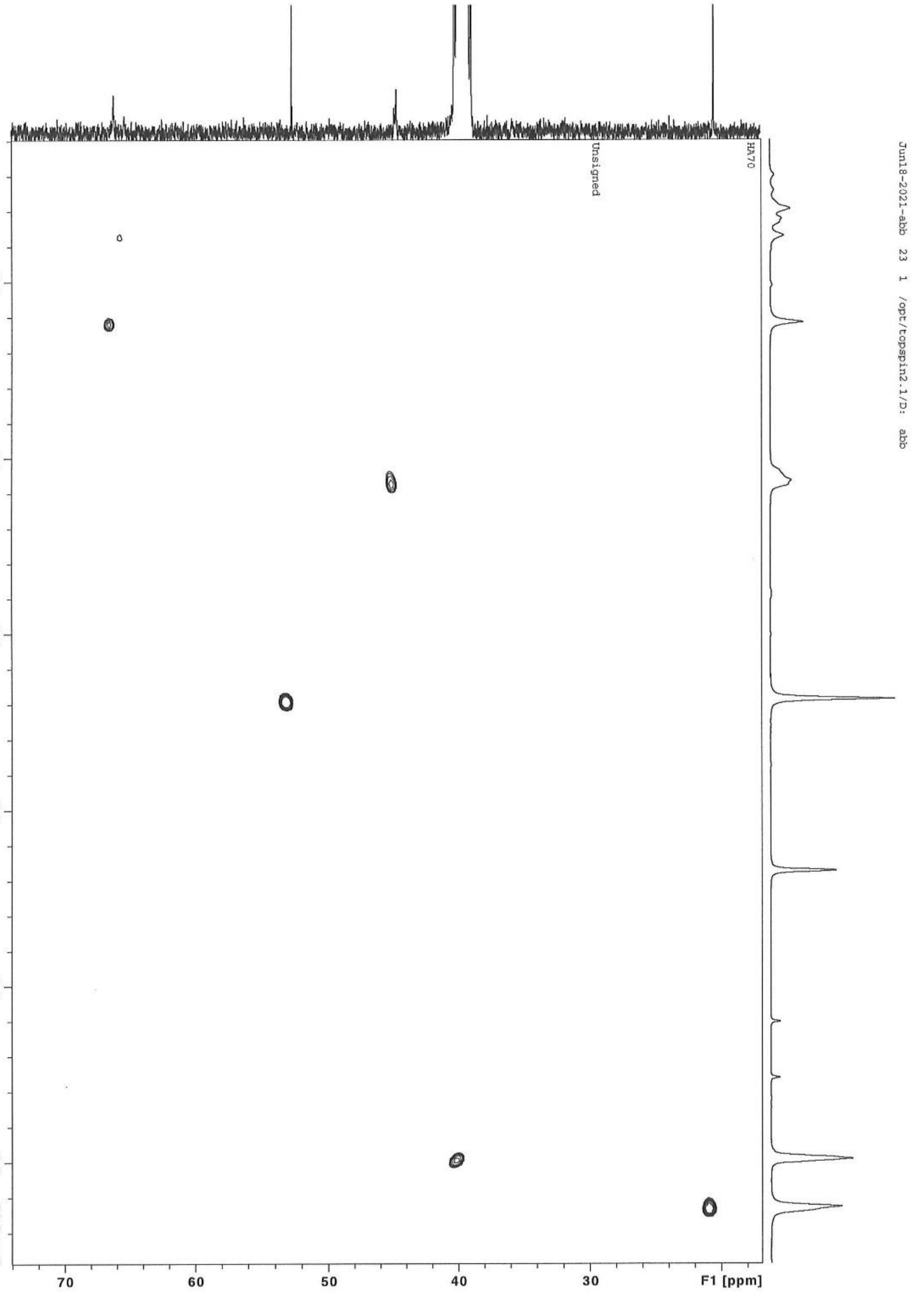

Figure S131. Detail of ^{1}H-^{13}C HSQC spectrum of compound **7f.**

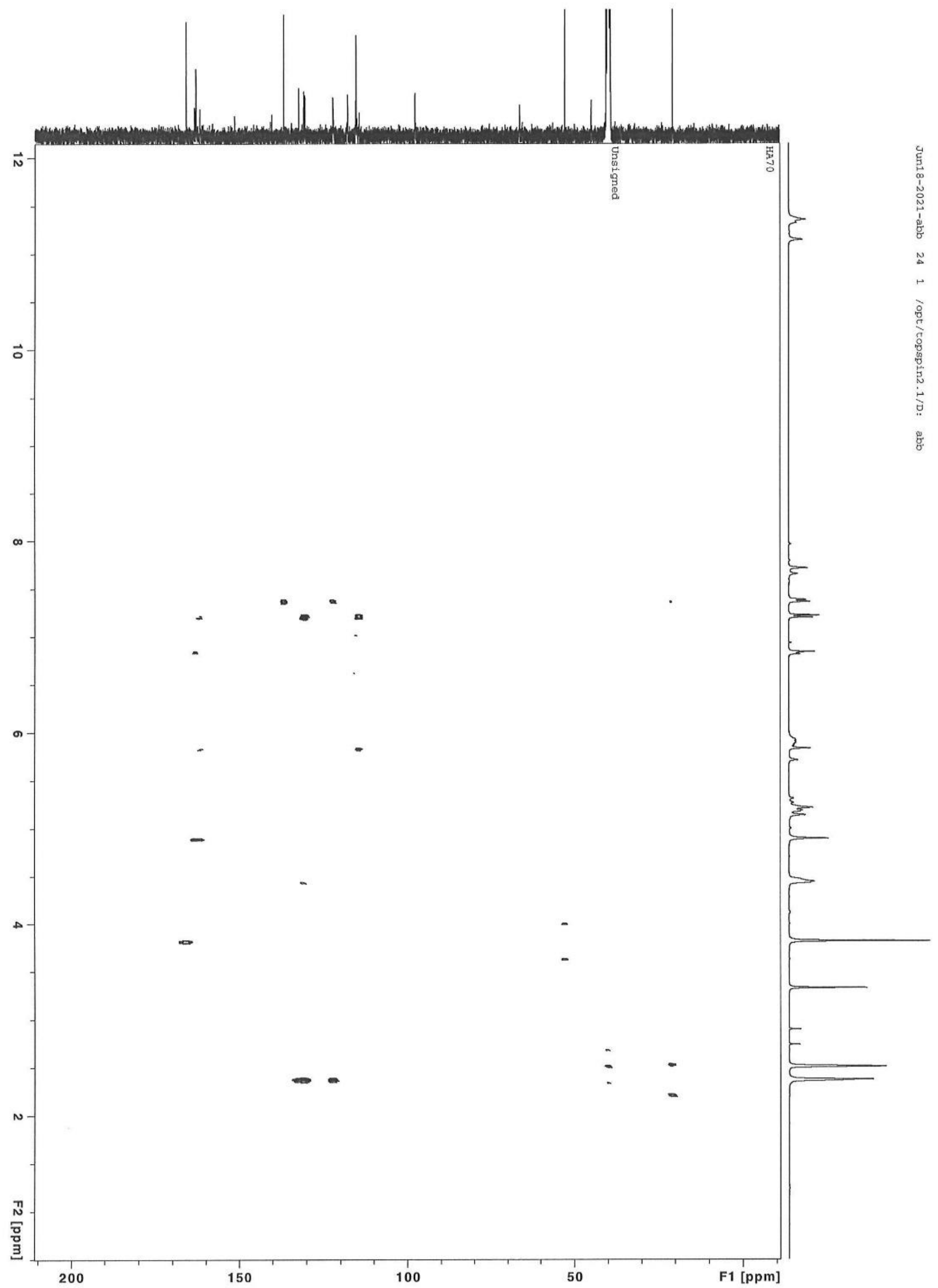

Figure S132. ^{1}H-^{13}C HMBC spectrum of compound **7f.**

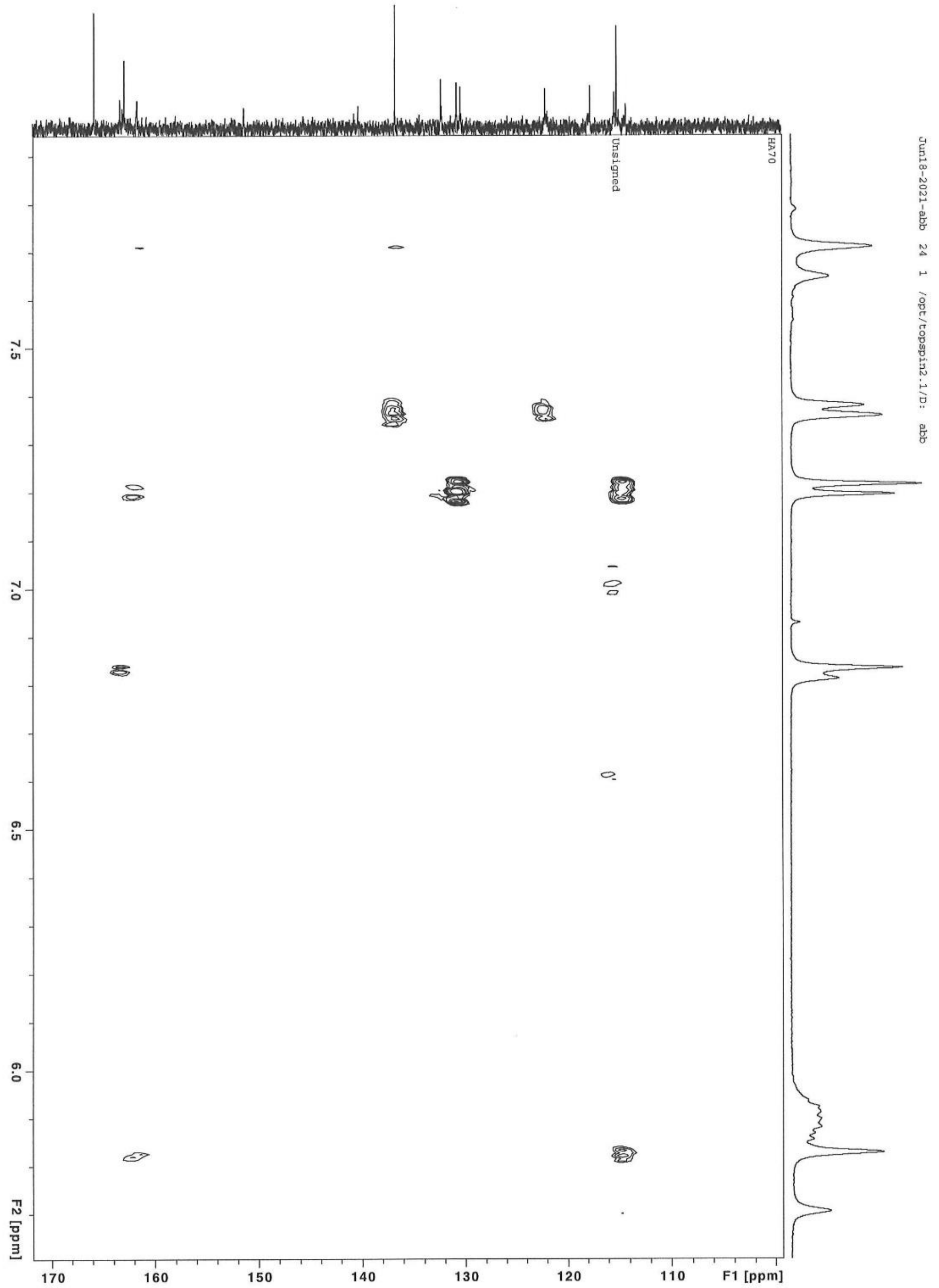

Figure S133. Detail of ^{1}H-^{13}C HMBC spectrum of compound **7f.**

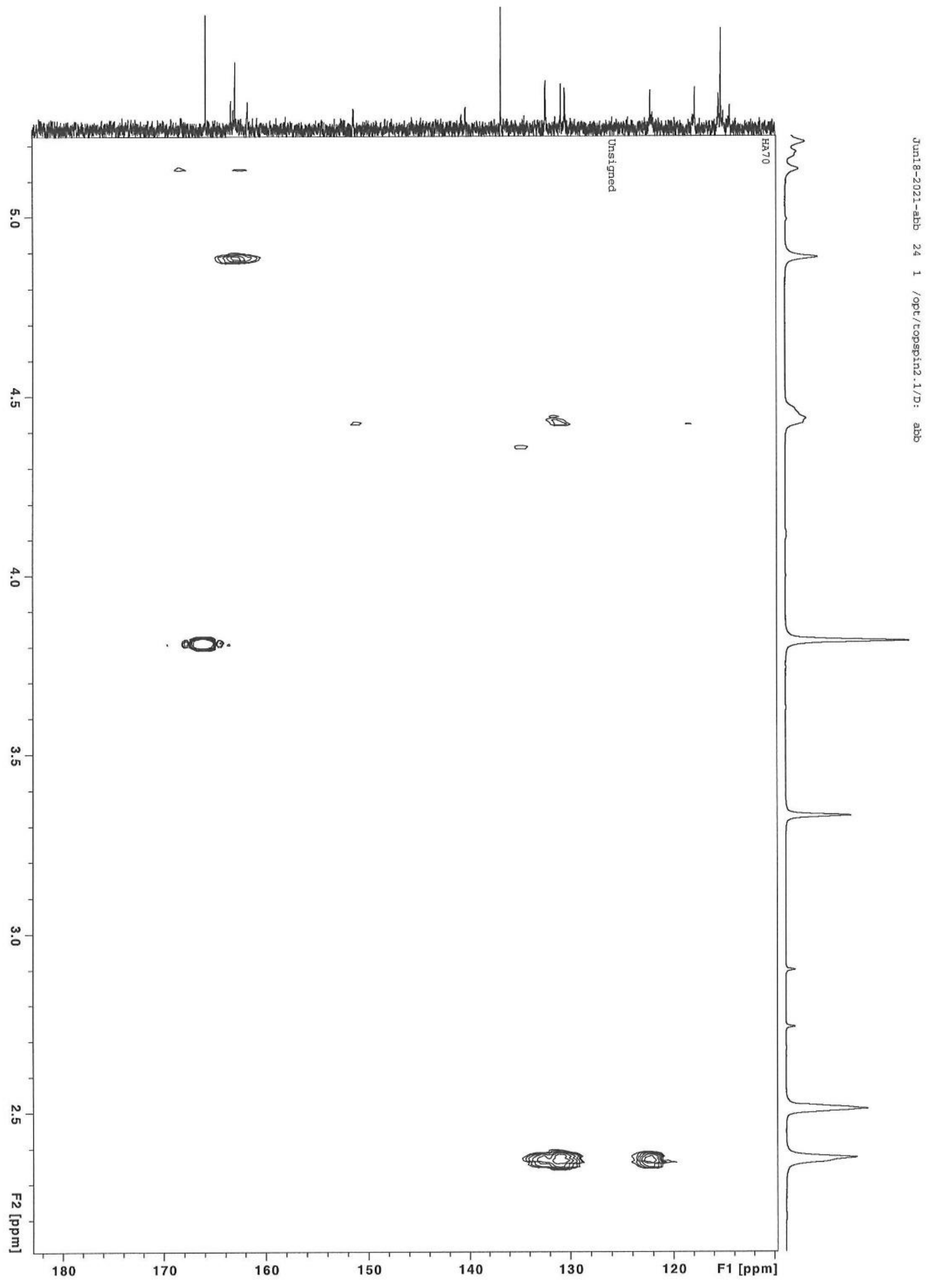

Figure S134. Detail of ^{1}H-^{13}C HMBC spectrum of compound **7f.**

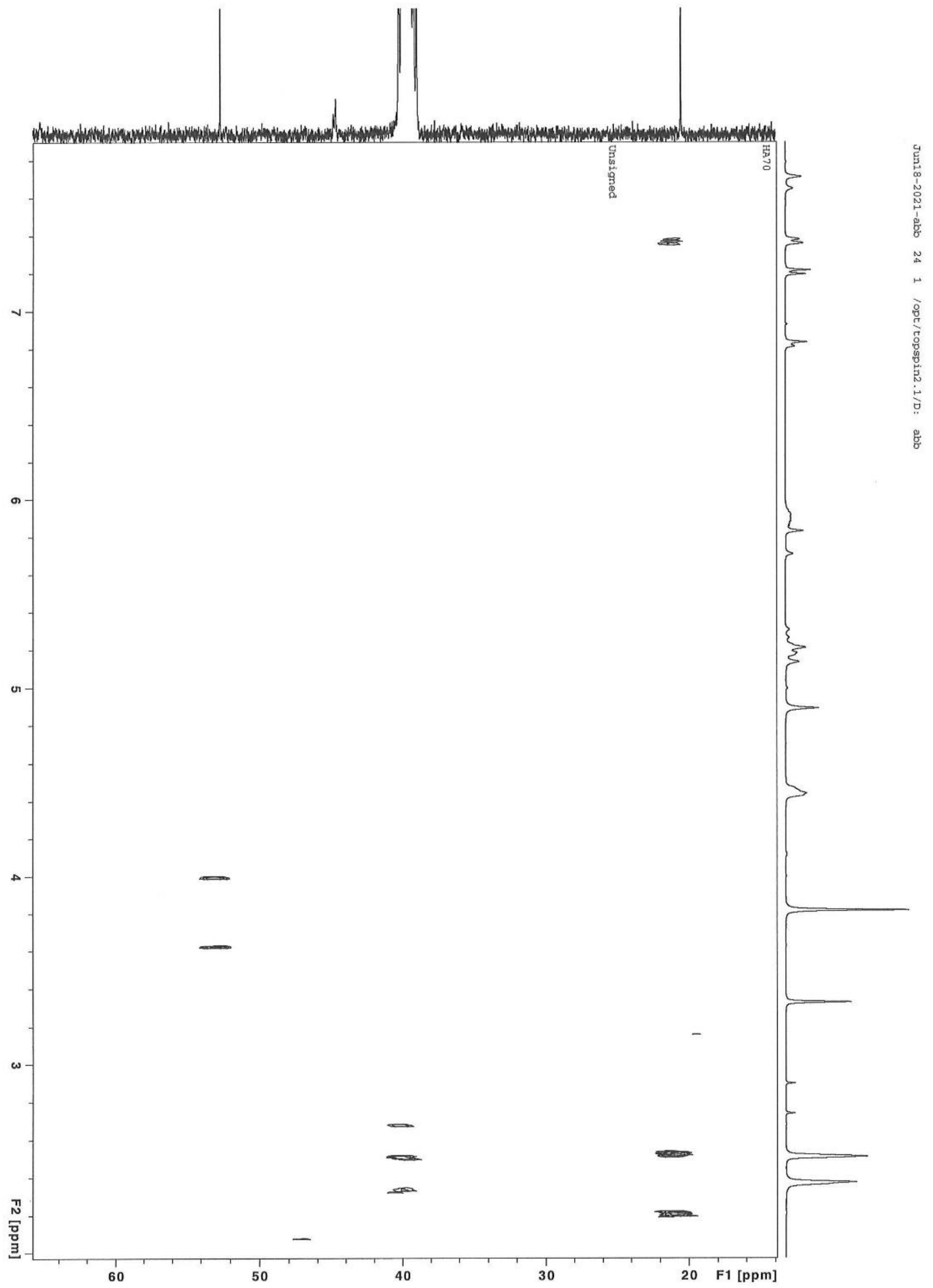

Figure S135. Detail of ^{1}H-^{13}C HMBC spectrum of compound **7f.**

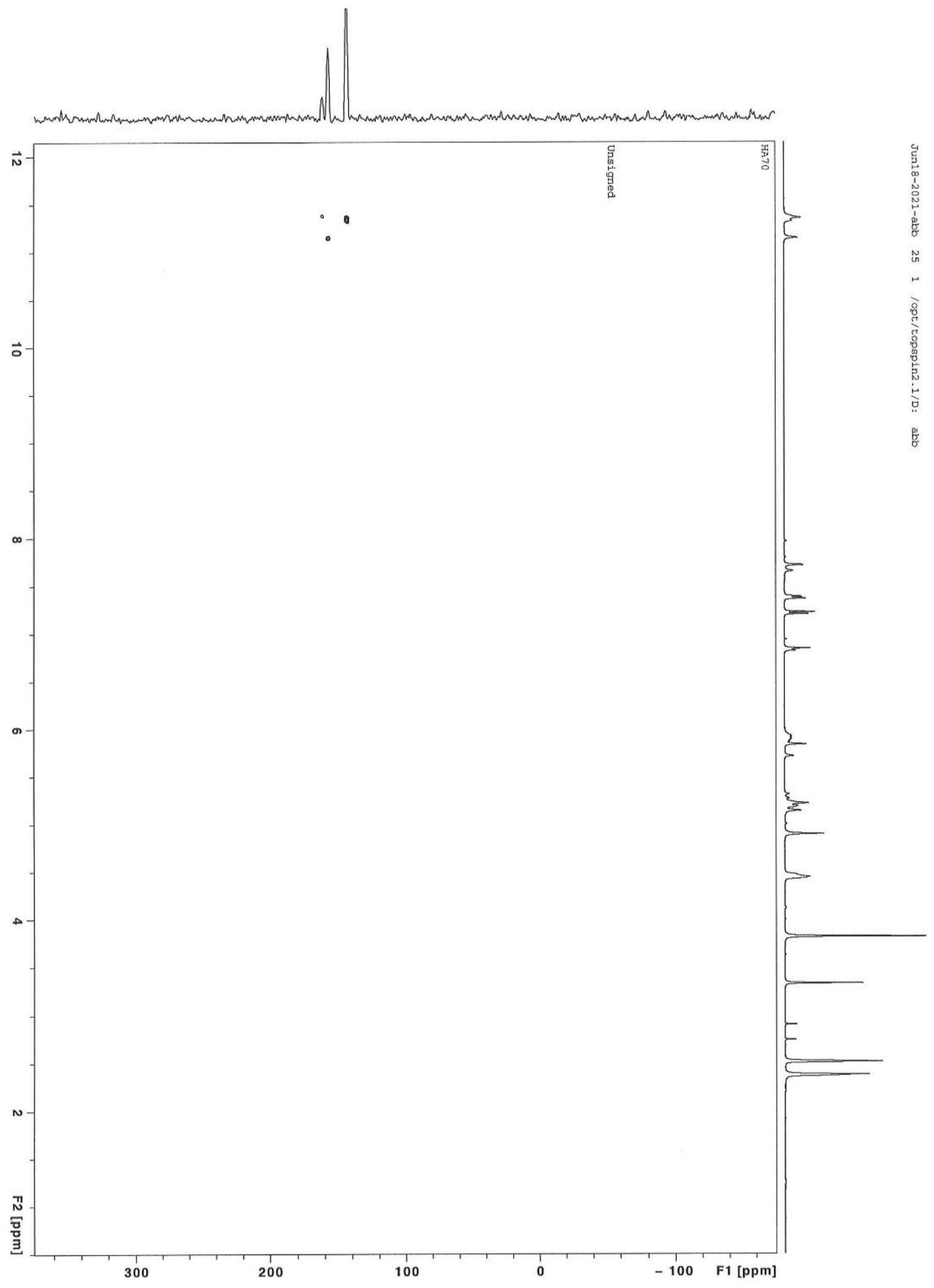

Figure S136. ^{1}H-^{15}N HSQC spectrum of compound **7f.**

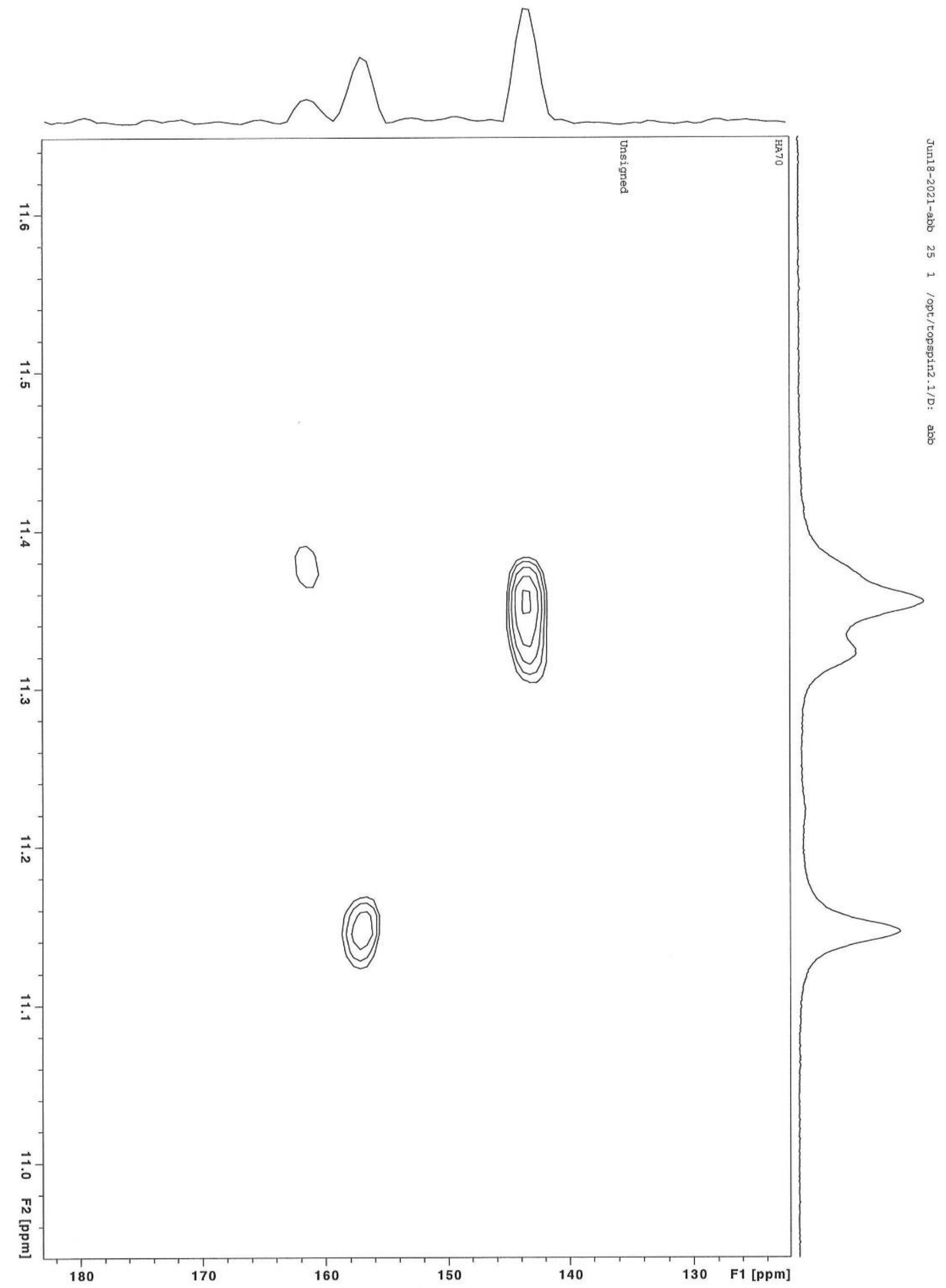

Figure S137. Detail of ^{1}H-^{15}N HSQC spectrum of compound **7f.**

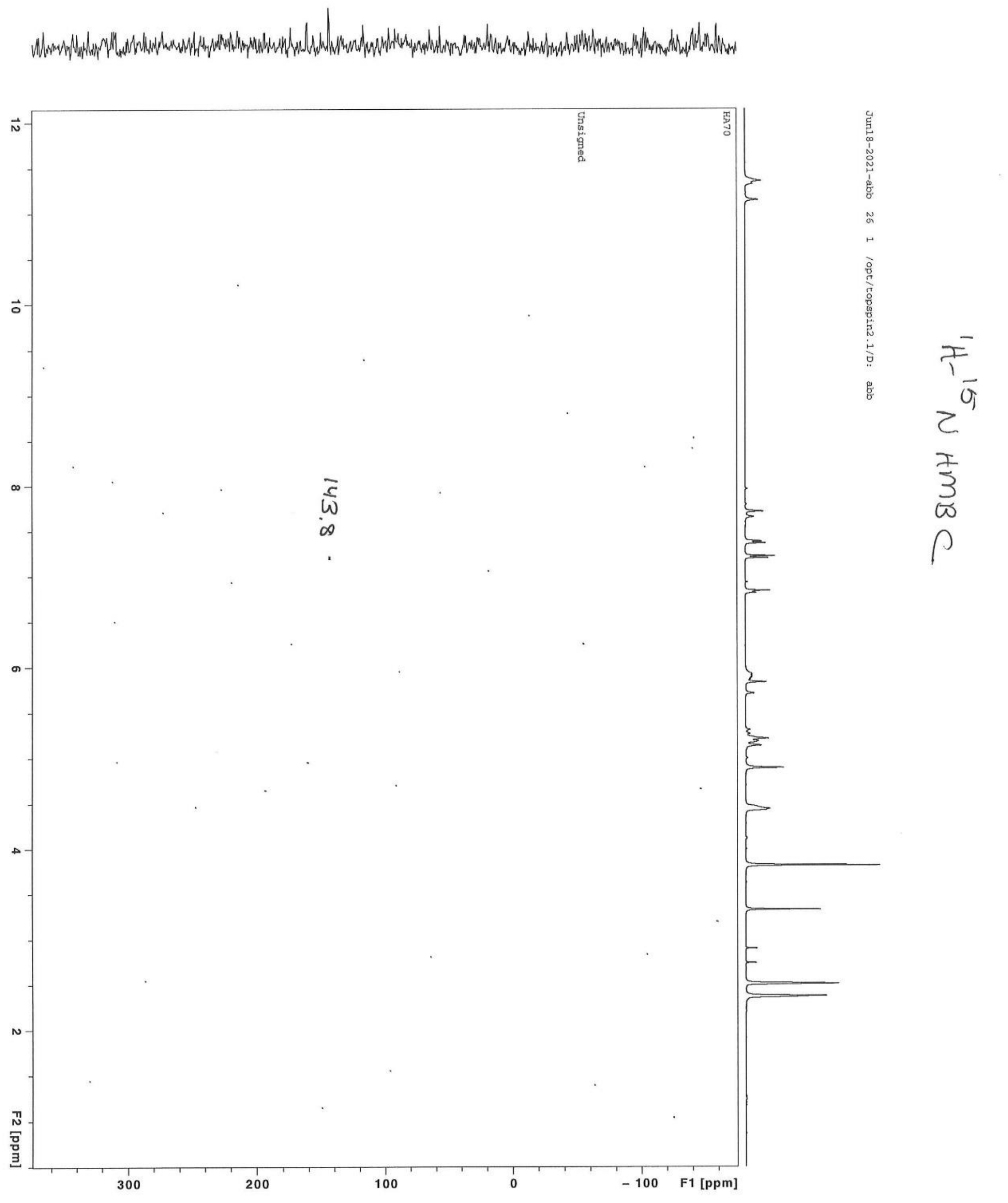

Figure S138. ^{1}H-^{15}N HMBC spectrum of compound **7f.**

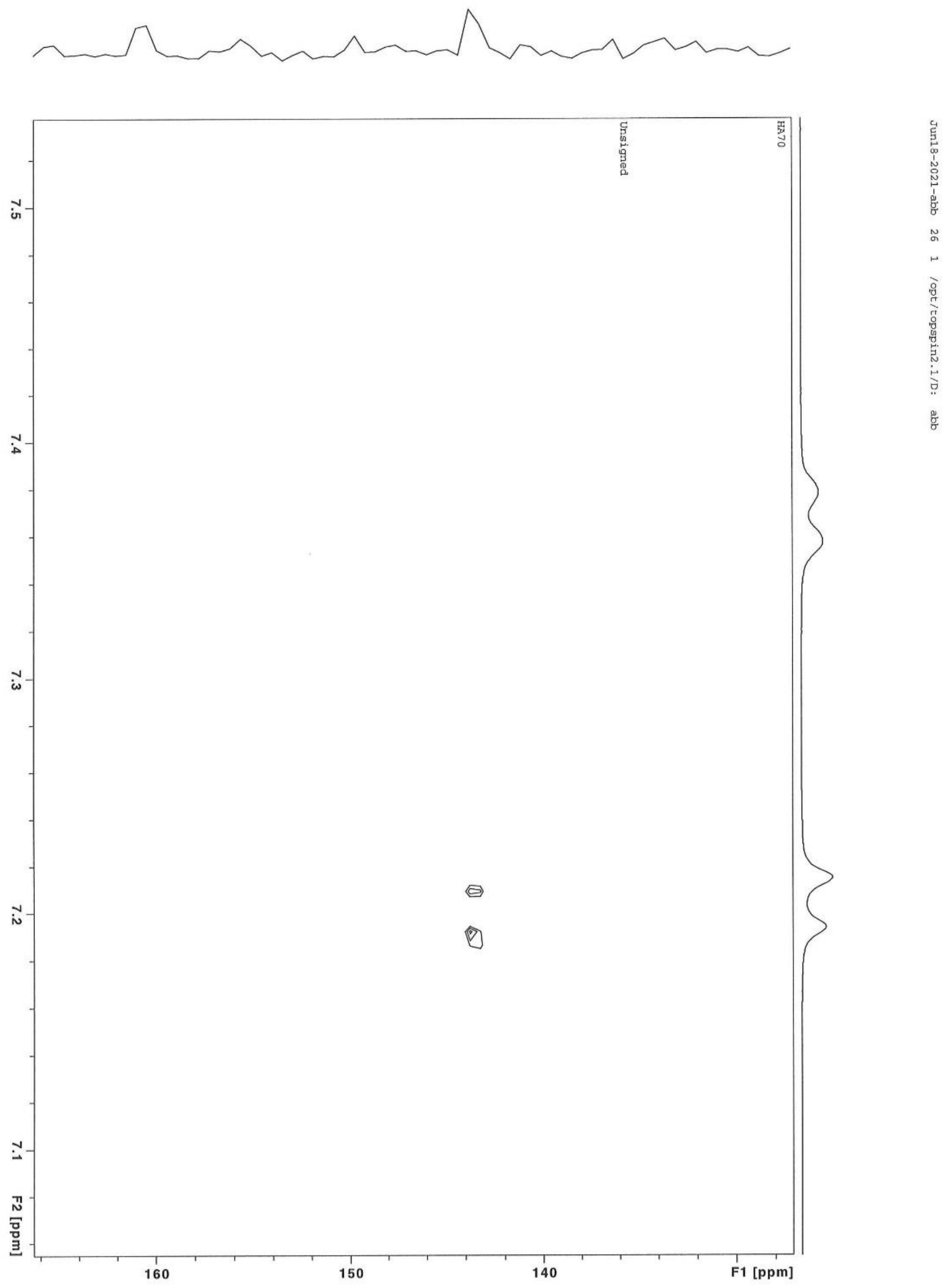

Figure S139. Detail of ^{1}H-^{15}N HMBC spectrum of compound **7f.**

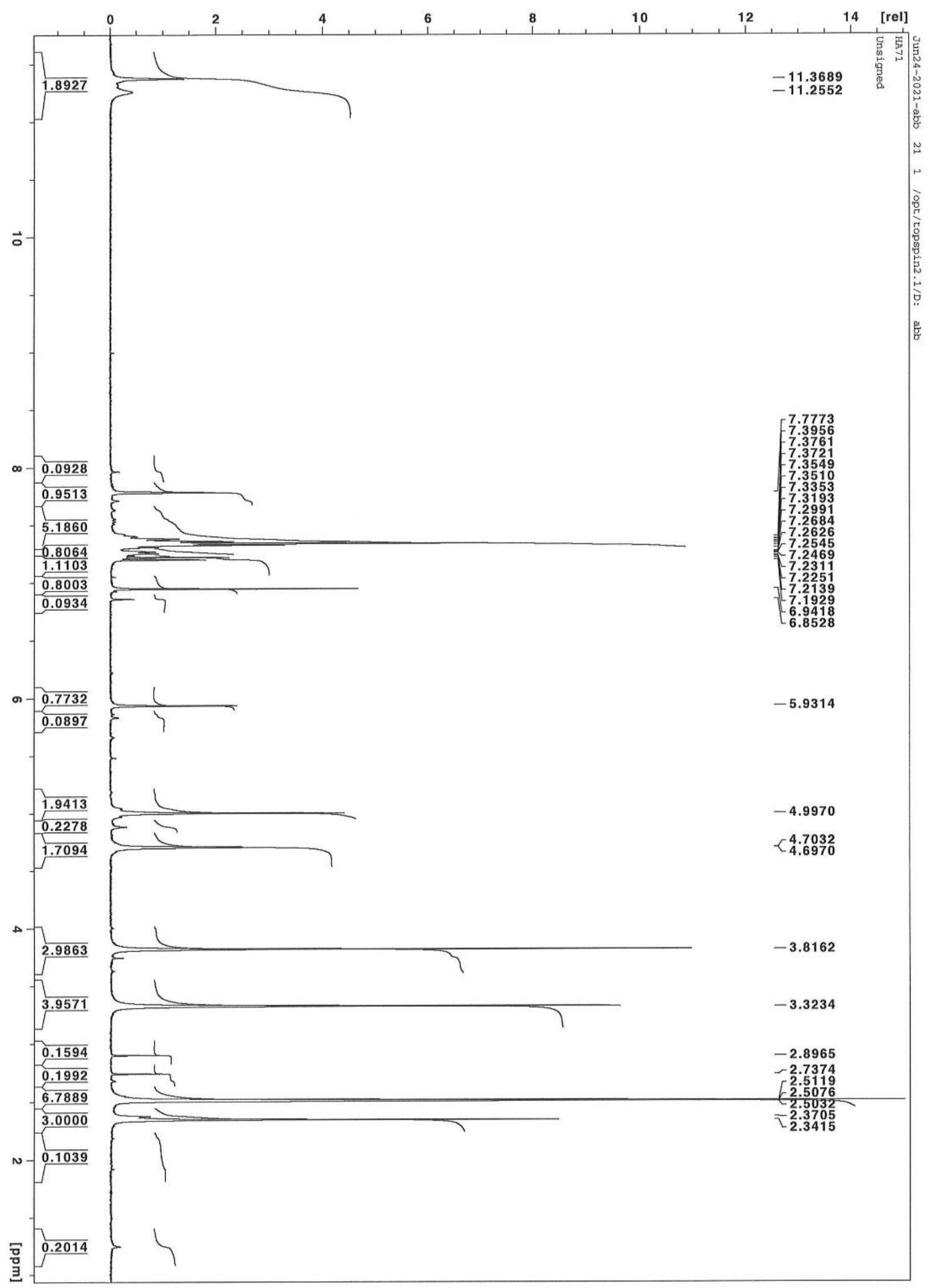

Figure S140. ^{1}H NMR spectrum of compound **7g** (400 MHz, DMSO-d_6).

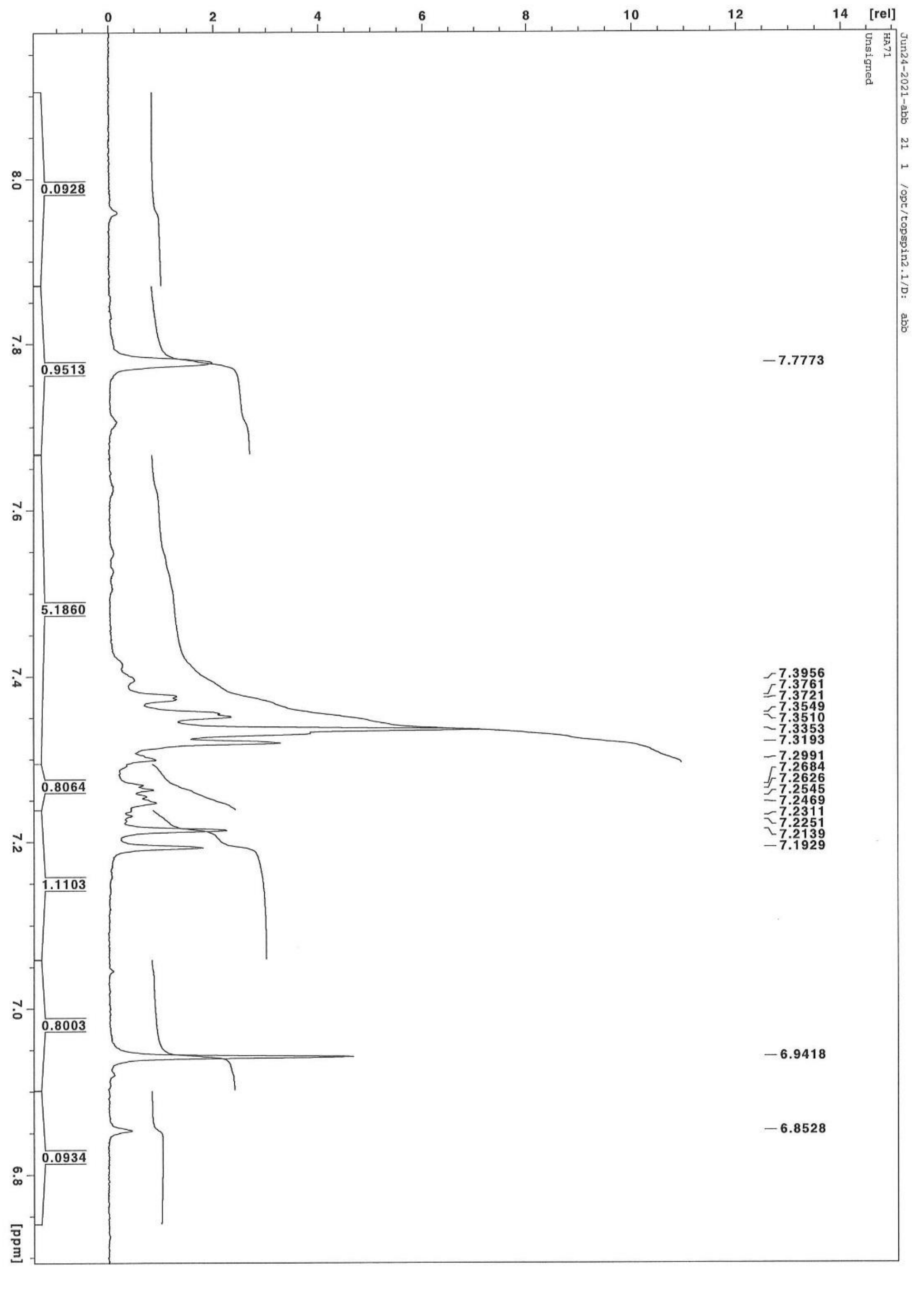

Figure S141. Detail of ^{1}H NMR spectrum of compound **7g.**

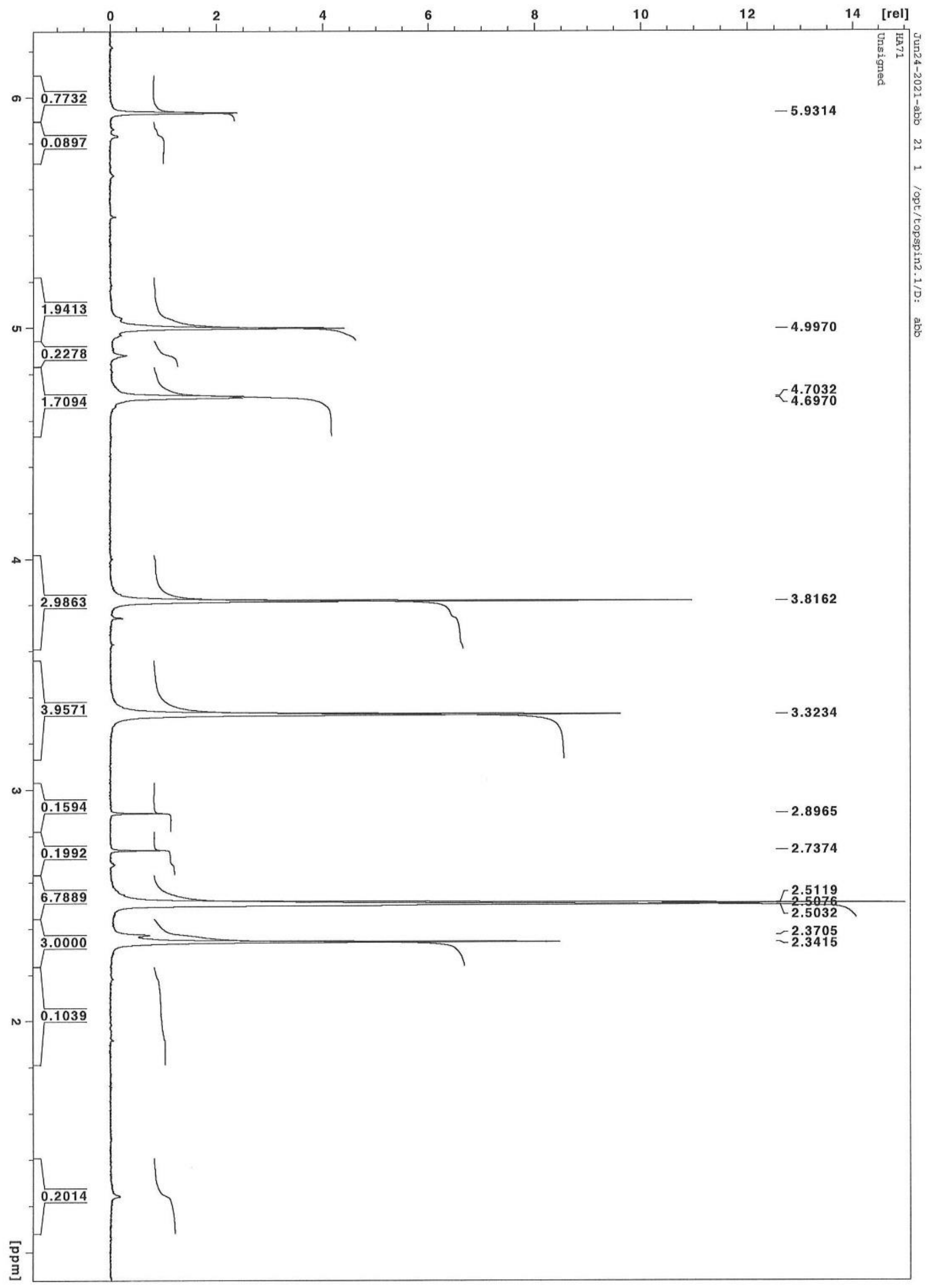

Figure S142. Detail of ^{1}H NMR spectrum of compound **7g.**

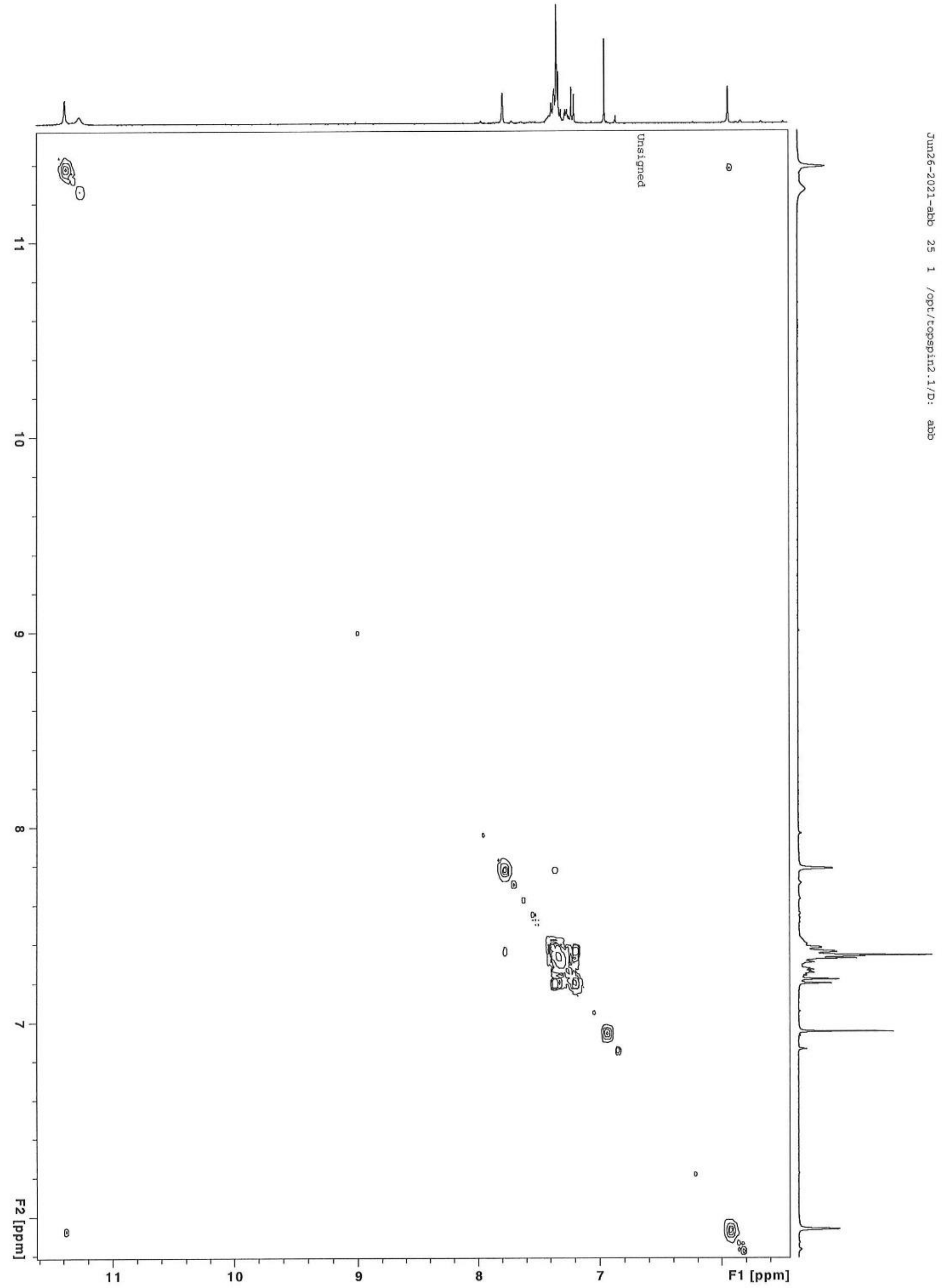

Figure S143. ^{1}H-^{1}H COSY spectrum of compound **7g.**

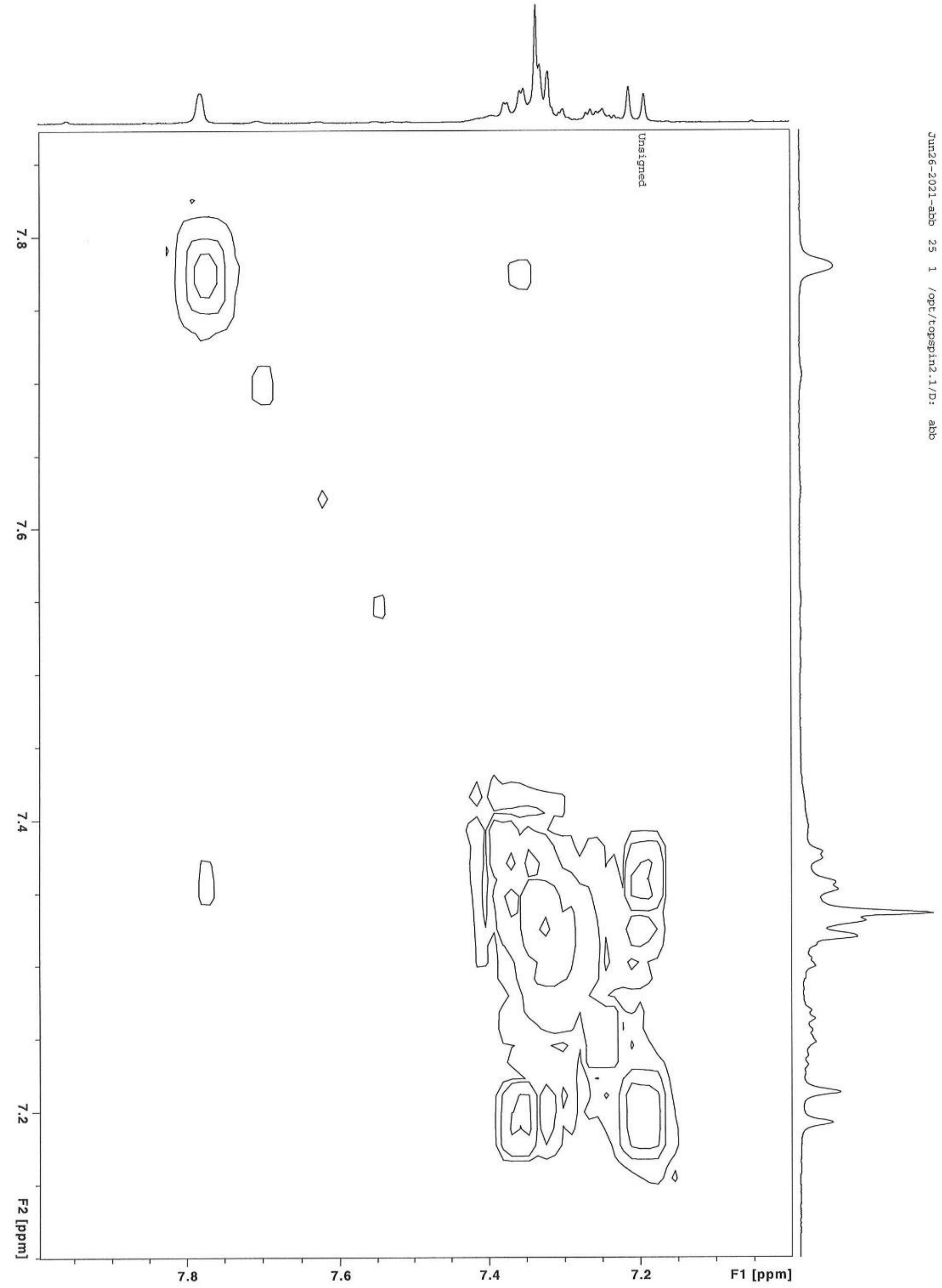

Figure S144. Detail of ^{1}H-^{1}H COSY spectrum of compound **7g.**

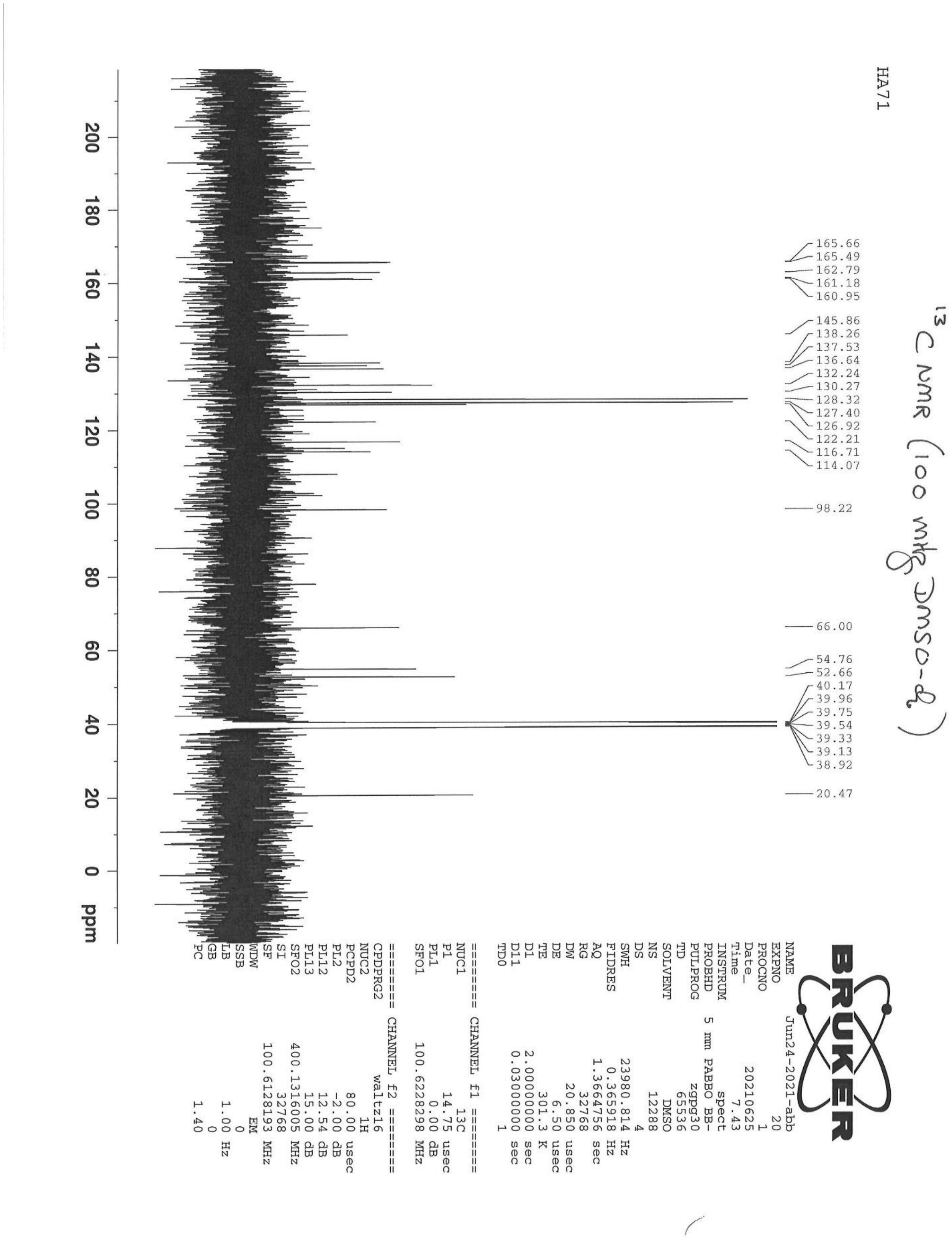

Figure S145. ^{13}C NMR spectrum of compound **7g** (100 MHz, DMSO-d_6).

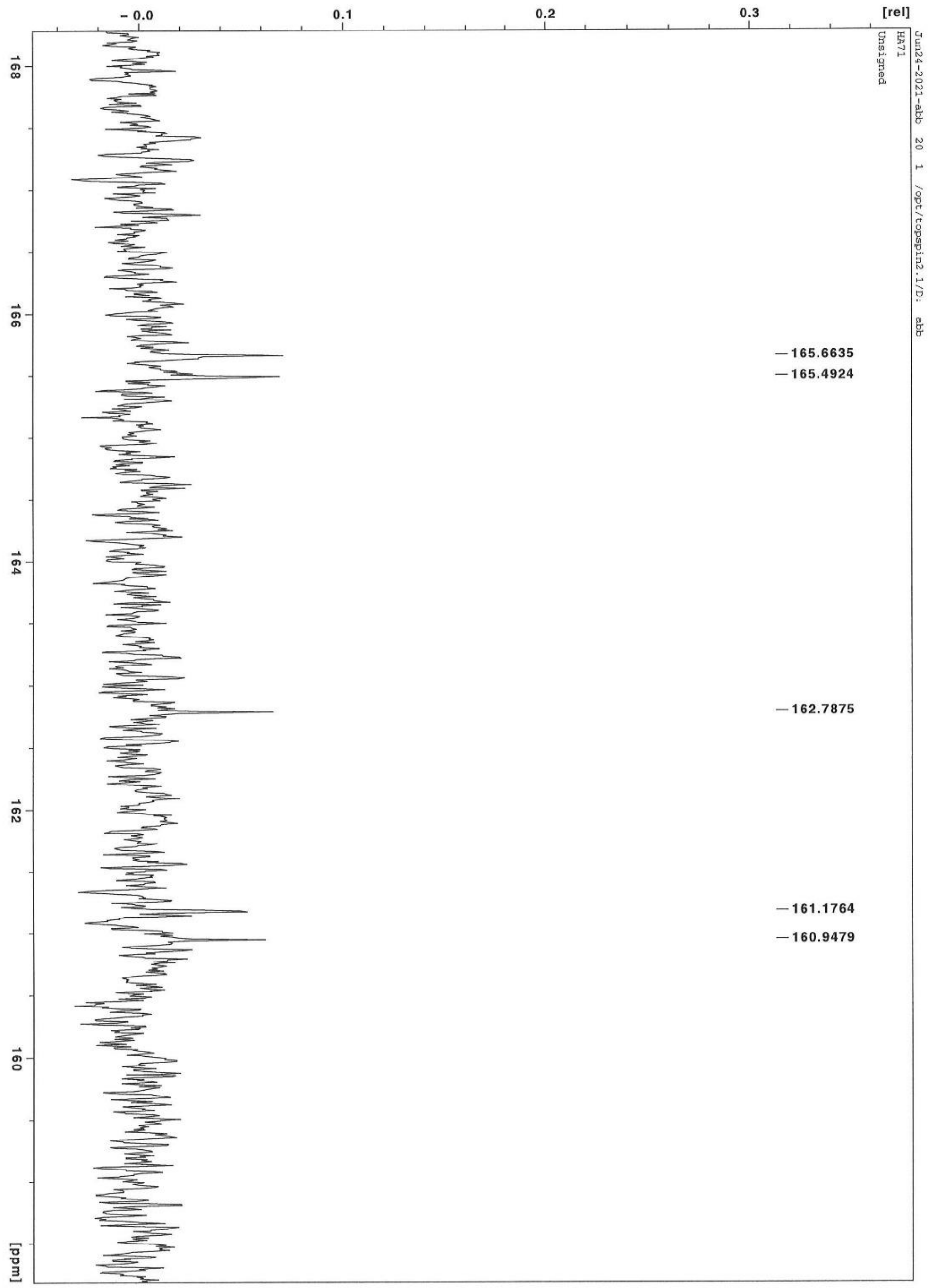

Figure S146. Detail of ^{13}C NMR spectrum of compound **7g.**

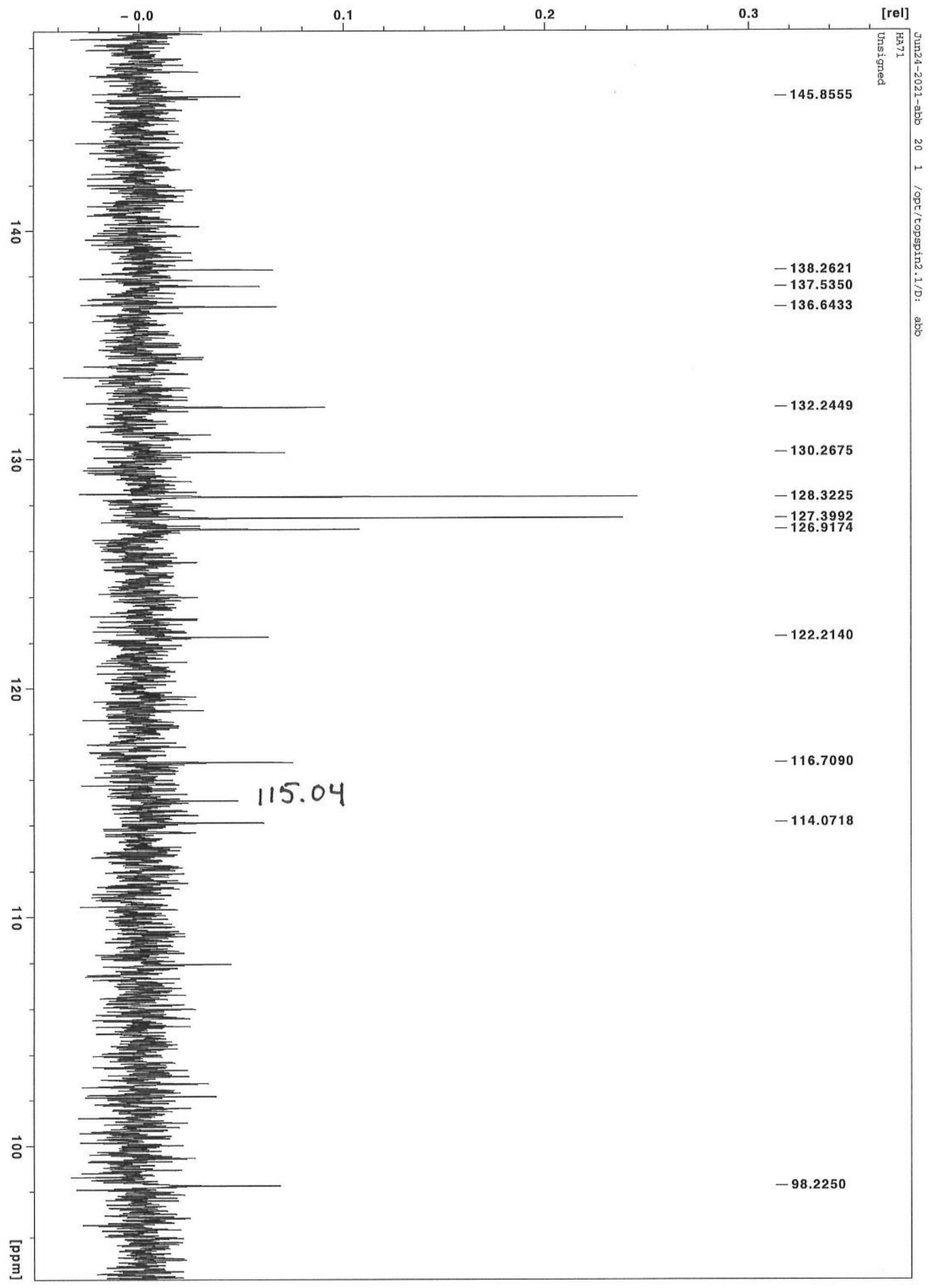

Figure S147. Detail of ^{13}C NMR spectrum of compound **7g.**

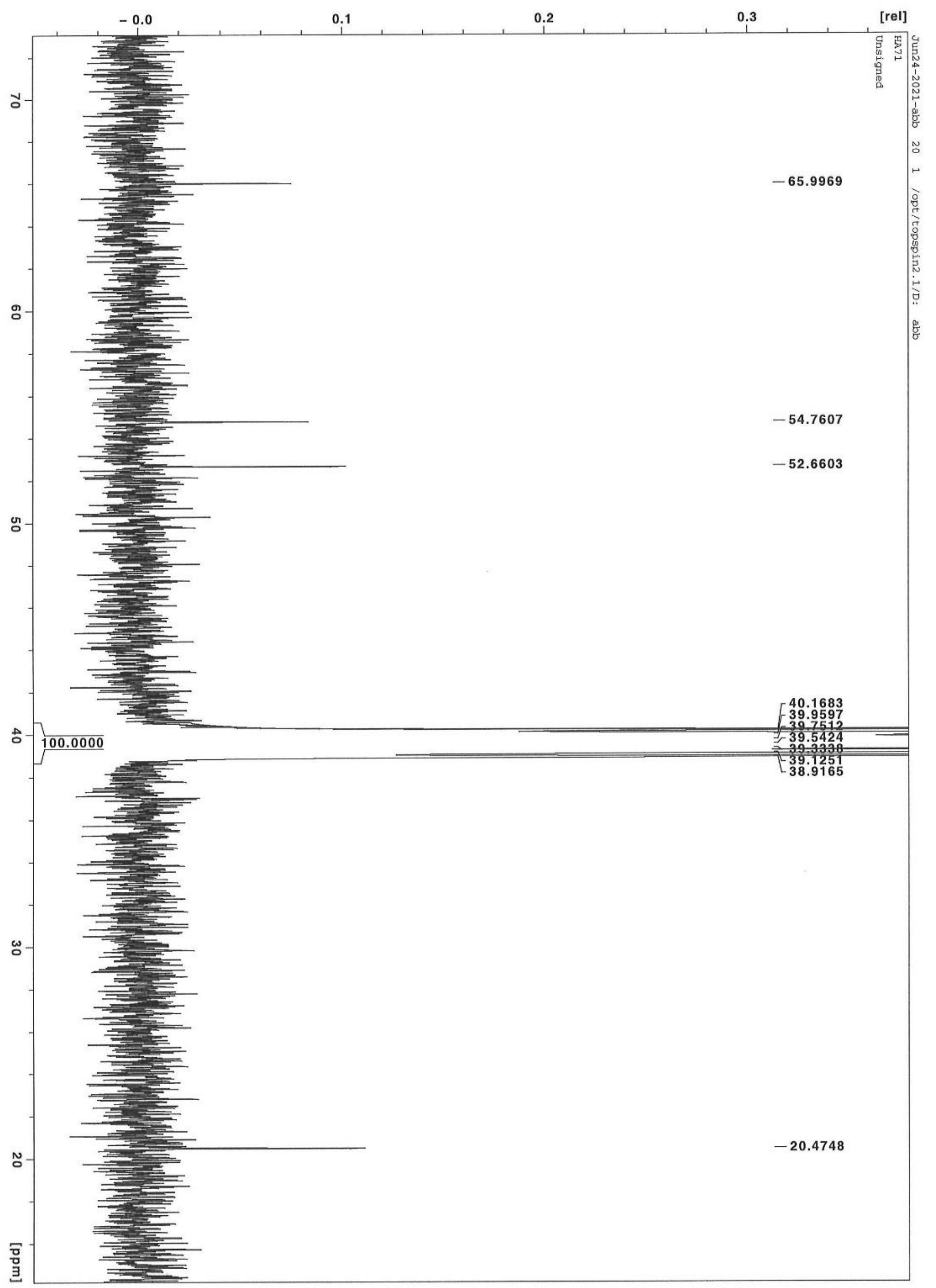

Figure S148. Detail of ^{13}C NMR spectrum of compound **7g.**

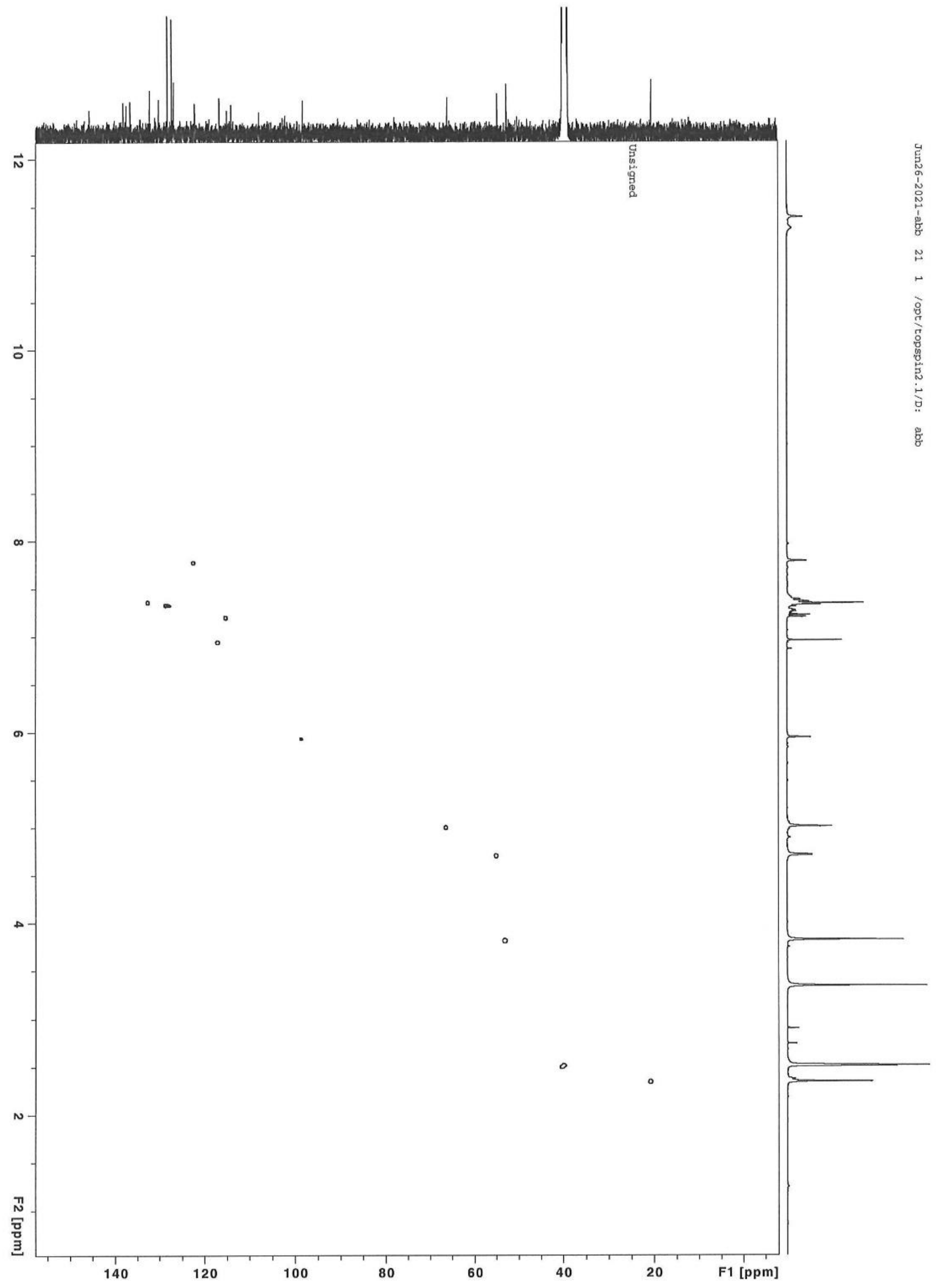

Figure S149. ^{1}H-^{13}C HSQC spectrum of compound **7g.**

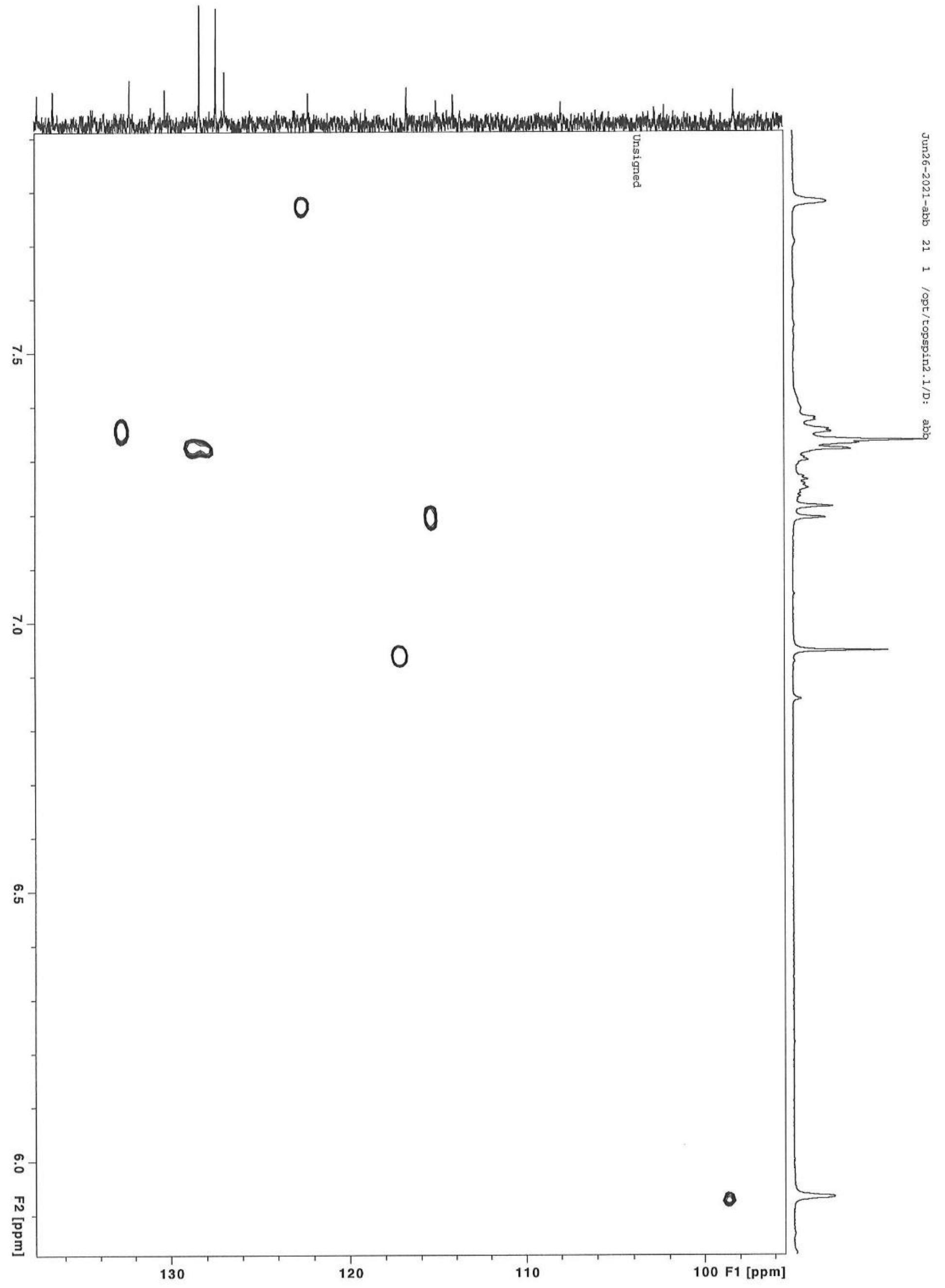

Figure S150. Detail of ^{1}H-^{13}C HSQC spectrum of compound **7g.**

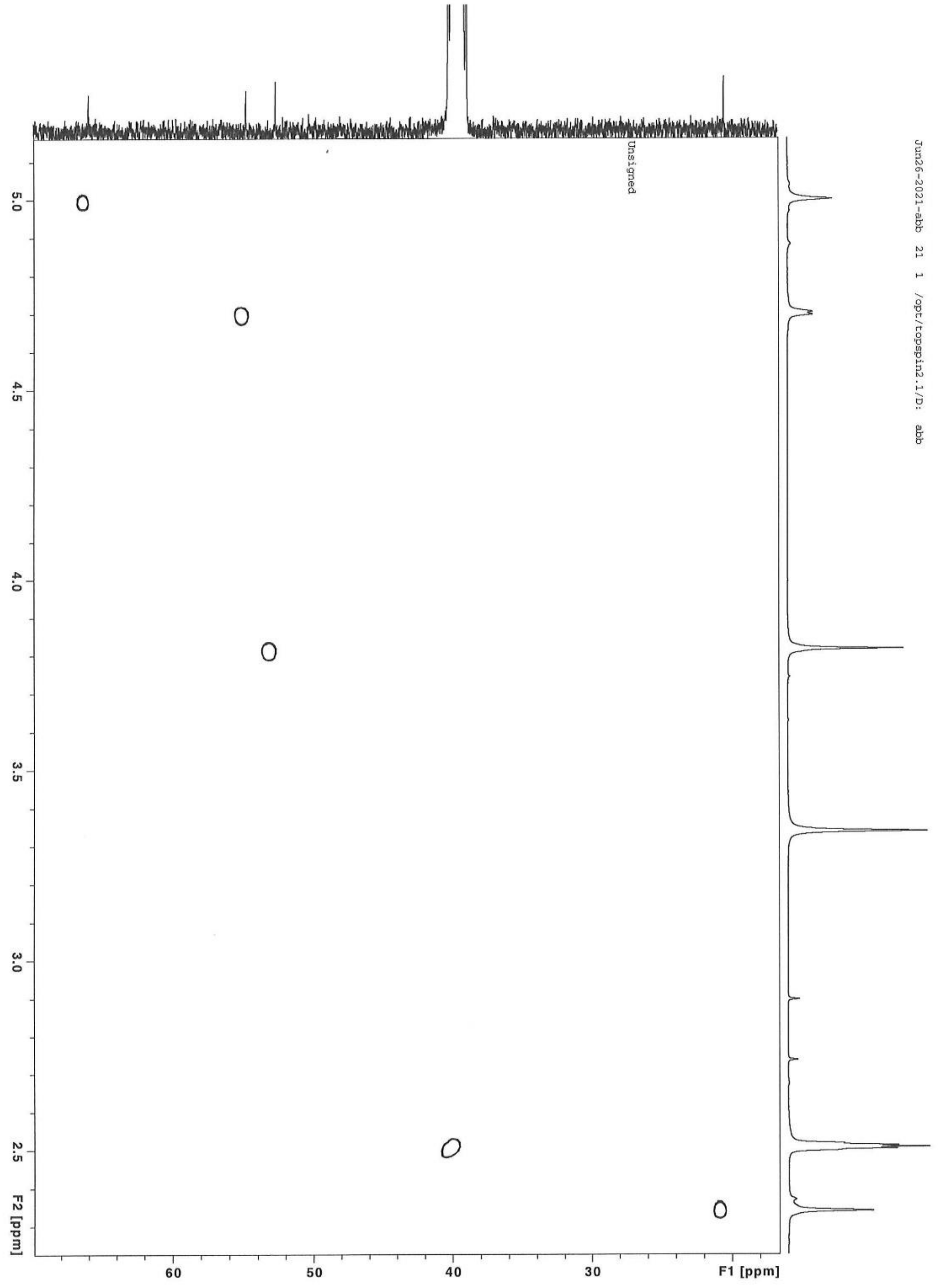

Figure S151. Detail of ^{1}H-^{13}C HSQC spectrum of compound **7g.**

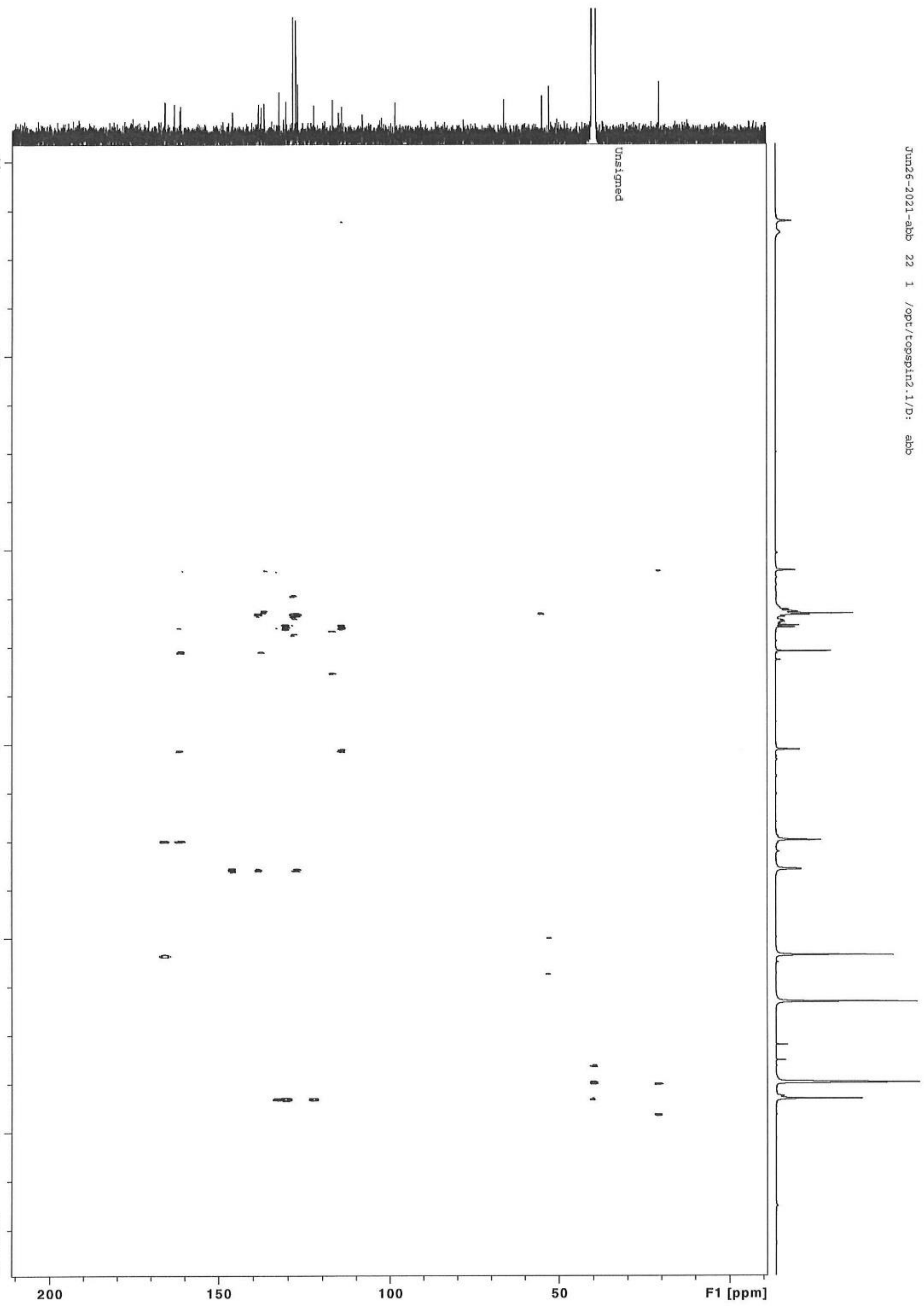

Figure S152. ^{1}H-^{13}C HMBC spectrum of compound **7g.**

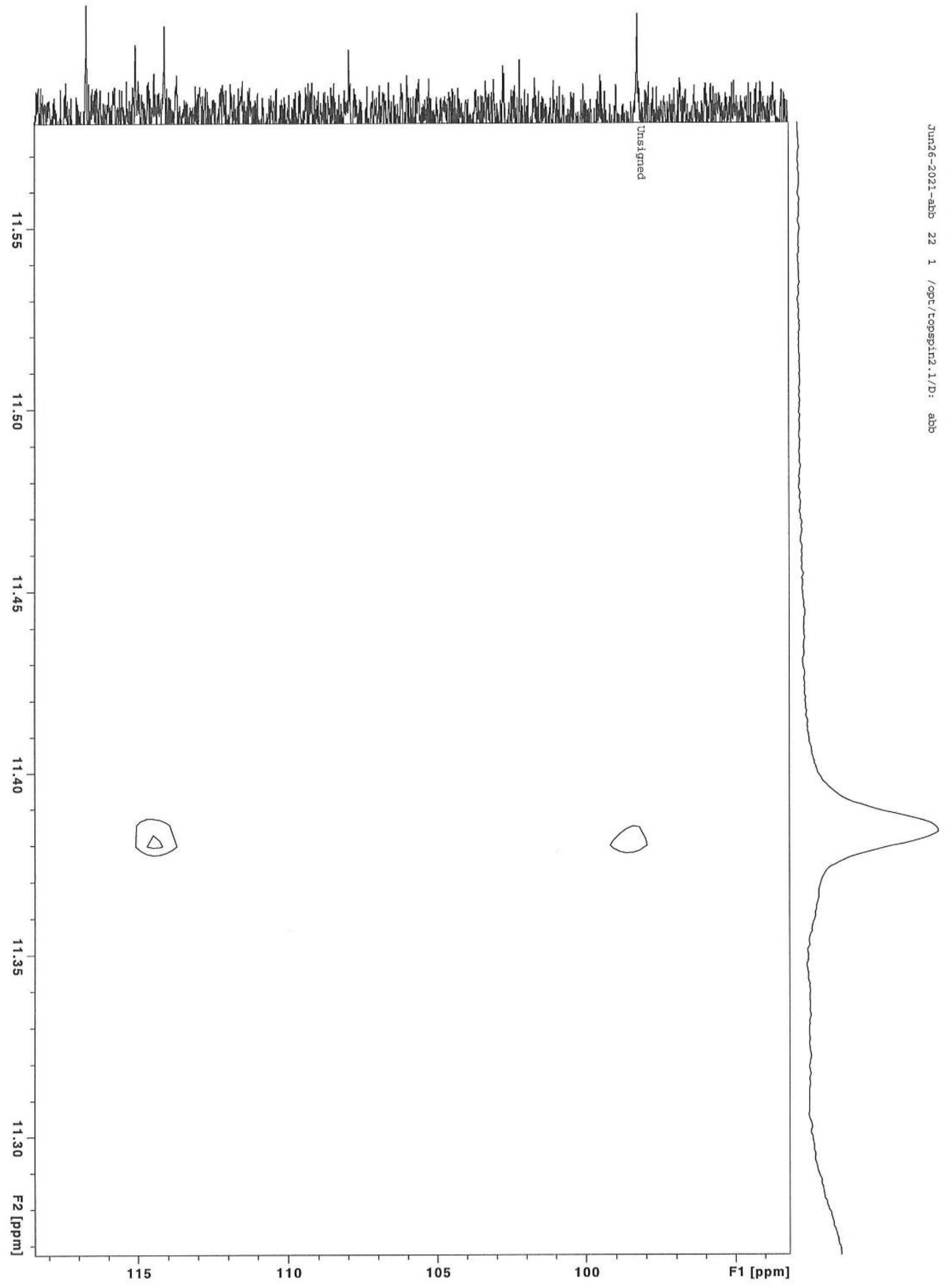

Figure S153. Detail of ^{1}H-^{13}C HMBC spectrum of compound **7g.**

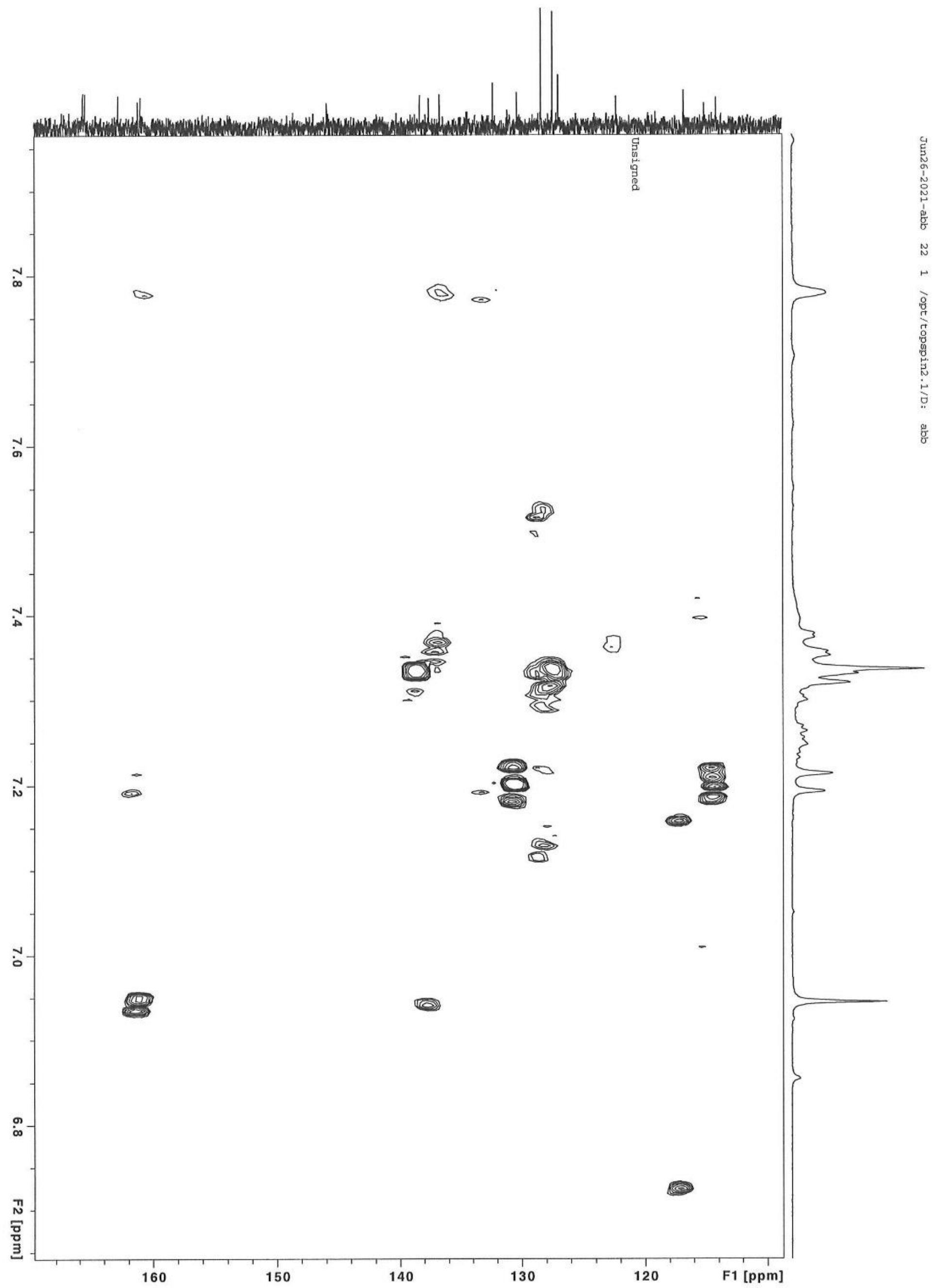

Figure S154. Detail of ^{1}H-^{13}C HMBC spectrum of compound **7g.**

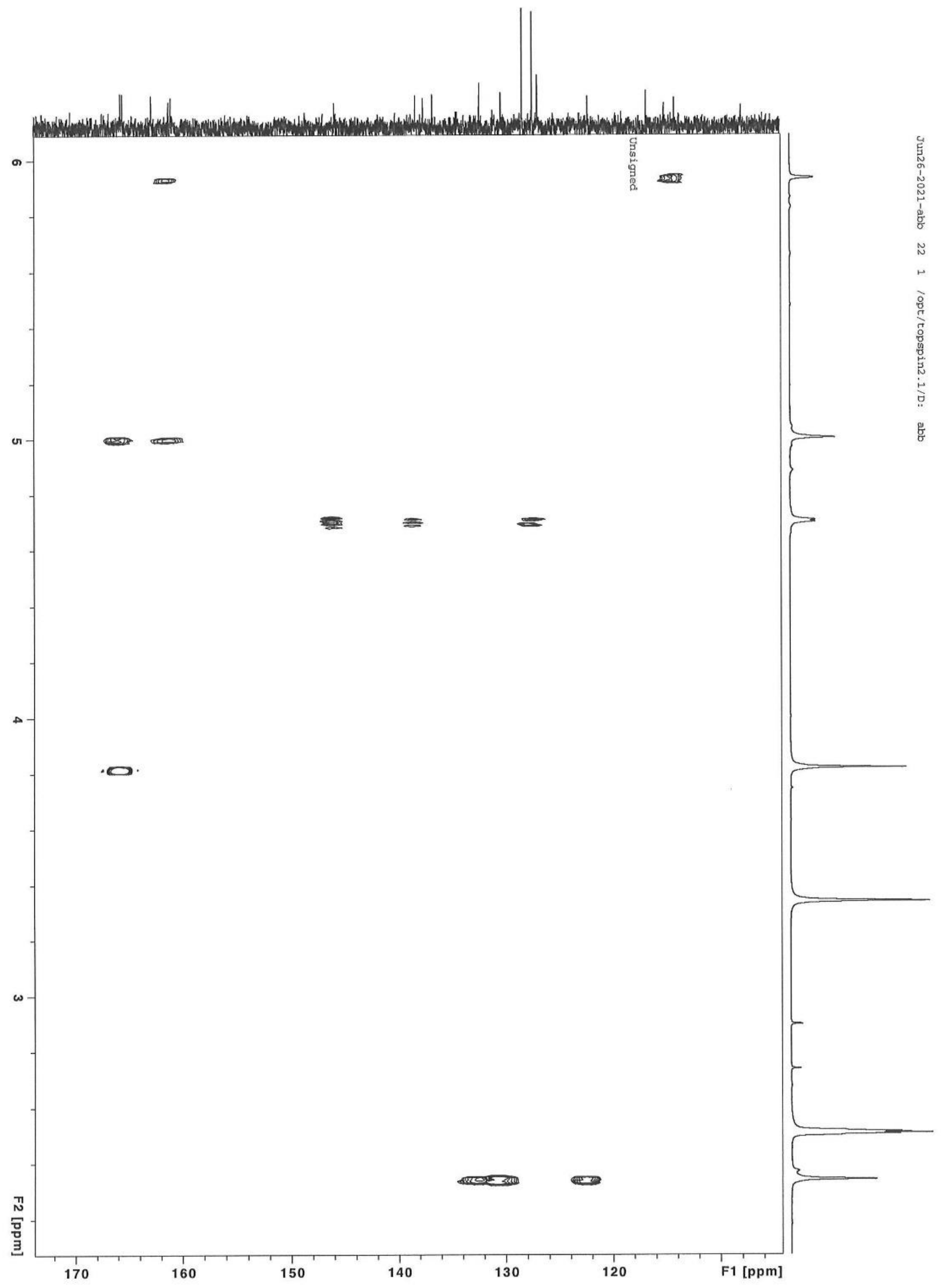

Figure S155. Detail of ^{1}H-^{13}C HMBC spectrum of compound **7g.**

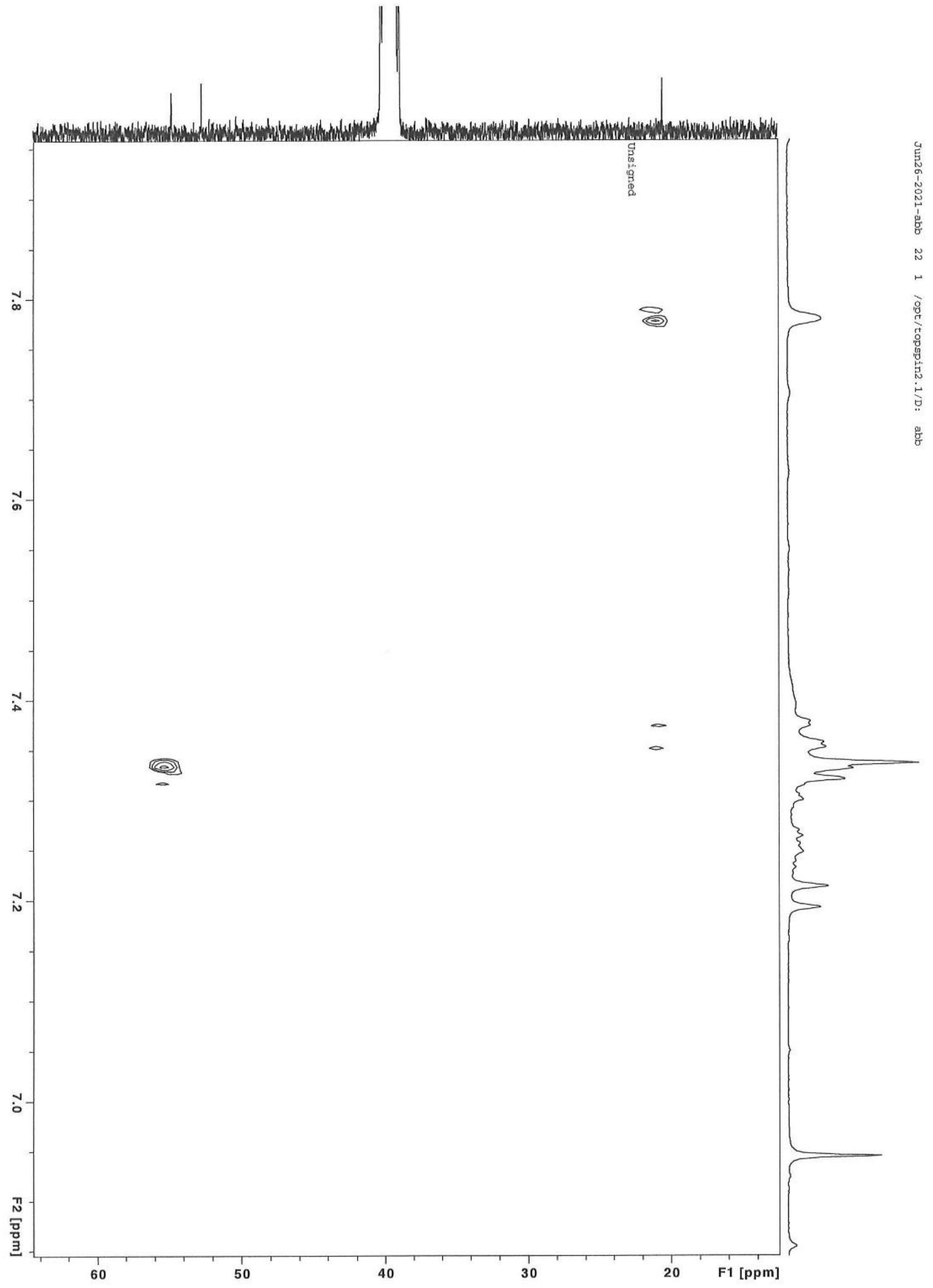

Figure S156. Detail of ^{1}H-^{13}C HMBC spectrum of compound **7g.**

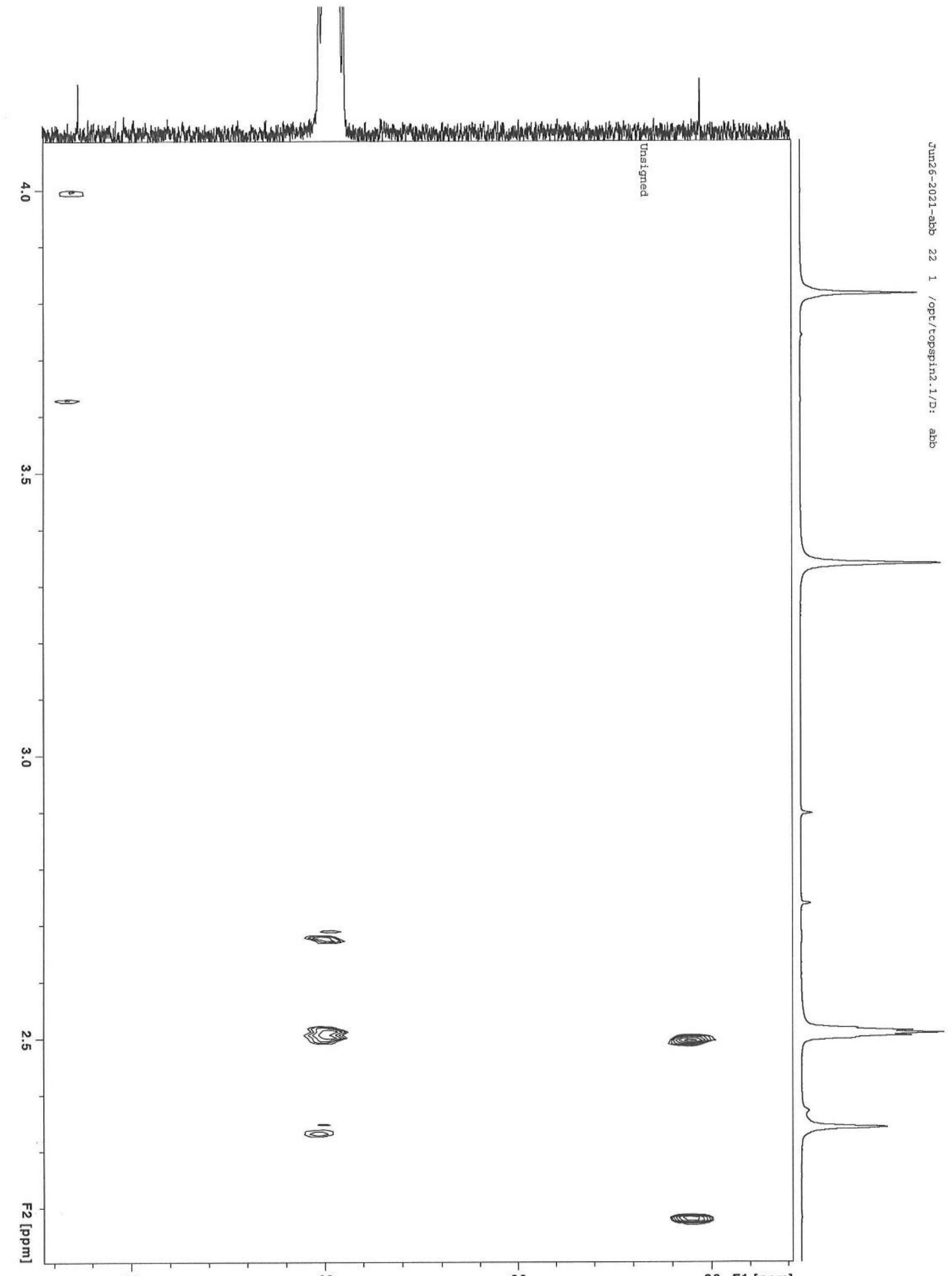

Figure S157. Detail of ^{1}H-^{13}C HMBC spectrum of compound **7g.**

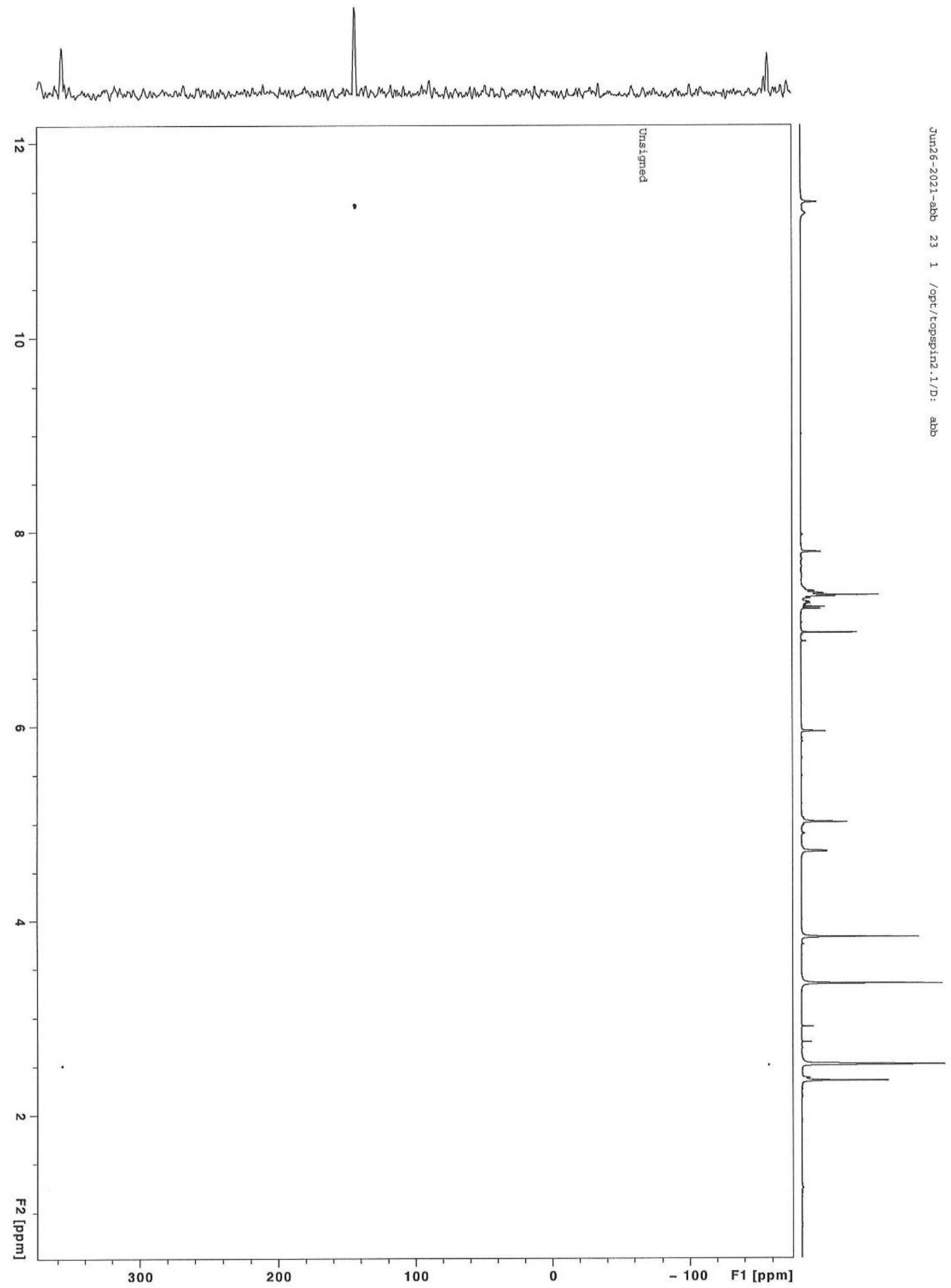

Figure S158. ^{1}H-^{15}N HSQC spectrum of compound **7g** (an HMBC experiment gave no cross peaks).

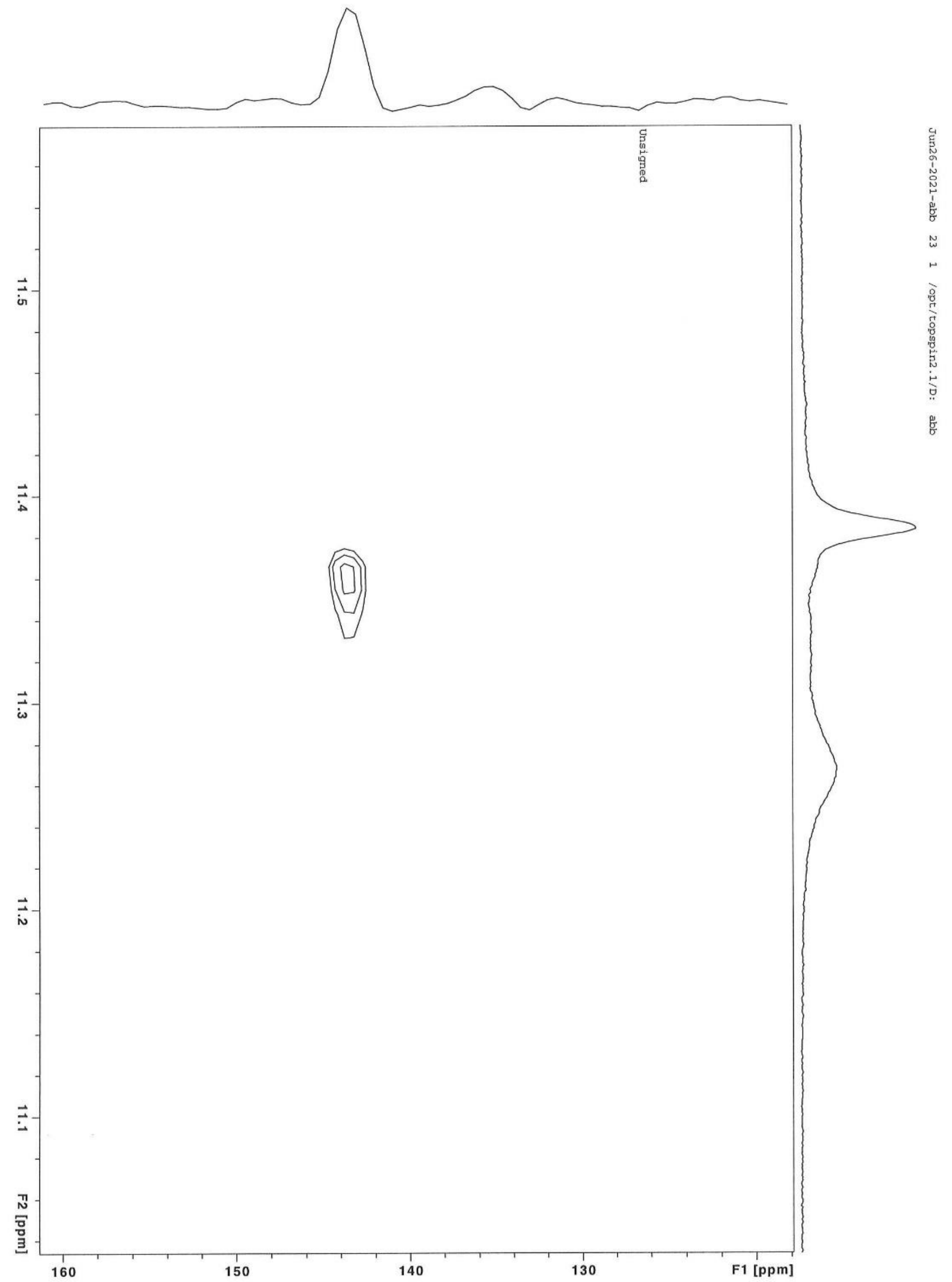

Figure S159. Detail of ^{1}H-^{15}N HSQC spectrum of compound **7g.**

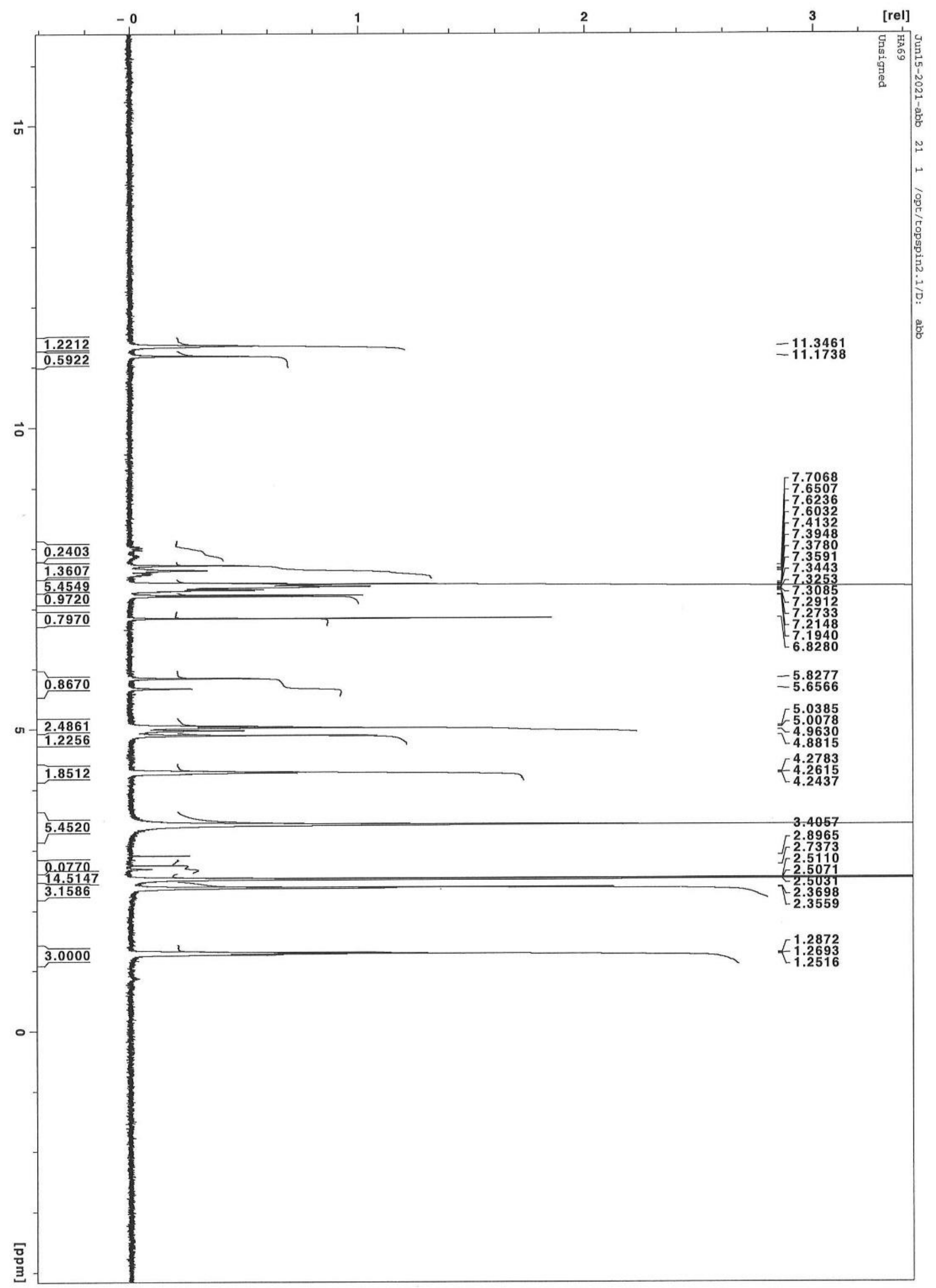

Figure S160. ^{1}H NMR spectrum of compound **7h** (400 MHz, DMSO-d_6).

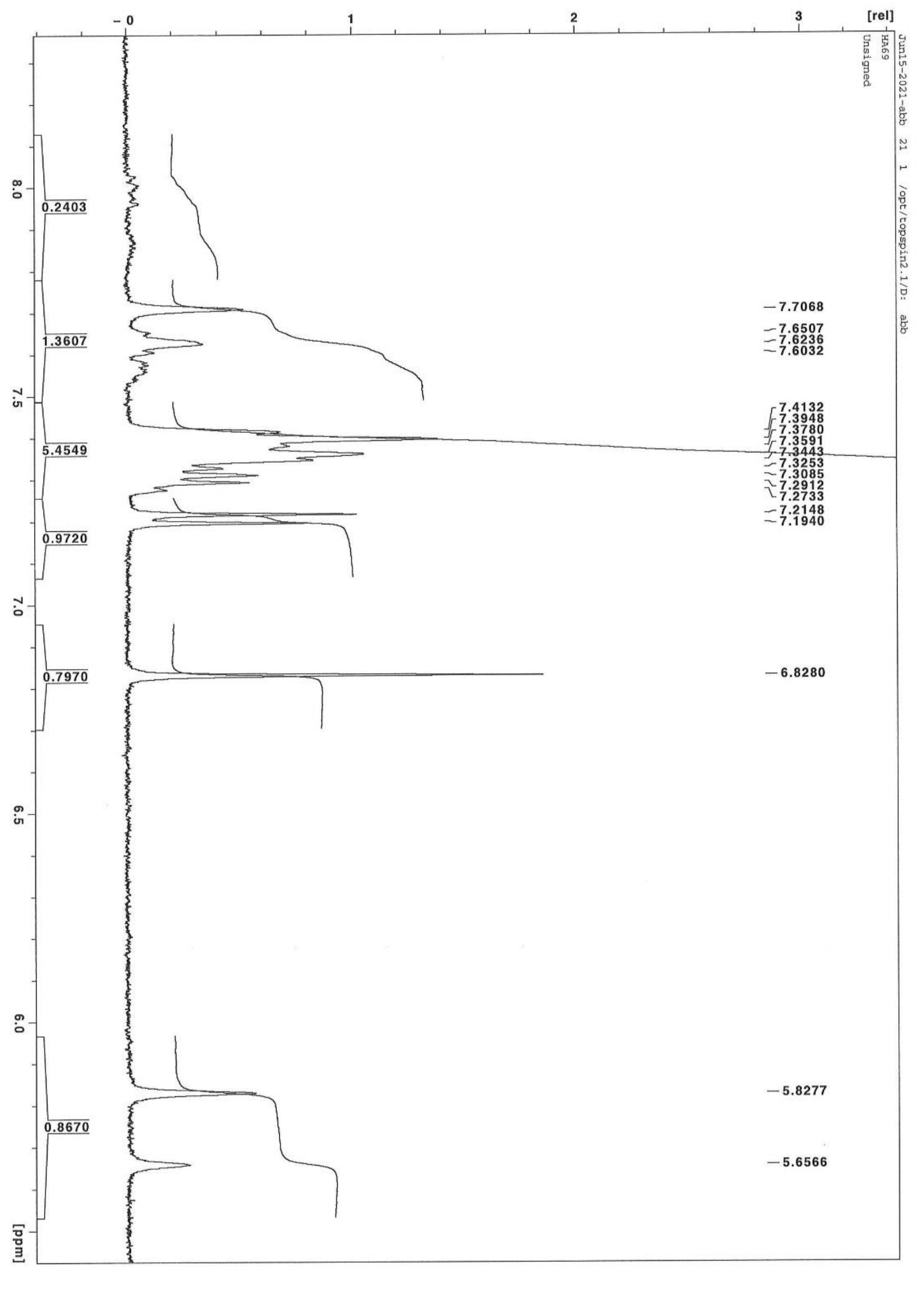

Figure S161. Detail of ^{1}H NMR spectrum of compound **7h.**

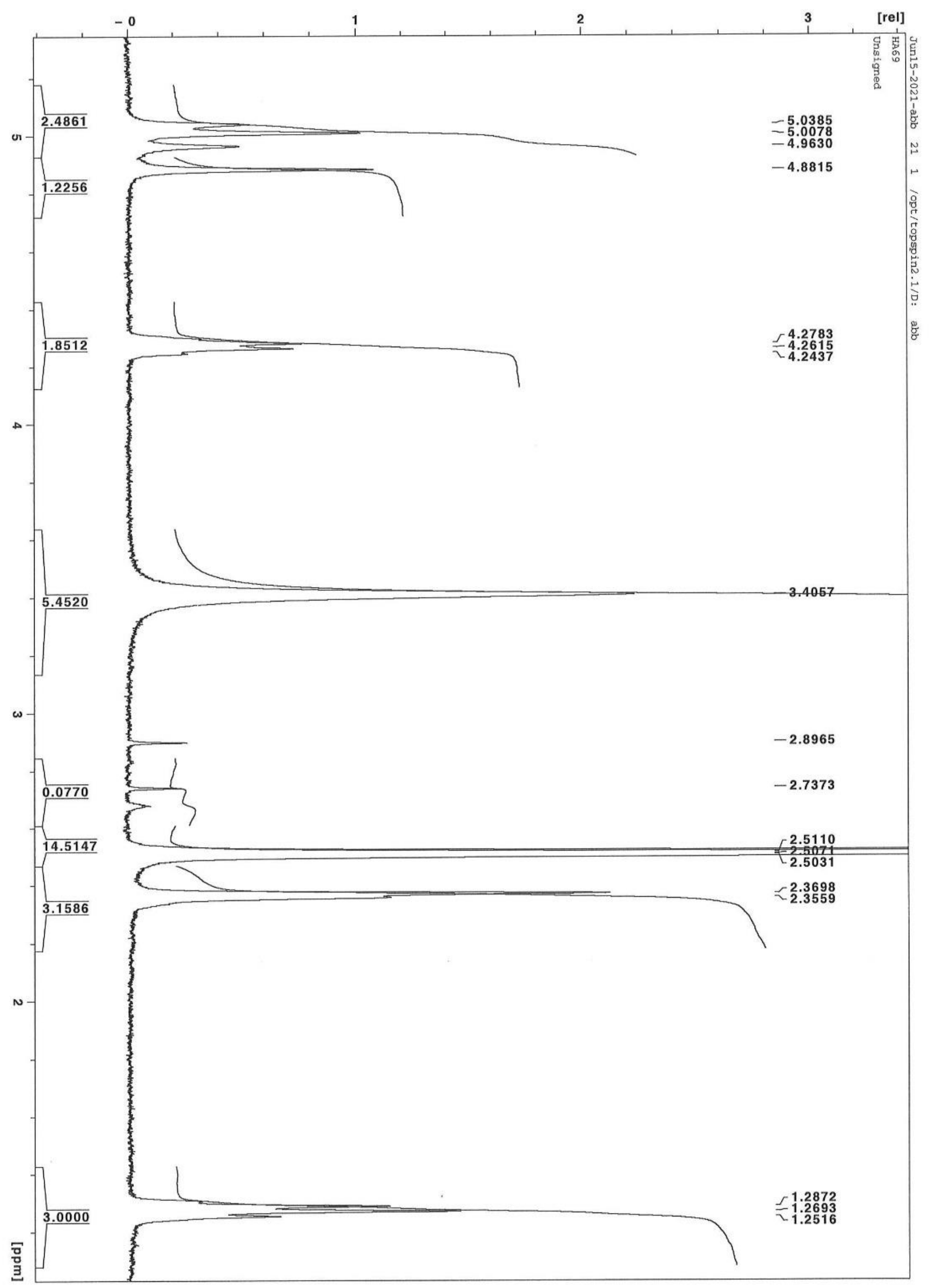

Figure S162. Detail of ^{1}H NMR spectrum of compound **7h.**

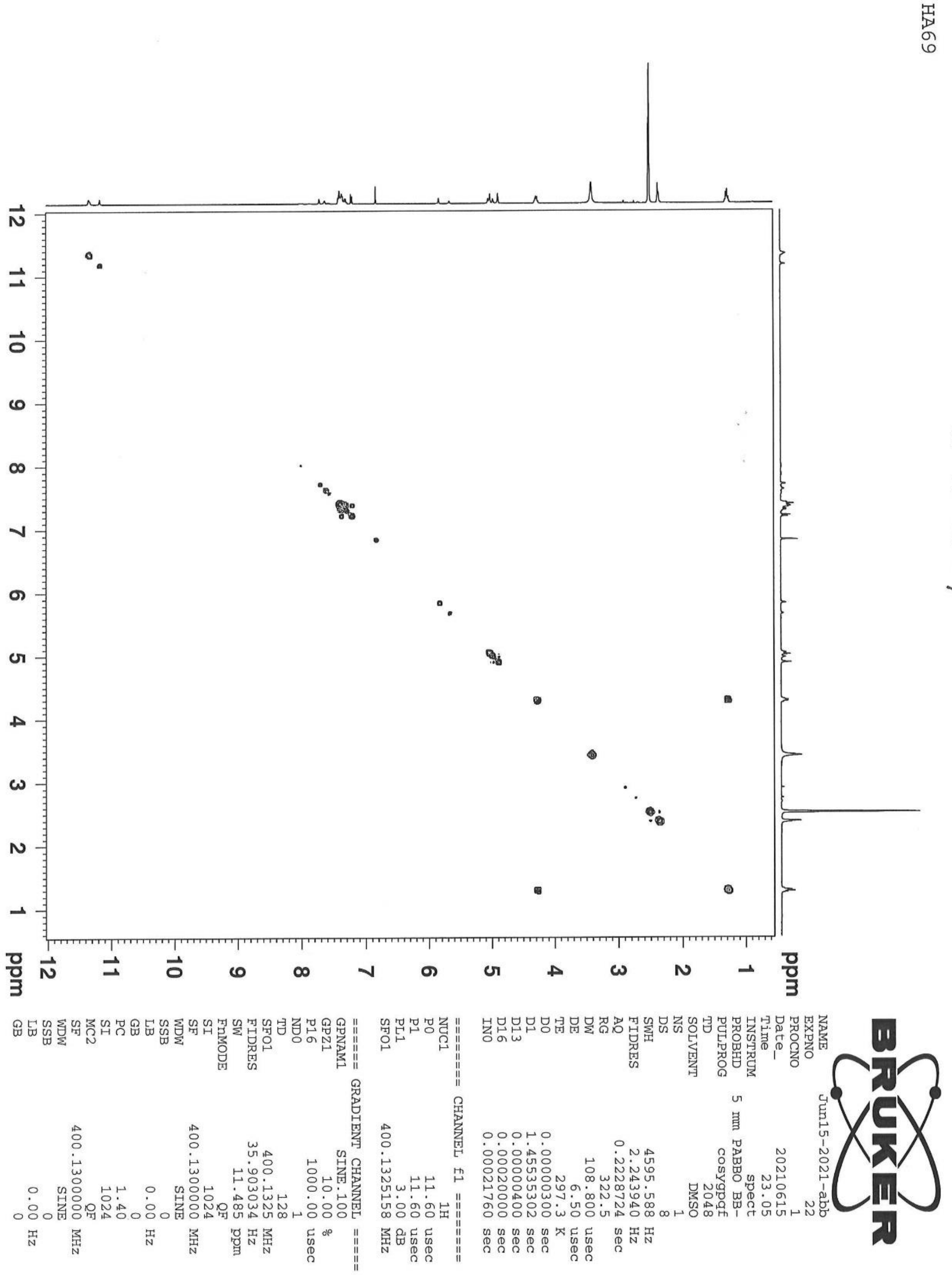

Figure S163. ^{1}H-^{1}H COSY spectrum of compound **7h.**

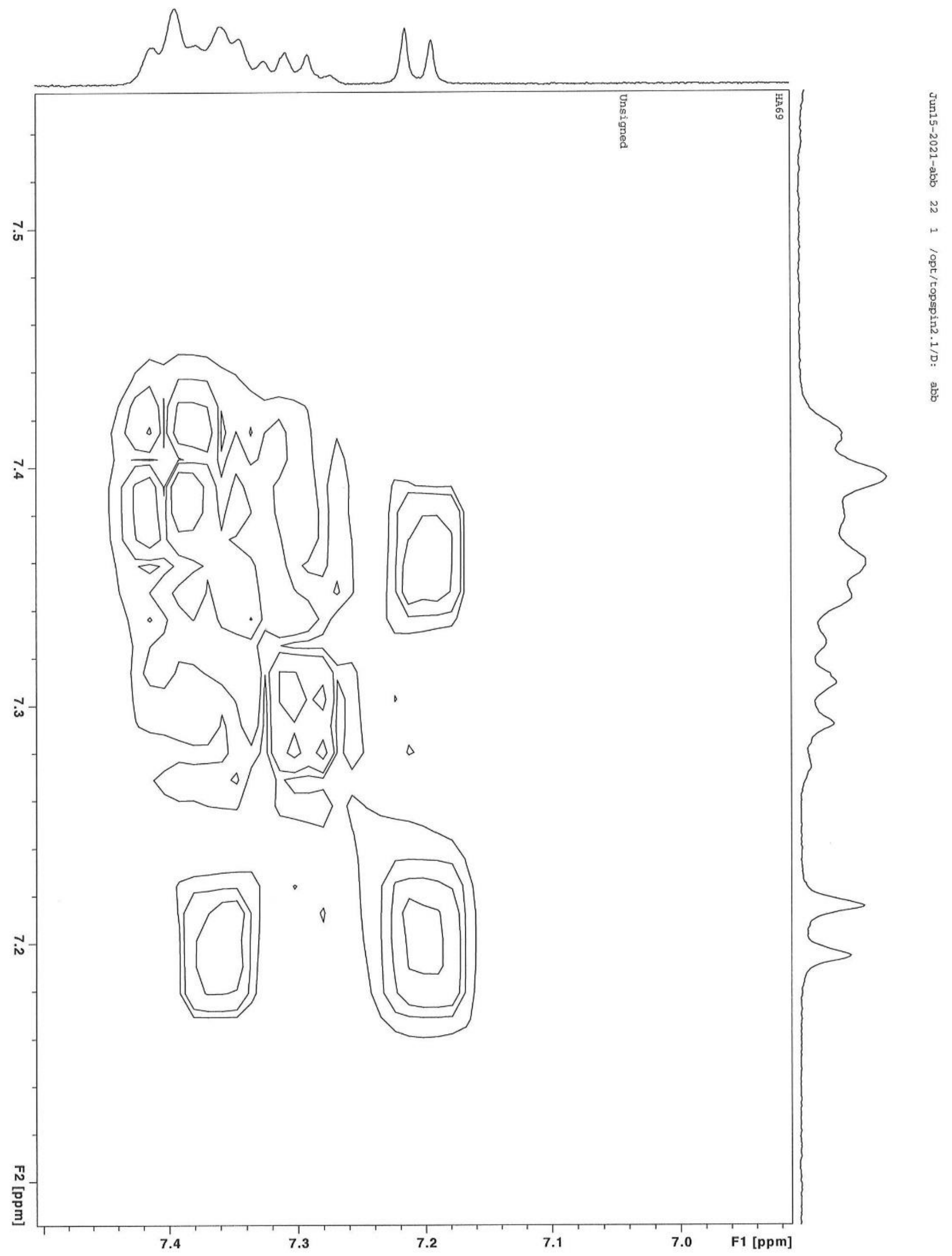

Figure S164. Detail of ^{1}H-^{1}H COSY spectrum of compound **7h.**

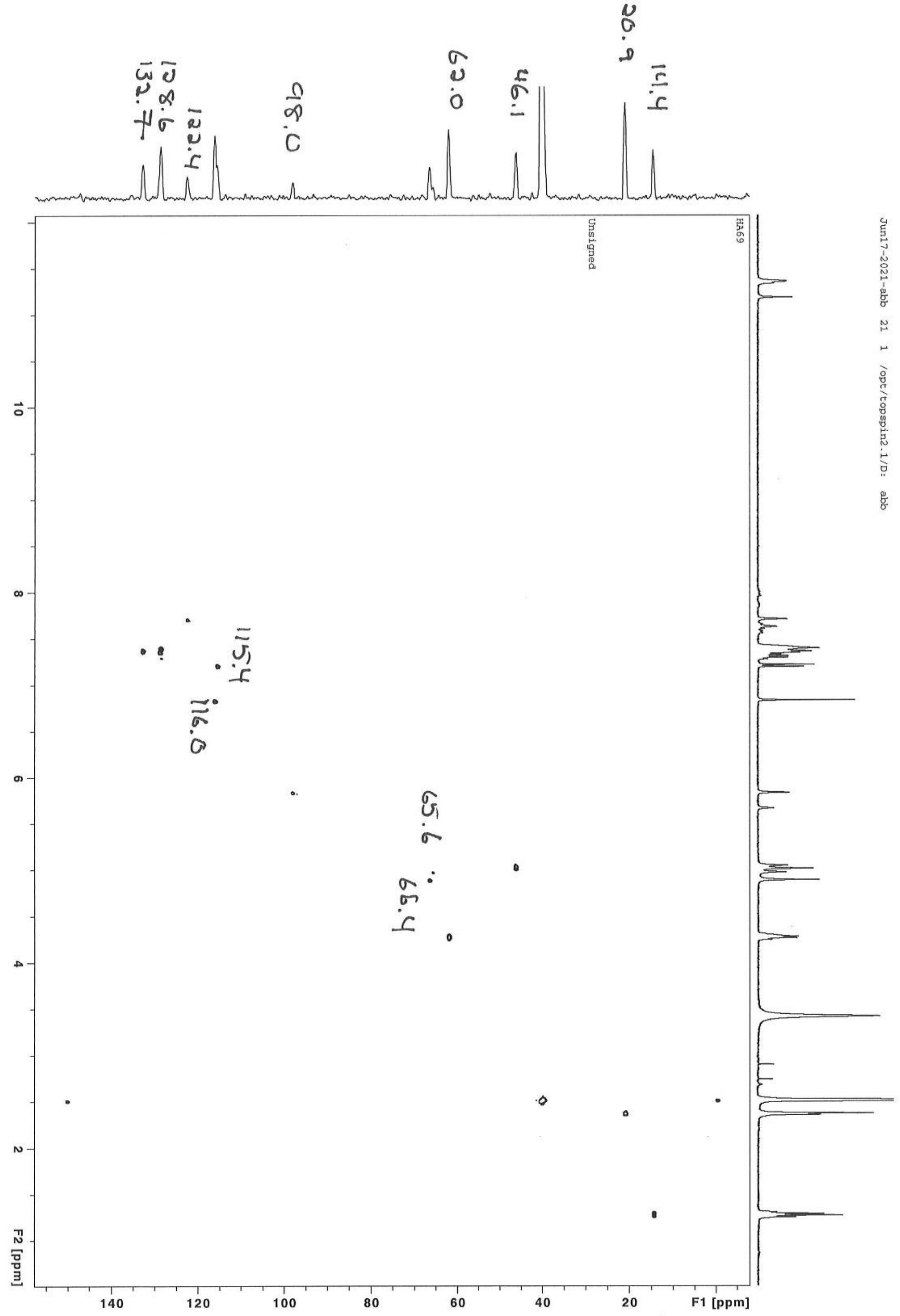

Figure S165. ^{1}H-^{13}C HSQC spectrum of compound **7h.** (Solubility was low: ^{13}C signals were detected indirectly, *via* HSQC and HMBC spectra.)

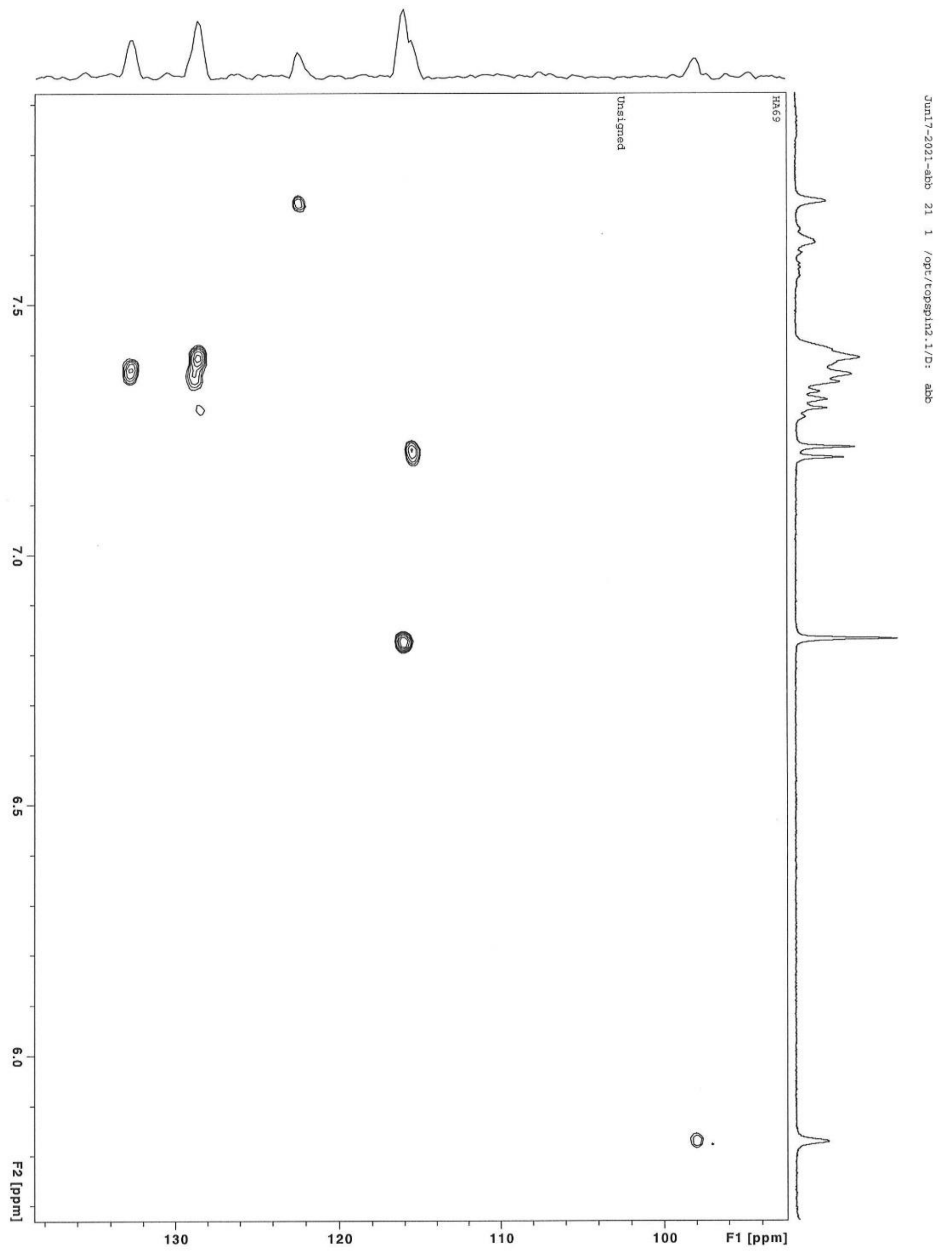

Figure S166. Detail of ^{1}H-^{13}C HSQC spectrum of compound **7h.**

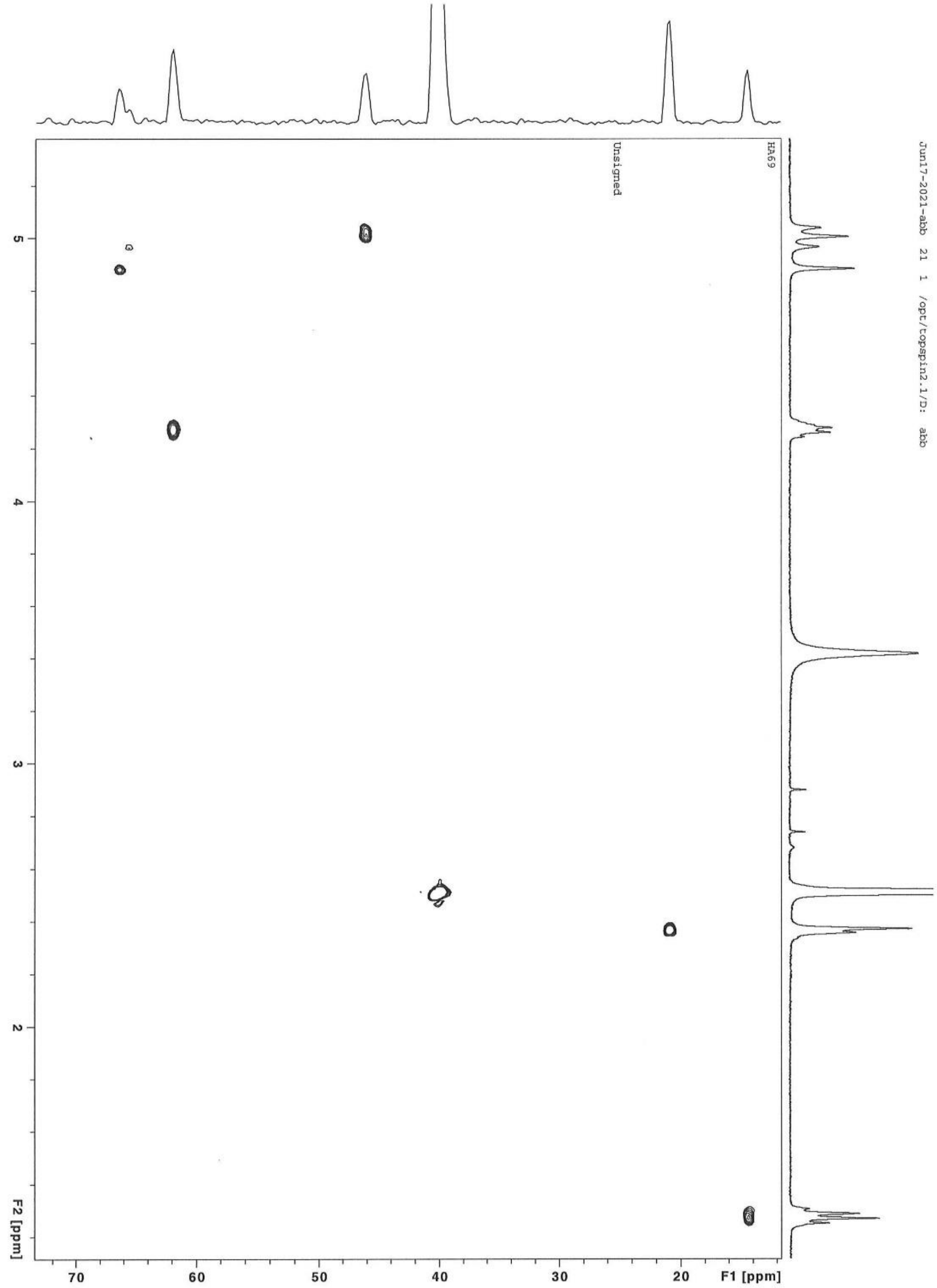

Figure S167. Detail of ^{1}H-^{13}C HSQC spectrum of compound **7h.**

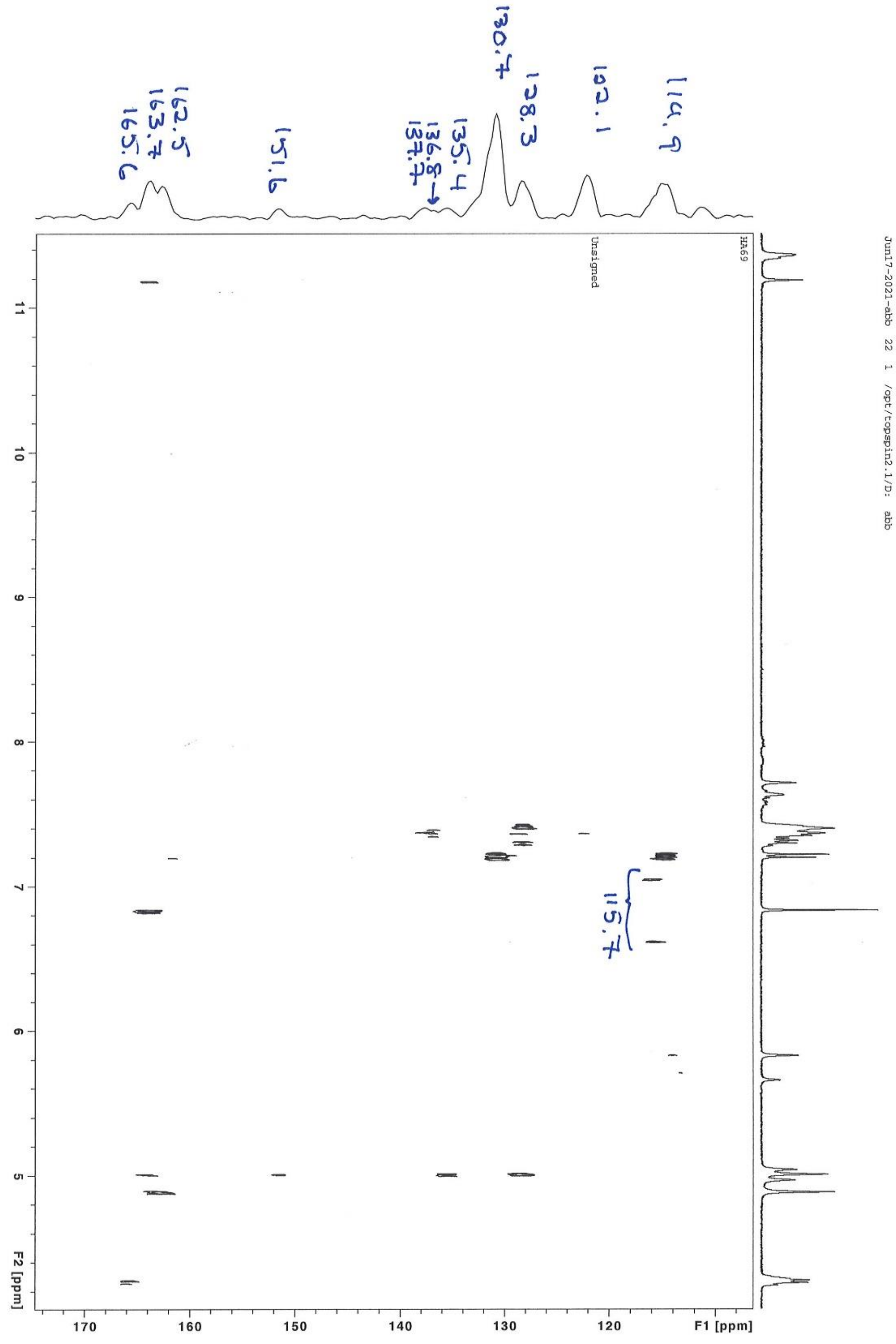

Figure S168. ^{1}H-^{13}C HMBC spectrum of compound **7h.**

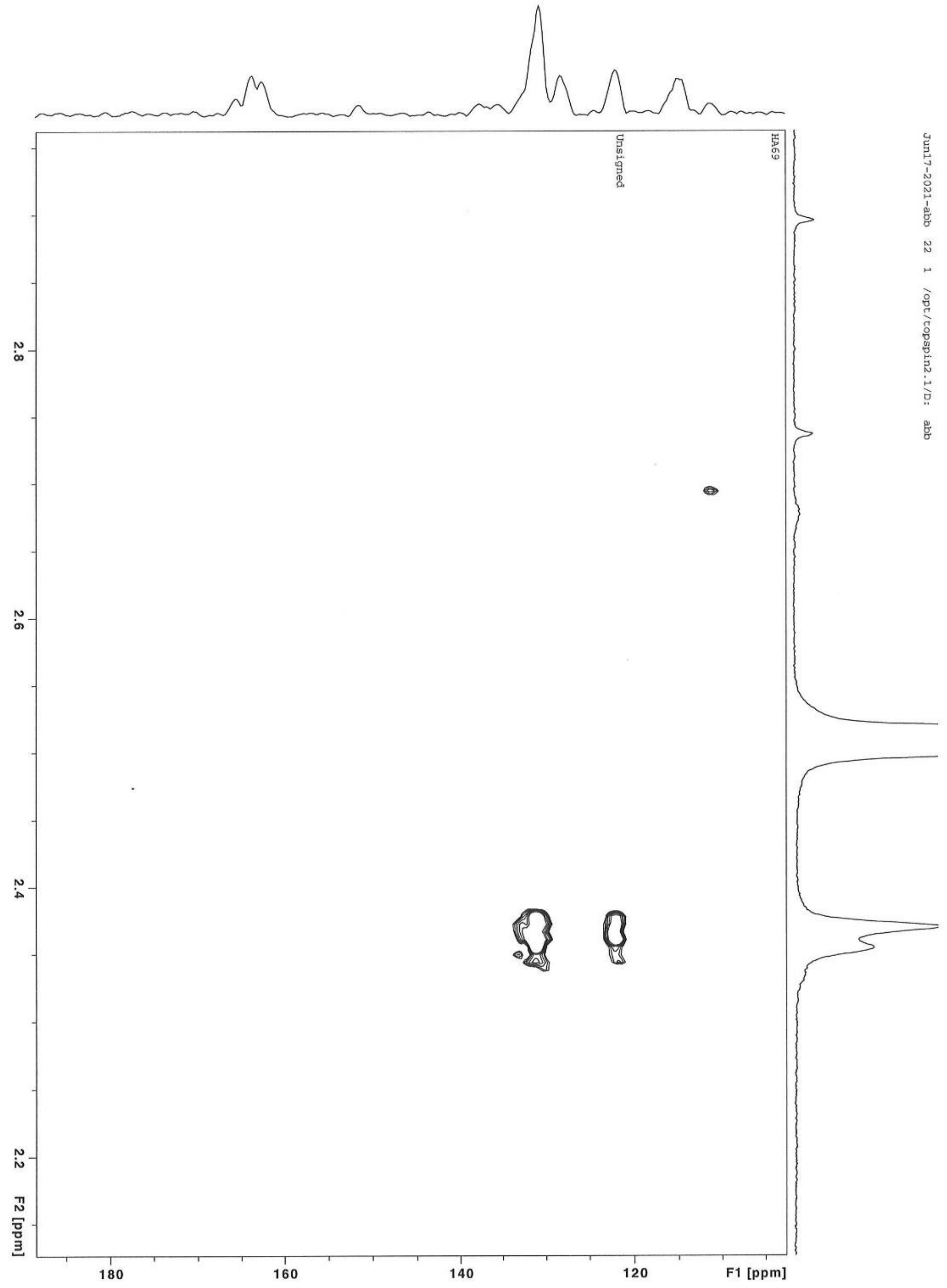

Figure S169. Detail of ^{1}H-^{13}C HMBC spectrum of compound **7h.**

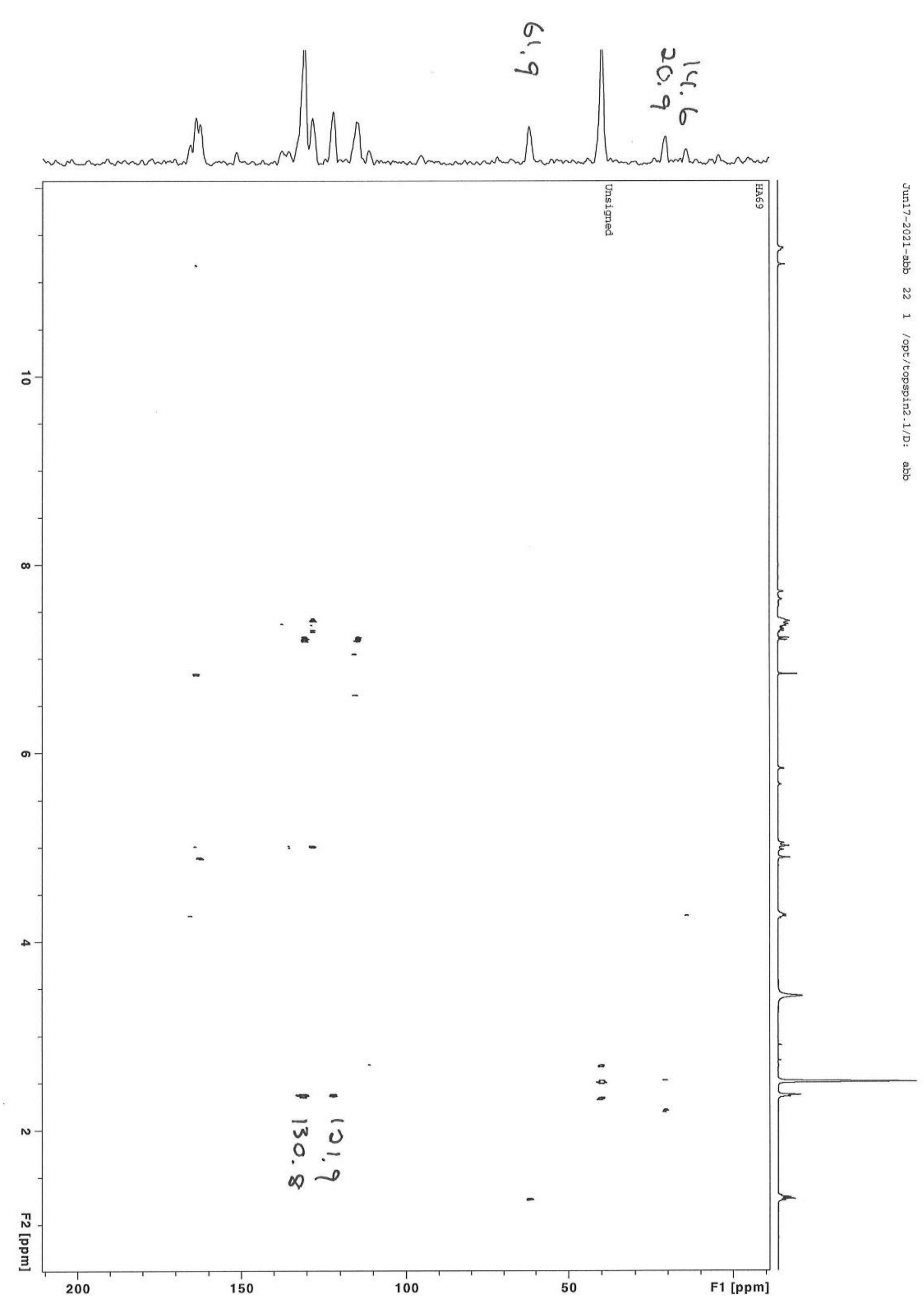

Figure S170. Detail of ^{1}H-^{13}C HMBC spectrum of compound **7h.**

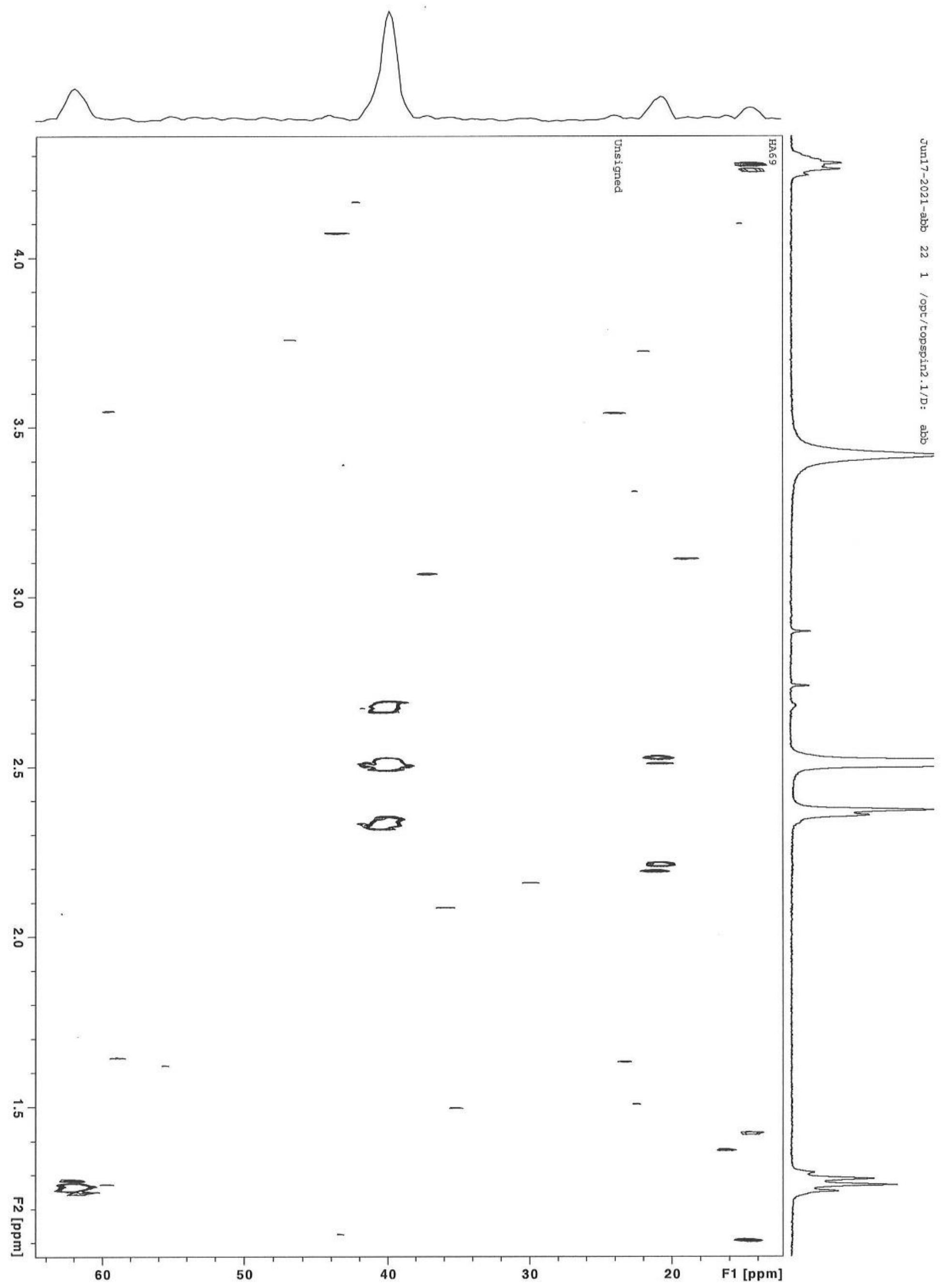

Figure S171. Detail of ^{1}H-^{13}C HMBC spectrum of compound **7h.** (^{1}H-^{15}N HSQC and HMBC experiments gave no cross peaks.)

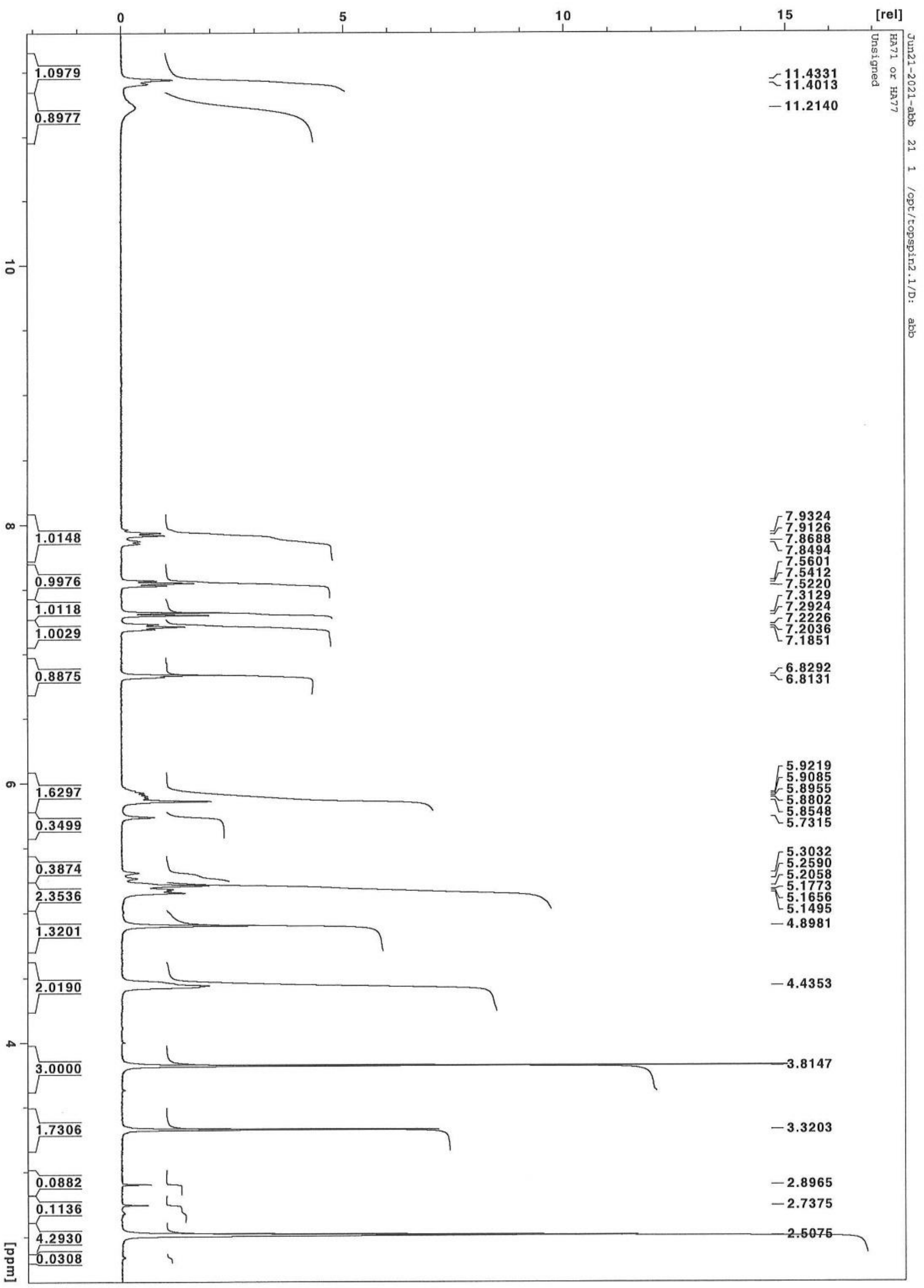

Figure S172. ^{1}H NMR spectrum of compound **7j** (400 MHz, DMSO-d_6).

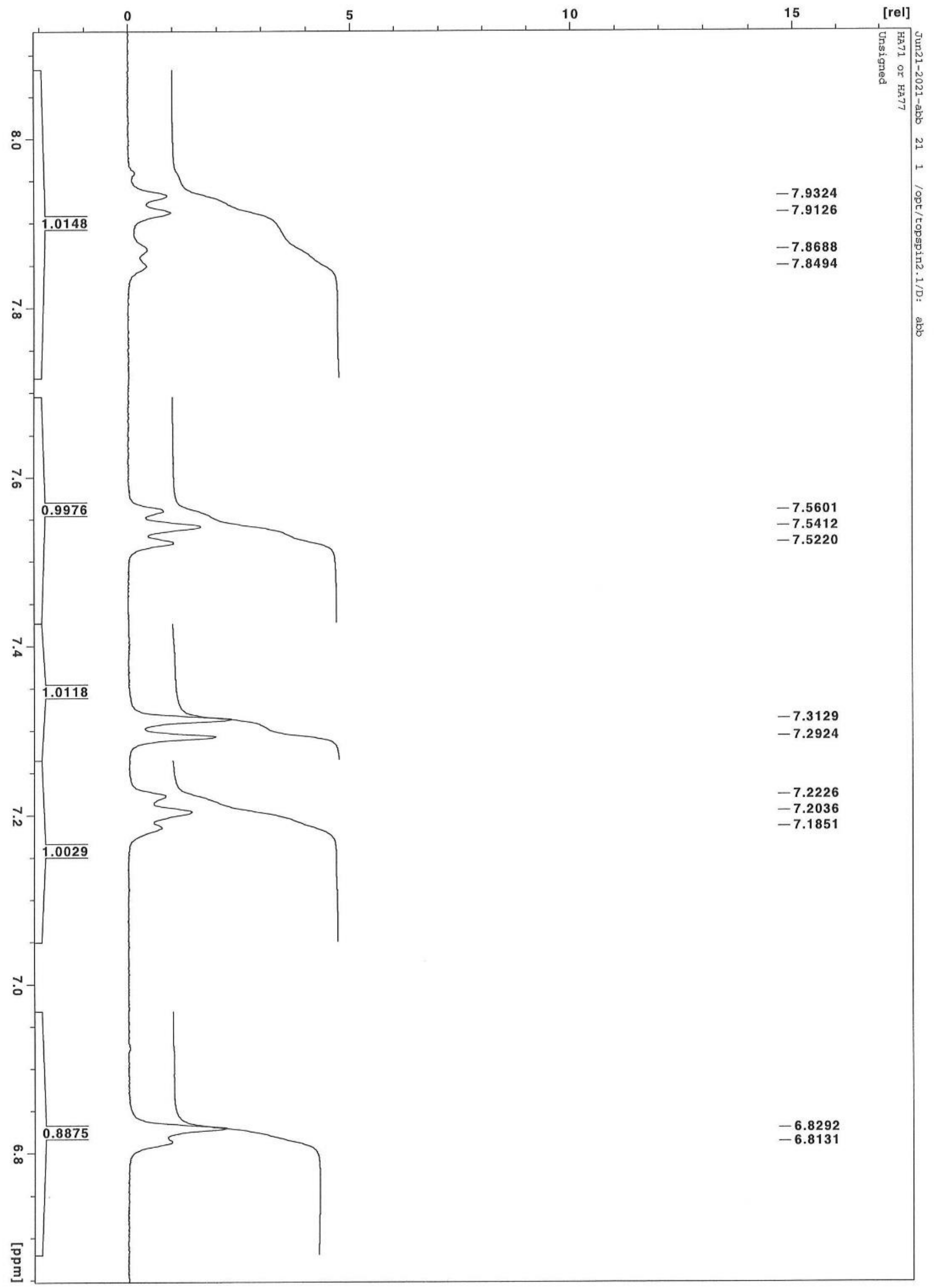

Figure S173. Detail of ^{1}H NMR spectrum of compound **7j.**

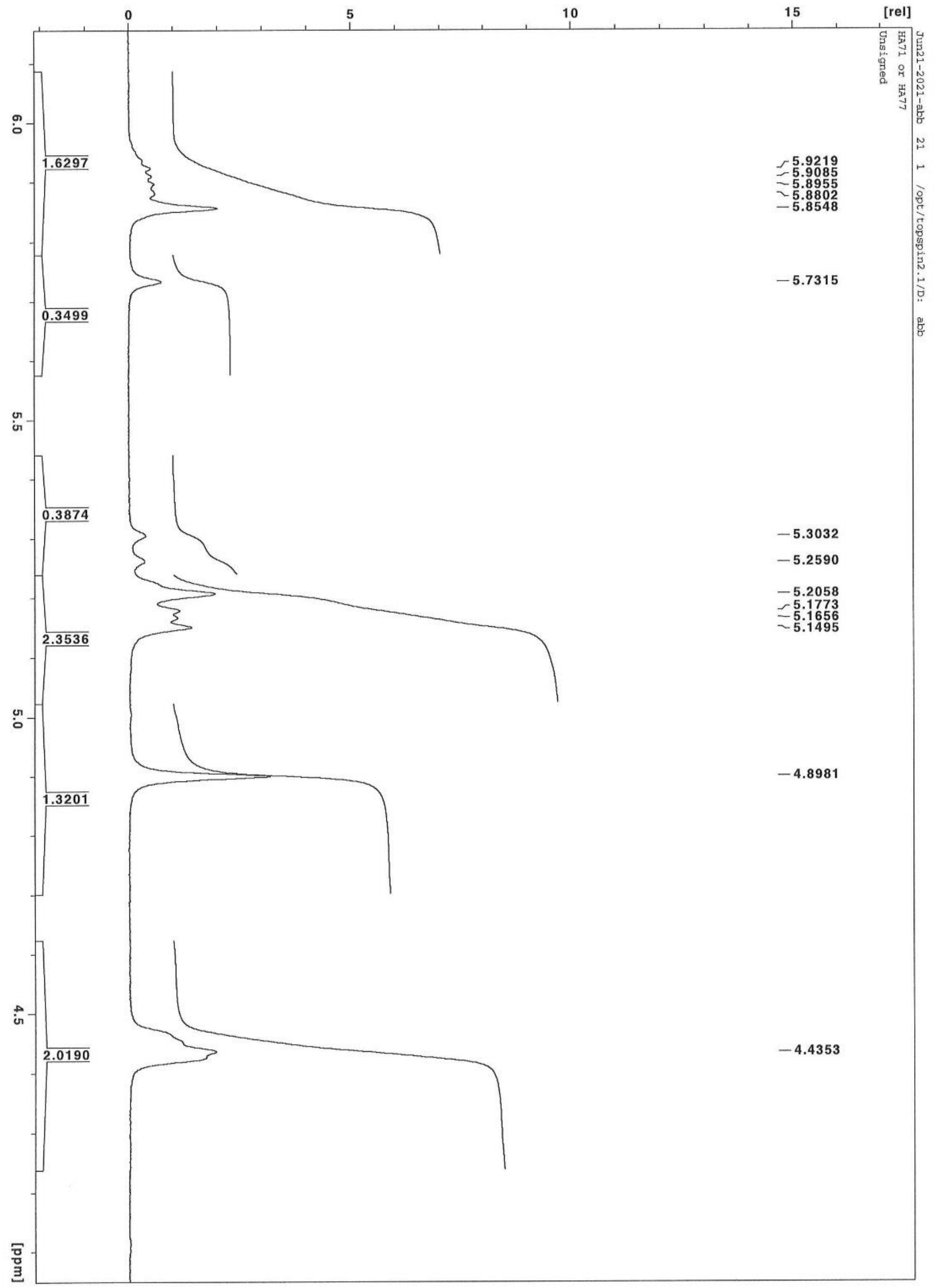

Figure S174. Detail of ^{1}H NMR spectrum of compound **7j.**

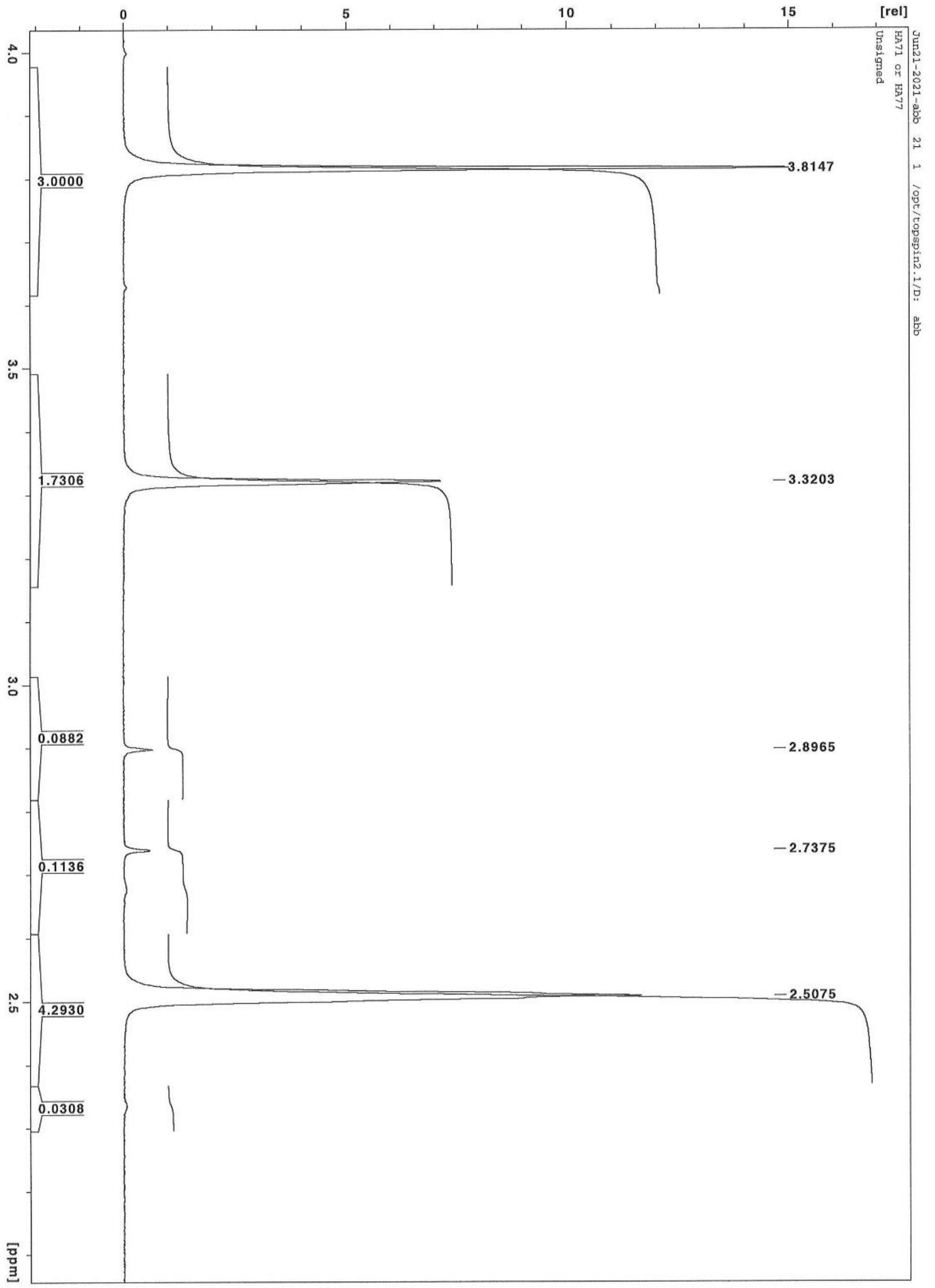

Figure S175. Detail of ^{1}H NMR spectrum of compound **7j.**

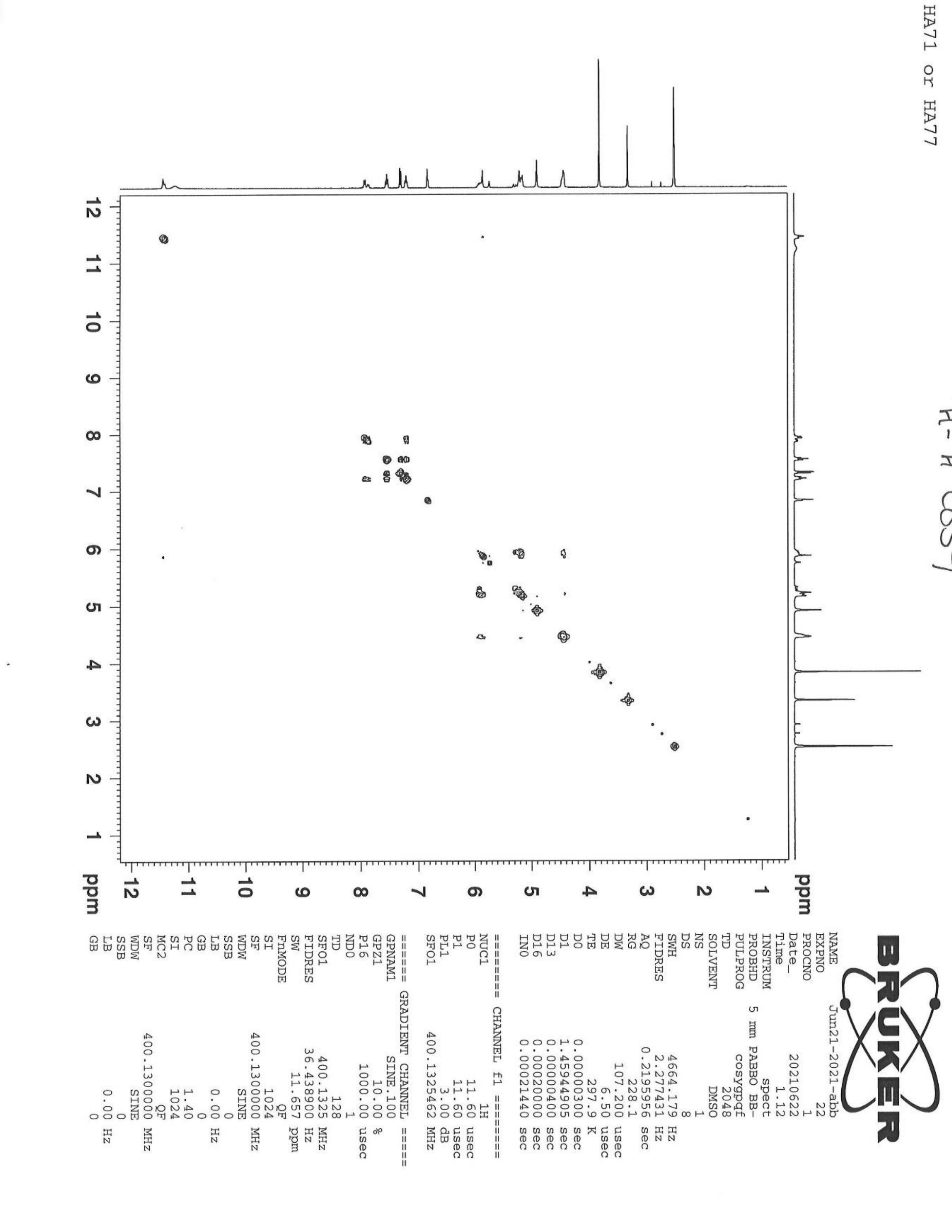

Figure S176. ^{1}H-^{1}H COSY spectrum of compound **7j.**

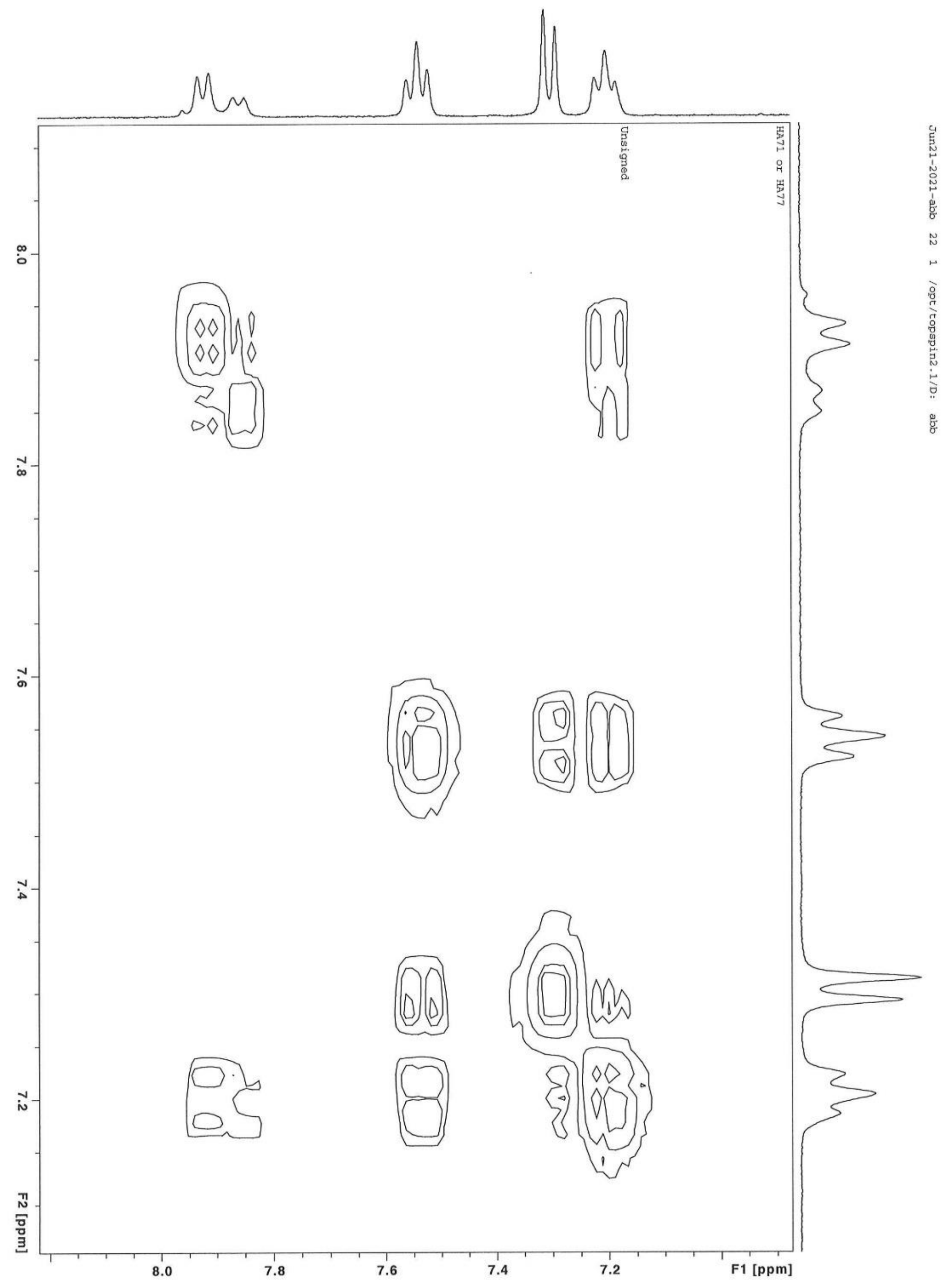

Figure S177. Detail of ^{1}H-^{1}H COSY spectrum of compound **7j.**

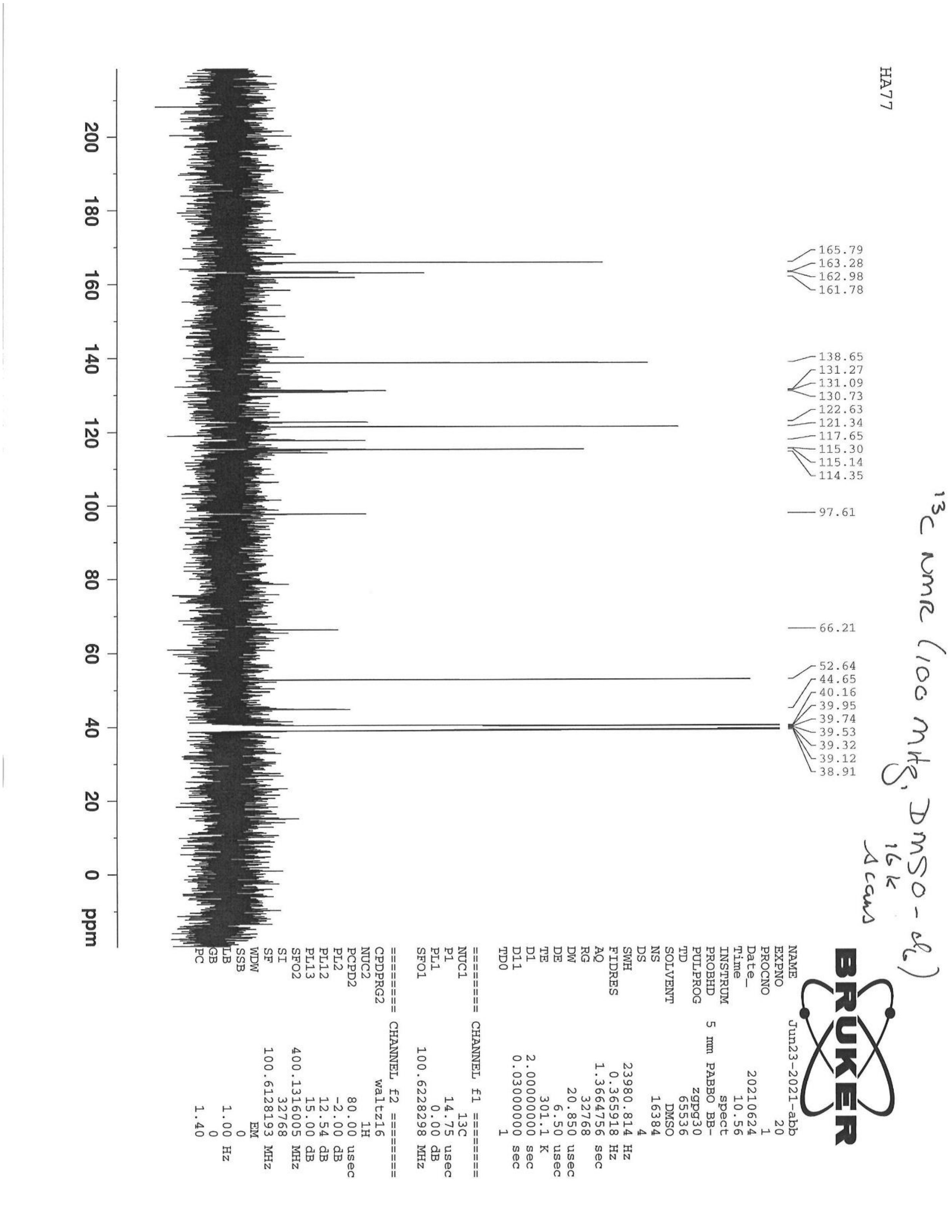

Figure S178. ^{13}C NMR spectrum of compound **7j** (100 MHz, DMSO-d_6).

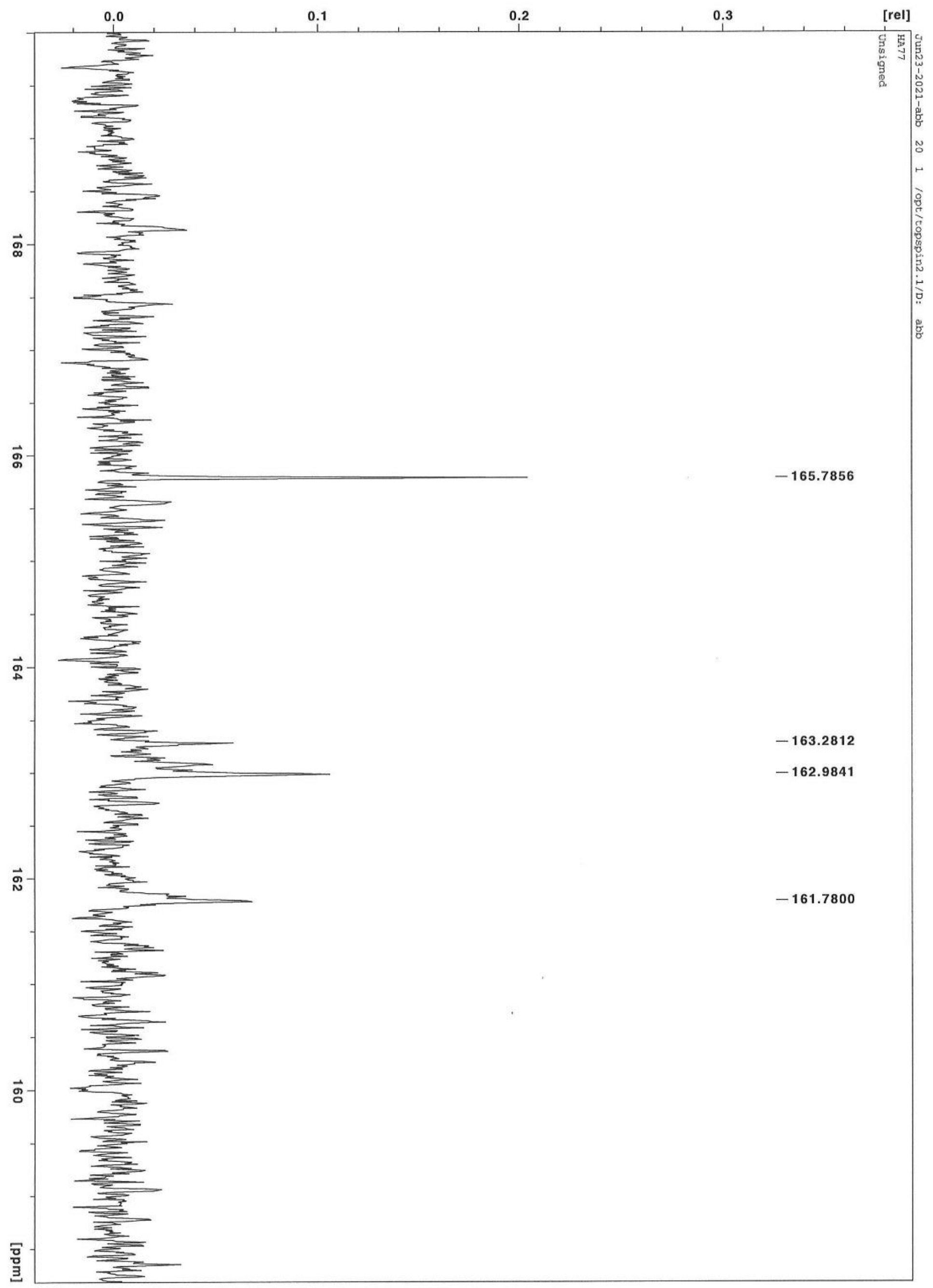

Figure S179. Detail of ^{13}C NMR spectrum of compound **7j.**

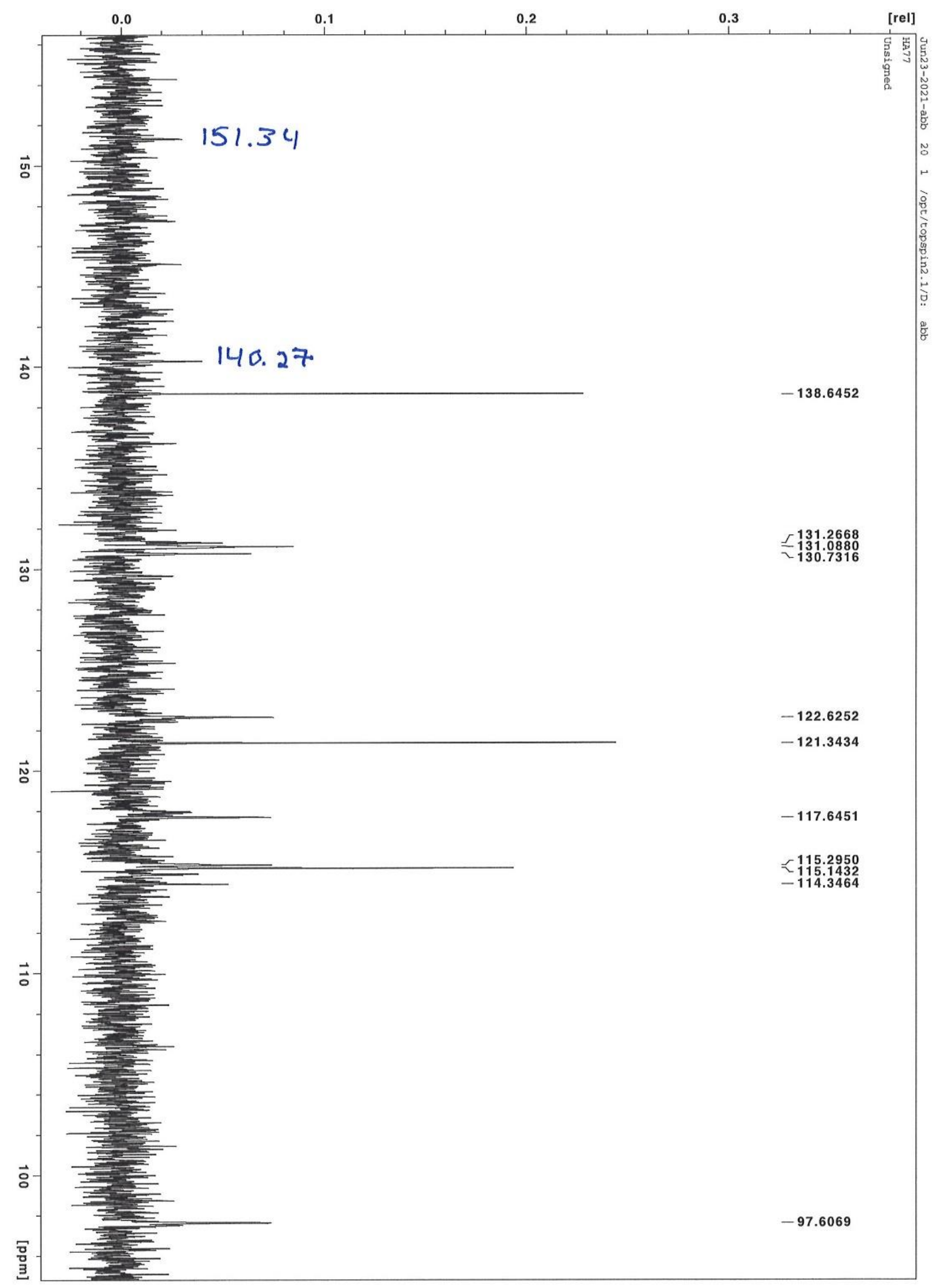

Figure S180. Detail of ^{13}C NMR spectrum of compound **7j.**

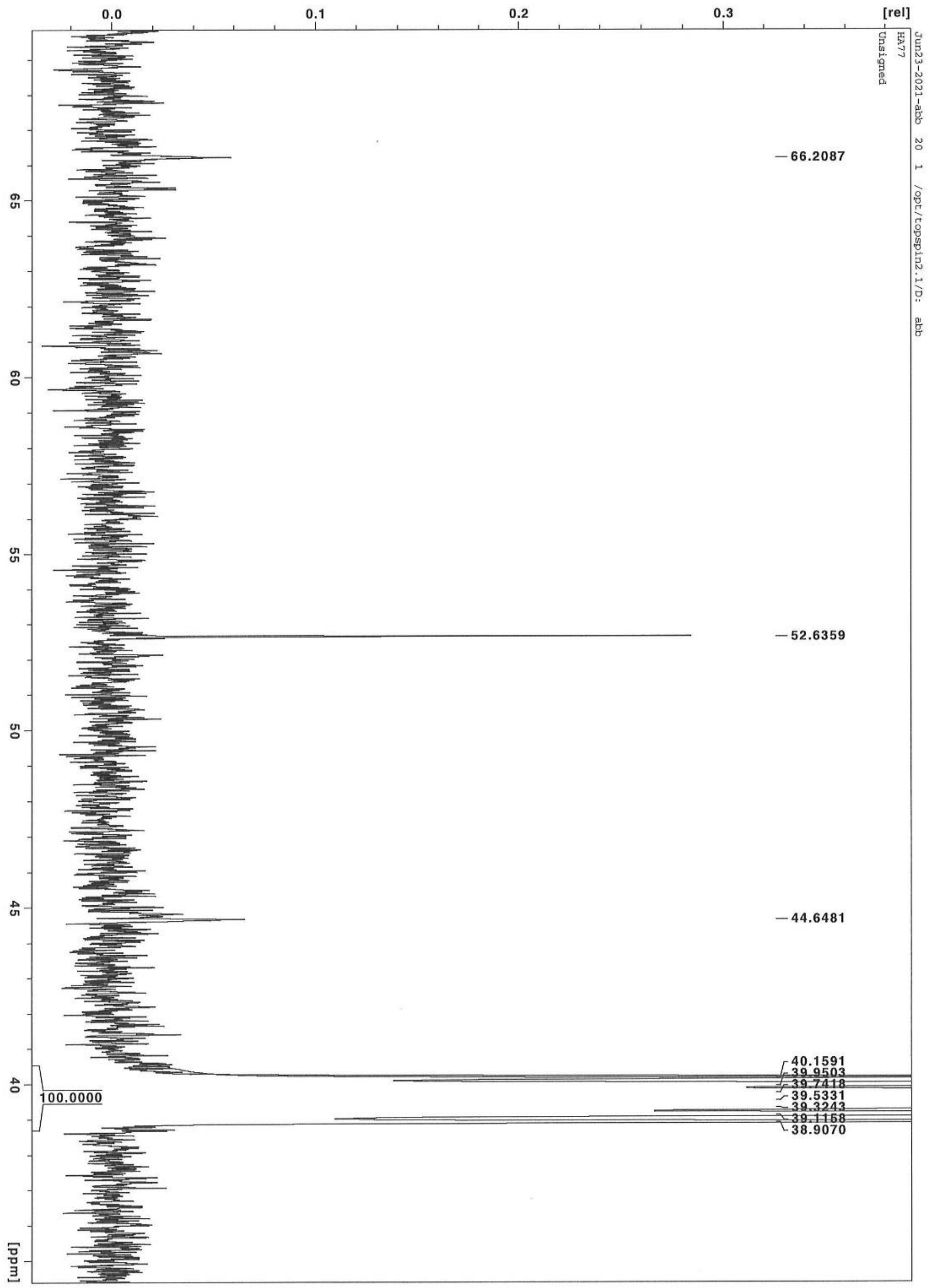

Figure S181. Detail of ^{13}C NMR spectrum of compound **7j.**

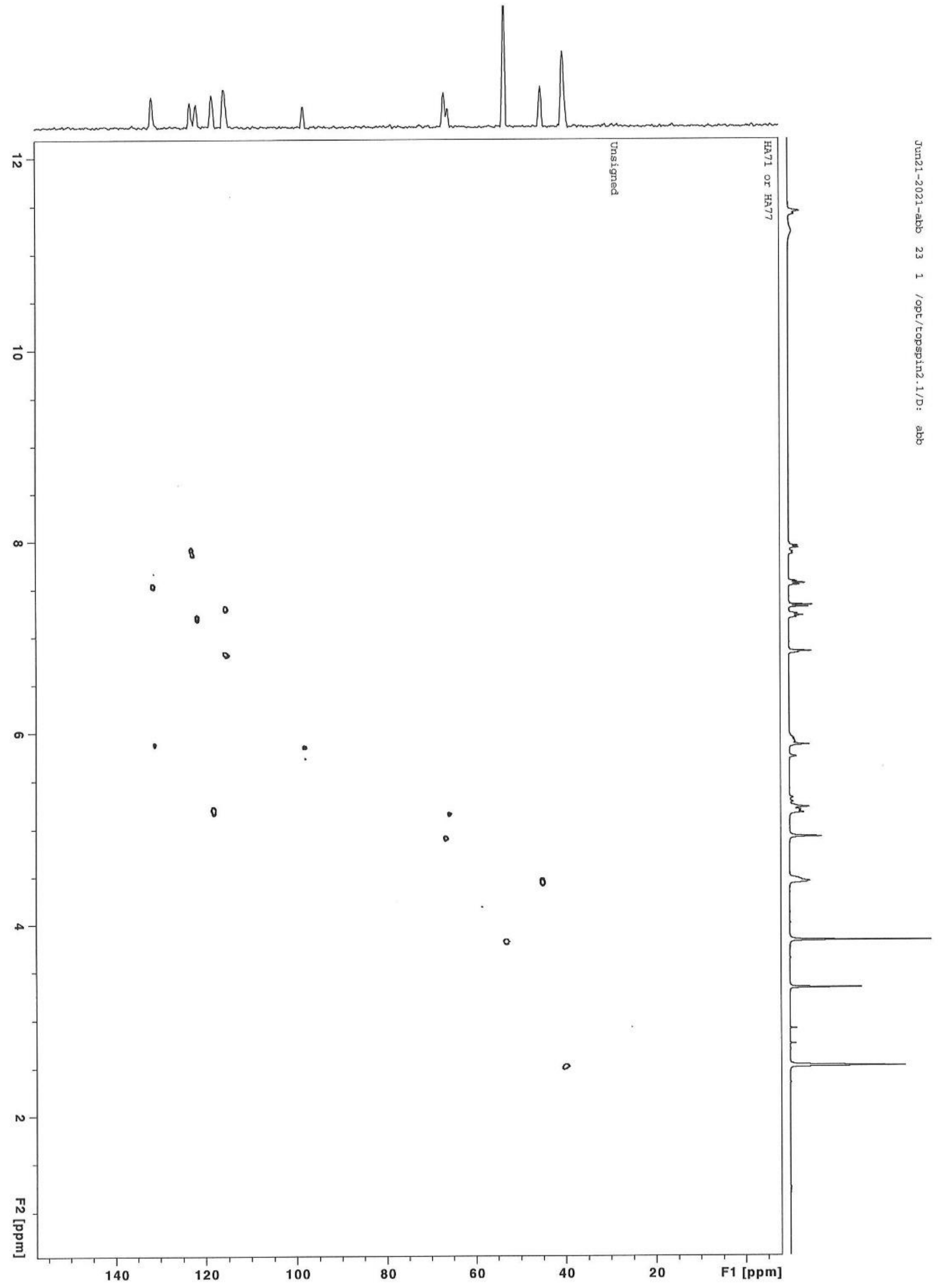

Figure S182. ^{1}H-^{13}C HSQC spectrum of compound **7j.**

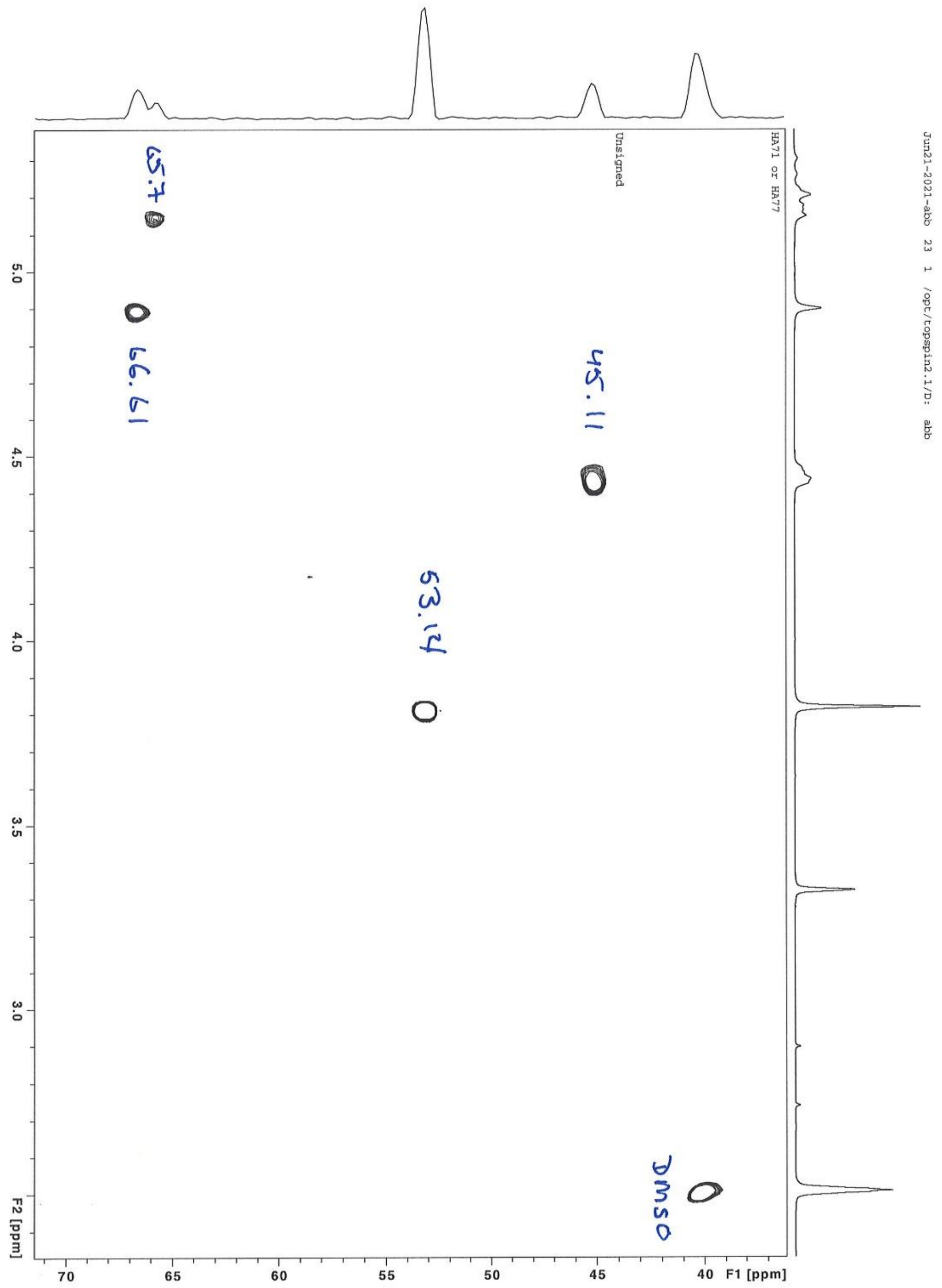

Figure S183. Detail of ^{1}H-^{13}C HSQC spectrum of compound **7j.**

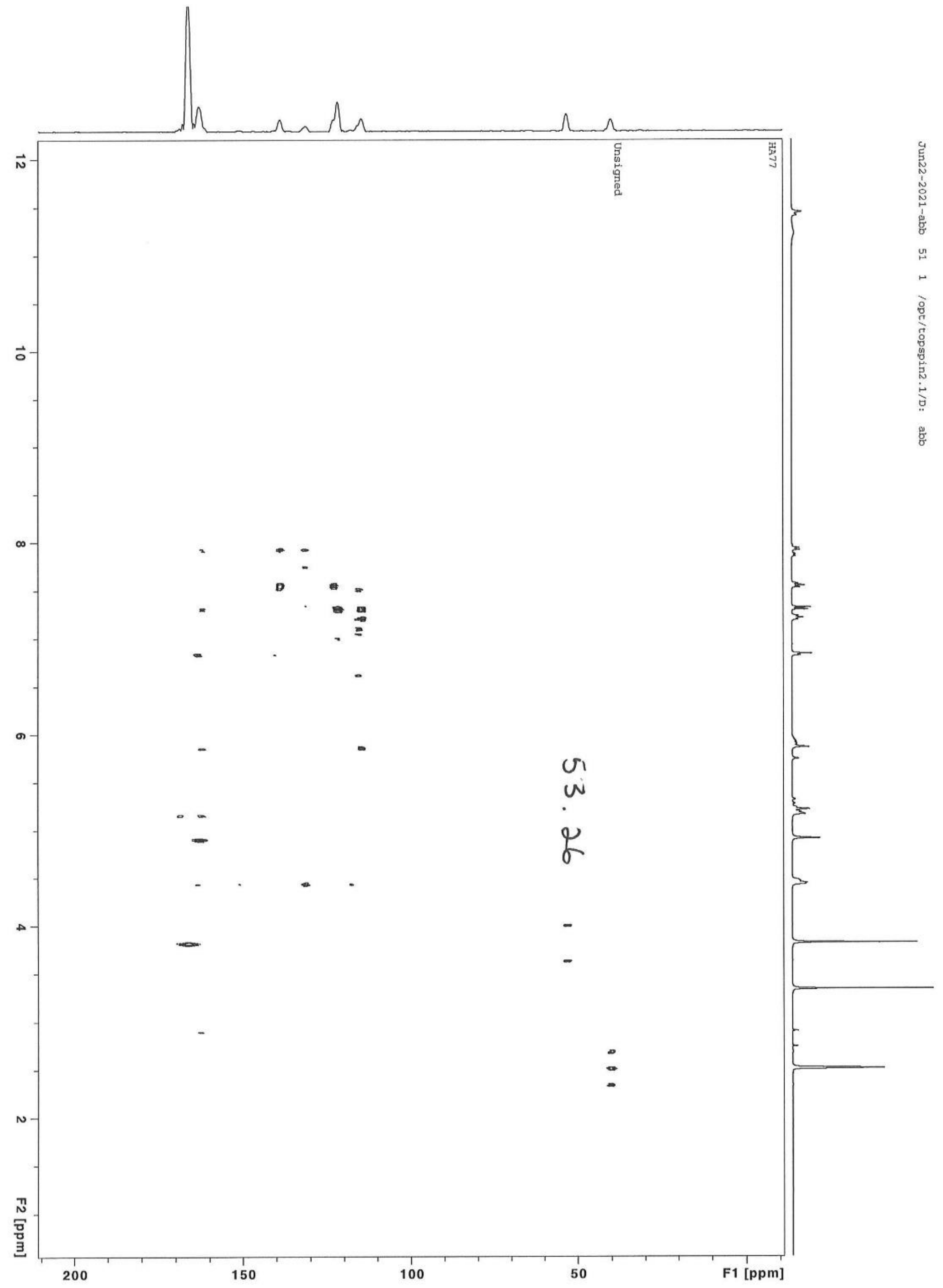

Figure S184. ^{1}H-^{13}C HMBC spectrum of compound **7j.**

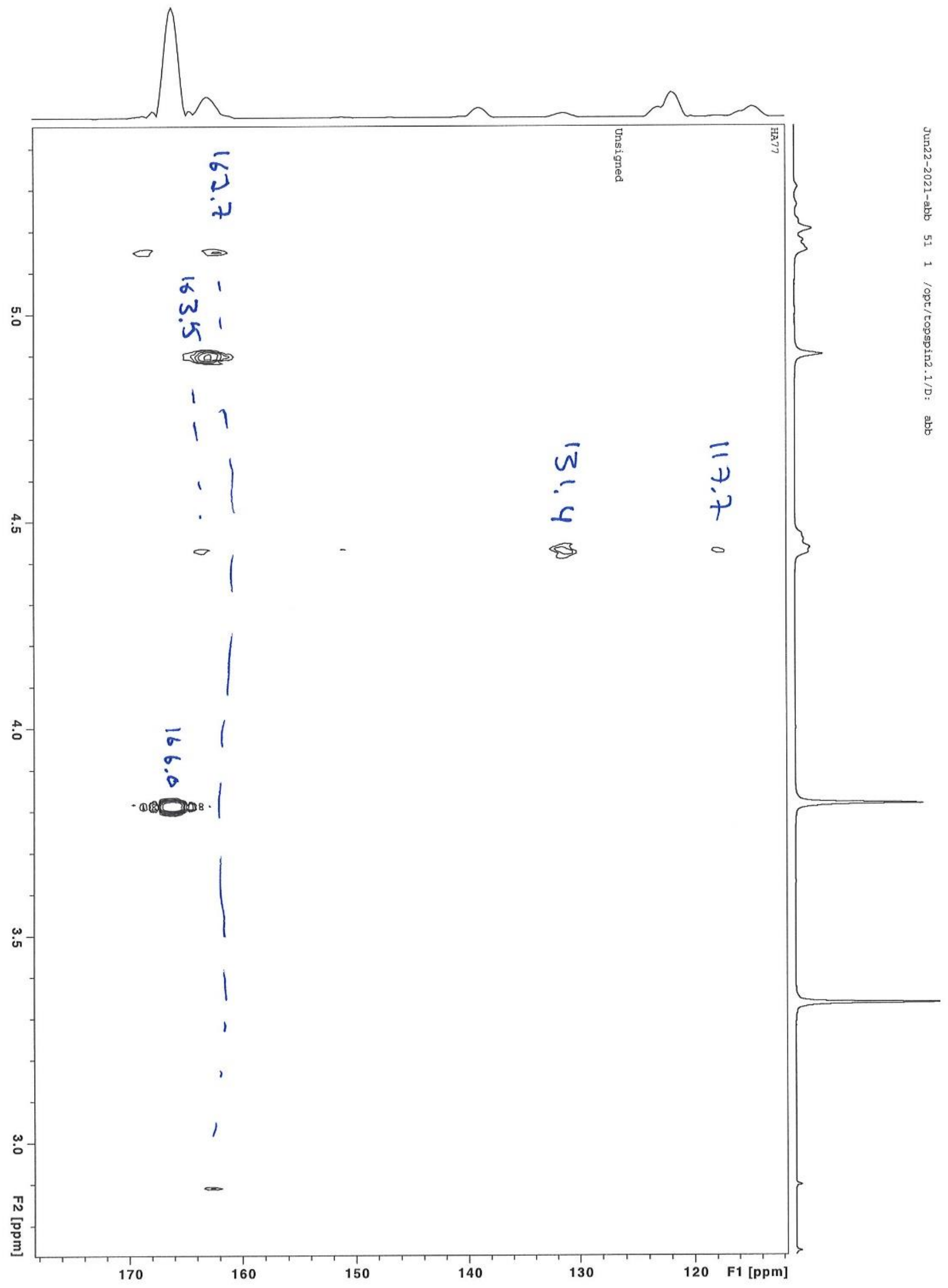

Figure S185. Detail of ^{1}H-^{13}C HMBC spectrum of compound **7j.**

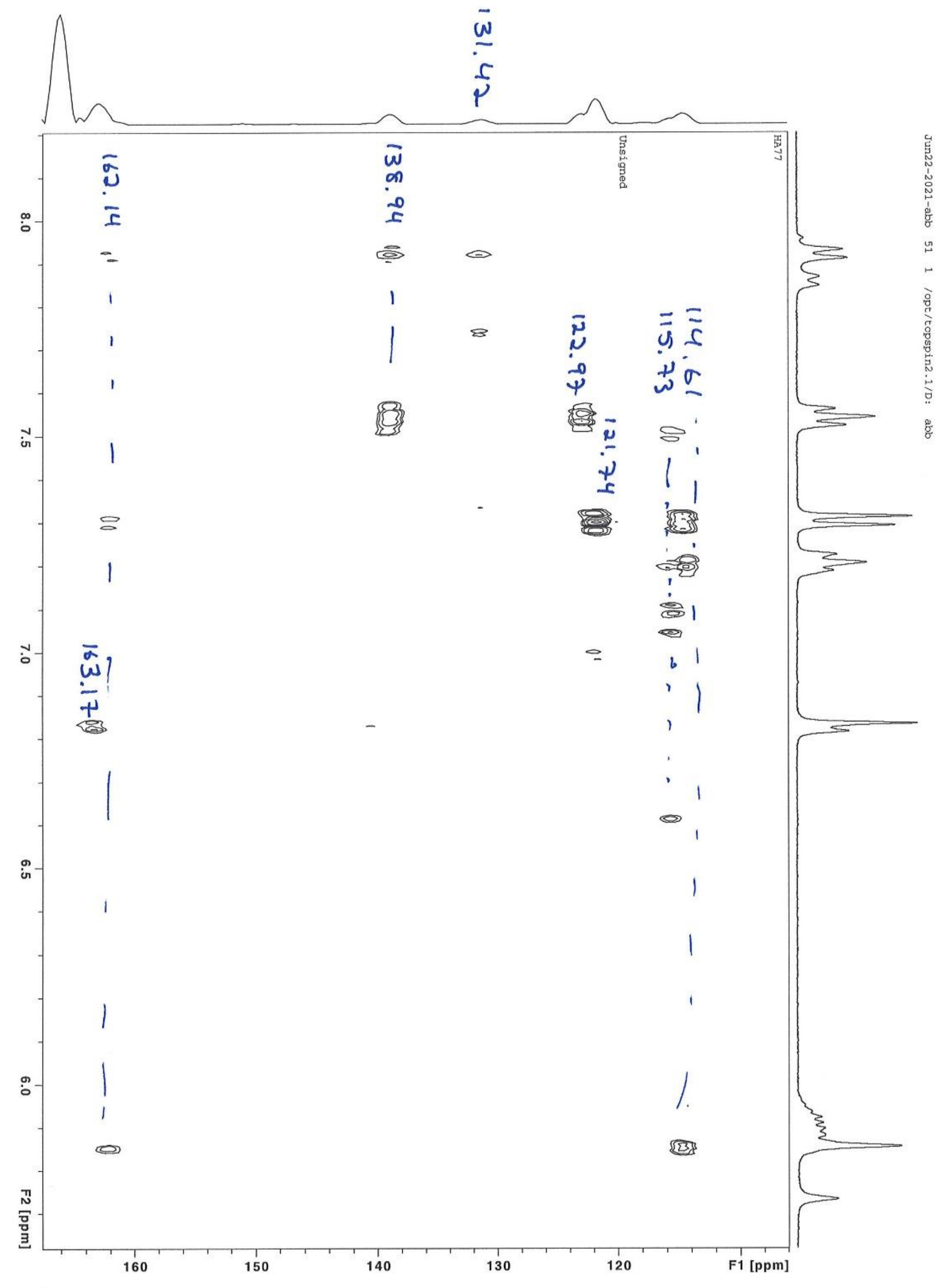

Figure S186. Detail of ^{1}H-^{13}C HMBC spectrum of compound **7j.**

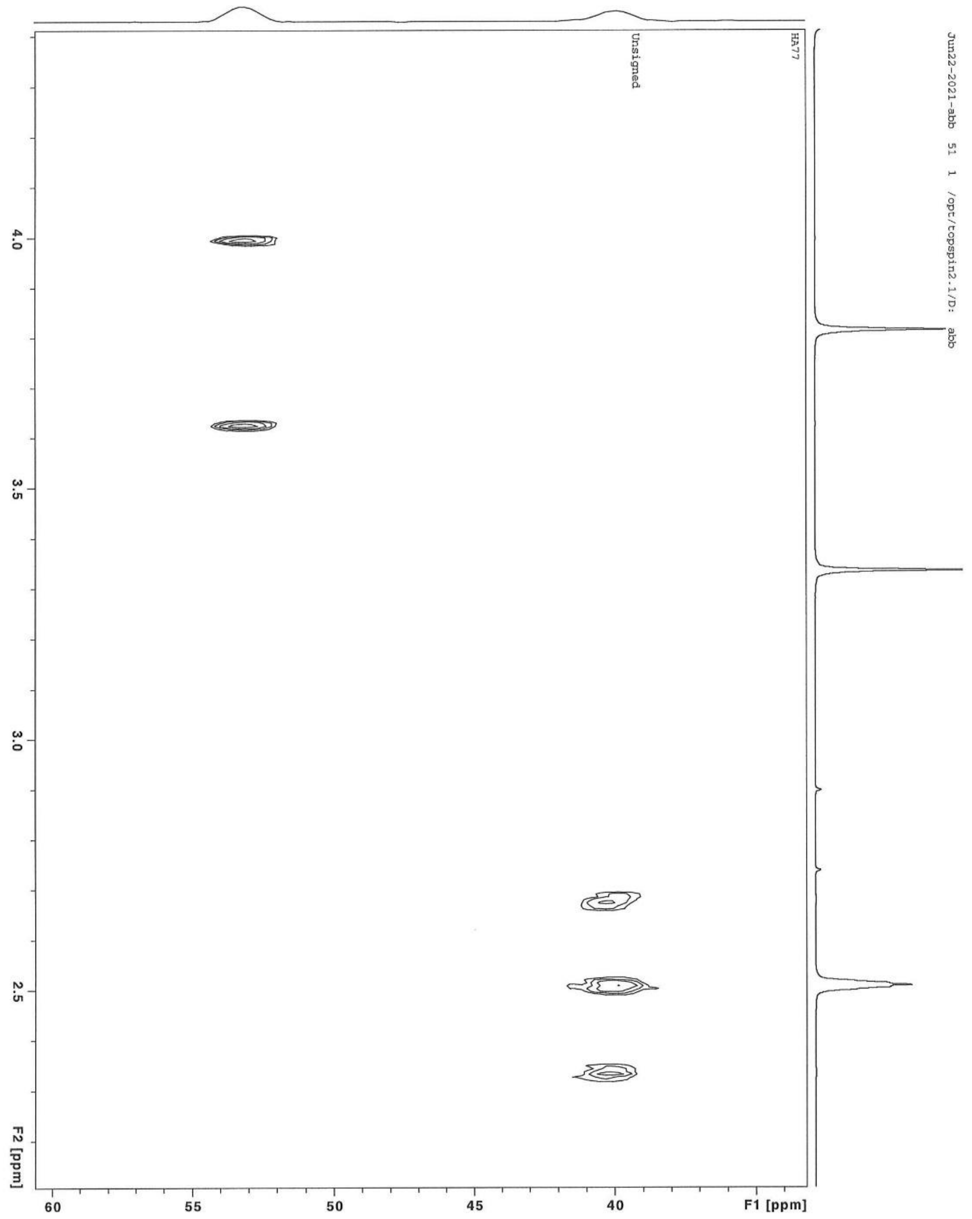

Figure S187. Detail of ^{1}H-^{13}C HMBC spectrum of compound **7j.**

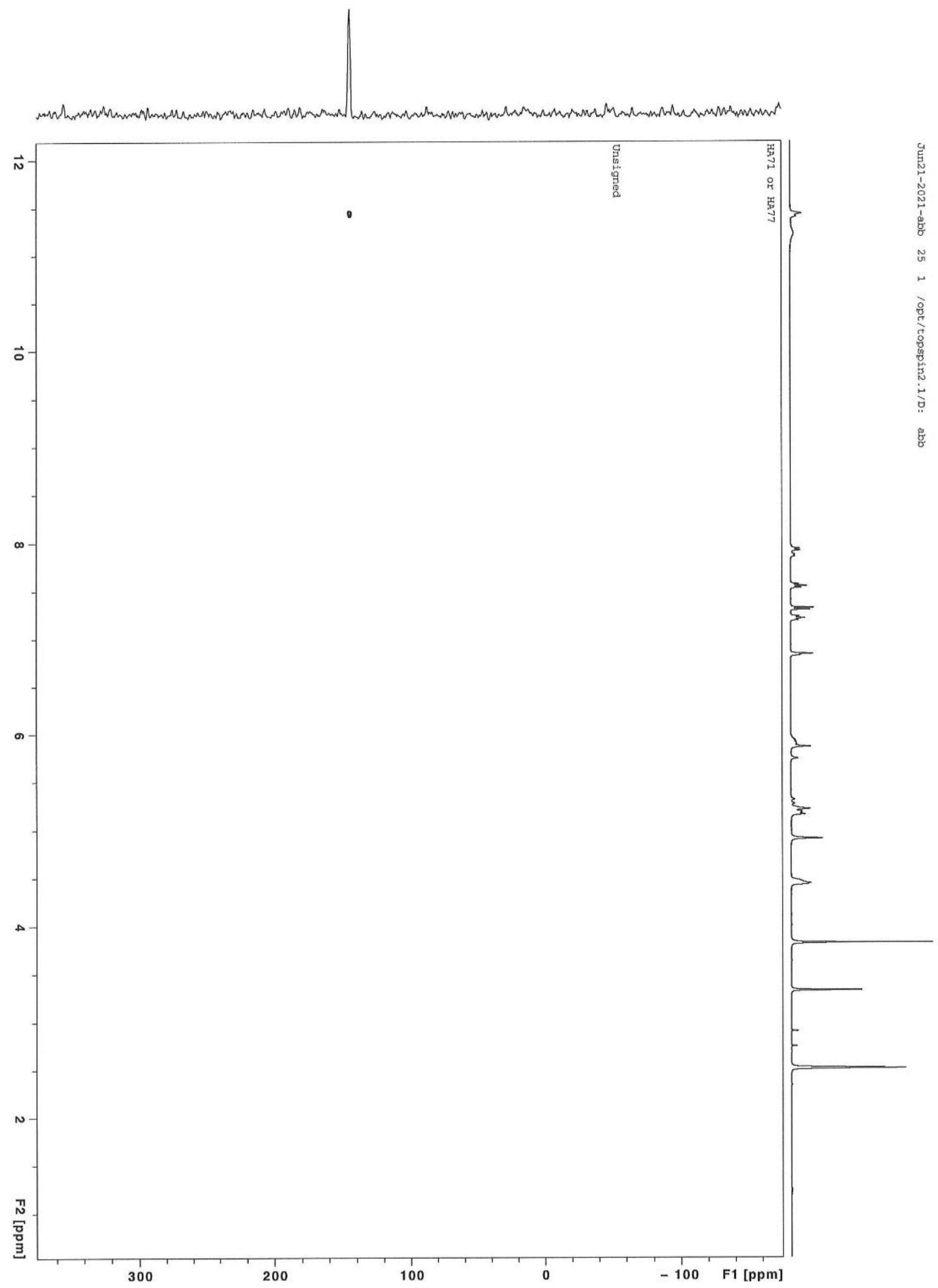

Figure S188. ^{1}H-^{15}N HSQC spectrum of compound **7j.**

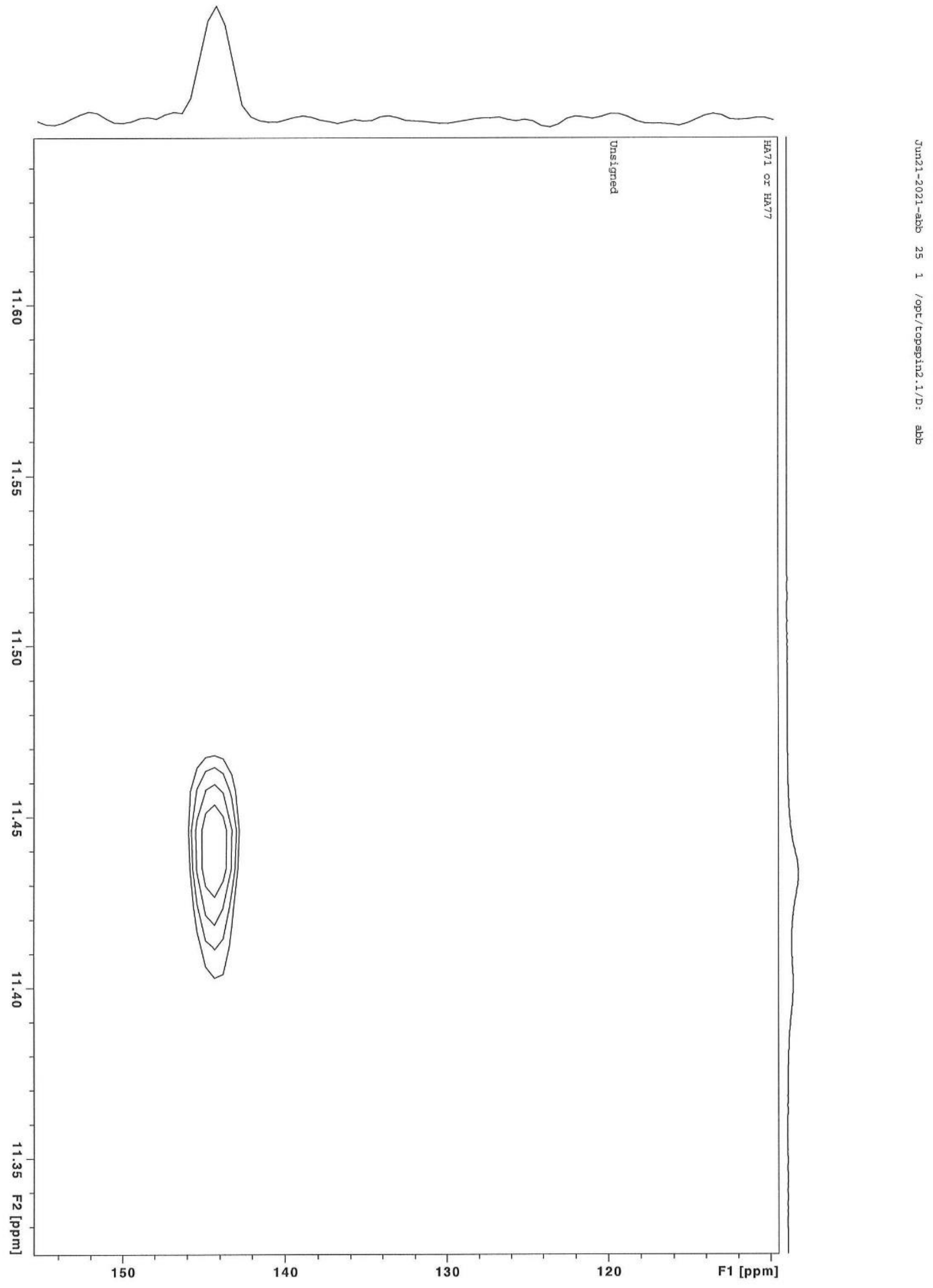

Figure S189. Detail of ^{1}H-^{15}N HSQC spectrum of compound **7j** (HMBC spectrum gave no cross peaks).

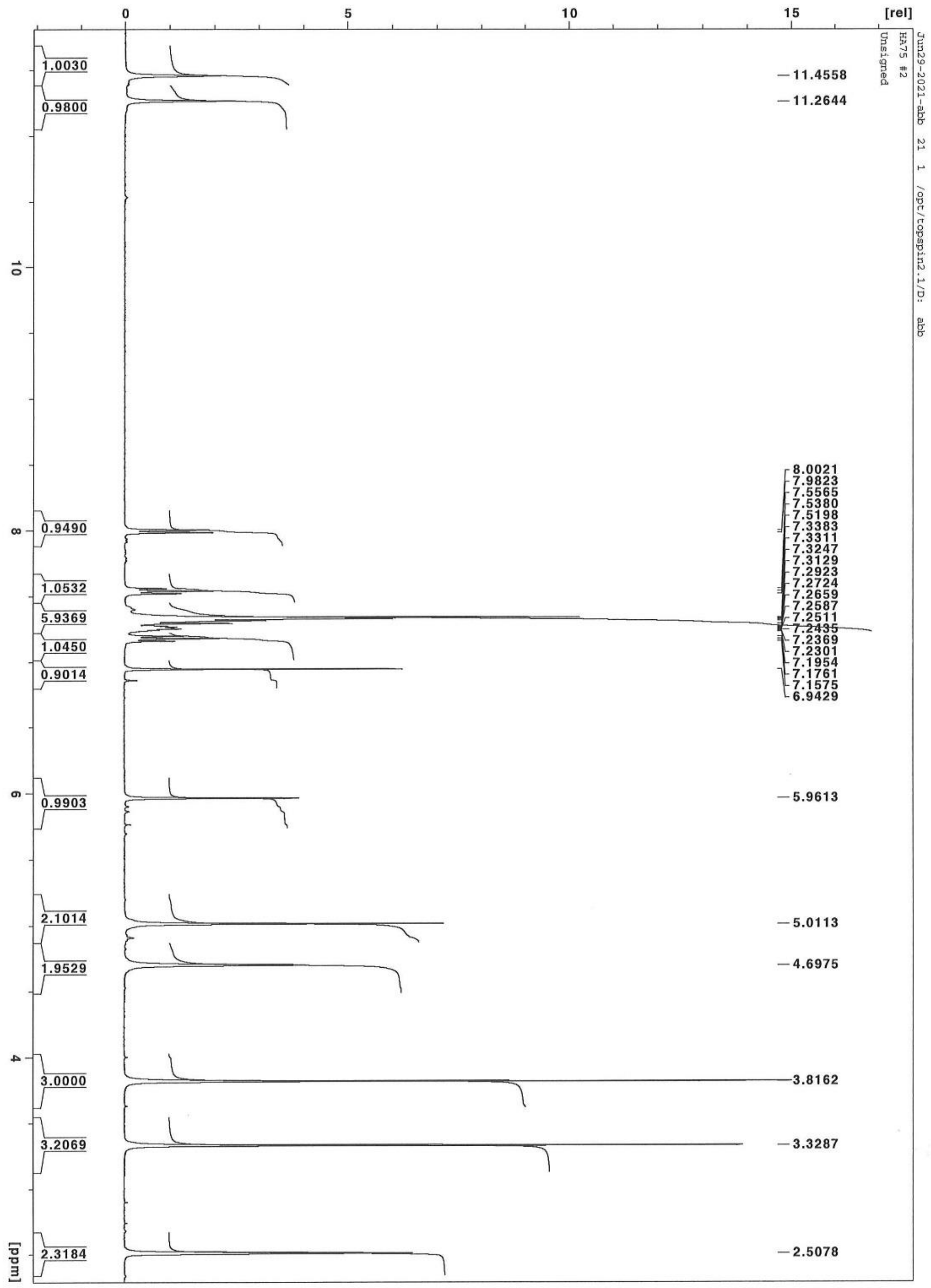

Figure S190. ^{1}H spectrum of compound **7k** (400 MHz, DMSO-d_6).

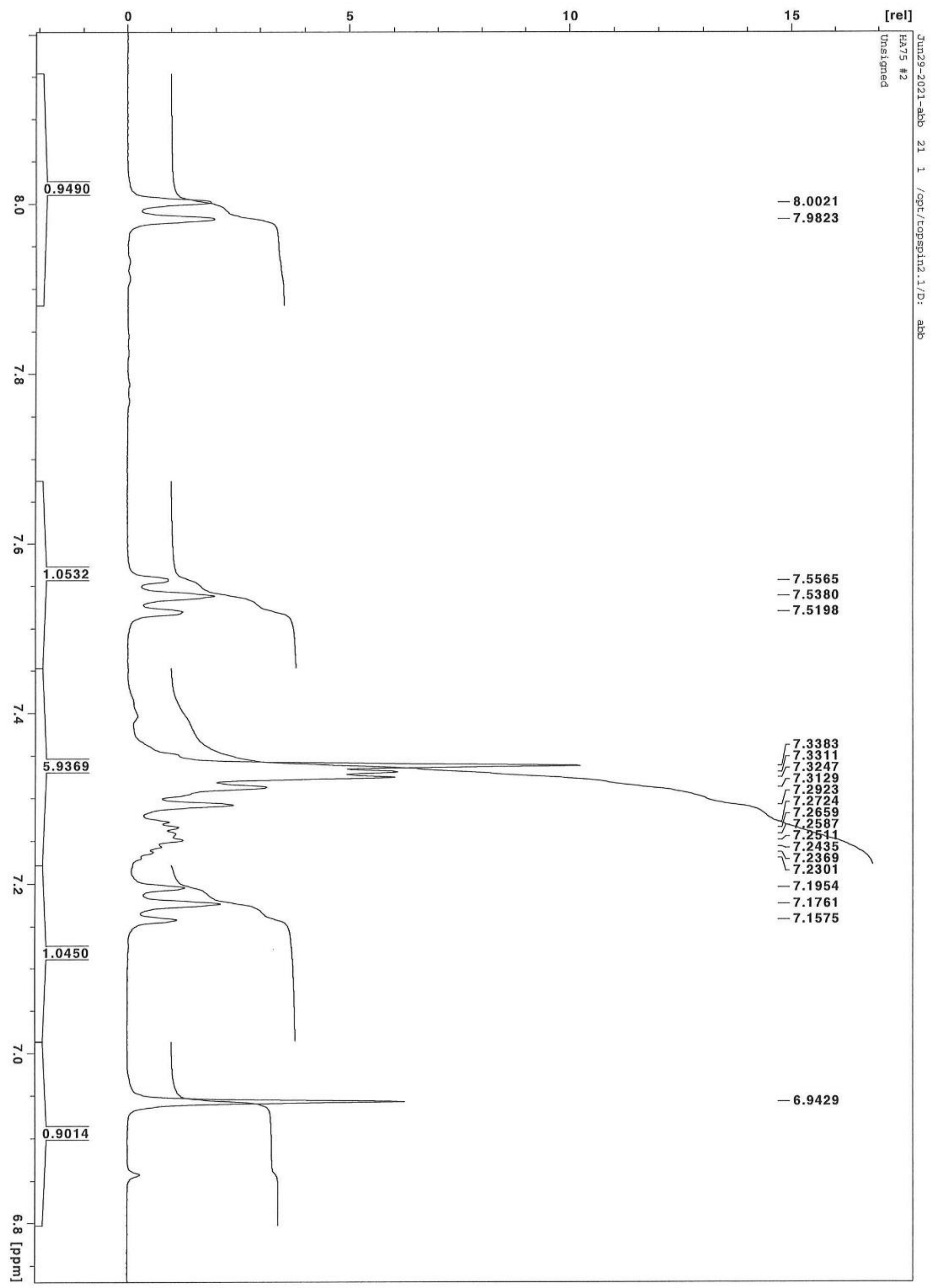

Figure S191. Detail of ^{1}H NMR spectrum of compound **7k.**

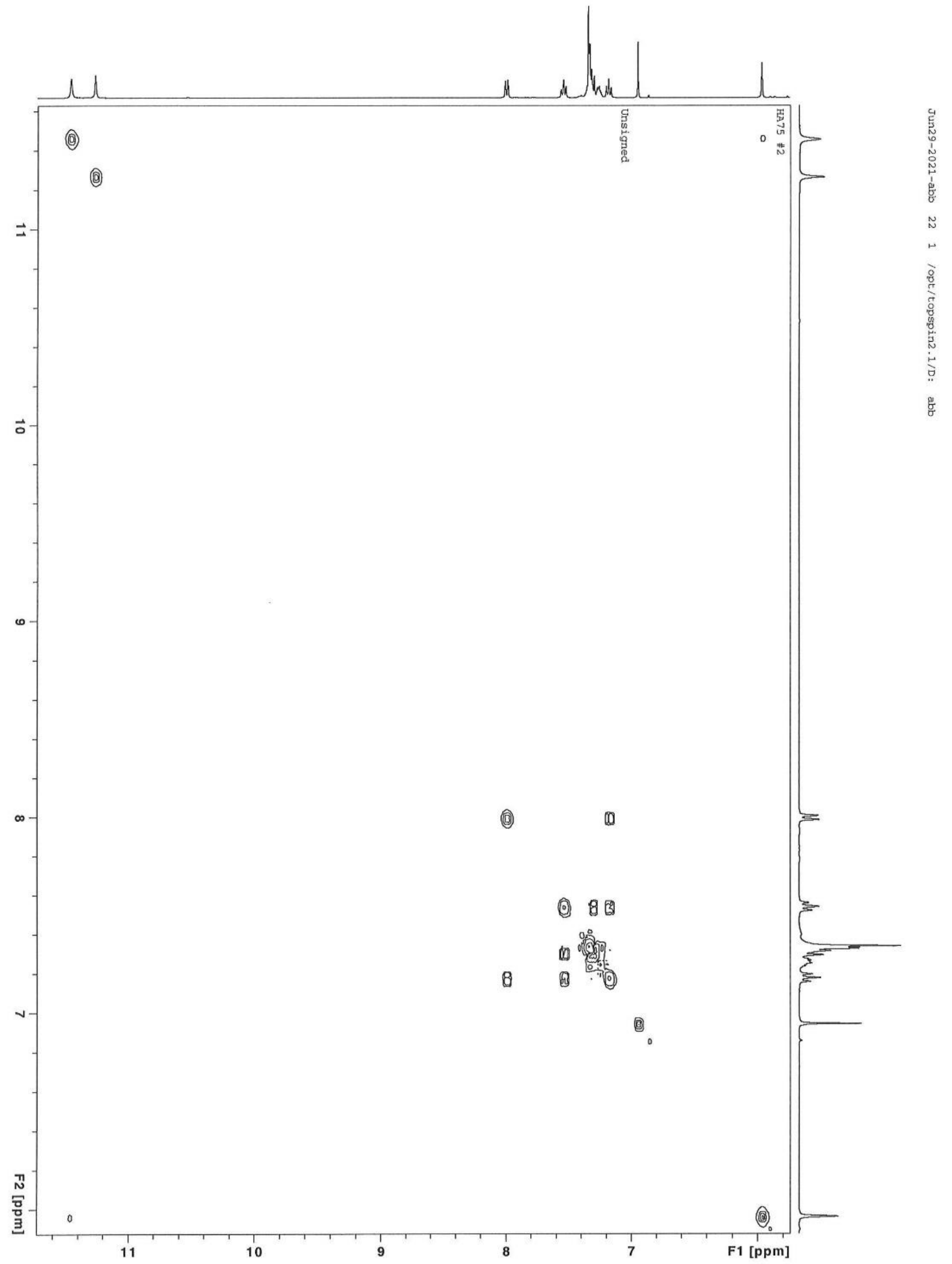

Figure S192. ^{1}H-^{1}H COSY spectrum of compound **7k.**

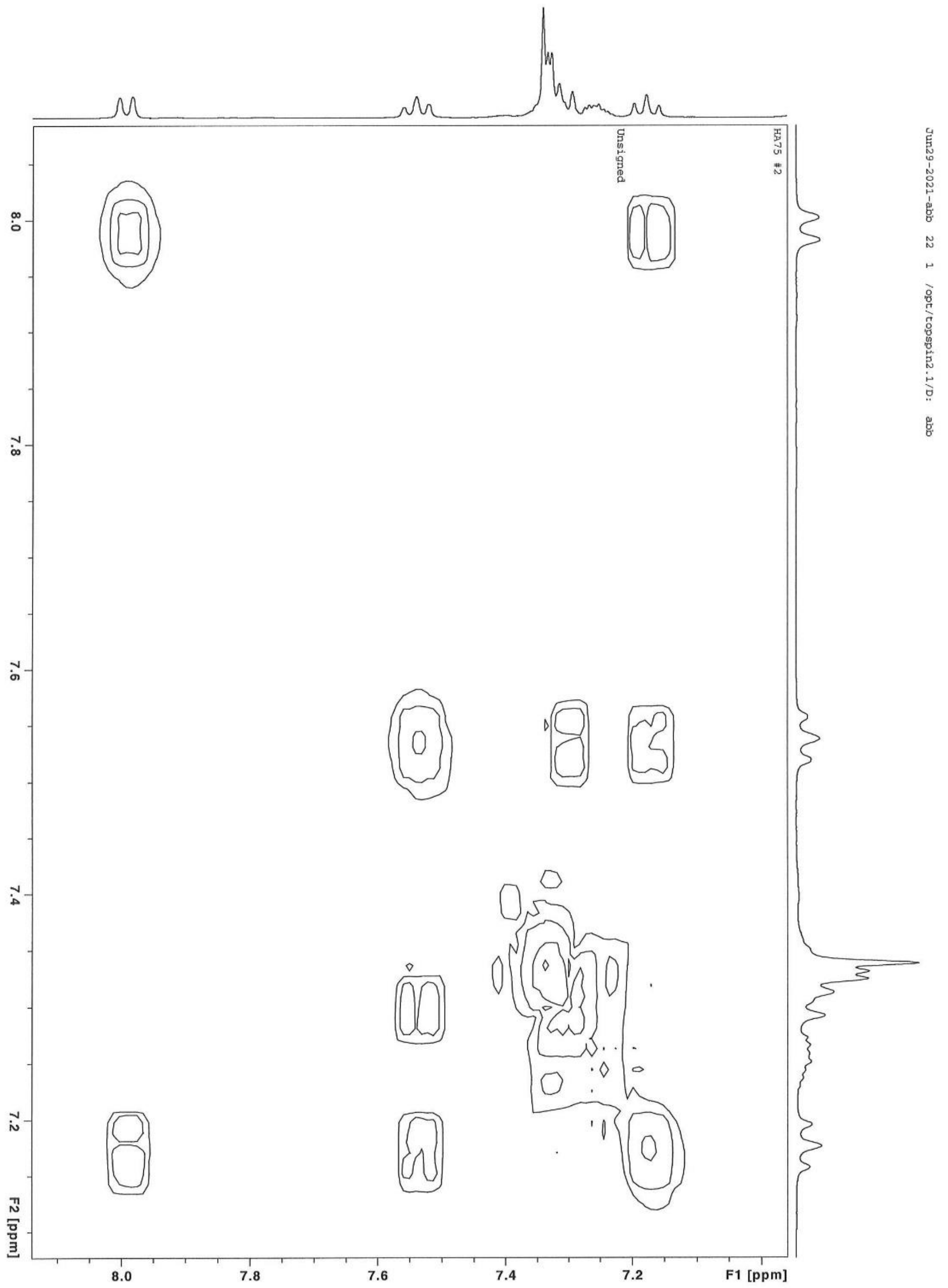

Figure S193. Detail of ^{1}H-^{1}H COSY spectrum of compound **7k.**

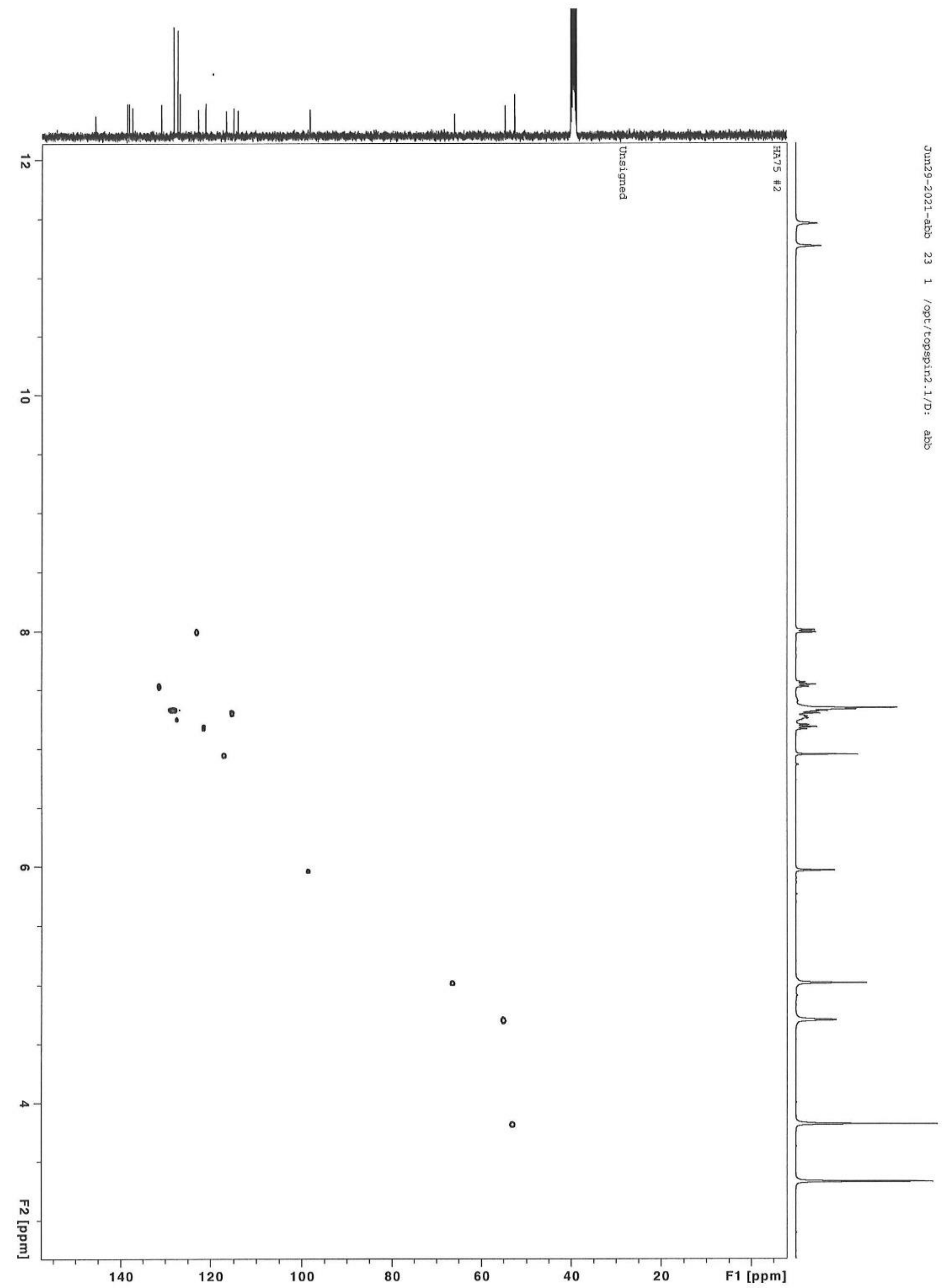

Figure S194. ^{1}H-^{13}C HSQC spectrum of compound **7k.**

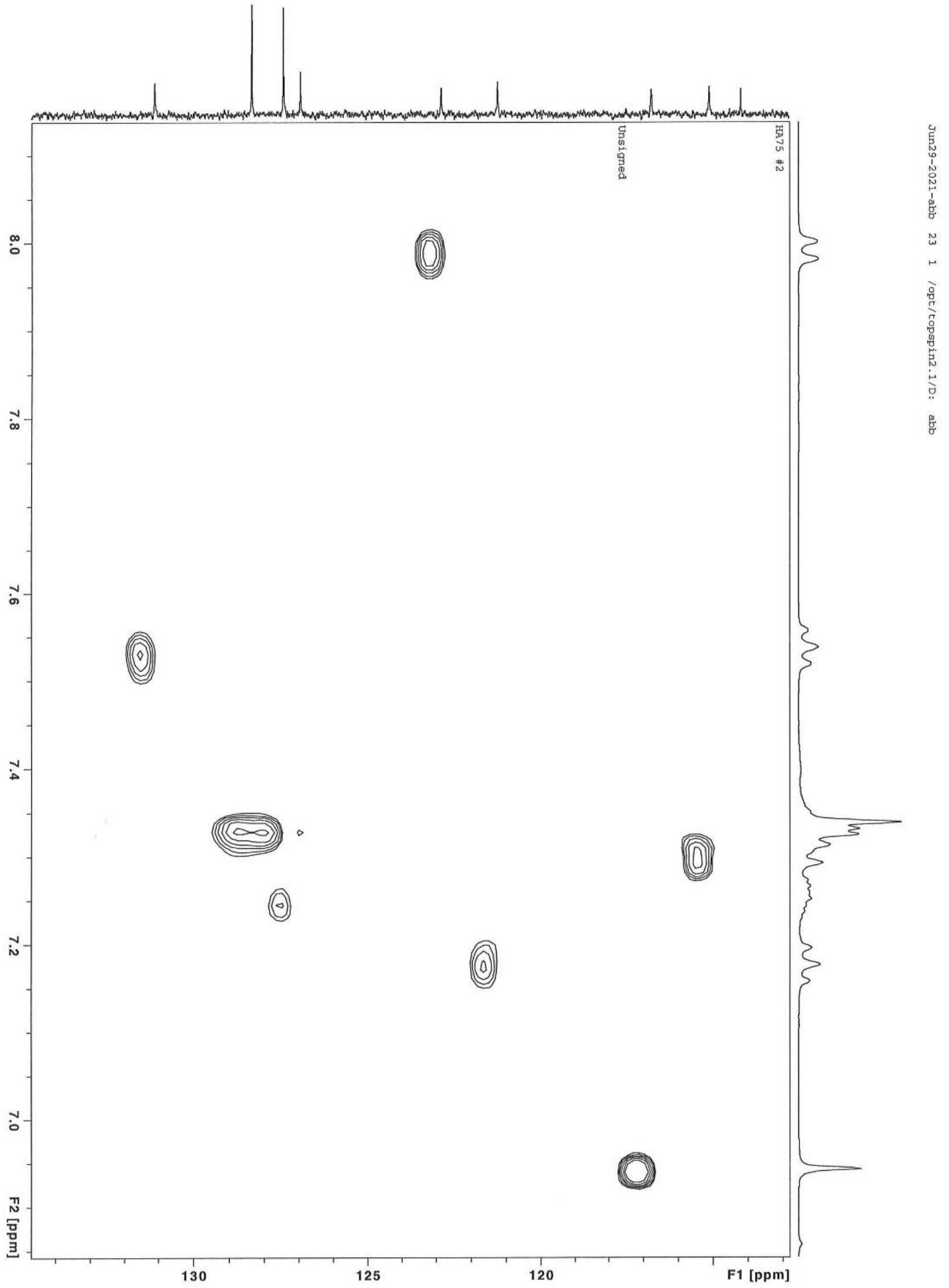

Figure S195. Detail of ^{1}H-^{13}C HSQC spectrum of compound **7k.**

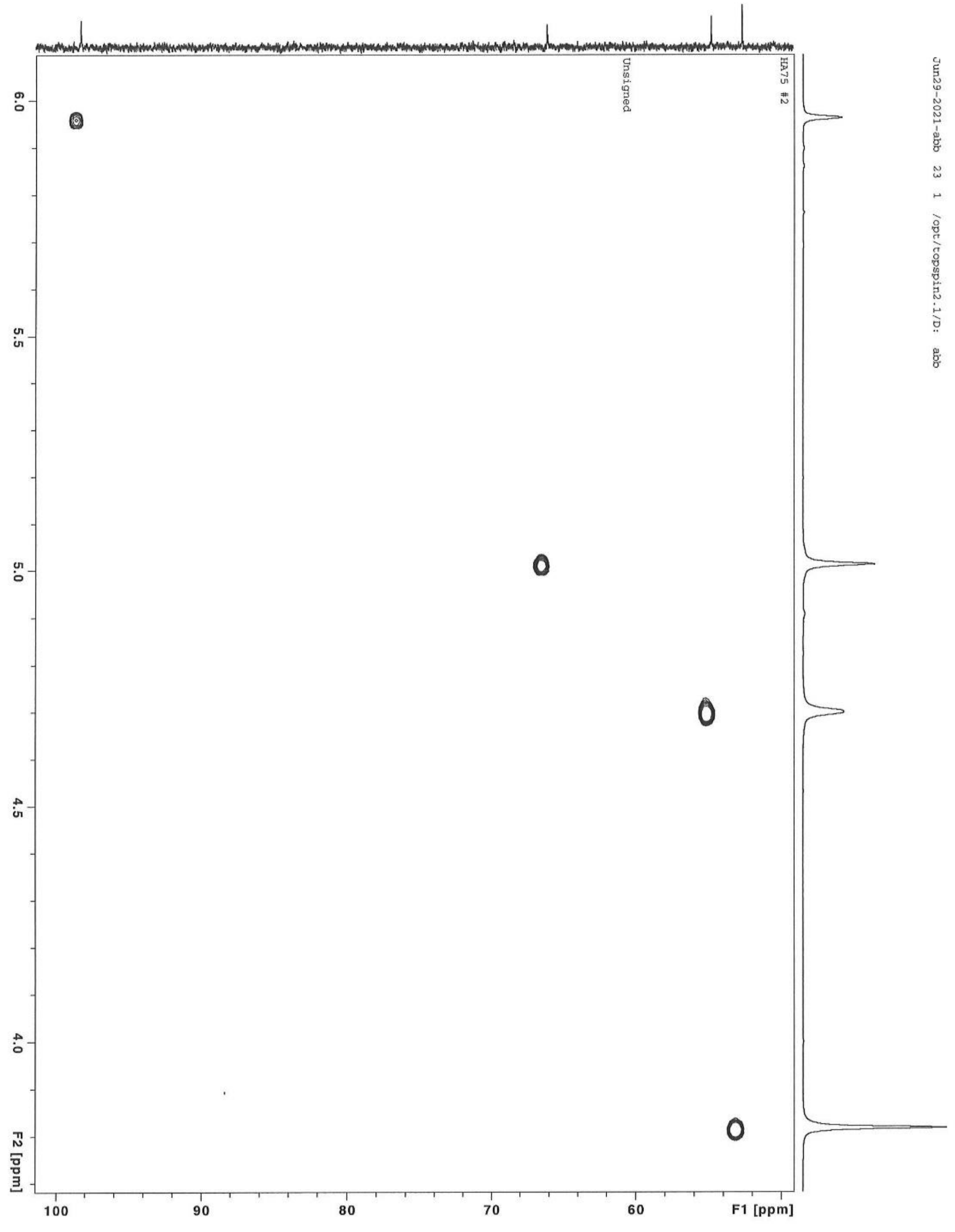

Figure S196. Detail of ^{1}H-^{13}C HSQC spectrum of compound **7k.**

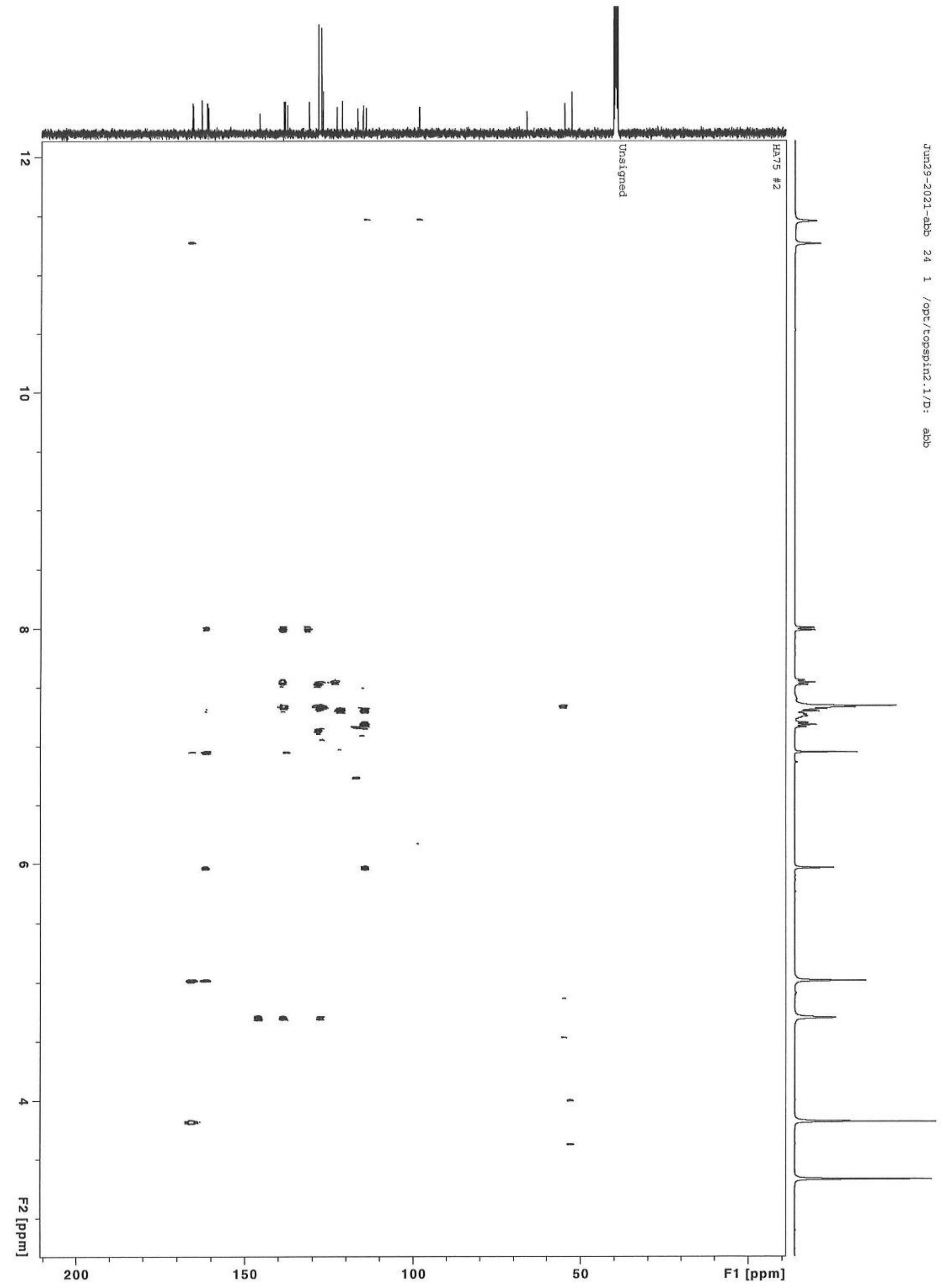

Figure S197. ^{1}H-^{13}C HMBC spectrum of sample **7k.**

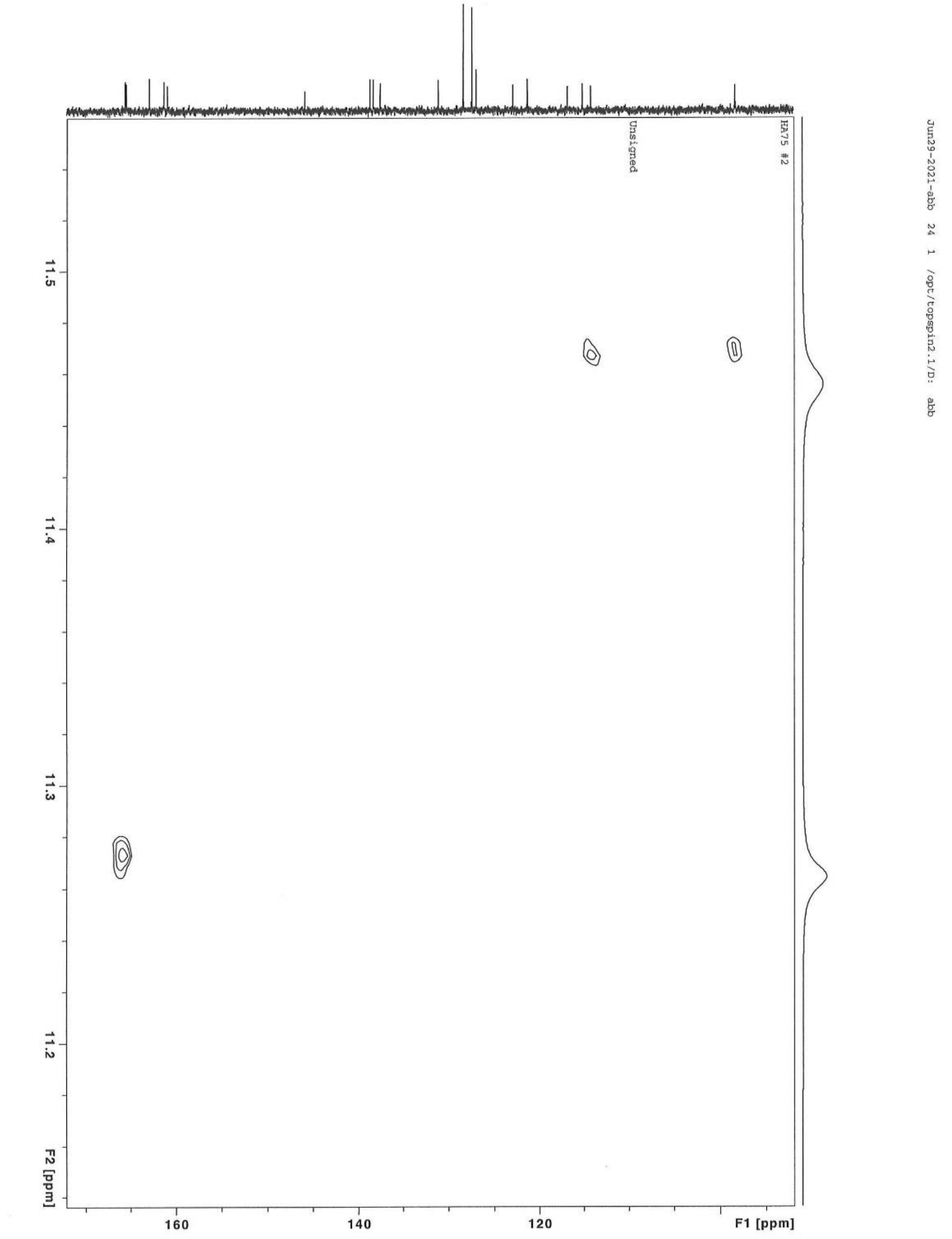

Figure S198. Detail of ^{1}H-^{13}C HMBC spectrum of compound **7k.**

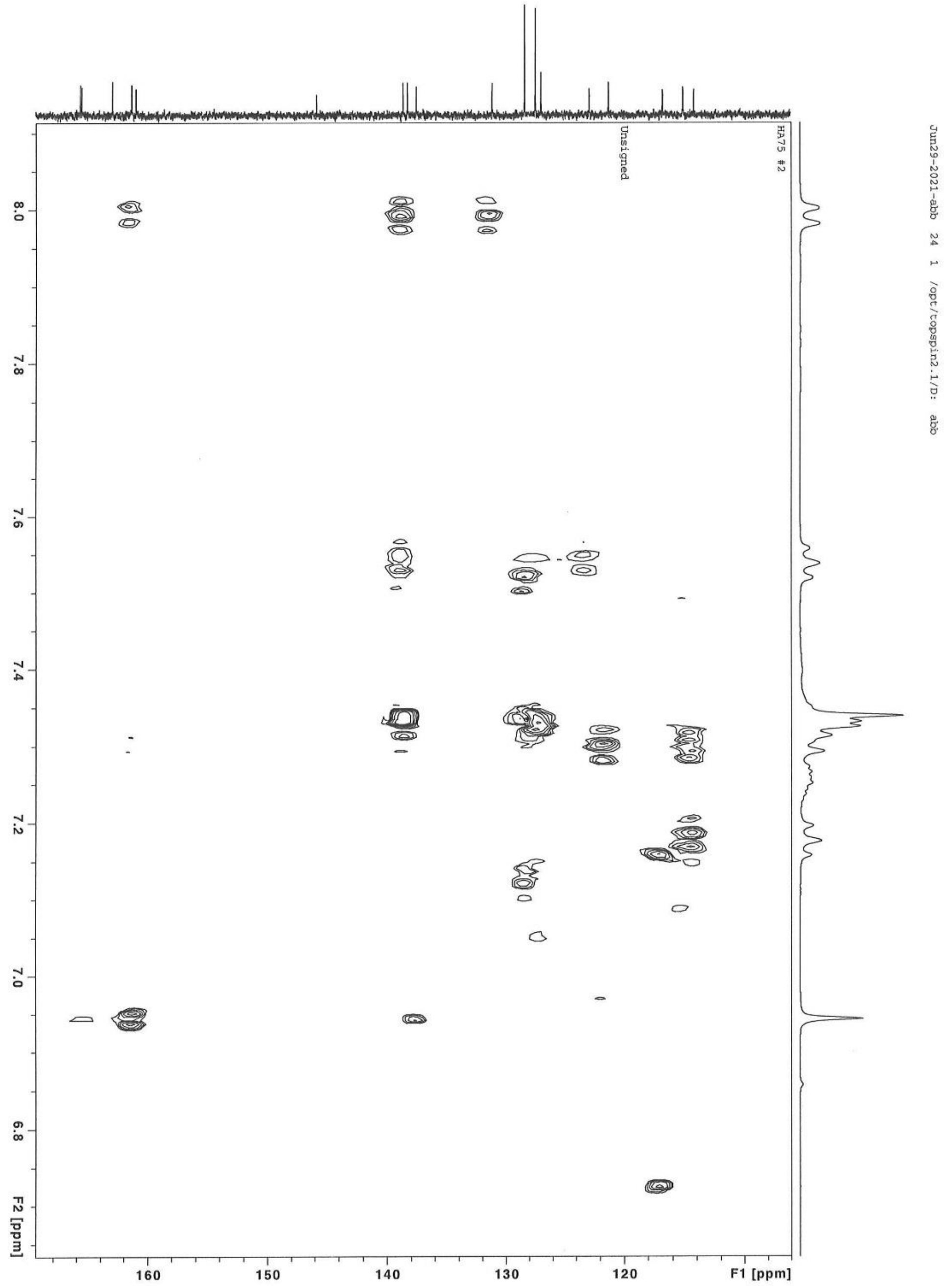

Figure S199. Detail of ^{1}H-^{13}C HMBC spectrum of compound **7k.**

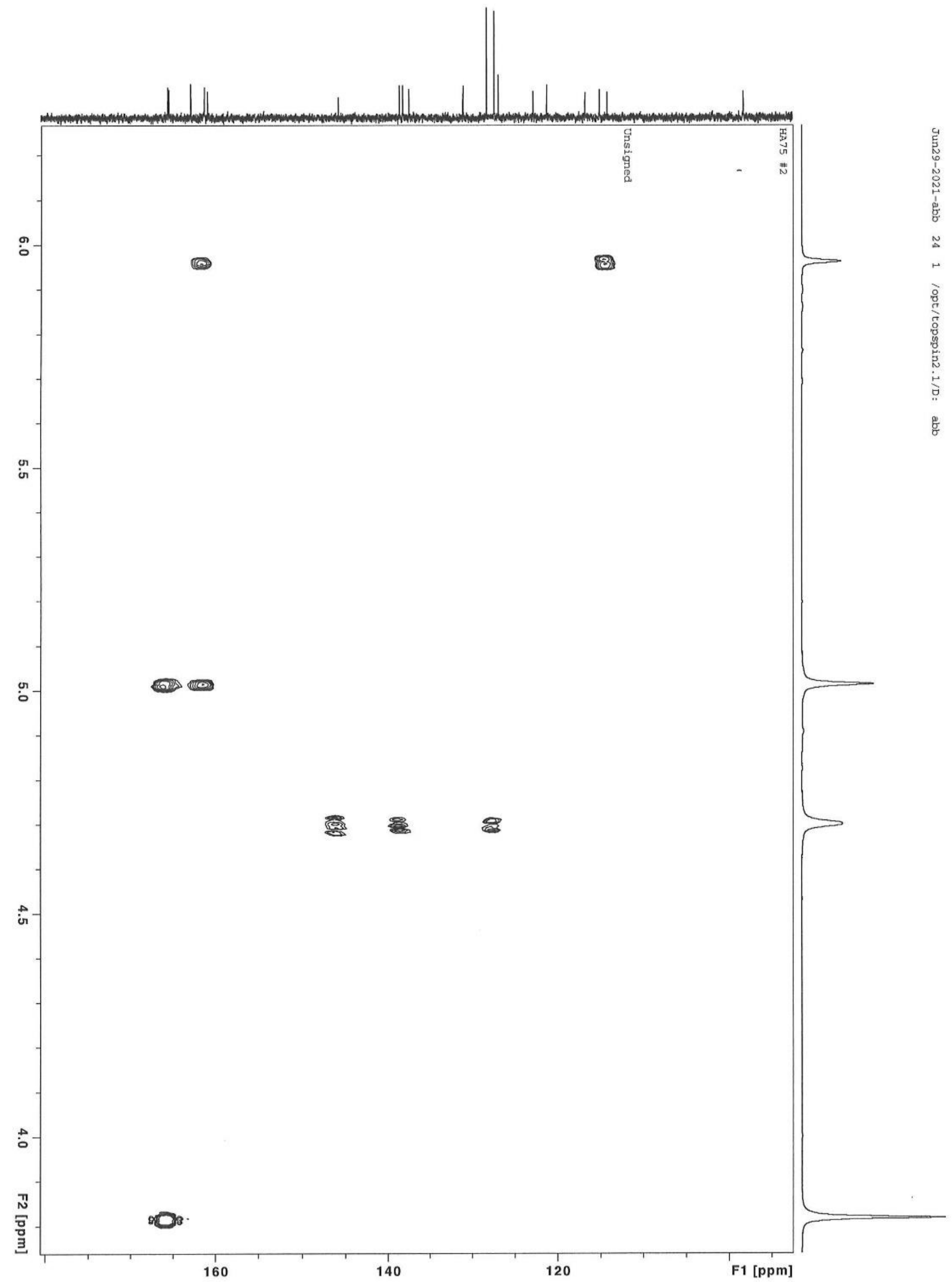

Figure S200. Detail of ^{1}H-^{13}C HMBC spectrum of compound **7k.**

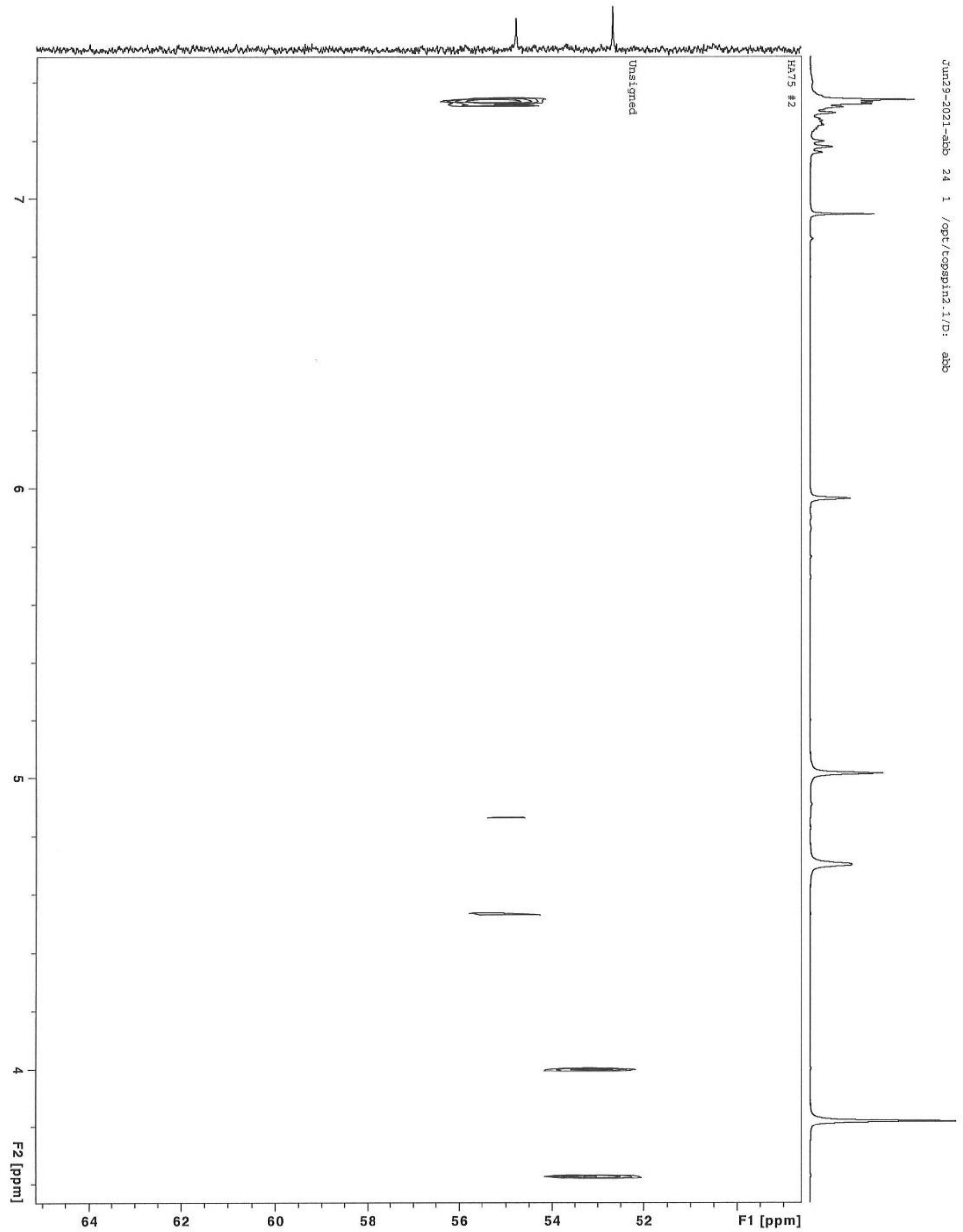

Figure S201. Detail of ^{1}H-^{13}C HMBC spectrum of compound **7k.**

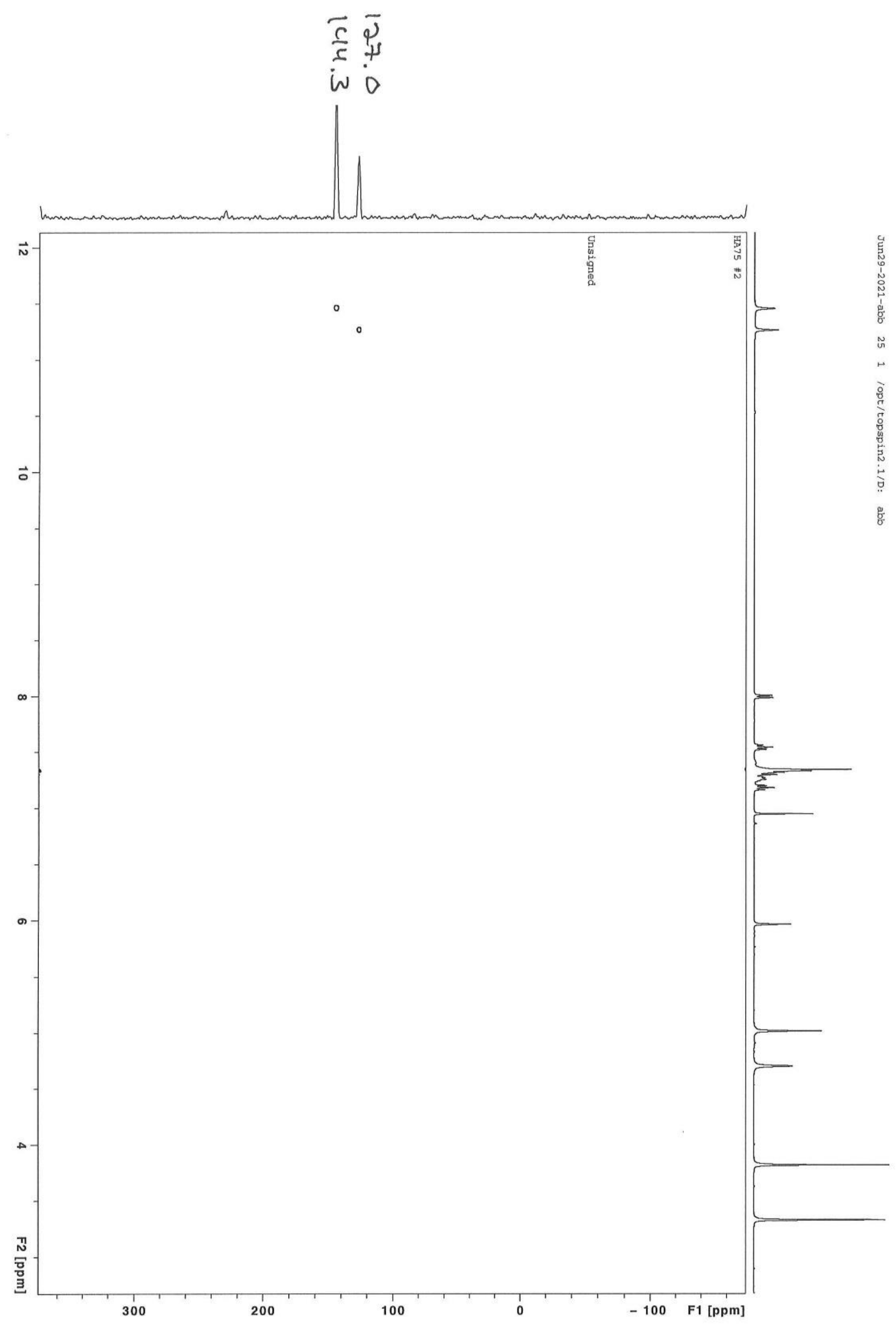

Figure S202. ^{1}H-^{15}N HSQC spectrum of compound **7k.**

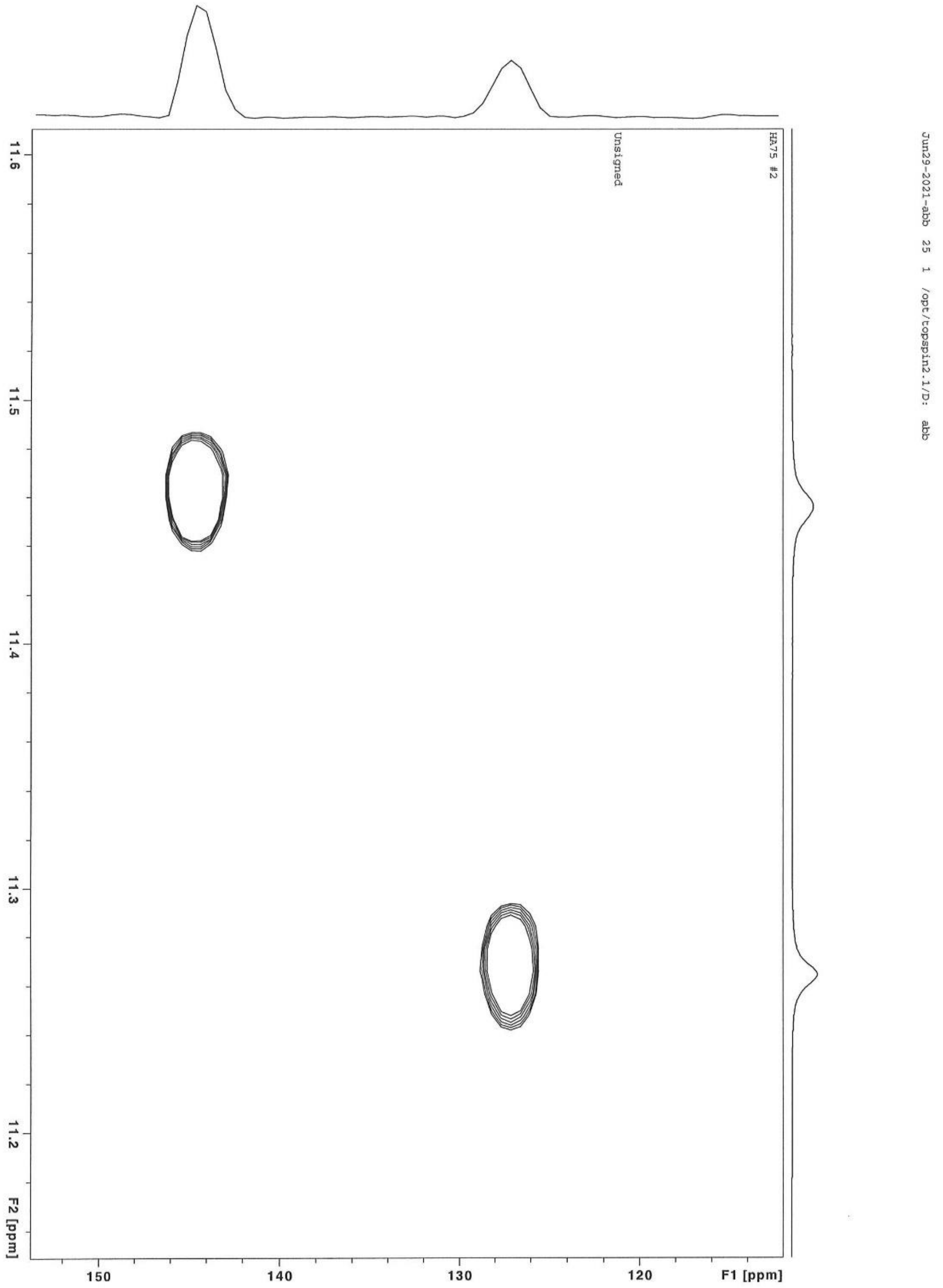

Figure S203. Detail of ^{1}H-^{15}N HSQC spectrum of compound **7k.**

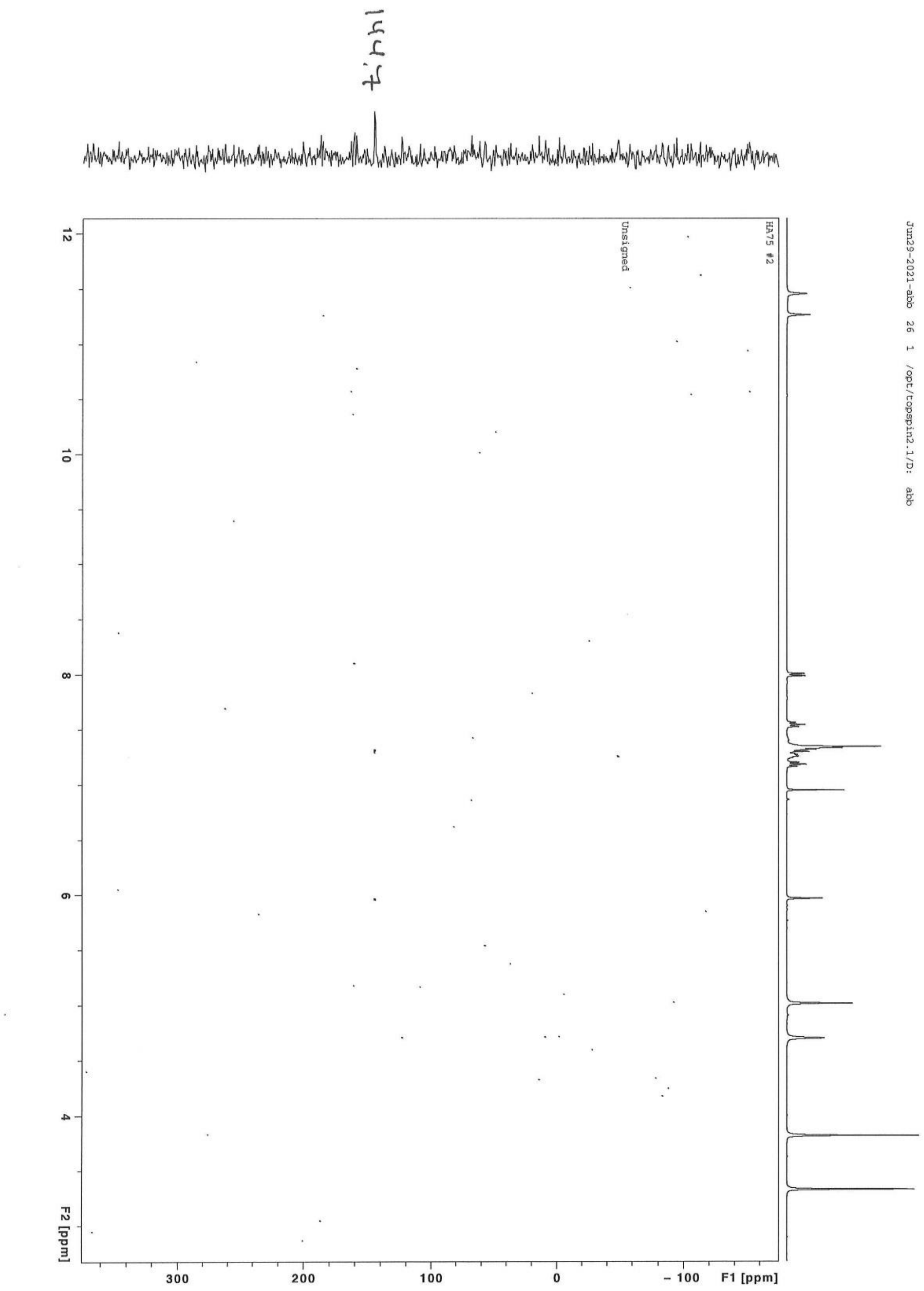

Figure S204. ^{1}H-^{15}N HMBC spectrum of compound **7k.**

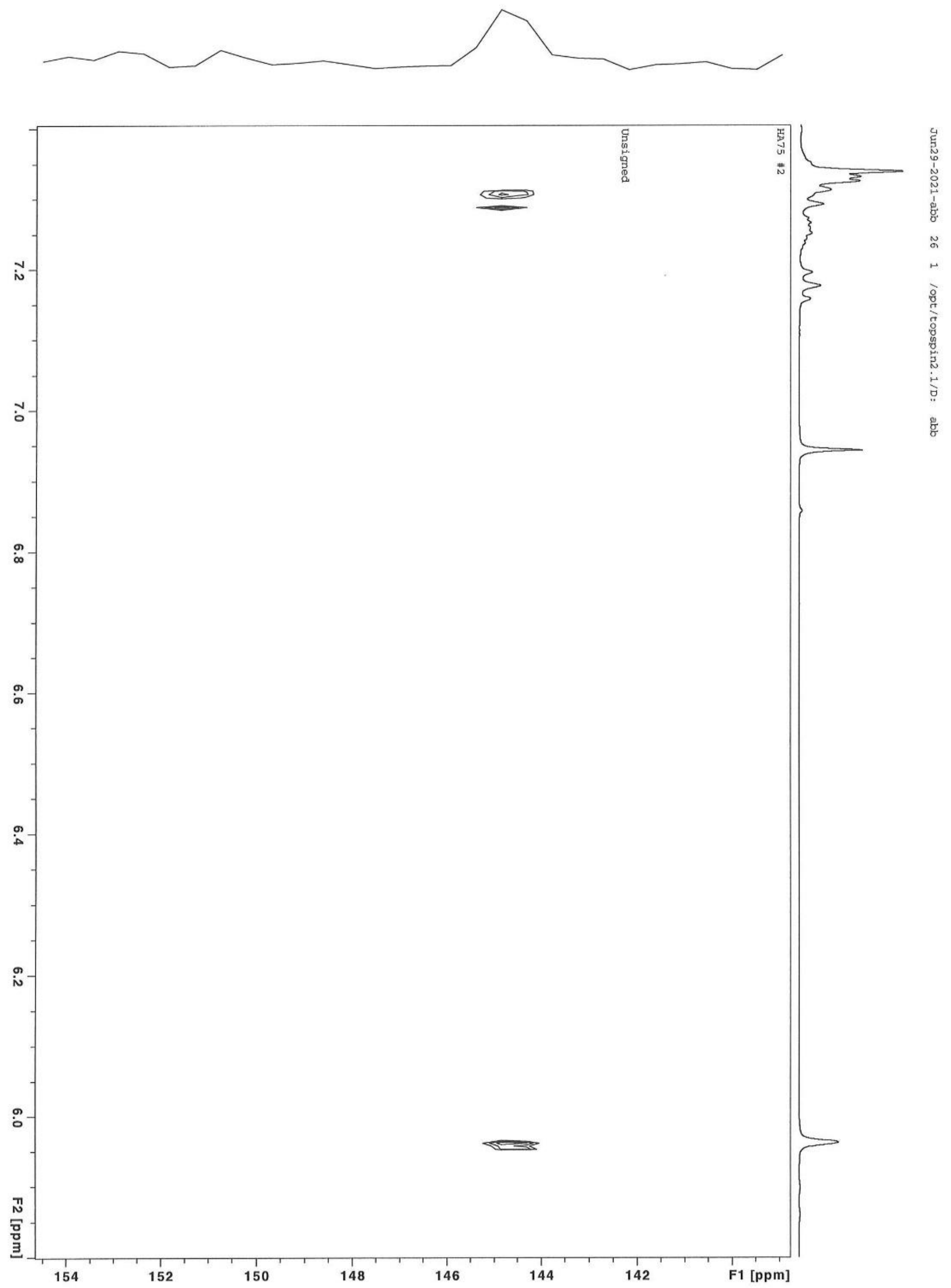

Figure S205. Detail of ^{1}H-^{15}N HMBC spectrum of compound **7k.**

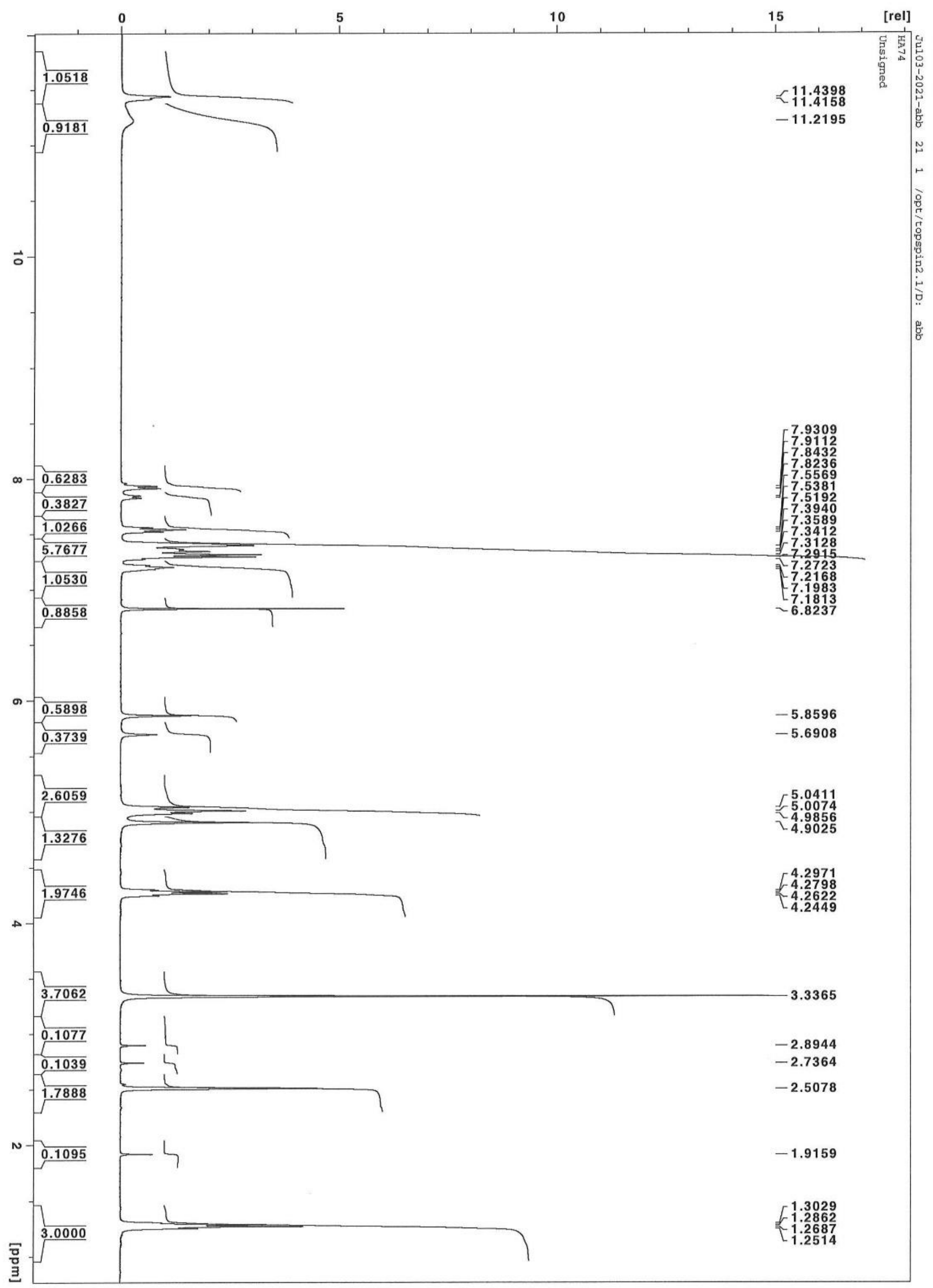

Figure S206. ^{1}H NMR spectrum of compound **7l** (400 MHz, DMSO-d_6).

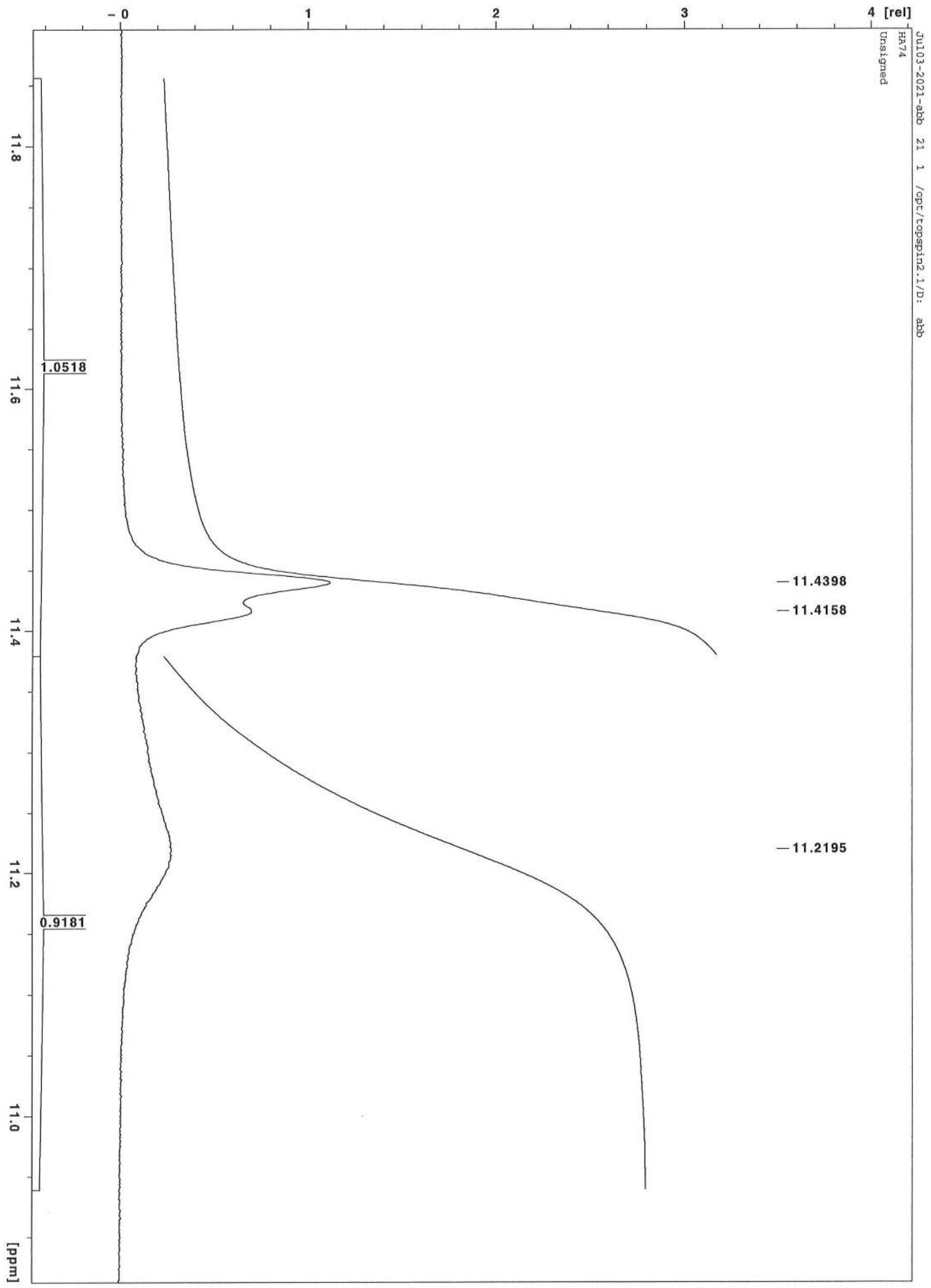

Figure S207. Detail of ^{1}H NMR spectrum of compound **7l.**

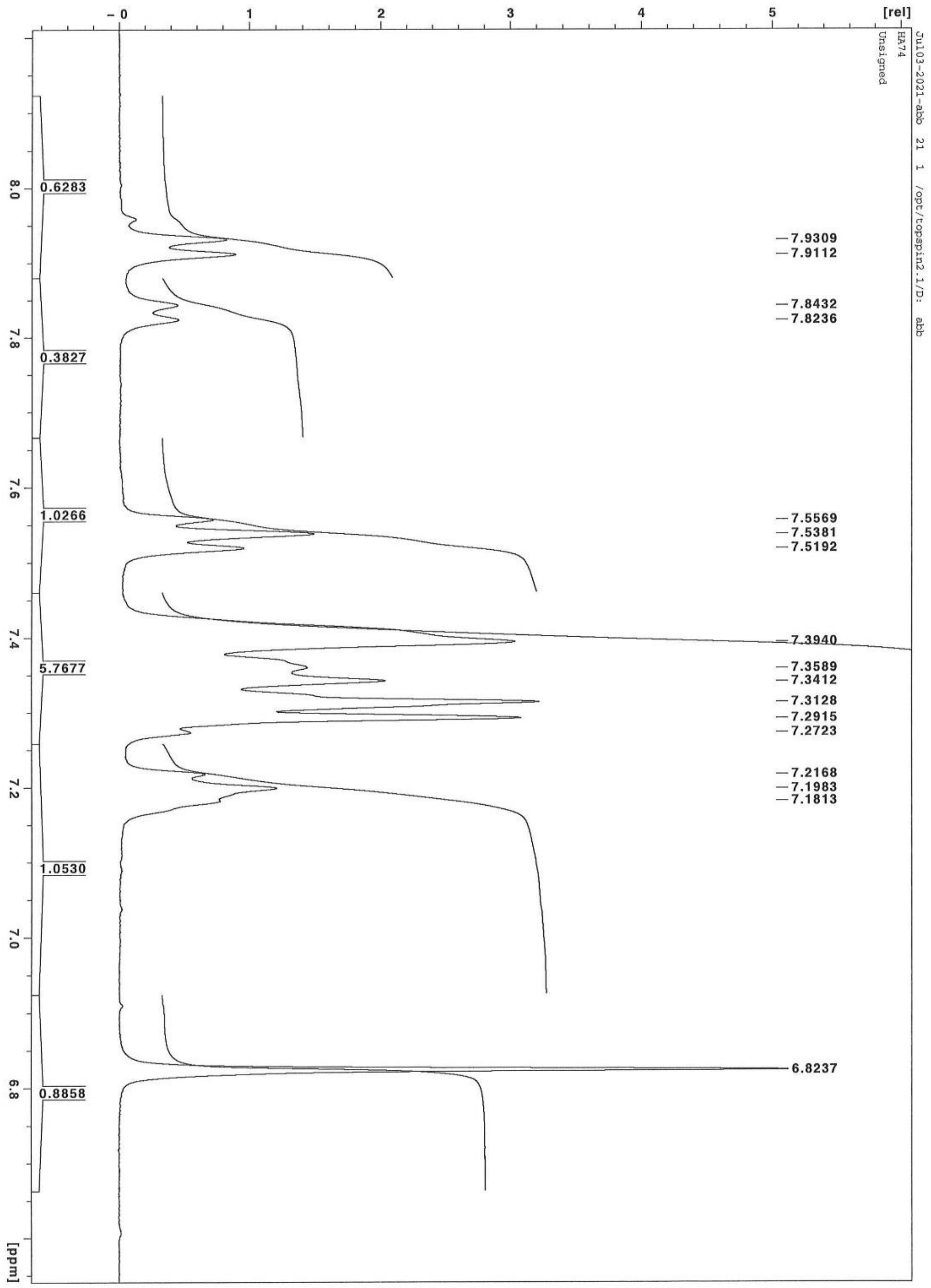

Figure S208. Detail of ^{1}H NMR spectrum of compound **7l.**

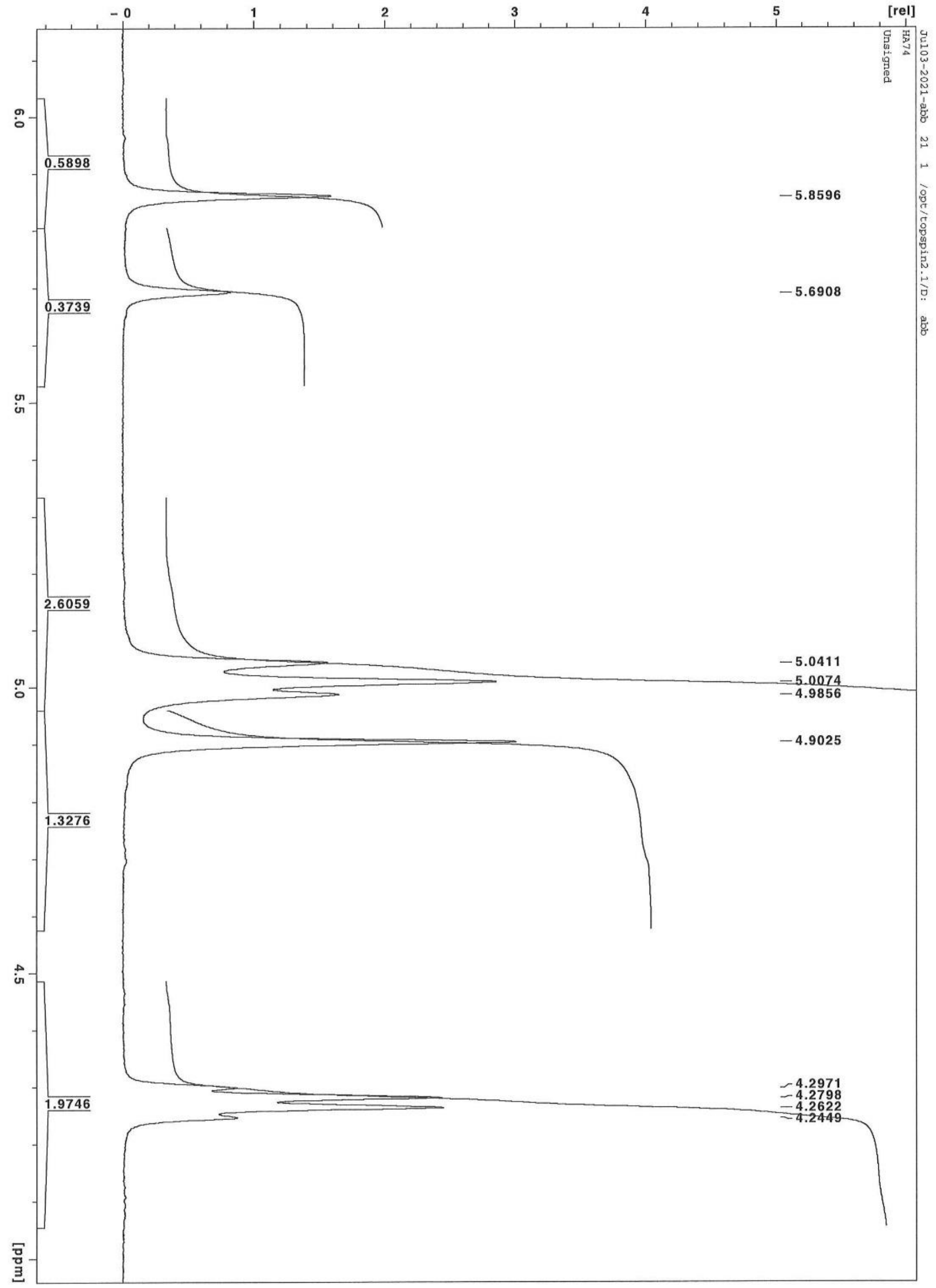

Figure S209. Detail of ¹H NMR spectrum of compound **7l.**

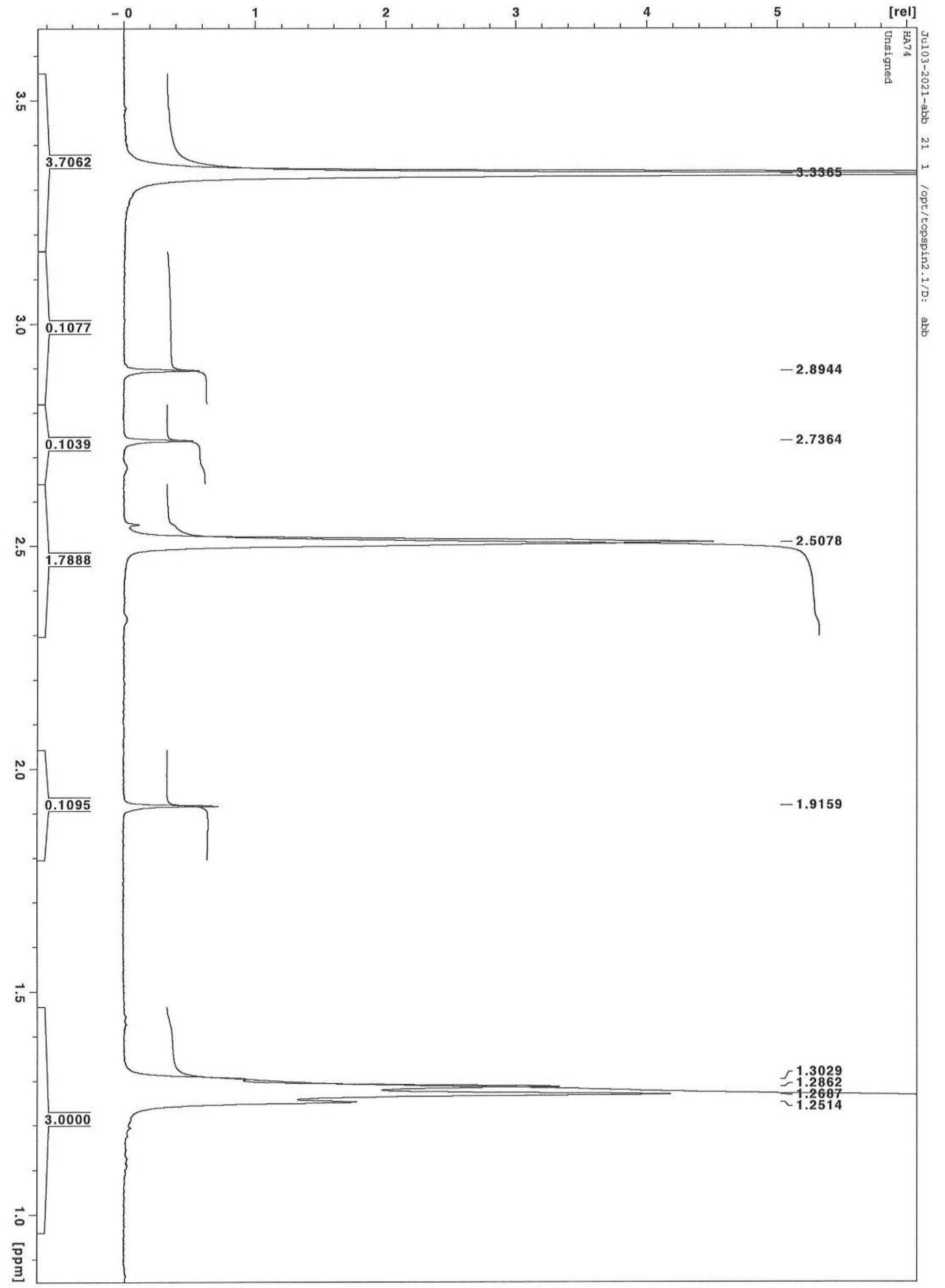

Figure S210. Detail of ^{1}H NMR spectrum of compound **7l.**

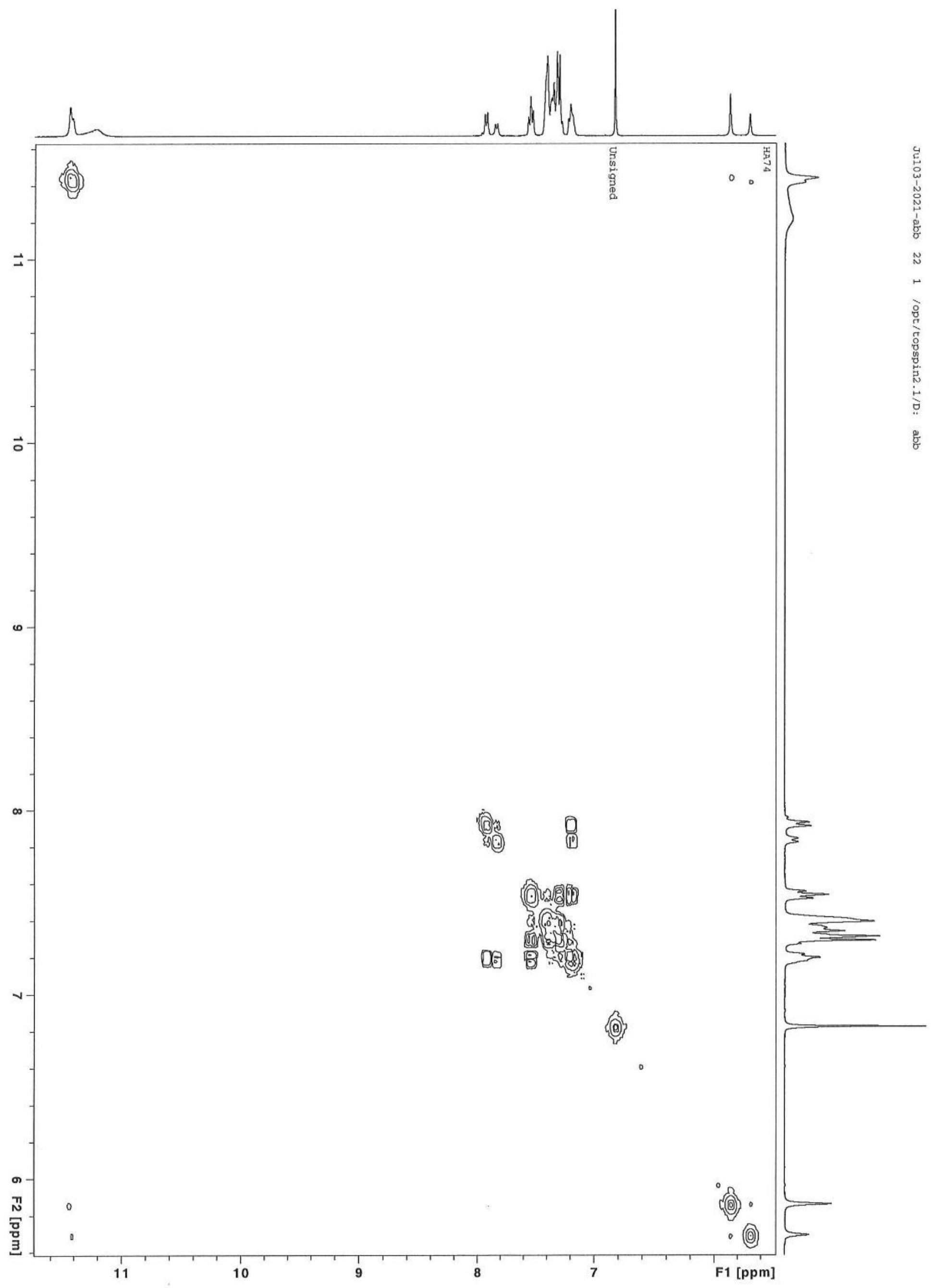

Figure S211. ^{1}H-^{1}H COSY spectrum of compound **7l.**

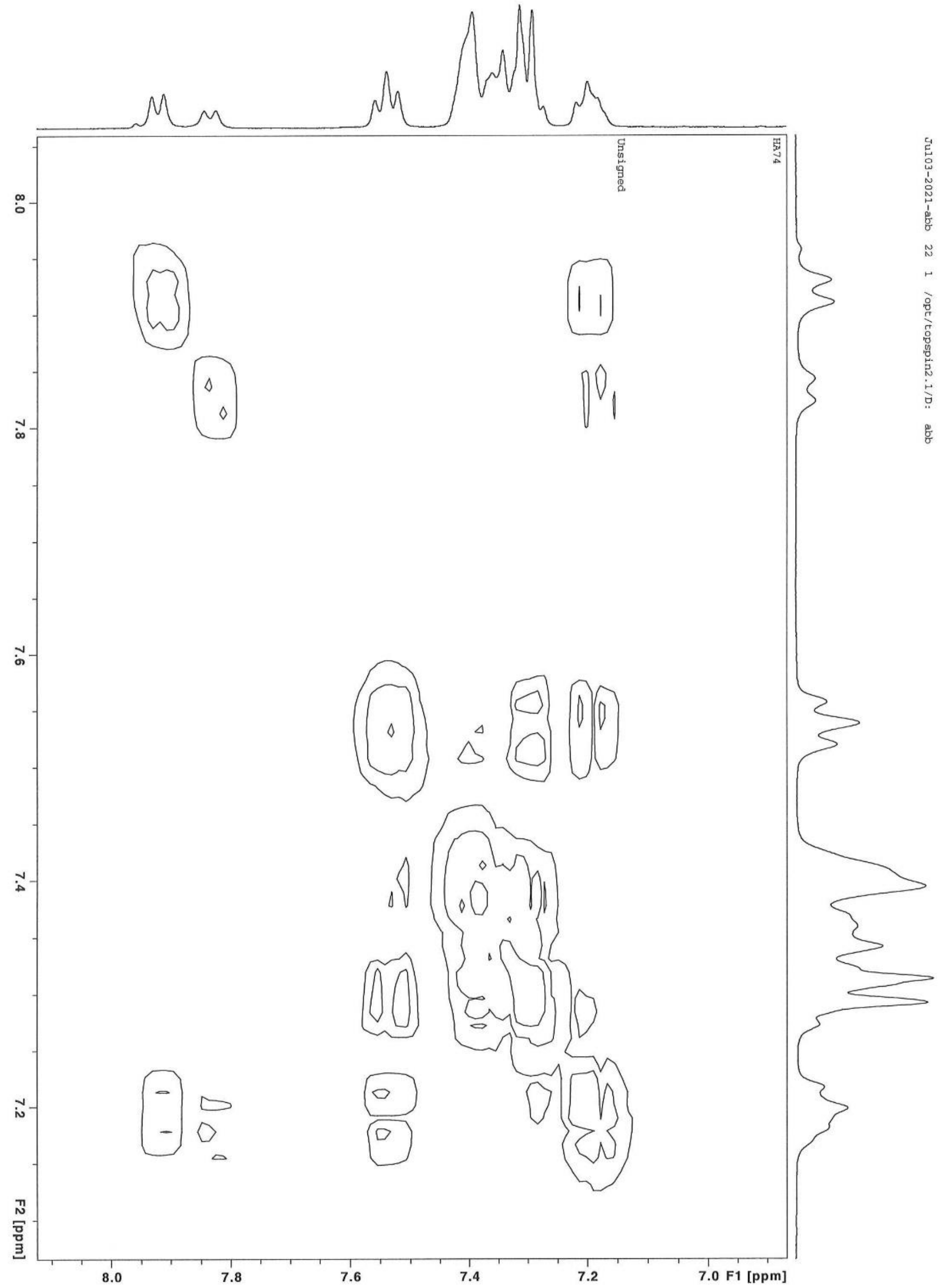

Figure S212. Detail of ^{1}H-^{1}H COSY spectrum of compound **7l.**

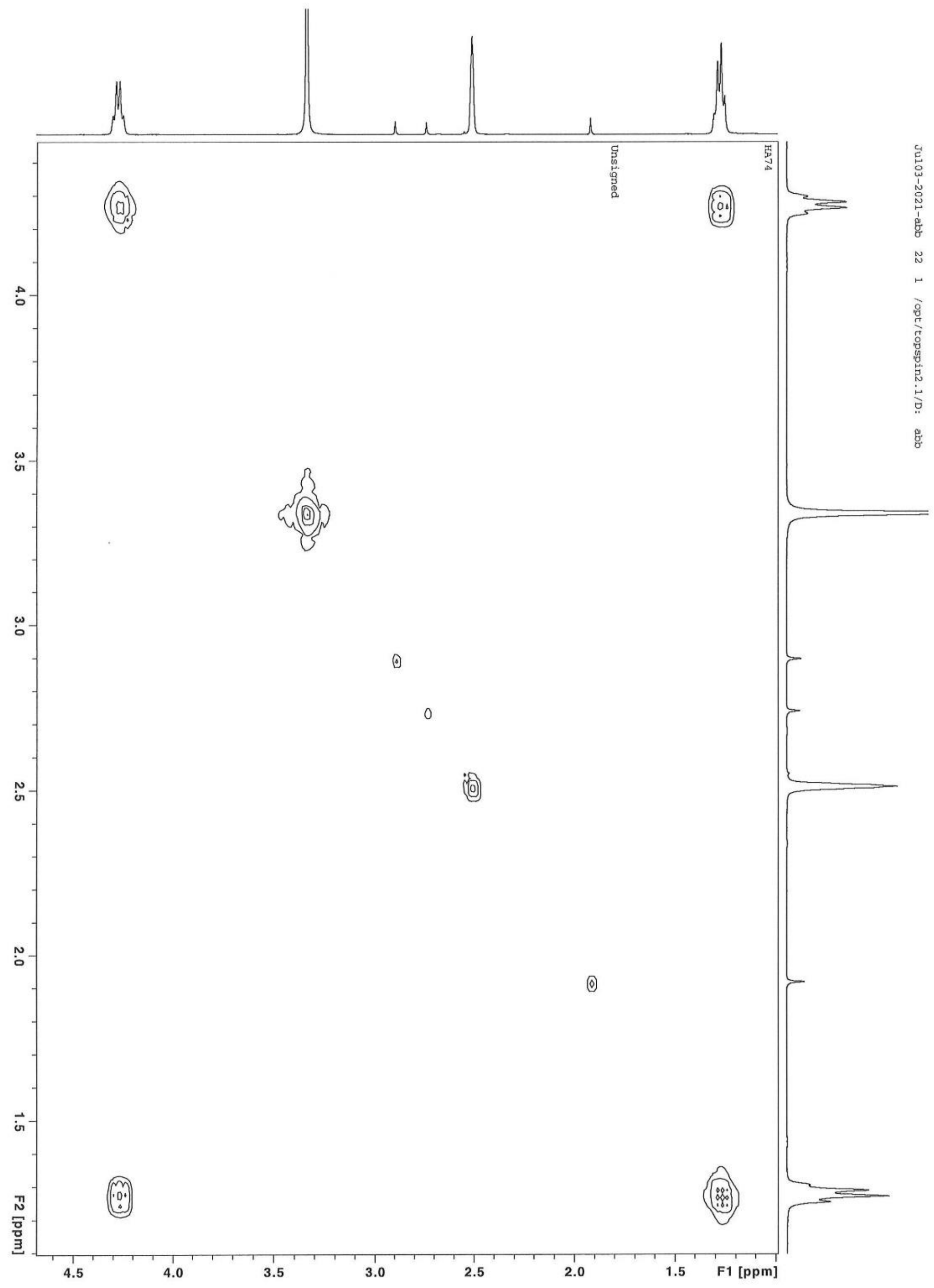

Figure S213. Detail of ^{1}H-^{1}H COSY spectrum of compound **7l.**

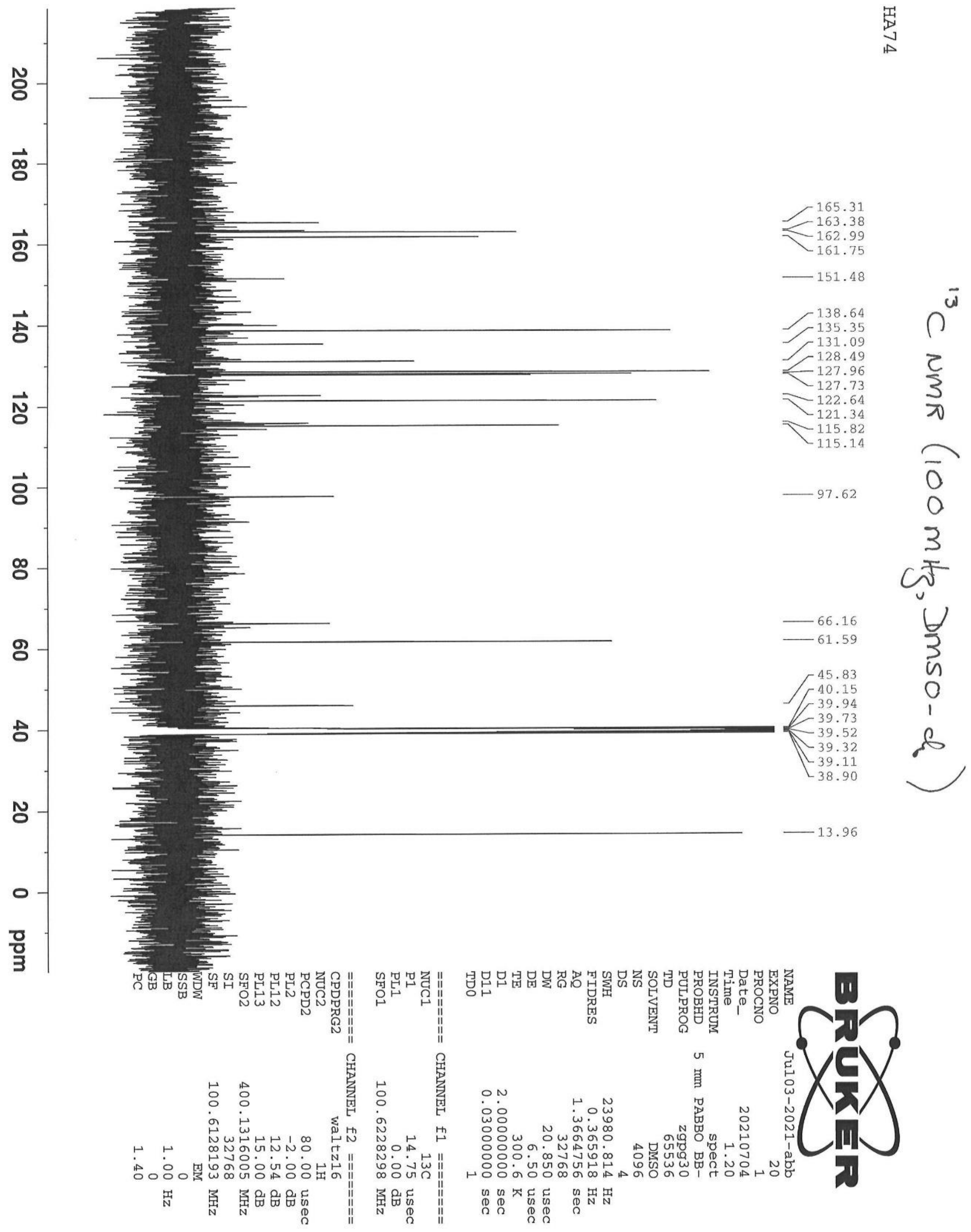

Figure S214. ^{13}C NMR spectrum of compound **7l** (100 MHz, DMSO-d_6)

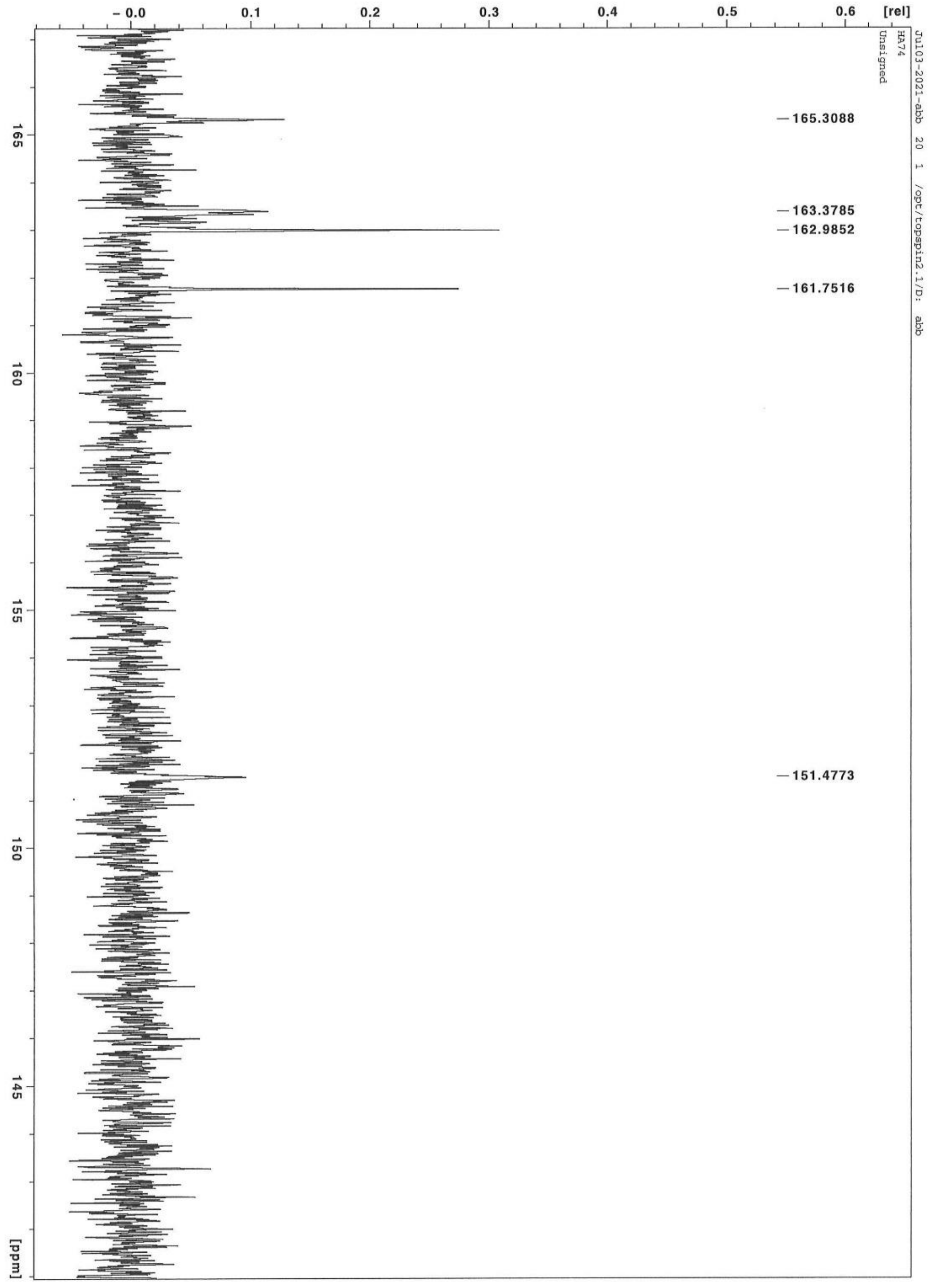

Figure S215. Detail of ^{13}C NMR spectrum of compound **7l.**

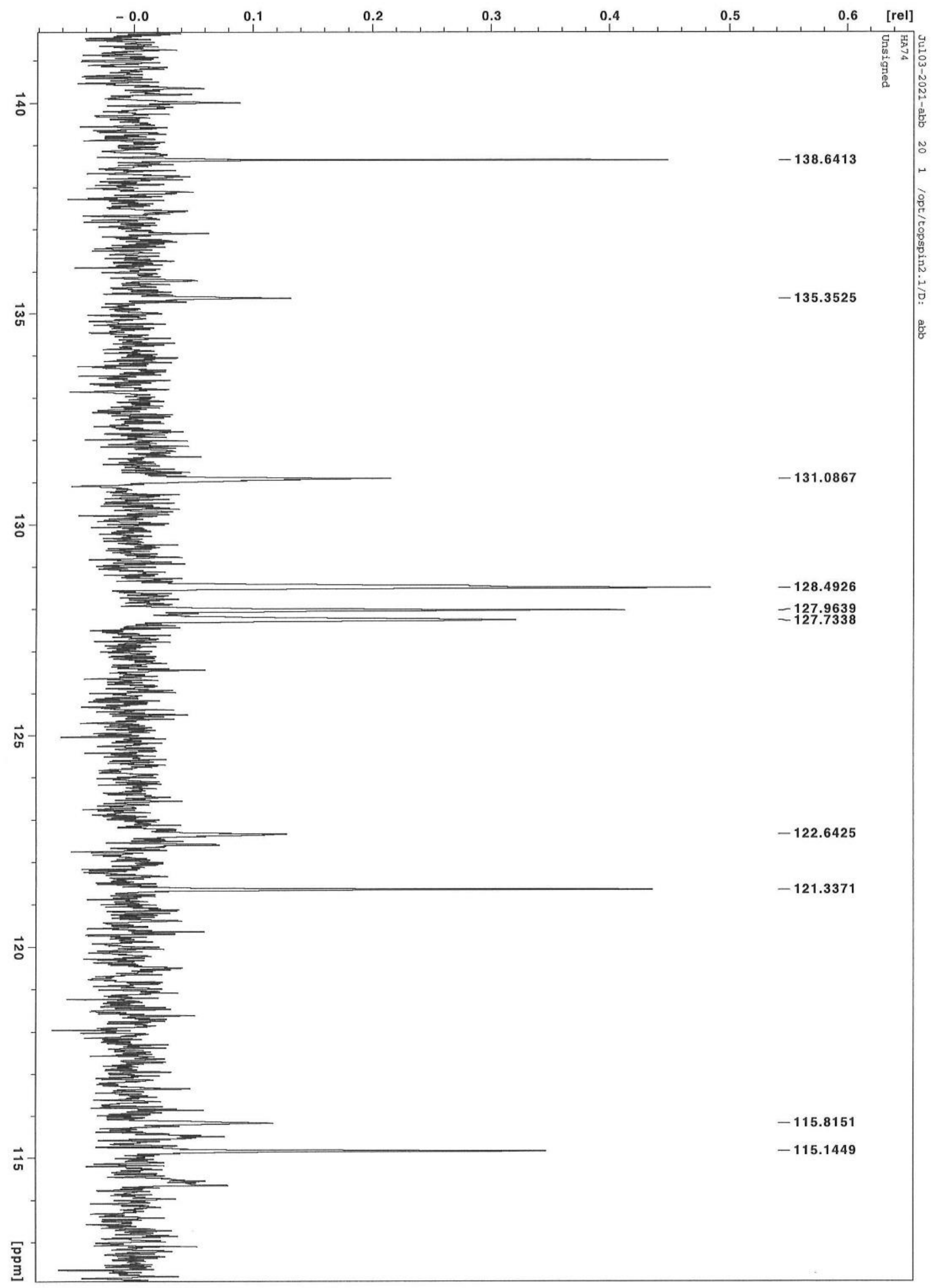

Figure S216. Detail of ^{13}C NMR spectrum of compound **7l.**

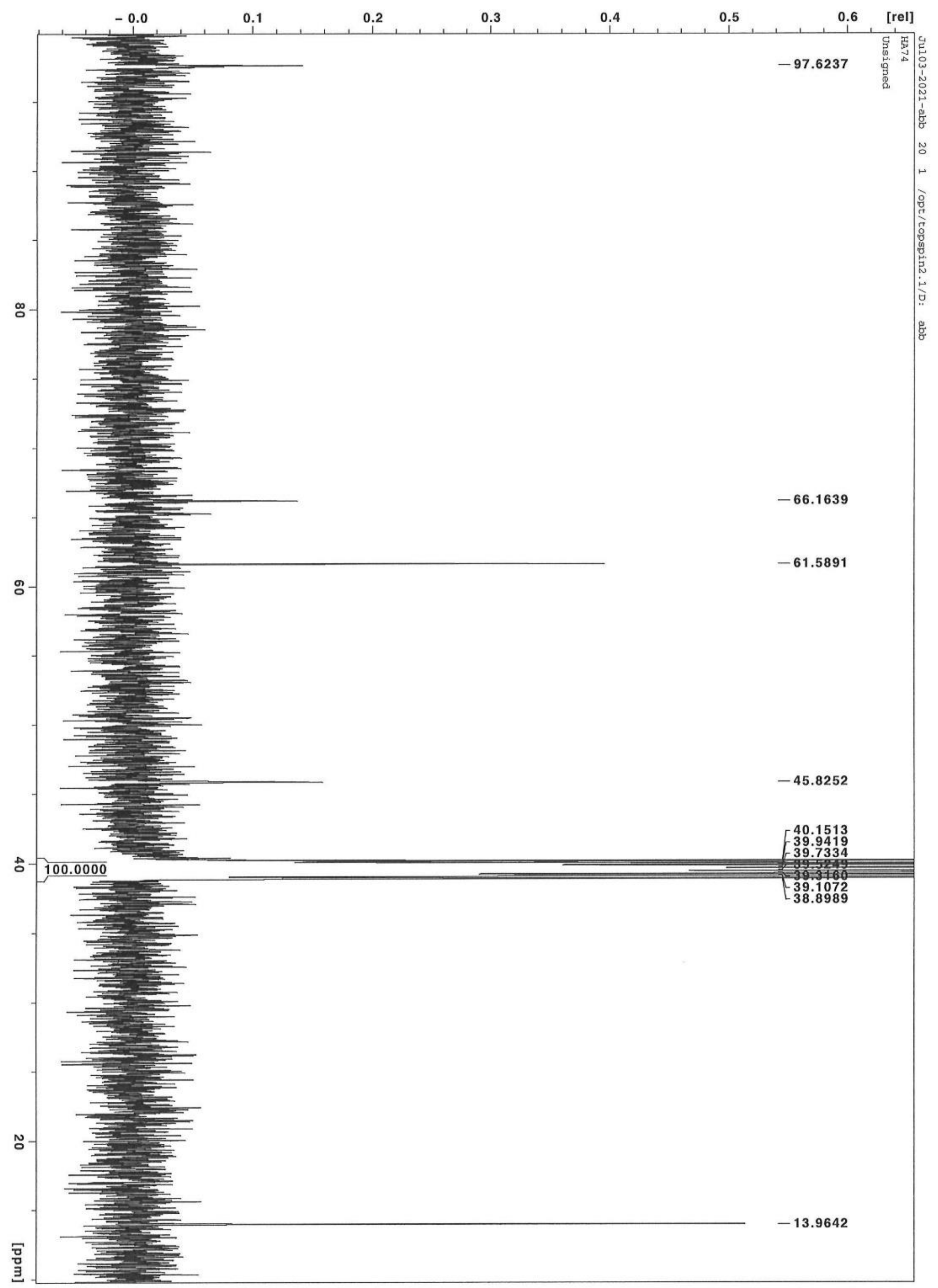

Figure S217. Detail of ^{13}C NMR spectrum of compound **7l.**

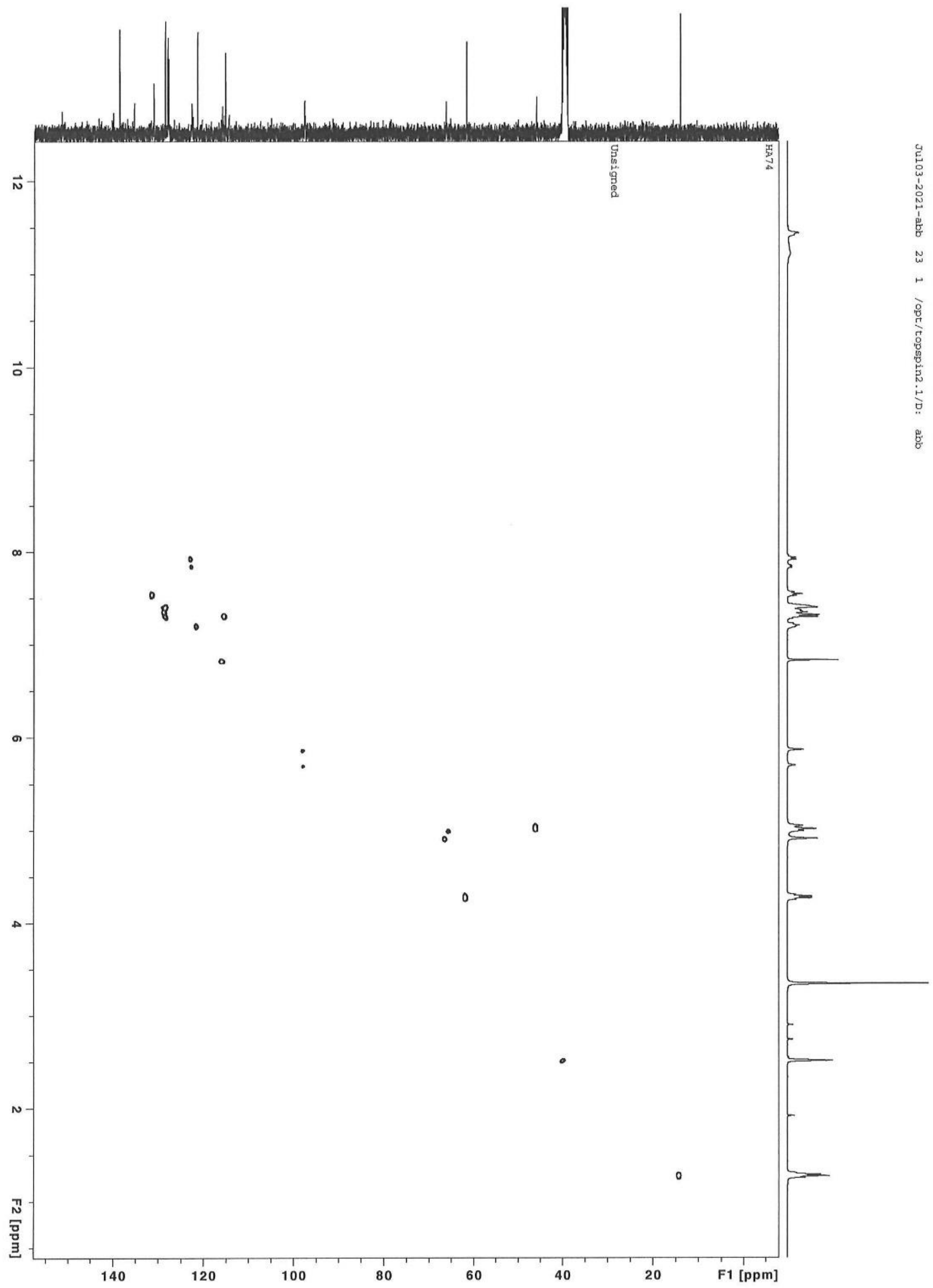

Figure S218. ^{1}H-^{13}C HSQC spectrum of compound **7l.**

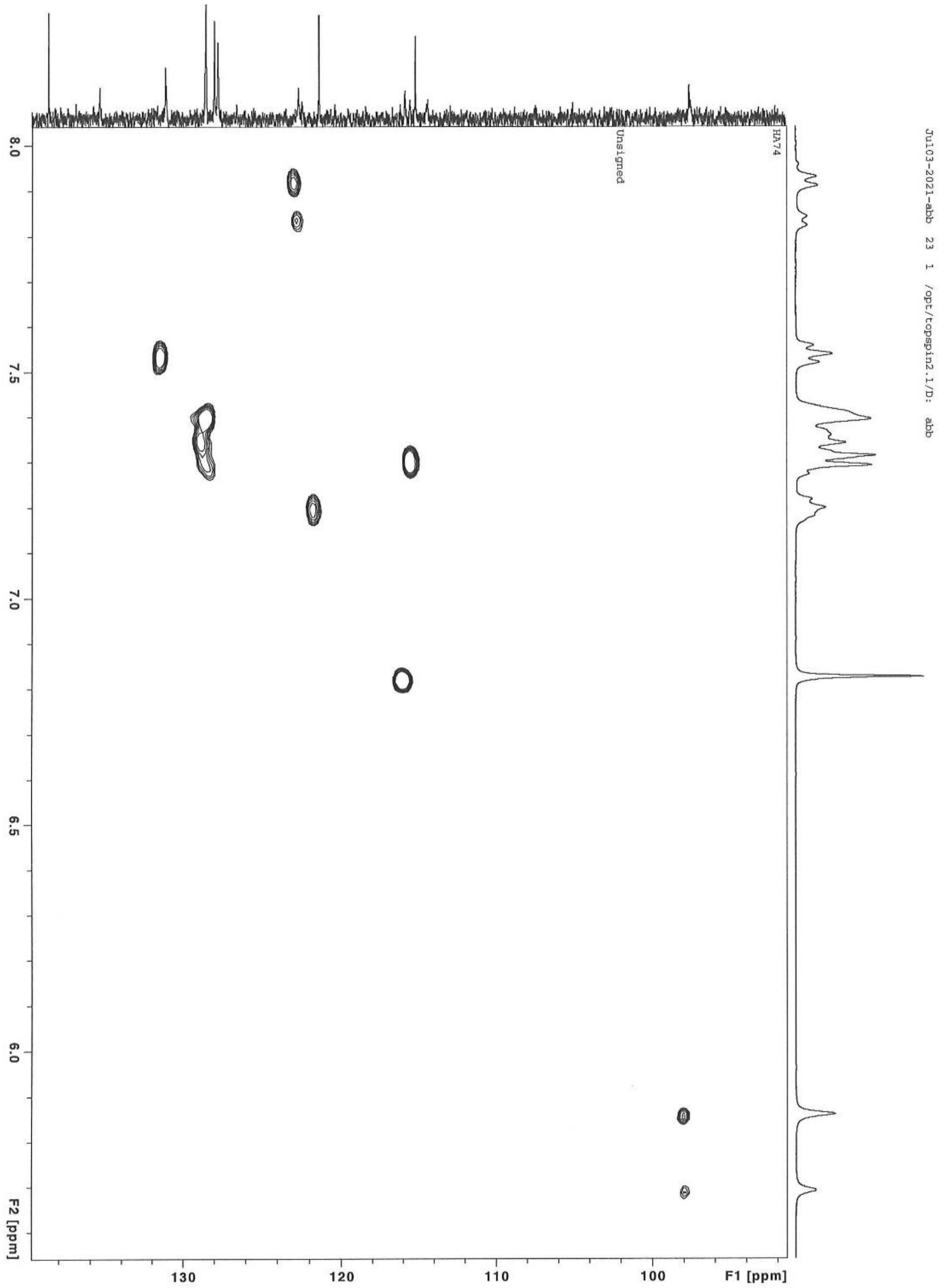

Figure S219. Detail of ^{1}H-^{13}C HSQC spectrum of compound **7l.**

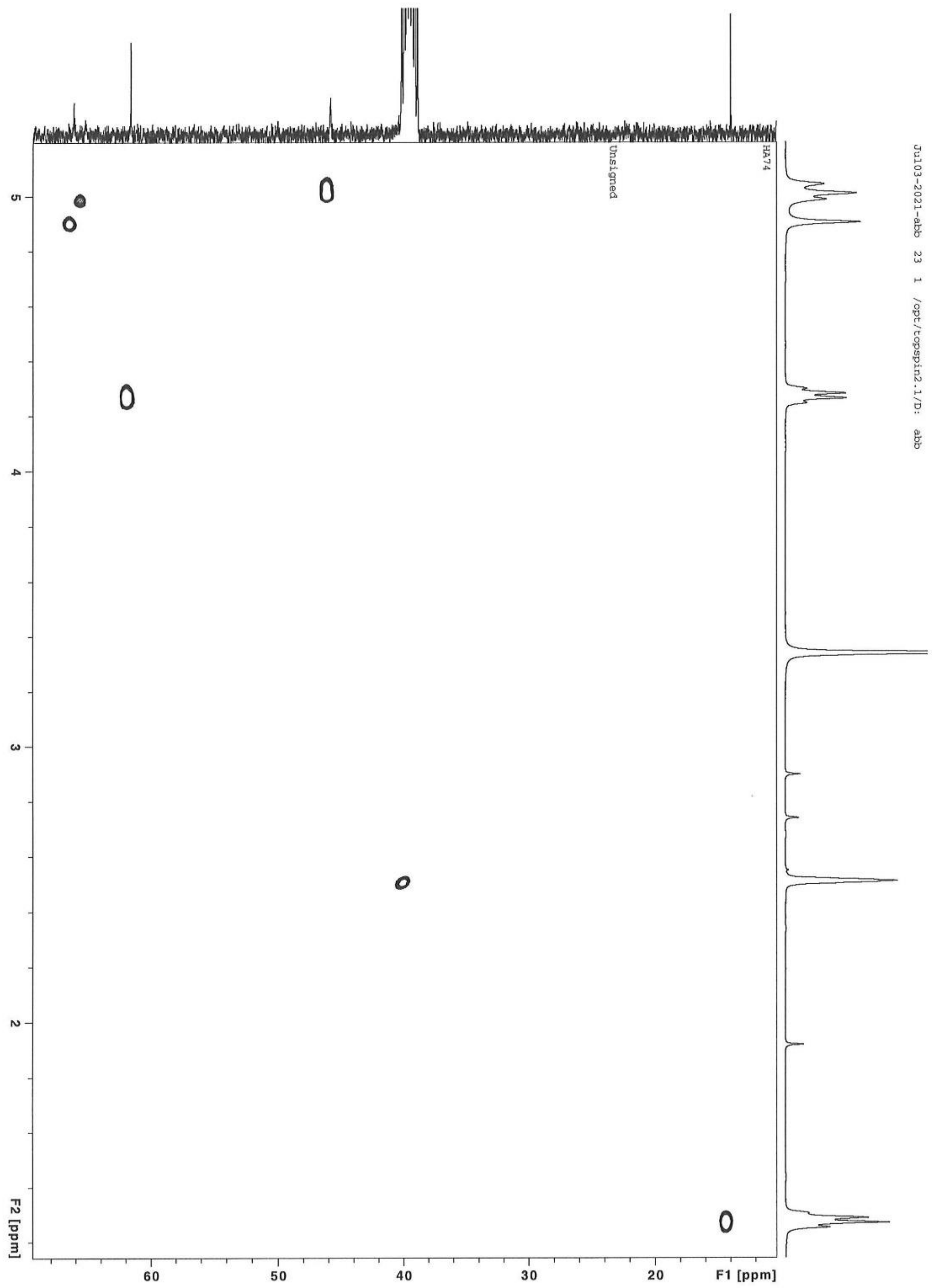

Figure S220. Detail of ^{1}H-^{13}C HSQC spectrum of compound **7l.**

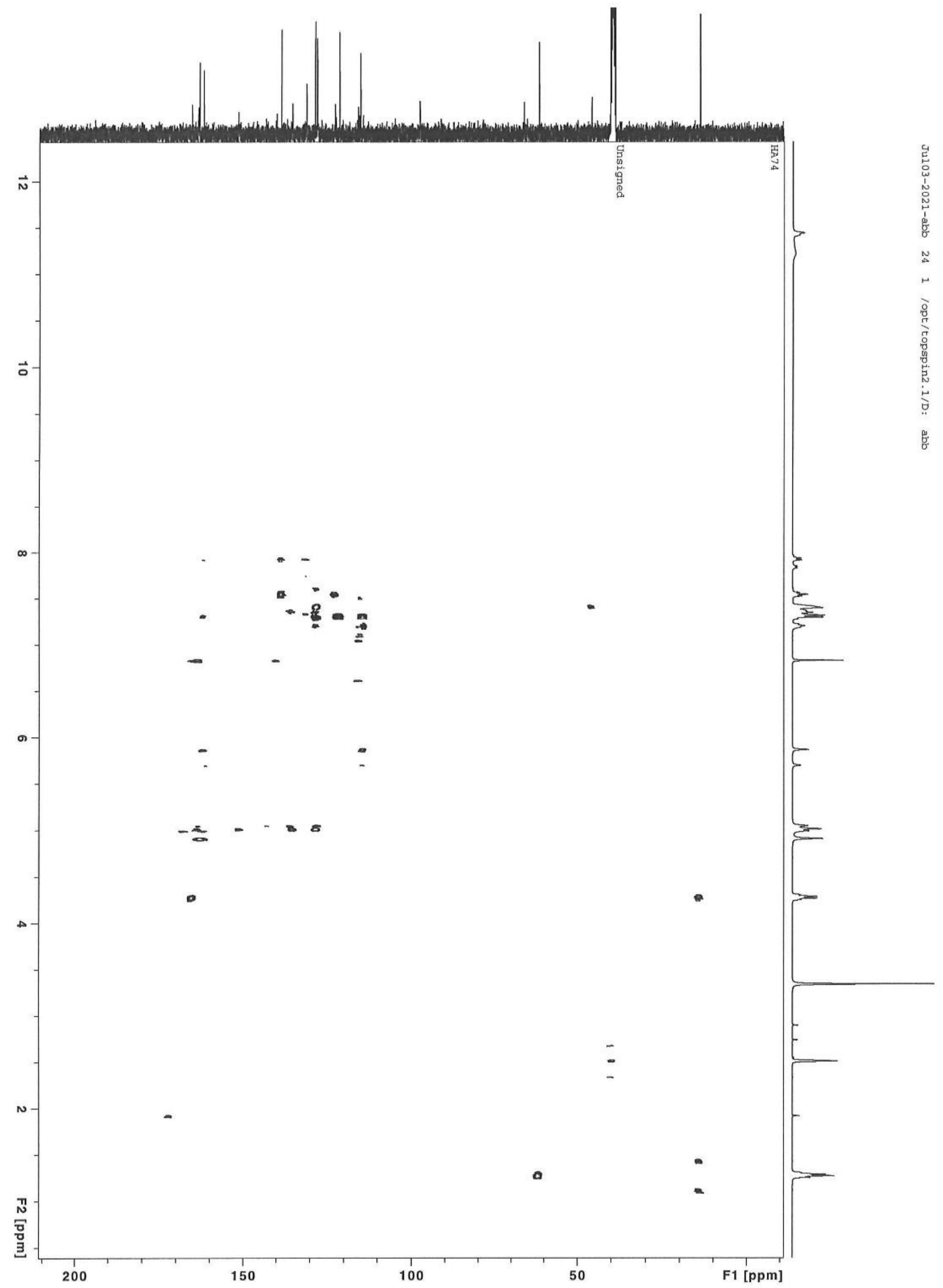

Figure S221. ^{1}H-^{13}C HMBC spectrum of compound **7l.**

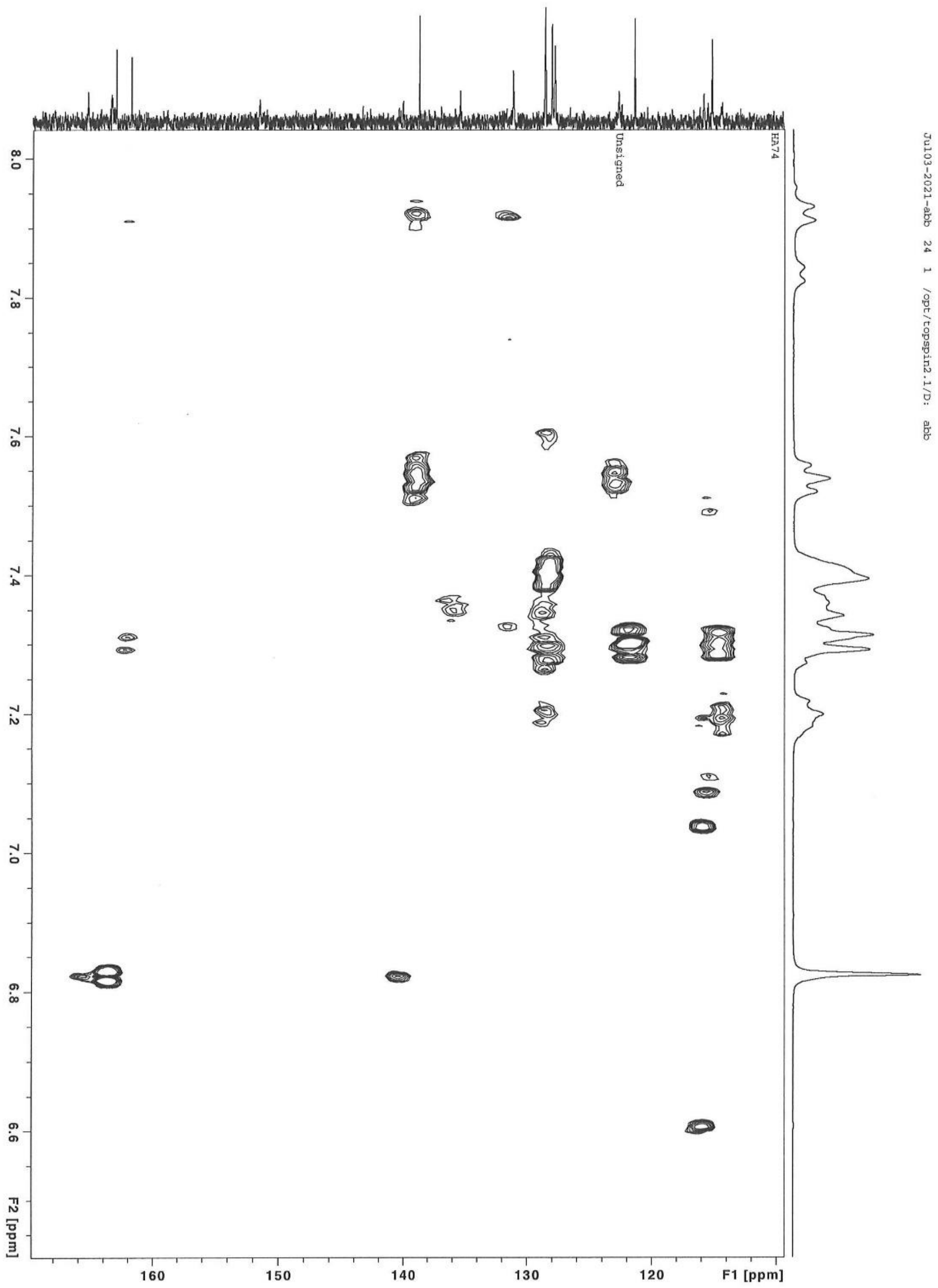

Figure S222. Detail of ^{1}H-^{13}C HMBC spectrum of compound **7l.**

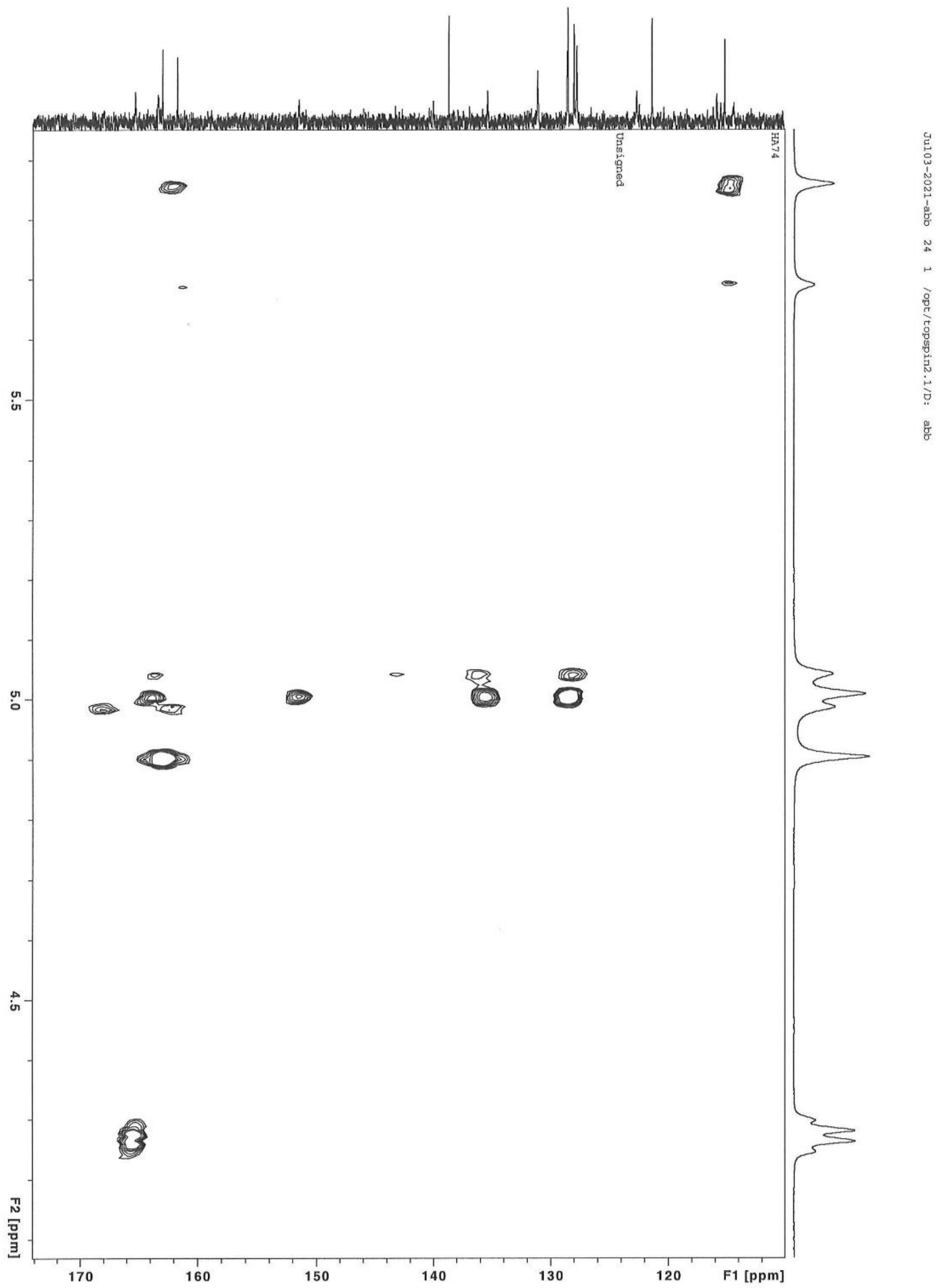

Figure S223. Detail of ^{1}H-^{13}C HMBC spectrum of compound **7l.**

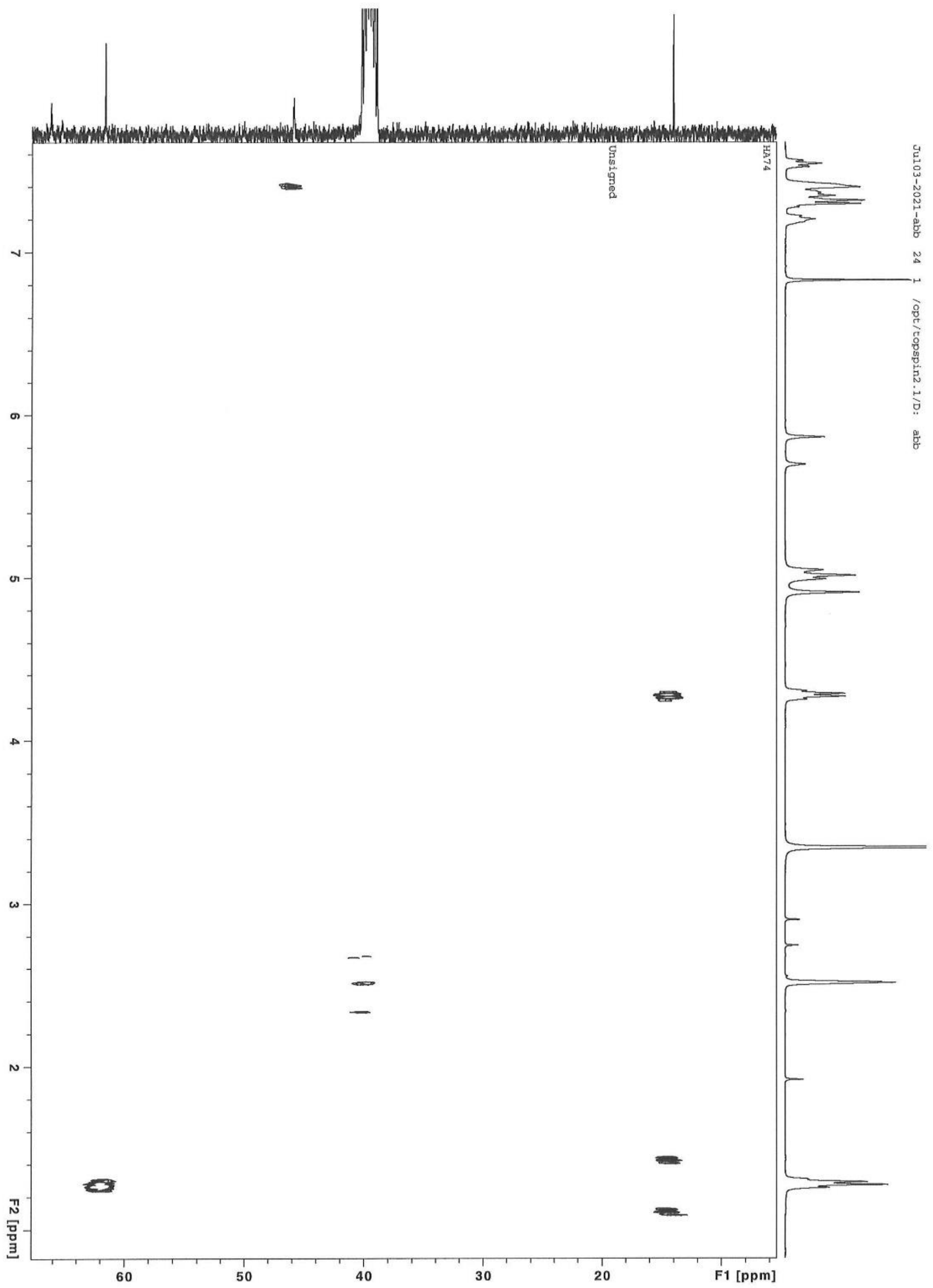

Figure S224. Detail of ^{1}H-^{13}C HMBC spectrum of compound **7l.**

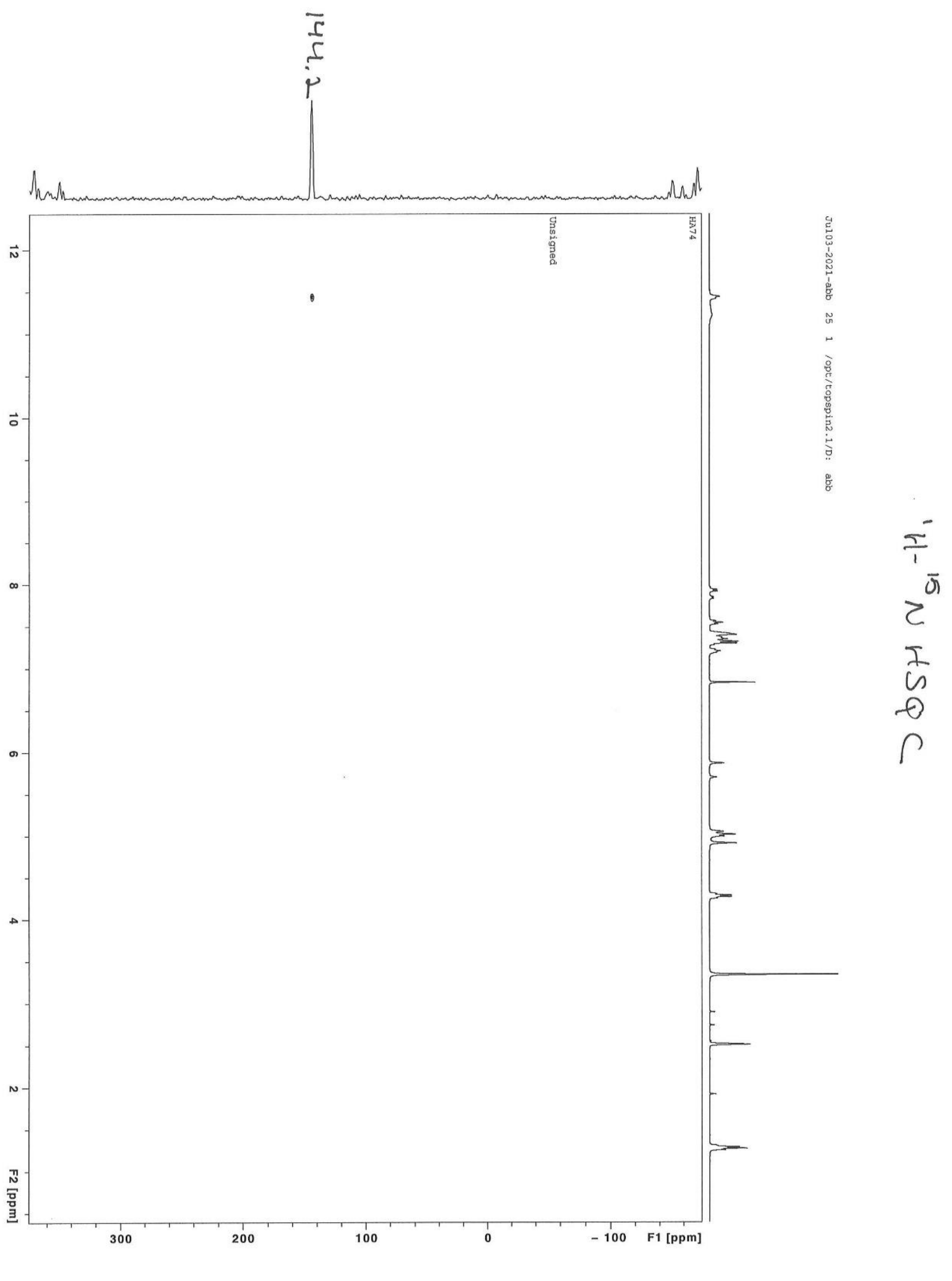

Figure S225. ^{1}H-^{15}N HSQC spectrum of compound **7l.**

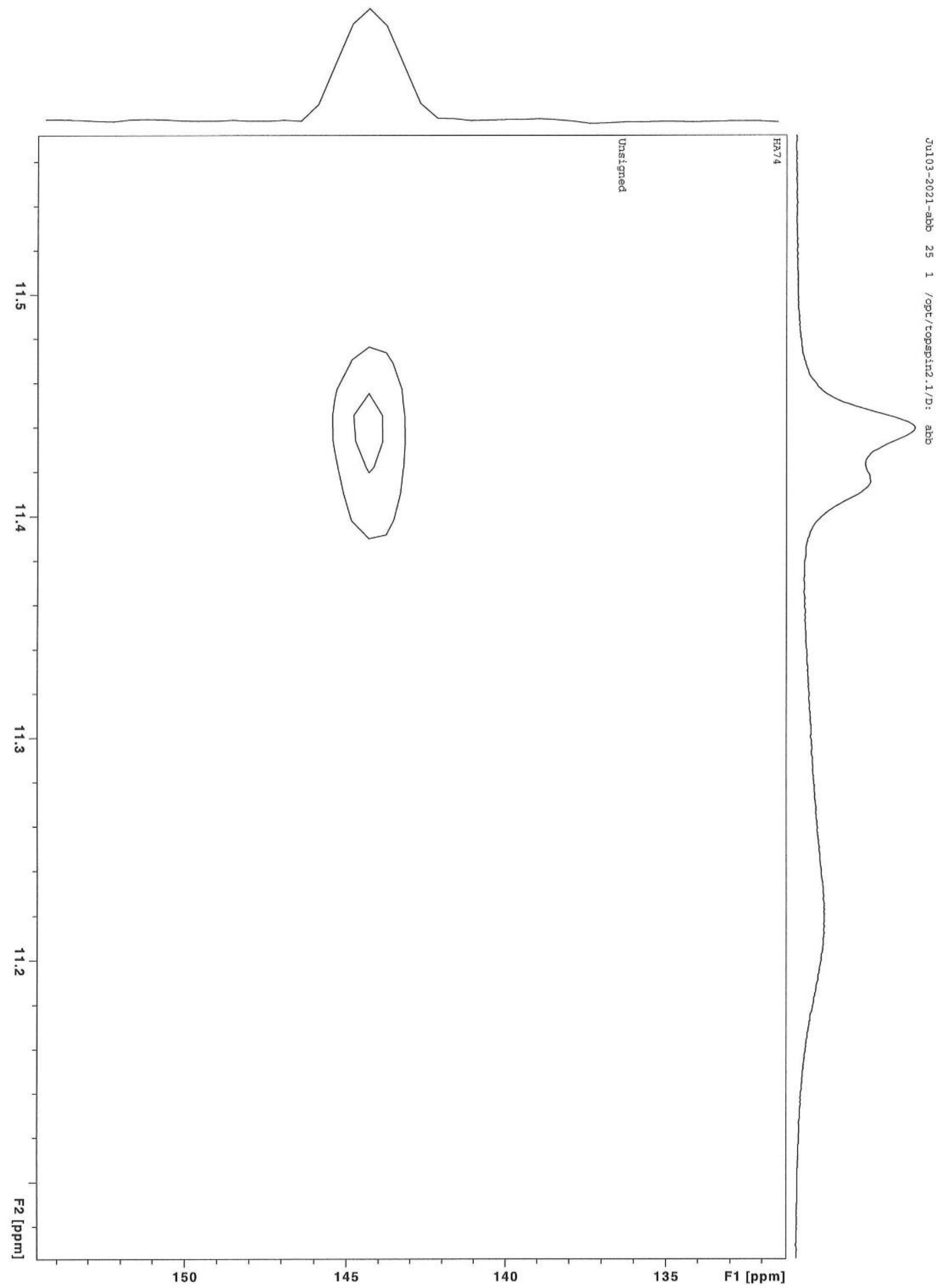

Figure S226. Detail of ^{1}H-^{15}N HSQC spectrum of compound **7l.**

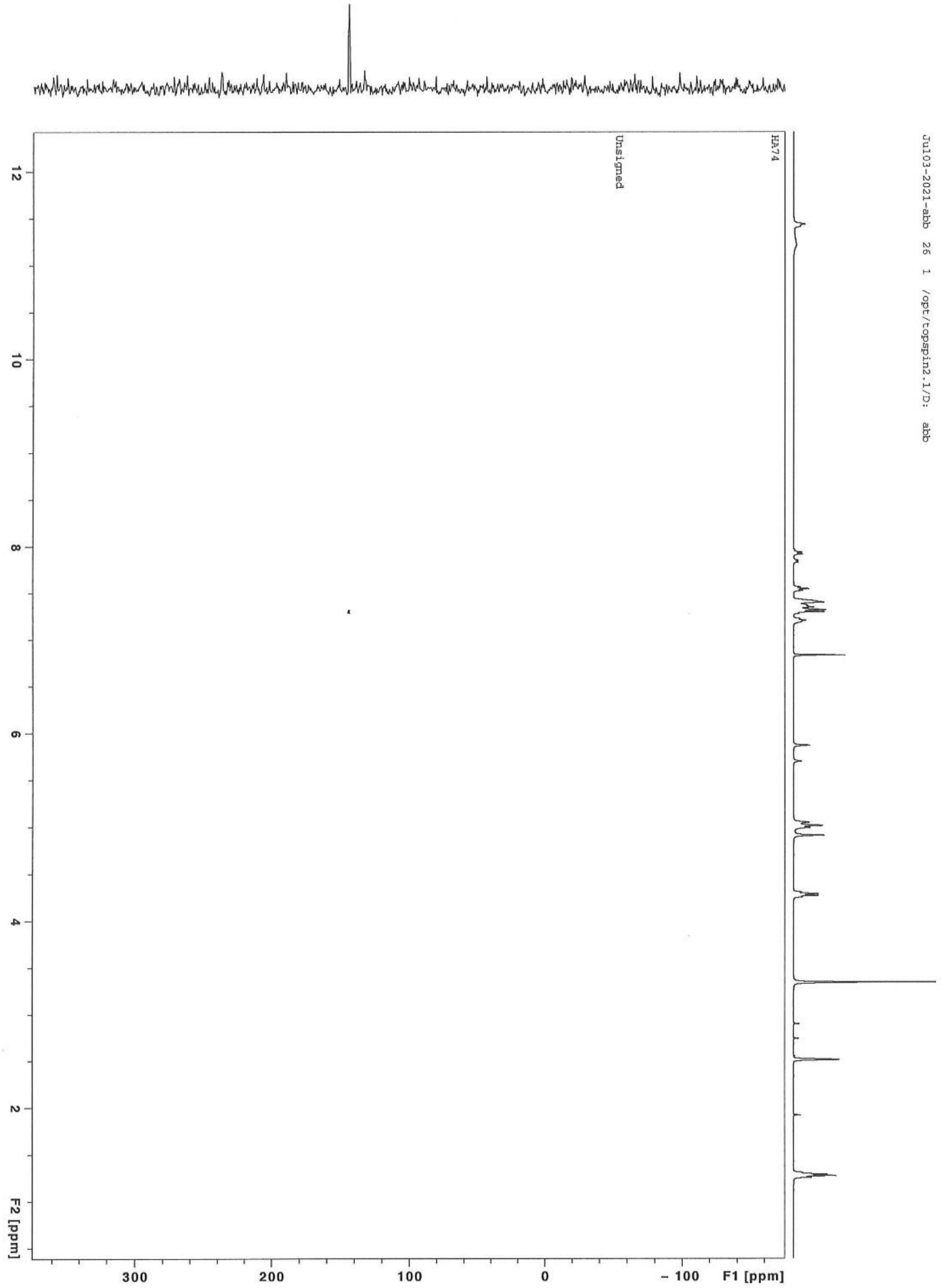

Figure S227. ^{1}H-^{15}N HMBC spectrum of compound **7l.**

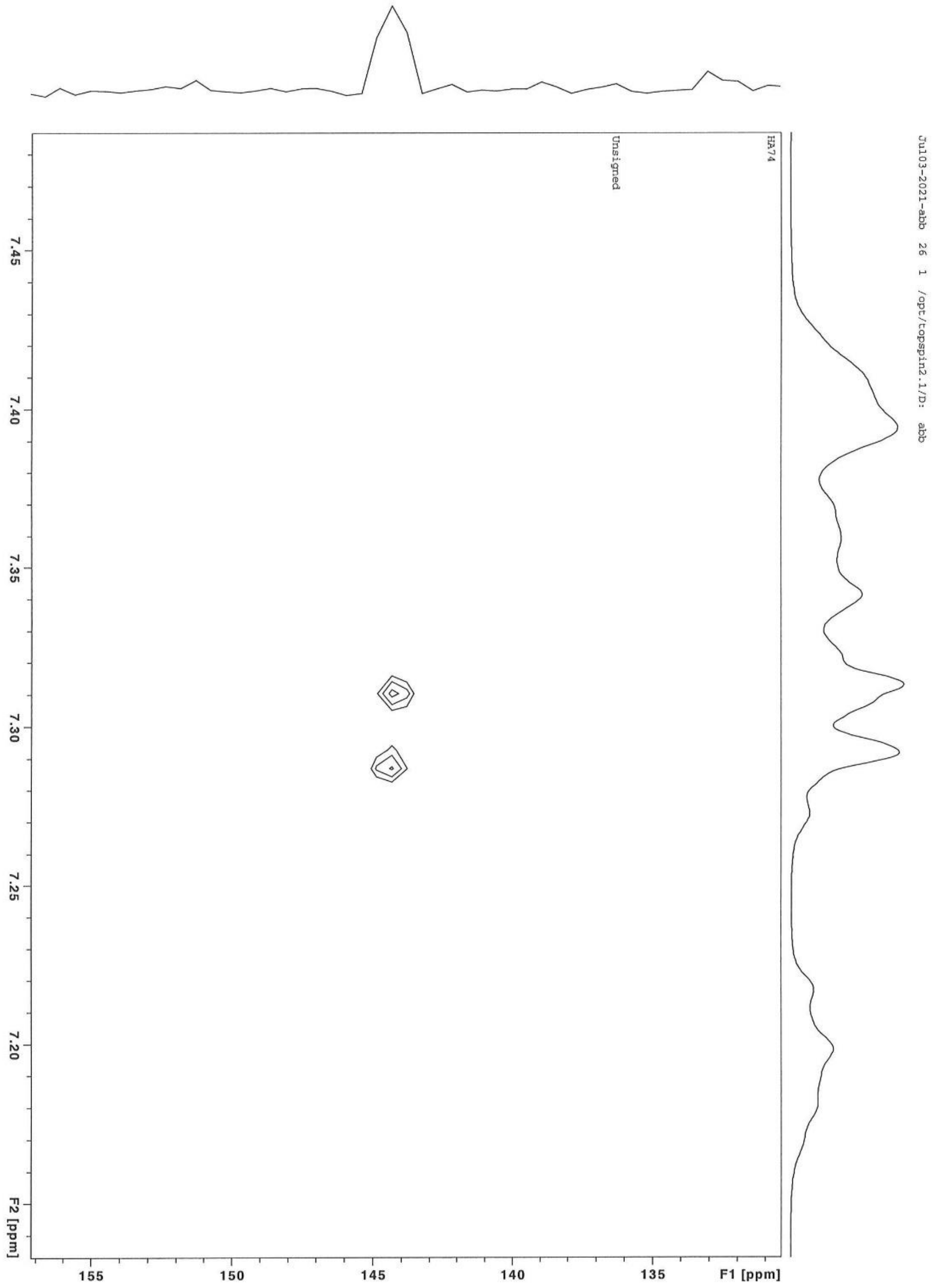

Figure S228. Detail of ^{1}H-^{15}N HMBC spectrum of compound **7l.**
